# Discrimination Between Invasive and *In Situ* Melanomas Using Clinical Close-Up Images and a *De Novo* Convolutional Neural Network

**DOI:** 10.3389/fmed.2021.723914

**Published:** 2021-09-14

**Authors:** Sam Polesie, Martin Gillstedt, Gustav Ahlgren, Hannah Ceder, Johan Dahlén Gyllencreutz, Julia Fougelberg, Eva Johansson Backman, Jenna Pakka, Oscar Zaar, John Paoli

**Affiliations:** ^1^Department of Dermatology and Venereology, Institute of Clinical Sciences, Sahlgrenska Academy, University of Gothenburg, Gothenburg, Sweden; ^2^Department of Dermatology and Venereology, Region Västra Götaland, Sahlgrenska University Hospital, Gothenburg, Sweden

**Keywords:** artificial intelligence, clinical decision-making, melanoma, neural networks, computer, supervised machine learning

## Abstract

**Background:** Melanomas are often easy to recognize clinically but determining whether a melanoma is *in situ* (MIS) or invasive is often more challenging even with the aid of dermoscopy. Recently, convolutional neural networks (CNNs) have made significant and rapid advances within dermatology image analysis. The aims of this investigation were to create a *de novo* CNN for differentiating between MIS and invasive melanomas based on clinical close-up images and to compare its performance on a test set to seven dermatologists.

**Methods:** A retrospective study including clinical images of MIS and invasive melanomas obtained from our department during a five-year time period (2016–2020) was conducted. Overall, 1,551 images [819 MIS (52.8%) and 732 invasive melanomas (47.2%)] were available. The images were randomized into three groups: training set (*n* = 1,051), validation set (*n* = 200), and test set (*n* = 300). A *de novo* CNN model with seven convolutional layers and a single dense layer was developed.

**Results:** The area under the curve was 0.72 for the CNN (95% CI 0.66–0.78) and 0.81 for dermatologists (95% CI 0.76–0.86) (*P* < 0.001). The CNN correctly classified 208 out of 300 lesions (69.3%) whereas the corresponding number for dermatologists was 216 (72.0%). When comparing the CNN performance to each individual reader, three dermatologists significantly outperformed the CNN.

**Conclusions:** For this classification problem, the CNN was outperformed by the dermatologist. However, since the algorithm was only trained and validated on 1,251 images, future refinement and development could make it useful for dermatologists in a real-world setting.

## Introduction

Melanomas are most often easy to recognize and many are spotted instantly even without the aid of dermoscopy. A more challenging task is to determine if a melanoma is *in situ* (MIS) or invasive. Notably, dermatologists are frequently confronted with this specific classification problem, particularly in a preoperative setting. While this issue may seem unimportant since the lesion still requires excision, this binary classification problem adds prognostic value that can be relayed to the patient preoperatively and might even have implications for the selection of the appropriate surgical margins for the first diagnostic excision. The current guidelines suggest an excisional biopsy be performed whenever there is a suspicion of melanoma (1, 2). The histopathological diagnosis will then guide the surgeon to select the appropriate margins. Nonetheless, for MIS, we advocate that the first diagnostic excision should preferably also be the only one needed to provide the cure. Contrarily, if invasive melanoma is the primary suspicion, a narrower excision margin may be selected since a subsequent excision with wider margins and potentially a sentinel node biopsy will be required (3). Finally, predicting if a melanoma is invasive or MIS preoperatively could also have relevance for urgent referral and triaging purposes.

For most cases, suspicion of melanoma is raised with naked eye examination. Dermoscopy is known to increase both the specificity and sensitivity compared to the naked eye examination for pigmented skin lesions (4). Nonetheless, while specific features have been described to be suggestive of MIS and invasive melanomas, respectively (5, 6), relatively few dermoscopic features have proven important to distinguish between these two classes once a decision has been made to remove the lesion.

Recently, machine-learning (ML) algorithms including convolutional neural networks (CNNs) have revolutionized image analysis at an extraordinary pace and have already found multiple applications in many domains of health care (7–9). These algorithms have proven useful in several dermatology investigations such as differentiating between nevi and melanomas as well as for classifying several other types of skin tumors (10–13). Moreover, investigations have also demonstrated the value of these algorithms when they are used in conjunction with human readers (14, 15). Furthermore, dermatologists as well as dermatopathologists are generally positive toward a development with an increased use of ML (16, 17), and patients seem to be optimistic toward artificial intelligence (AI) in skin cancer screening as long as it preserves the integrity of the human doctor-patient relationship (18, 19). While all the above mentioned factors may support its use, broad clinical implementation of ML-derived tools within the field of dermatology is still pending (20). In a previous investigation, we built and evaluated a *de novo* CNN (i.e., model with no pretrained parameters) designed to discriminate between MIS and invasive melanomas using dermoscopic images, which was not outperformed by the dermatologists that were given the same classification problem (21). In a primary health-care setting, the access to dermoscopes is often limited, which means that general practitioners are often limited to evaluation of clinical close-up images.

The aims of this investigation were to create a *de novo* CNN for differentiating between MIS and invasive melanomas based on clinical close-up images and to test performance status of the model compared to seven independent dermatologists from our department.

## Materials and Methods

This retrospective study included clinical images of MIS and invasive melanomas obtained from the department of Dermatology at Sahlgrenska University Hospital during a 5-year time period (2016–2020). Lesions with low quality and lesions that could not be appropriately anonymized were excluded from the analysis. When possible, rotations of the images were performed before cropping to exclude medical rulers or irrelevant background. After exclusion, 1,551 cropped and resized close-up images [819 MIS (52.8%) and 732 invasive melanomas (47.2%)] were available (Supplementary Figure 1). All lesions were histopathologically verified by a dermatopathologist. The images were randomized into three groups: training set (*n* = 1,051), validation set (*n* = 200), and test set (*n* = 300). The proportion of MIS and invasive melanomas over or under 1.0 mm in Breslow thickness as well as the minimum value of width/height resolution of the manually cropped images (0–300, 301–600, >600 pixels) were maintained in each group.

Different CNNs were evaluated on the validation set after each training run on the entire training set (an epoch). The number of convolutional layers in different models varied between 6 and 9 and the depth of each convolutional layer varied from 16 to 256 filters. The kernel size was set to 3 × 3 in all convolutional layers. Each model had between one and five fully connected layers ranging in size from 32 to 128 neurons. A rectified linear unit activation function was used after each convolutional layer. Augmentation (random transformations including variations in brightness, rotations, scalings, and flips) was used in the training set (Supplementary Figure 2). Different models were evaluated with 200 epochs each to see where they reached peak accuracy. This was usually reached after 60–100 epochs. Finally, a model with seven convolutional layers (with depths of 16, 32, 64, 128, 128, 128, and 128 filters) and a single dense layer (size 128) was selected (Supplementary Appendices 1–3). This model achieved an optimal accuracy for the validation set after being trained during 75 epochs (Supplementary Figure 3).

**Figure 1:**
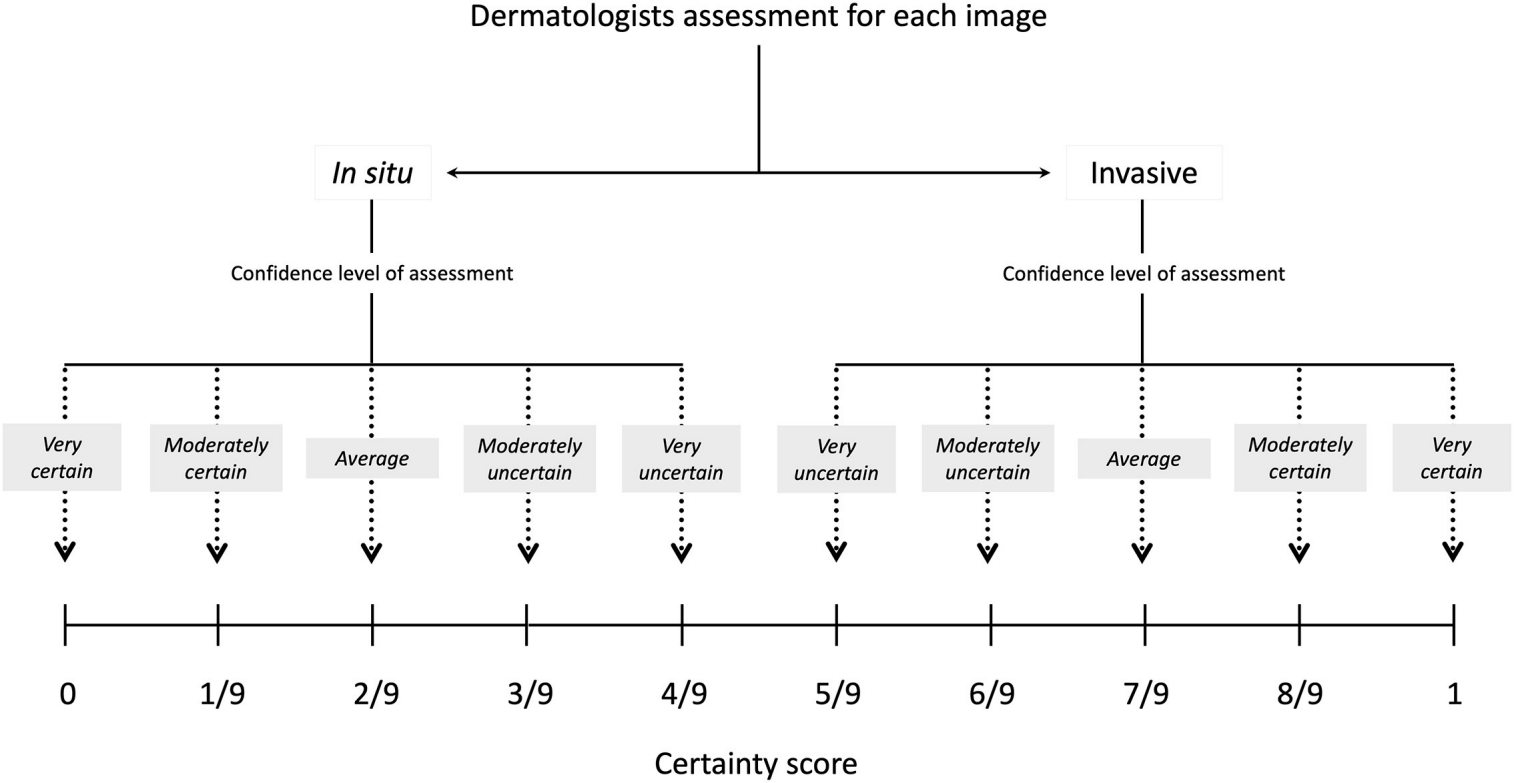
Certainty score. For all image classifications, the dermatologists had to select the degree of certainty. The following scores were available; very certain (*Swe*. “mycket säker”), moderately certain (*Swe*. “ganska säker”), average (*Swe*. “medel”), moderately uncertain (*Swe*. “ganska osäker”), and very uncertain (*Swe*. “mycket osäker”). The same weights were applied for each step (i.e., for every increasing or decreasing one-ninth) in the scoring system.

The final CNN model was then evaluated on the test set. This evaluation was monitored by MG, GA, and SP and these authors all verify that only the selected model was evaluated on the test set. The performance of the model was compared to seven dermatologists (one resident physician and six board-certified dermatologists), who independently reviewed all test set lesions. The dermatologists were given the same images (i.e., 600 × 600 pixels) as the CNN and were required to answer if they thought that the images represented MIS or invasive melanomas. If the reader responded invasive melanoma, a suggestion of estimated Breslow depth (≤ 1.0 mm or >1.0 mm) was required. Finally, for all cases, the readers reported a certainty score relating to their level of confidence in their assessment (MIS/invasive), which enabled generation of individual receiver operating characteristics (ROC) curves for each dermatologist. This score ranged from 1 (very uncertain) to 5 (very certain), which translated into a score with 10 possible values (i.e., 9 intervals) (Figure 1). To restrict the analysis to clinical images only, neither dermoscopic images nor other metadata were made available to the readers. All clinical images in the test set can be accessed in Supplementary Appendix 4. The study was reviewed and approved by the Regional Ethical Review Board in Gothenburg (approval number 283–18).

### Statistical Analysis

All data were analyzed using R version 3.5.3 (https://www.r-project.org/). DeLong's test for two correlated ROC curves was used to compare the performance of dermatologists and the CNN. The exact binomial test was used to compare the two points on the ROC curves of the dermatologists and the CNN, respectively, where the sensitivity and specificity were closest within each curve. The CNN output ranged from 0 to 1 where higher scores indicated invasive melanoma and lower scores indicated MIS. The point on the CNN ROC curve where sensitivity and specificity were closest was considered as the assessment of CNN of whether the melanoma was MIS or invasive. Interobserver agreement between all readers was calculated with Fleiss' kappa (κ) (22, 23). All tests were two-sided and *P* < 0.05 was considered as statistically significant.

## Results

For all included cases (*n* = 1,551), the median age at melanoma diagnosis (interquartile range) was 68 (55–77) years and 53.5% occurred in males. Overall, the test set (*n* = 300) included 158 (52.7%) MIS and 142 (47.3%) invasive melanomas (Table 1). In total, 259 (86.3%) lesions were located on the trunk or the extremities and 41 (13.7%) were located in the head and neck area. The proportion of MIS and invasive melanomas did not differ significantly in these the body regions (*P* = 0.86). The interobserver agreement between the readers in terms of answering MIS or invasive melanomas was moderate (κ = 0.56, 95% CI 0.53–0.58).

**Table 1:** Distribution of melanomas included in the test set.

	**Frequency (%)**
MIS	158 (52.7)
Invasive melanoma	142 (47.3)
≤ 1.0 mm	96 (32.0)
Ulcerated	1
Not ulcerated	95
>1.0 mm	46 (15.3)
Ulcerated	13
Not ulcerated	33

*MIS, melanoma in situ*.

**Figure 2:**
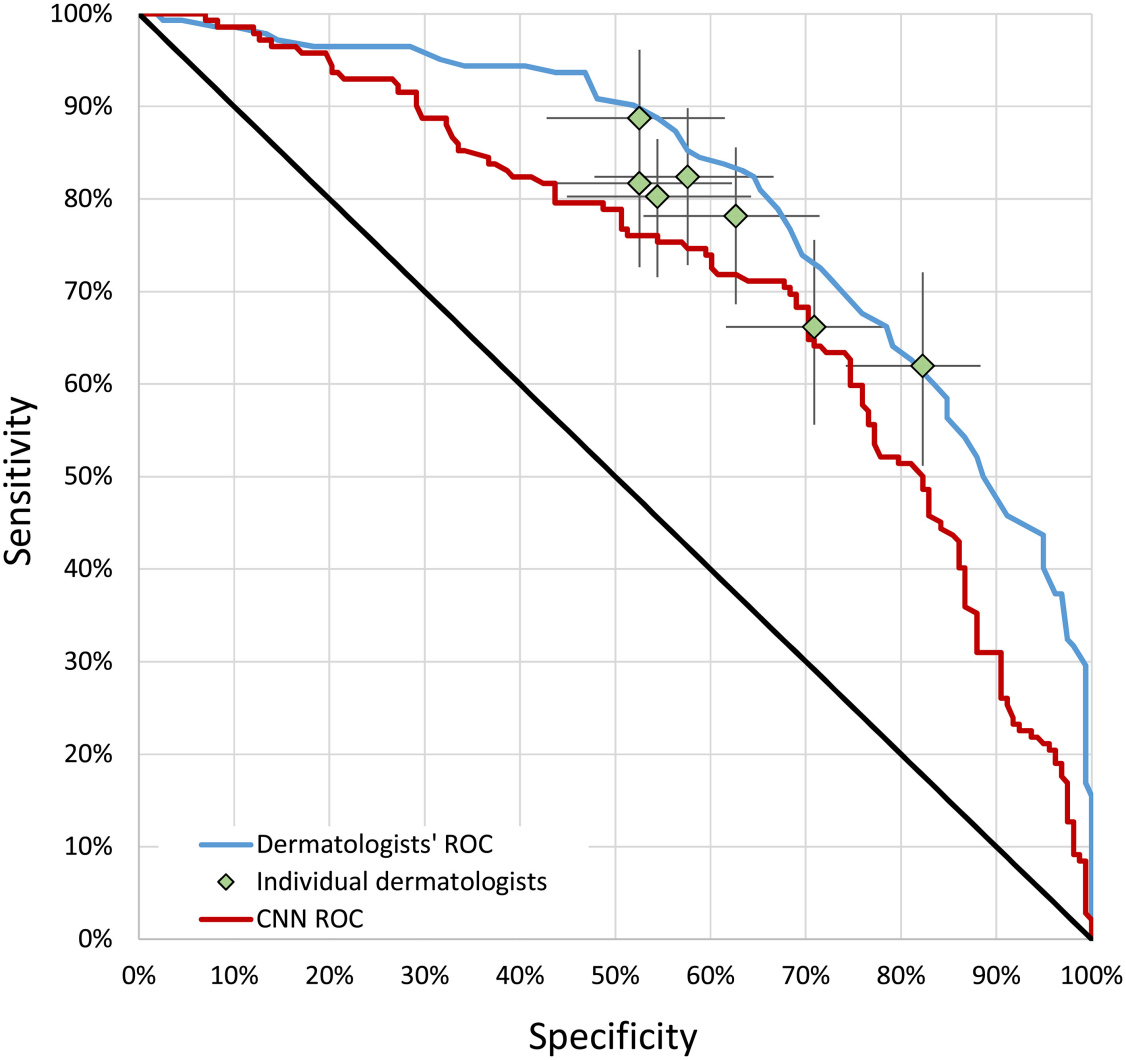
ROC curves. Each point on the figure represents one dermatologist with respect to specificity and sensitivity in terms of correctly classifying a melanoma as invasive. CNN, convolutional neural network; ROC, receiver operating characteristics.

**Figure 3:**
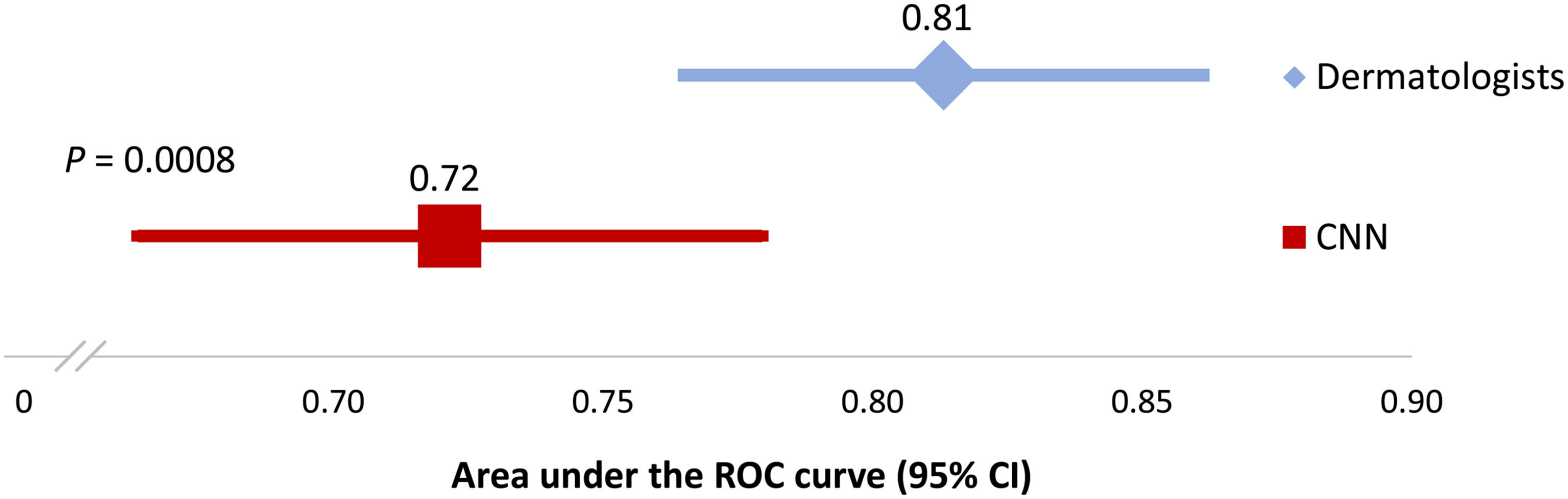
Area under the ROC curve. CNN, convolutional neural network; CI, confidence interval; ROC, receiver operating characteristics.

The ROC curves for the CNN and the combined assessment of dermatologists are presented in Figure 2. The area under the curve (AUC) was 0.72 for the CNN (95% CI 0.66–0.78) and 0.81 for dermatologists (95% CI 0.76–0.86) (*P* < 0.001) (Figure 3). At the points where the sensitivity and specificity were closest within each ROC curve, the CNN correctly classified 208 out of 300 lesions (69.3%), whereas the corresponding number for dermatologists was 216 (72.0%). The answer of CNN was accurate in 34 cases in which the dermatologists were wrong, whereas dermatologists were accurate in 42 cases where the answer of CNN was wrong (*P* = 0.42). For melanomas with a Breslow thickness >1.0 mm, the CNN downgraded 6 out of 46 cases (13.0%) as MIS. The corresponding value for dermatologists was 3 out of 46 cases (6.5%); (*P* = 0.45; exact binomial test) (Table 2). There was no difference in accuracy rates when assessing lesions located on the trunk, extremities, and in the head and neck for the CNN (*P* = 0.60) or the dermatologists (*P* = 0.34).

**Table 2:** Breakdown of incorrectly upgraded and downgraded lesions.

	**HDC**		
	***MIS***	**Invasive ≤1.0 mm**	**Invasive >1.0 mm**
CNN downgraded vs. HDC	–	37 (39%)	6 (13%)
CNN equal to HDC	109 (69%)	59 (61%)	40 (87%)
CNN upgraded vs. HDC	49 (31%)	–	–
Total	158	96	46
Dermatologists downgraded vs. HDC	–	36 (38%)	3 (7%)
Dermatologists equal to HDC	113 (72%)	60 (63%)	43 (93%)
Dermatologists upgraded vs. HDC	45 (28%)	–	-
Total	158	96	46
CNN downgraded vs. Dermatologists^*^	21 (13%)	12 (13%)	5 (11%)
CNN equal to Dermatologists^*^	112 (71%)	73 (76%)	39 (85%)
CNN upgraded vs. Dermatologists^*^	25 (16%)	11 (11%)	2 (4%)
Total	158	96	46

*Downgraded = diagnosed as MIS instead of invasive, upgraded = diagnosed as invasive instead of MIS*.

* *There was no significant difference (P = 0.29; Fisher's exact test) in distribution between the three histopathological diagnostic categories and whether AI < Dermatologist, AI = Dermatologist, or AI > Dermatologist. Also, there was no significant difference (P = 0.15; Fisher's exact test) in distribution between the three histopathological diagnostic categories and whether AI = Dermatologist or AI ≠ Dermatologist. CNN, convolutional neural network; HDC, histopathological diagnostic categories; MIS, melanoma in situ*.

**Figure 4:**
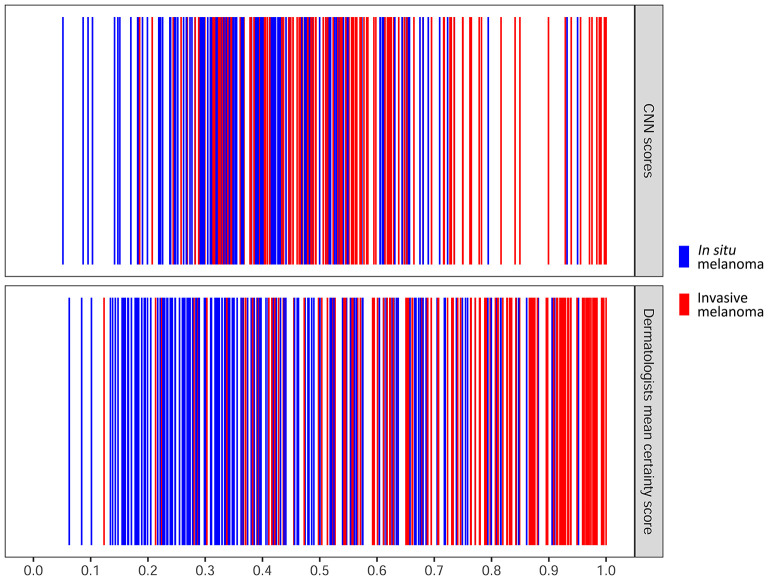
Distribution map of dermatologists and CNN output of all cases. Each individual line represents one case. Higher scores indicated that dermatologists and the CNN considered the lesion to be invasive with a higher degree of certainty. The x-axis for the dermatologists represents the mean of certainty scores of all the seven dermatologists ranging from 0 to 1. The x-axis for the CNN represents the output score of CNN ranging from 0 to 1. CNN, convolutional neural network.

When comparing the CNN performance to each individual reader, three dermatologists significantly outperformed the CNN (Table 3) (Supplementary Figure 4). For lesions that were invasive, the mean certainty score of dermatologists was more often closer to 1 compared to the CNN (Figure 4) (Supplementary Figures 5, 6).

**Table 3:** Comparison of AUC achieved by the CNN, the dermatologists combined, and each dermatologist separately.

			**95 % CI**		
		**AUC**	**lower**	**upper**	***P*-value**
Dermatologists combined		0.81	0.76	0.86	<0.001
CNN		0.72	0.66	0.78	
Reader 1	vs. CNN	0.78	0.73	0.83	0.037
Reader 2		0.78	0.73	0.83	0.060
Reader 3		0.78	0.72	0.83	0.081
Reader 4		0.80	0.75	0.85	0.003
Reader 5		0.78	0.73	0.83	0.044
Reader 6		0.75	0.70	0.81	0.30
Reader 7		0.77	0.71	0.82	0.12

*CI, confidence interval; CNN, convolutional neural network*.

When the certainty score was used to produce the combined assessments of dermatologists, the ROC yielded a significantly higher AUC compared to a corresponding AUC where no consideration was taken to the degree of certainty (i.e., dichotomous answers, 0 = MIS; 1 = invasive melanoma) (Supplementary Figure 7). Compared to the combined AUC of the seven readers alone, addition of the CNN output generated a slightly higher AUC (0.81 vs. 0.82) albeit without statistical significance (*P* = 0.29).

Finally, eight lesion images (four in which the CNN was correct and the dermatologists were wrong and four in which the dermatologists were correct and the CNN was wrong) were chosen for which the CNN and dermatologists had maximum disagreement (i.e., the discrepancy between the scores of CNN and dermatologists was as high as possible). For these cases, class activation maps were performed to highlight aspects of the images that were important for the CNN output (Supplementary Figure 8).

## Discussion

In this investigation, the combined response of the seven readers performed better in terms of classification of MIS and invasive melanomas than the *de novo* CNN. Three out of seven dermatologists significantly outperformed the CNN.

In a recent investigation, we evaluated another *de novo* CNN trained on 749 dermoscopic images using the same classification problem. When the model was evaluated on a test set (*n* = 200 images), there was no statistical difference in AUC between the combined score of dermatologists and the CNN (21). Interestingly, the AUCs for dermatologists and the CNN in this investigation aligned well-with the values in the former investigation on dermoscopic images. Although close-up clinical images and dermoscopic images are complementary in a clinical situation, it is not absolutely certain that using both image modalities in the preoperative setting will result in a better score compared to any of the techniques alone. For example, a clinical image entails more details on the surrounding skin as well as whether a lesion is raised allowing the dermatologist to make a more global assessment. Dermoscopic images, on the other hand, offer a higher resolution of features within the lesion itself. In this context, it is important to remember that relatively few dermoscopic features have been described that help clinicians to differentiate a MIS from invasive melanomas (5, 6). Using this line of argument, it is interesting that the readers in this investigation performed in par with our previous investigation when we limited the analysis to dermoscopic images alone.

In a previous publication by Tschandl et al. CNNs were trained and validated on clinical close-up and dermoscopic images, respectively, for non-pigmented skin lesions. The aim was to predict the correct malignancy status (i.e. benign vs. malignant) (13). For melanoma images in the test set (*n* = 35), the dermoscopy CNN performed better than the close-up CNN (50.5 vs. 22.9% correct classifications). However, for nevi (*n* = 73), the close-up CNN performed better than the dermoscopy CNN (79.4 vs. 69.8%). While this investigation does not easily compare to the results presented here and only included non-pigmented lesions, it is still a reminder that, as for physicians, clinical and dermoscopic images are useful in different settings. In upcoming investigations, it would be interesting to compare our CNN output for dermoscopic and clinical images. It is possible that a higher sensitivity and specificity can be obtained if the output of these two CNNs can be interpreted together.

While there are melanomas that are undoubtedly invasive, in many cases, this classification problem is often challenging even with access to both dermoscopic images as well as clinical ones (24–26). The problem gets even more challenging since most melanomas are detected early either as MIS or thin invasive lesions. Noteworthily, in this investigation, we introduced a new concept of using a certainty score and demonstrated its use in term of human readers. For the combined output of dermatologists, including this type of score generated a significantly higher AUC compared to a situation where no consideration was given to the level of certainty. Although this score may seem contrived, and the fact that we applied the same weight for all steps in the scoring system, we are confident that most colleagues can relate to varying levels of confidence influencing our clinical decisions. Consequently, to better imitate the clinical setting for other binary classification problems, we suggest other researchers to include a similar certainty score.

In an investigation by Fujisawa et al. a preconditioned CNN was trained on a relatively small data set of clinical images consisting of 4,867 lesions including 458 pigmented and 51 non-pigmented melanomas. The model was then tested on 1,142 images including 82 pigmented melanomas and 13 non-pigmented melanomas (27). In the second-level classification, the CNN could select from any of the following four labels: malignant epithelial tumor, malignant melanocytic tumor (i.e., melanoma), benign epithelial tumor, and benign melanocytic tumor. The accuracy rate for melanomas was 73% (69 correctly classified out of 95), whereas the corresponding figure for benign melanocytic lesions was 90.9% (299 correctly classified out of 329). Nonetheless, most included melanomas (52.6%) were of acral type, which is rare in a Nordic setting. Moreover, it is unclear if the melanoma group also included MIS.

Limitations of this investigation include the retrospective design, the low number of readers, and the artificial setup where relevant metadata and dermoscopic images were intentionally omitted. While the CNN model described in this study included 1,051 and 200 images in the training and validation set, studies involving many more patient images are clearly needed to determine whether this method is better for differentiating between MIS and invasive melanoma compared to a dermatologist. It is likely that the algorithm output will improve when including more images. As such, this investigation should be regarded as a proof-of-concept. The images included come from patients with Nordic skin types with a certain distribution of melanoma thicknesses and appearances, which must also be considered in regard to its reproducibility.

Moreover, only a limited amount of surrounding skin was made available in the clinical close-up images, making it hard to evaluate the degree of sun damage and other pigmented lesions in the surrounding skin. In reality, dermatologists possibly deploy an automatic comparable approach in this classification problem. Moreover, future inclusion of relevant metadata in studies assessing new CNNs for melanoma diagnosis will most likely be of significance. In a real-life situation, for example, palpation of the lesion is important in the preoperative setting when estimating the possible Breslow thickness of a melanoma. This clinical finding might also be added in future prospective investigations. Finally, the melanomas in this investigation principally emerged in patients with fair skin (i.e., Fitzpatrick skin types ranging from 1 to 3) and in non-acral, and non-facial skin.

In our investigation, images with imperfections such as surgical markings were not excluded. Other studies have shown that skin markings can interfere with CNN output. In a model set out to differentiate nevi and melanomas, several benign nevi were upgraded if there were adjacent skin markings resulting in a significant drop in specificity (28). However, since the lesions in our data set all required excision and did not include any benign lesions, this was probably less of a problem. Although there is a theoretical risk that more suspicious looking lesions (i.e., thick melanomas) might be outlined with a surgical marker to a larger extent than MIS, we do not believe dermatologists are more likely to mark out invasive melanomas more often than any other melanocytic lesion once a decision has been made to remove it. It is also important to train and validate CNNs on imperfect and annotated images that reflect real-word data.

It is very likely that ML-derived tools eventually will find their way into clinical practice, but we must be wise in selecting the appropriate setting for these algorithms to increase the sensitivity and specificity for selected clinical classification problems. The ultimate aim of developing new algorithms must be to improve human intelligence and the physician-patient relationship rather than replace it. Specifically, ML algorithms will most likely be used in conjunction with the human dermatologist as a support system (i.e., augmented intelligence) (29–31). Also, creating tools that can help primary health-care physicians recognize MIS and invasive melanomas without access to dermoscopy might be useful for our patients in terms of urgent referral and triaging purposes.

In upcoming investigations, we intend to set up an algorithm that includes both clinical and dermoscopic images and to evaluate if it may serve useful for dermatologists. Needless to say, to critically evaluate the clinical transferability of this application, prospective evaluation is essential and the interplay between algorithm developers and dermatologists is instrumental when codesigning these future applications.

To summarize, the *de novo* CNN developed in this study was slightly outperformed by the combined dermatologist assessment in discrimination between MIS and invasive melanomas using clinical close-up images. Future updates and refinements of the algorithm are necessary along with prospective trials to evaluate its potential in a clinical setting.

## Data Availability Statement

The test dataset for this study can be found in the Supplementary Material. Further inquiries can be directed to the corresponding author.

## Ethics Statement

The studies involving human participants were reviewed and approved by Regional Ethical Review Board in Gothenburg (approval number 283-18). Written informed consent for participation was not required for this study in accordance with the national legislation and the institutional requirements.

## Author Contributions

SP: conceptualization-lead, data curation-supporting, formal analysis-equal, investigation-lead, methodology-supporting, project administration-lead, supervision-lead, validation-equal, visualization-supporting, writing-original draft-lead, and writing-review and editing-lead. MG: conceptualization-supporting, data curation-lead, formal analysis-equal, investigation-supporting, methodology-lead, software-lead, validation-equal, visualization-lead, writing-original draft-supporting, and writing-review and editing-supporting. GA: data curation-supporting, investigation-supporting, validation-supporting, writing-original draft-supporting, and writing-review and editing-supporting. HC, JD, JF, EJ, JeP, and OZ: investigation-supporting, writing-original draft-supporting, and writing-review and editing-supporting. JoP: conceptualization-supporting, data curation-supporting, funding acquisition-lead, investigation-supporting, methodology-supporting, resources-lead, supervision-supporting, validation-supporting, writing-original draft-supporting, and writing-review and editing-supporting. All authors contributed to the article and approved the submitted version.

## Funding

The study was financed by grants from the Swedish state under the agreement between the Swedish government and the county councils, the ALF-agreement (ALFGBG-728261).

## Conflict of Interest

The authors declare that the research was conducted in the absence of any commercial or financial relationships that could be construed as a potential conflict of interest.

## Publisher's Note

All claims expressed in this article are solely those of the authors and do not necessarily represent those of their affiliated organizations, or those of the publisher, the editors and the reviewers. Any product that may be evaluated in this article, or claim that may be made by its manufacturer, is not guaranteed or endorsed by the publisher.
