# Supplementary material for: Discrimination Between Invasive and In Situ Melanomas Using Clinical Close-Up Images and a De Novo Convolutional Neural Network
Source: Front Med (Lausanne). 2021 Sep 14;8:723914. doi: 10.3389/fmed.2021.723914 (PMC8476836; doi:10.3389/fmed.2021.723914)
Supplement: Supplementary file 1 [file Data_Sheet_1.docx]

Supplementary Material

# Supplementary Appendix 1

R source code for model definition and training

target_size <- c(600, 600)

cat_list <- c("In_situ", "Invasiv")

###### DATA AUMENTATION FOR TRAINING SET ######

train_data_gen_AUG <- image_data_generator(

rescale = 1/255,

rotation_range = 360,

width_shift_range = 0.2,

height_shift_range = 0.2,

shear_range = 0.2,

zoom_range = 0.2,

horizontal_flip = TRUE,

vertical_flip = TRUE,

fill_mode = "nearest",

brightness_range = c(0.8, 1.2) # 1 is default. <1 darkens, >1 brightens

)

validation_data_gen <- image_data_generator(

rescale = 1/255

)

###### DEFINE BATCH SIZES FOR TRAINING AND VALIDATION SET ######

train_image_array_gen_AUG <- flow_images_from_directory(train_image_files_path,

train_data_gen_AUG,

target_size = target_size,

classes = cat_list,

batch_size = 10,

class_mode = "binary")

validation_image_array_gen <- flow_images_from_directory(validation_image_files_path,

validation_data_gen,

target_size = target_size,

classes = cat_list,

batch_size = 10,

class_mode = "binary")

########### MODEL DEFINITION #############

model <- keras_model_sequential() %>%

layer_conv_2d(filters = 16, kernel_size = c(3, 3), activation = "relu",

input_shape = c(600, 600, 3)) %>%

layer_max_pooling_2d(pool_size = c(2, 2)) %>%

layer_conv_2d(filters = 32, kernel_size = c(3, 3), activation = "relu") %>%

layer_max_pooling_2d(pool_size = c(2, 2)) %>%

layer_conv_2d(filters = 64, kernel_size = c(3, 3), activation = "relu") %>%

layer_max_pooling_2d(pool_size = c(2, 2)) %>%

layer_conv_2d(filters = 128, kernel_size = c(3, 3), activation = "relu") %>%

layer_max_pooling_2d(pool_size = c(2, 2)) %>%

layer_conv_2d(filters = 128, kernel_size = c(3, 3), activation = "relu") %>%

layer_max_pooling_2d(pool_size = c(2, 2)) %>%

layer_conv_2d(filters = 128, kernel_size = c(3, 3), activation = "relu") %>%

layer_max_pooling_2d(pool_size = c(2, 2)) %>%

layer_conv_2d(filters = 128, kernel_size = c(3, 3), activation = "relu") %>%

layer_max_pooling_2d(pool_size = c(2, 2)) %>%

layer_flatten() %>%

layer_dropout(rate = 0.5) %>%

layer_dense(units = 128, activation = "relu") %>%

layer_dense(units = 1, activation = "sigmoid")

############# COMPILE THE MODEL #############

model %>% compile(

loss = "binary_crossentropy",

optimizer = optimizer_rmsprop(lr = 1e-4),

metrics = c("acc")

)

############# TRAIN THE MODEL #############

history <- model %>% fit_generator(

train_image_array_gen_AUG,

steps_per_epoch = 106,

epochs = 75,

validation_data = validation_image_array_gen,

validation_steps = 20

)

# Supplementary Appendix 2

Model summary

Model: "sequential_13"

Layer (type) Output Shape Param #

conv2d_84 (Conv2D) (None, 598, 598, 16) 448

max_pooling2d_84 (MaxPooling2D) (None, 299, 299, 16) 0

conv2d_85 (Conv2D) (None, 297, 297, 32) 4640

max_pooling2d_85 (MaxPooling2D) (None, 148, 148, 32) 0

conv2d_86 (Conv2D) (None, 146, 146, 64) 18496

max_pooling2d_86 (MaxPooling2D) (None, 73, 73, 64) 0

conv2d_87 (Conv2D) (None, 71, 71, 128) 73856

max_pooling2d_87 (MaxPooling2D) (None, 35, 35, 128) 0

conv2d_88 (Conv2D) (None, 33, 33, 128) 147584

max_pooling2d_88 (MaxPooling2D) (None, 16, 16, 128) 0

conv2d_89 (Conv2D) (None, 14, 14, 128) 147584

max_pooling2d_89 (MaxPooling2D) (None, 7, 7, 128) 0

conv2d_90 (Conv2D) (None, 5, 5, 128) 147584

max_pooling2d_90 (MaxPooling2D) (None, 2, 2, 128) 0

flatten_13 (Flatten) (None, 512) 0

dropout_13 (Dropout) (None, 512) 0

dense_26 (Dense) (None, 128) 65664

dense_27 (Dense) (None, 1) 129

Total params: 605,985

Trainable params: 605,985

Non-trainable params: 0

# Supplementary Appendix 3

Software and Hardware:

The Keras library (version 2.3.1) using the Tensorflow backend (version 1.14.0) was used running on Python version 3.6.9. Model construction was done using R version 3.5.3 (https://www.r-project.org/) and the R-package Keras was used to call Python and its above libraries. All images were manually cropped to only include the tumor using Microsoft Paint version 1909 (Microsoft, Redmond, WA, USA). When necessary, Photoshop CS4 version 11.0 (Adobe Inc., San Jose, CA, USA) was used to rotate images before cropping. XnView version 2.20 was used to scale and crop all images to quadratic shape and a final resolution of 600x600 pixels, preserving the aspect ratio of the original image. All images were converted from JPEG to PNG-format. A 24-bit color depth was used (3 RGB channels with 8 bits in each channel). The computer running the training was using the GPU version on the Keras/Tensorflow routines. The graphics card used was a Nvidia Geforce GTX 1070 with 8 GB GPU memory using CUDA version 10.0 and cudnn version 7.6.3.30. The processor used was an Intel Core i5-2400 @ 3.10 GHz and the amount of RAM was 24 GB. The training of the final model (75 epochs) took 2 hours and 57 minutes.

# Supplementary Appendix 4

All 300 close-up images included in the training set.

pTis; melanoma *in situ*.


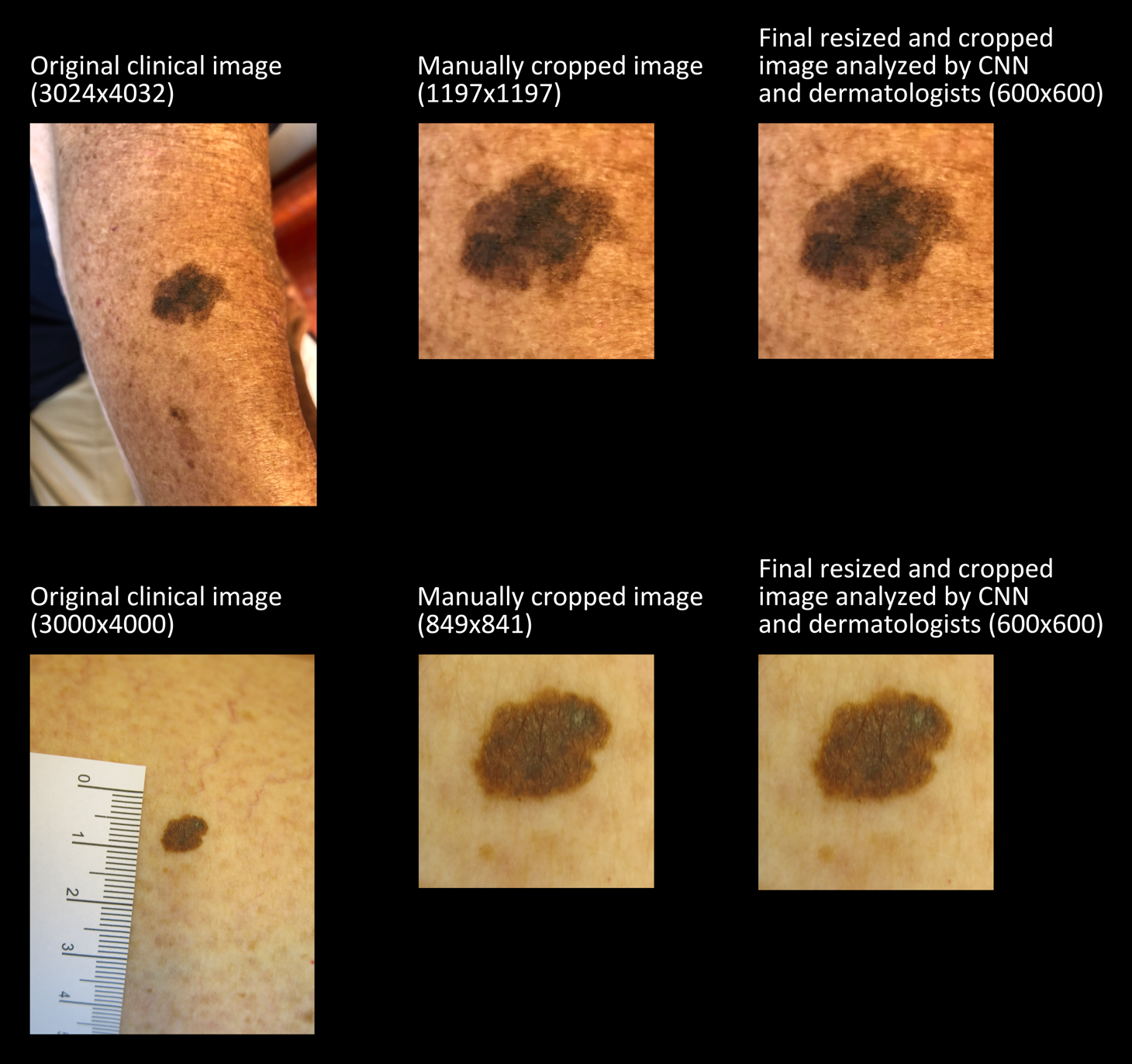


**Supplementary Figure 1.** Example images of the cropping process.


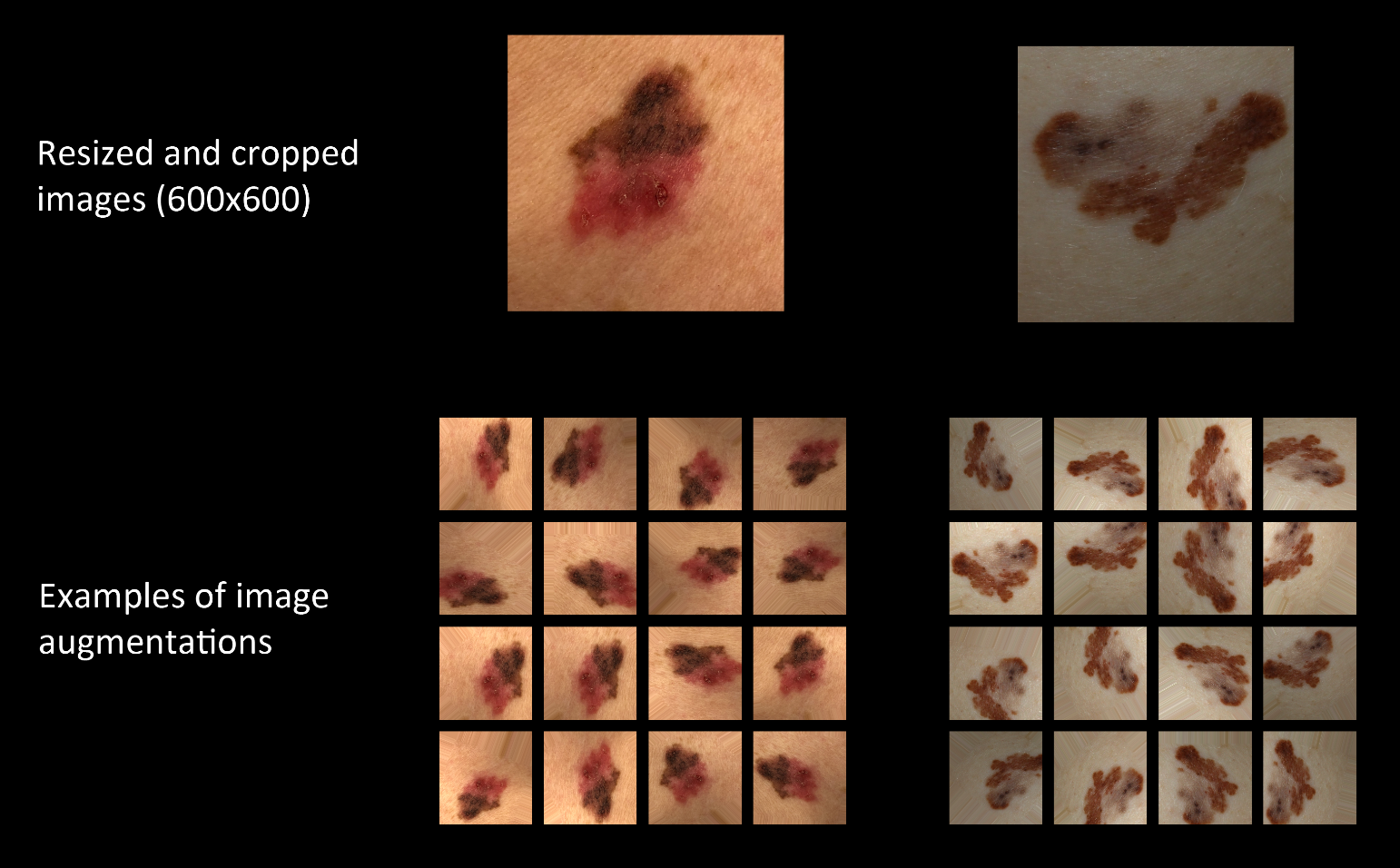


**Supplementary Figure 2.** Example images of the augmentation process.


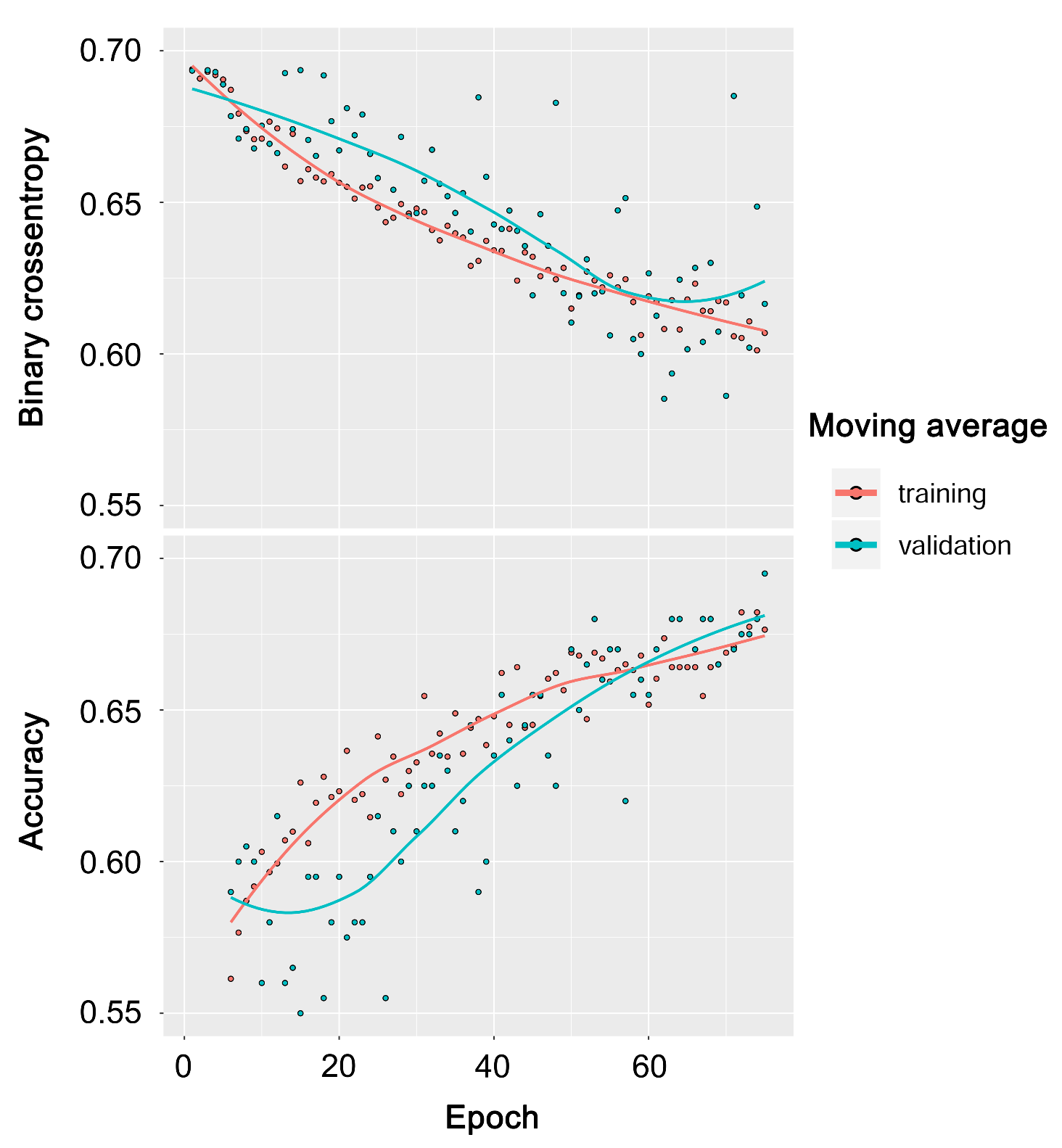


**Supplementary Figure 3.** Accuracy rates and binary crossentropy in nats*.*

The mean of -y log_e_ s – (1-y) log_e_ (1-s) for all cases where “s” is the CNN’s output score and “y” is the true label for each case (lower is better) during validation of the selected model.


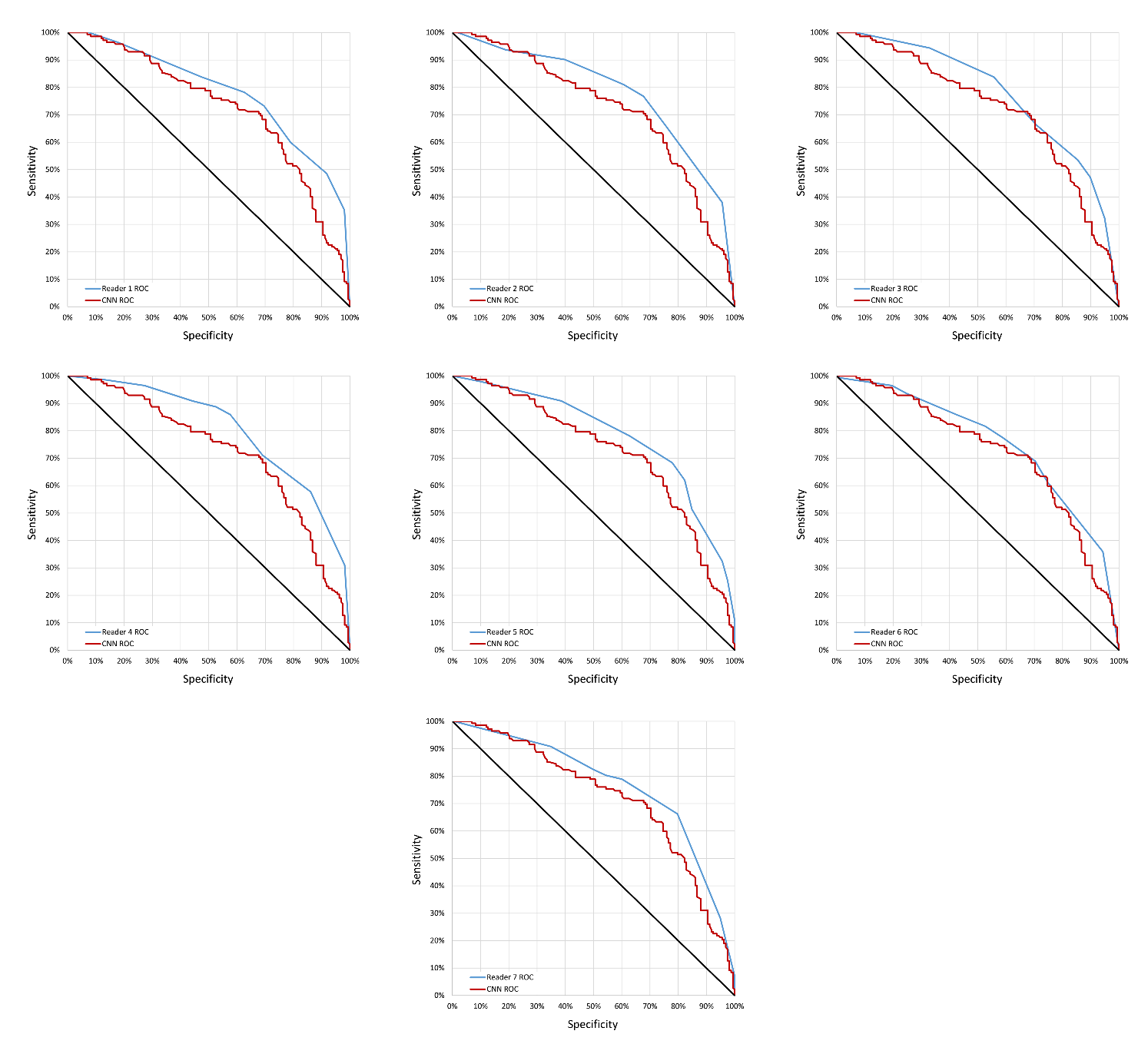


**Supplementary Figure 4.** ROC curves for each individual dermatologist compared to the CNN.

CNN, convolutional neural network.


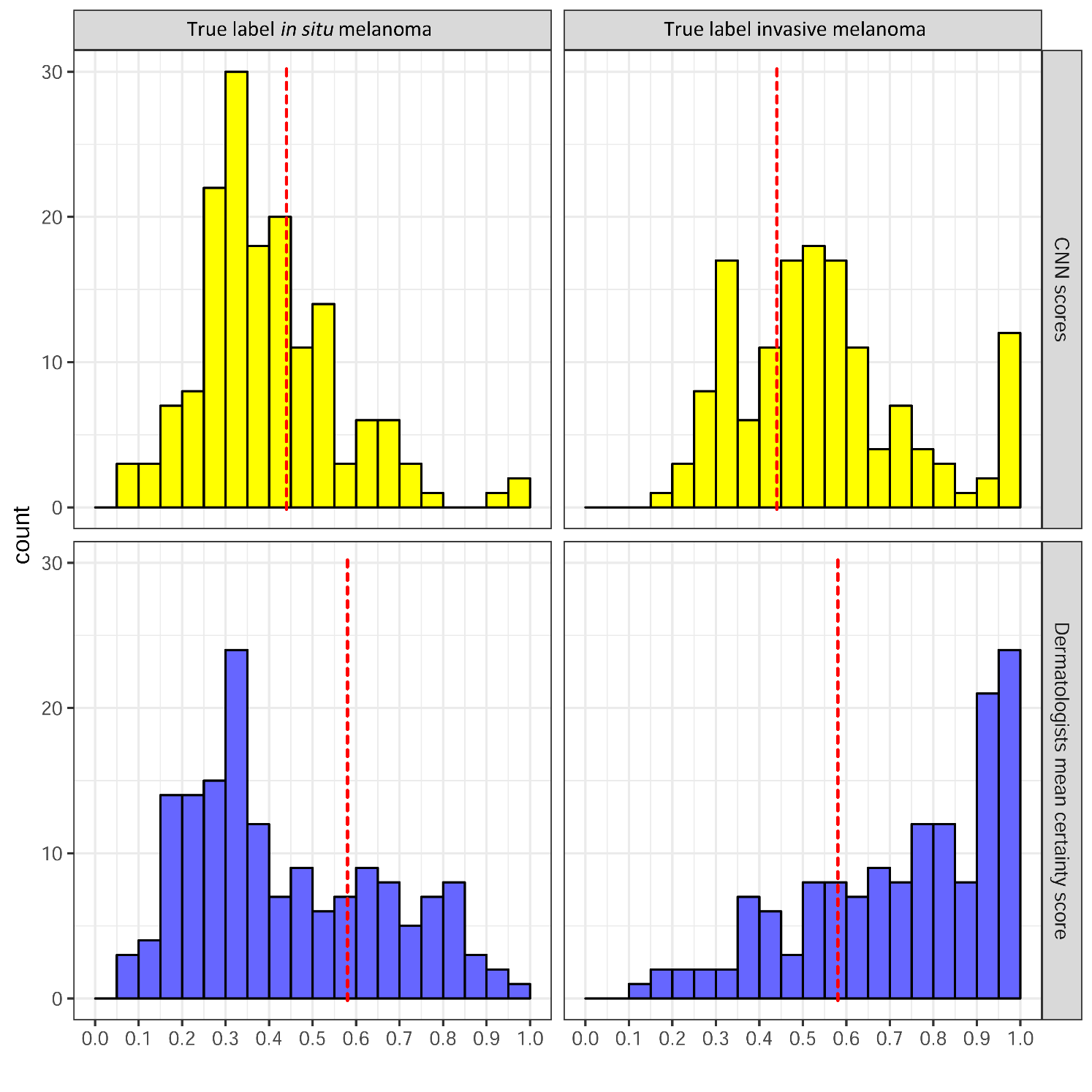


**Supplementary Figure 5.** Histogram of CNN and dermatologists’ output.

The dotted red lines correspond to the threshold where sensitivity and specificity was closest. For the CNN, this threshold value was 0.44 and corresponded to a sensitivity and specificity of 69.7% and 69.0%, respectively. For the dermatologists, this threshold value was 0.58 and corresponded to a sensitivity and specificity of 72.5% and 71.5%, respectively.

CNN, convolutional neural network.


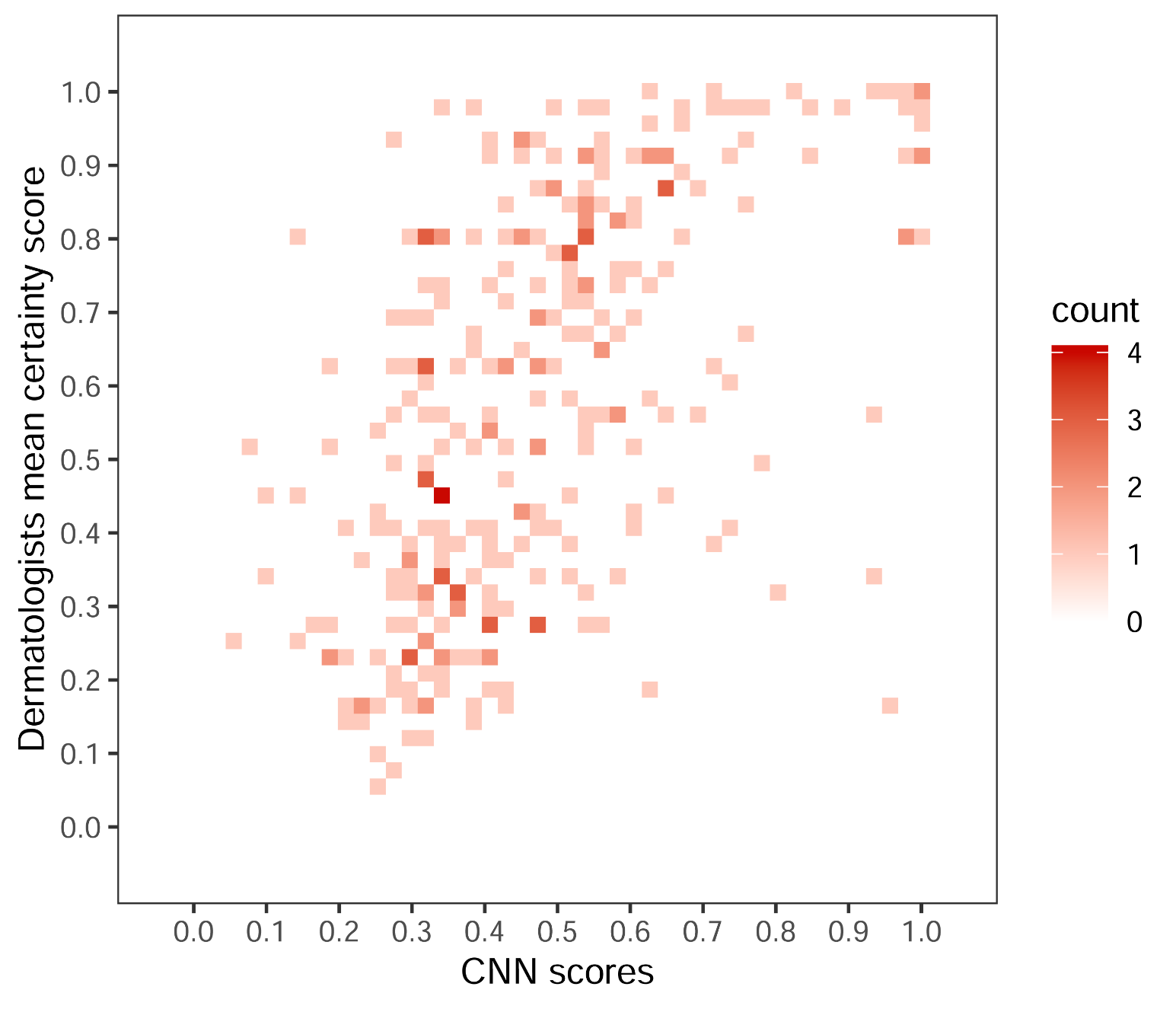


**Supplementary Figure 6.** Correlation map of CNN and dermatologists’ output.

Each box represents a number of cases according to the color scale.

CNN, convolutional neural network.


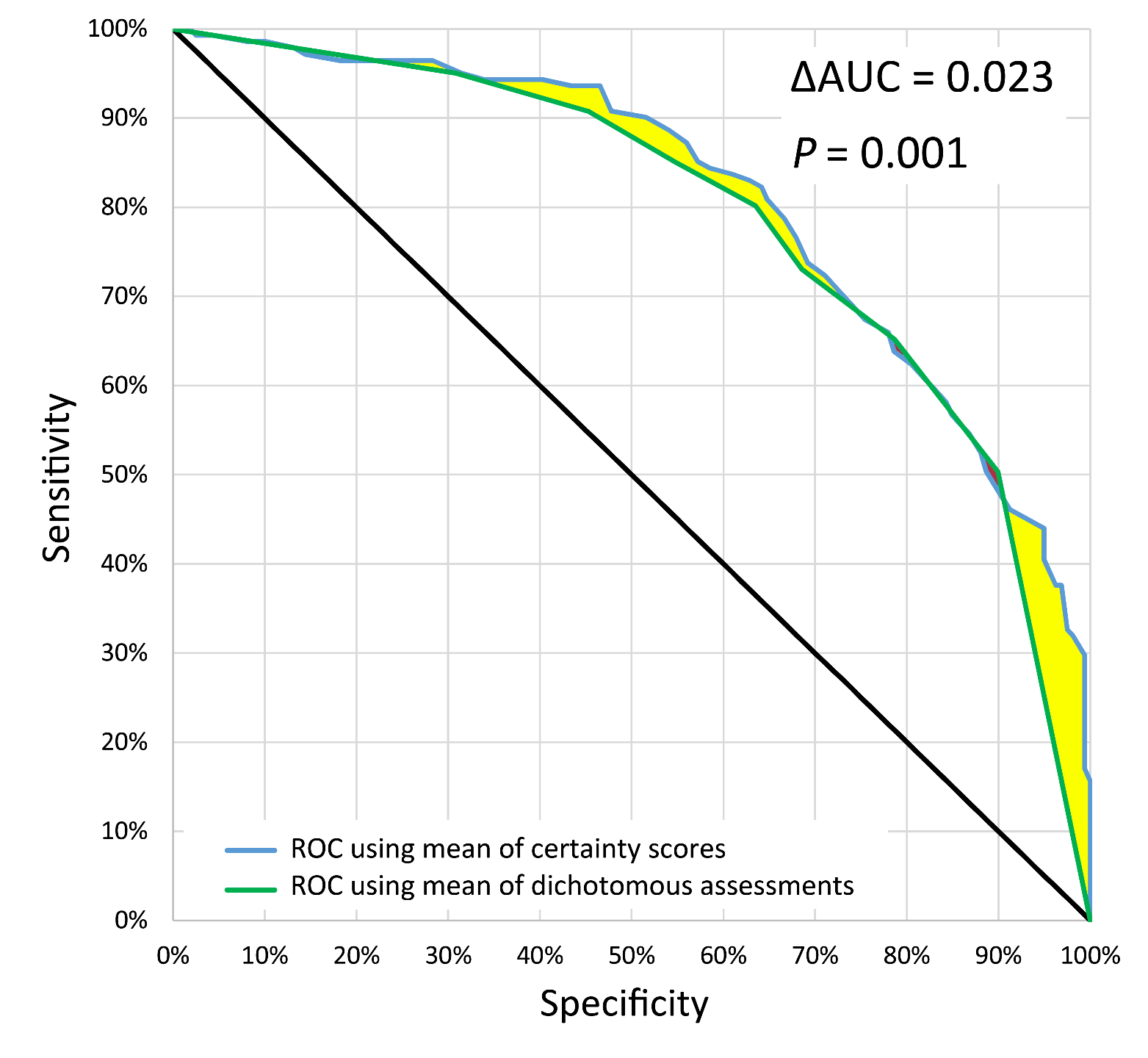


**Supplementary Figure 7.** Difference in area under the dermatologists’ ROC curves*.*

The blue line represents the combined ROC based on taking the mean of the individual seven dermatologists’ certainty scores ranging from 0-1 (nine intervals). The green line represents the ROC based on taking the mean of the individual seven dermatologists’ dichotomous answers (0=MIS and 1=Invasive melanoma).

AUC, area under the ROC curve; MIS, melanoma *in situ*; ROC, receiver operating characteristics.


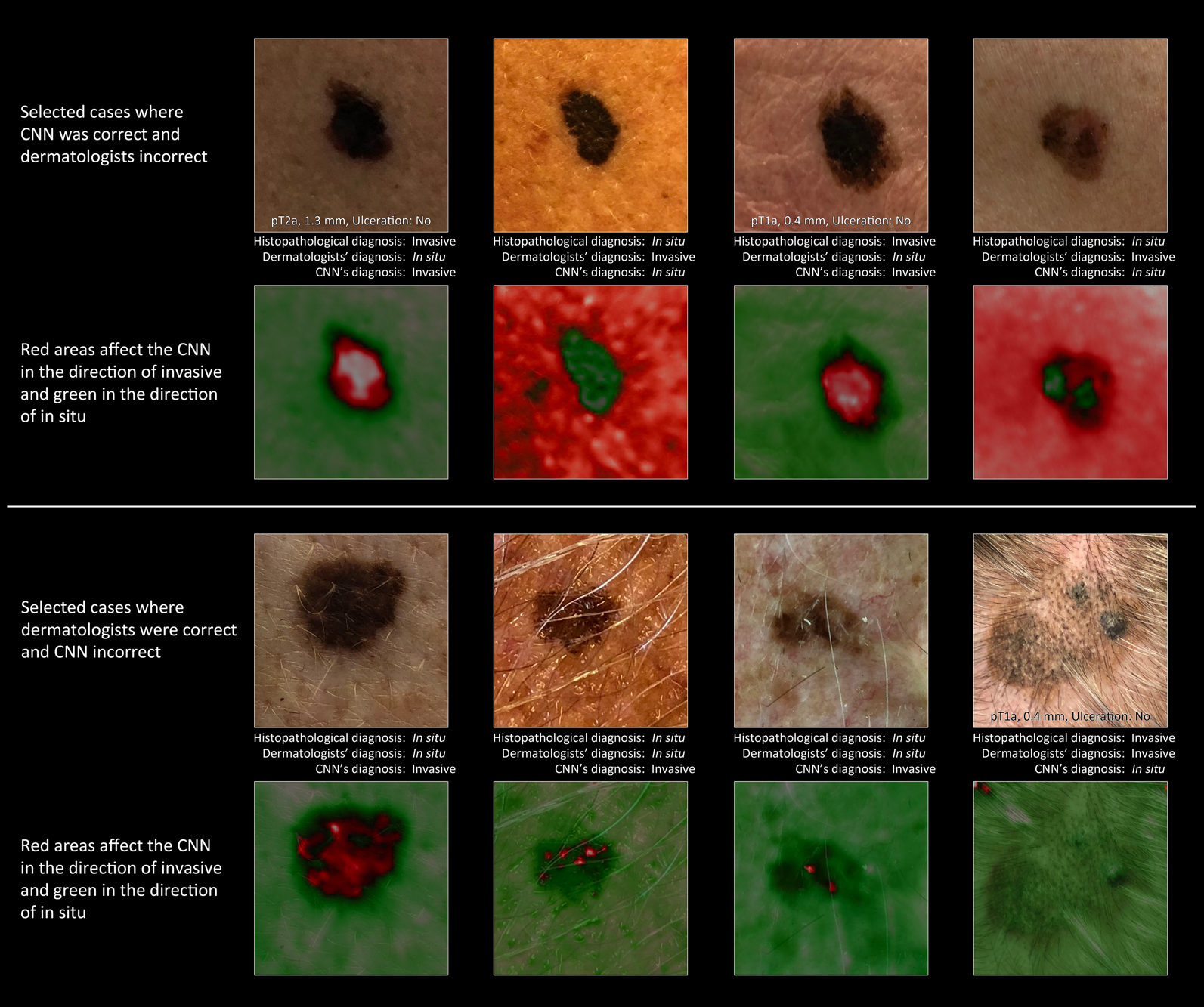


**Supplementary Figure 8.** Class activation maps of selected images*.*

Selected cases where there was disagreement in output between the dermatologists and CNN. The answers are with respect to the point on the ROC curves where sensitivity and specificity was the closest. The red-green images represent CAMs using the fourth convolutional layer in the model. These illustrations can be considered as ‘heat maps’ highlighting pixels that were important for each category (invasive/*in situ*) for each selected case. Each channel (red/green) was normalised with respect to itself.

CAMs, class activation maps; CNN, convolutional neural network; ROC, receiver operating characteristic.
