# Supplementary material for: Discrimination Between Invasive and In Situ Melanomas Using Clinical Close-Up Images and a De Novo Convolutional Neural Network
Source: Front Med (Lausanne). 2021 Sep 14;8:723914. doi: 10.3389/fmed.2021.723914 (PMC8476836; doi:10.3389/fmed.2021.723914)
Supplement: Supplementary file 2 [file Data_Sheet_1.PDF]

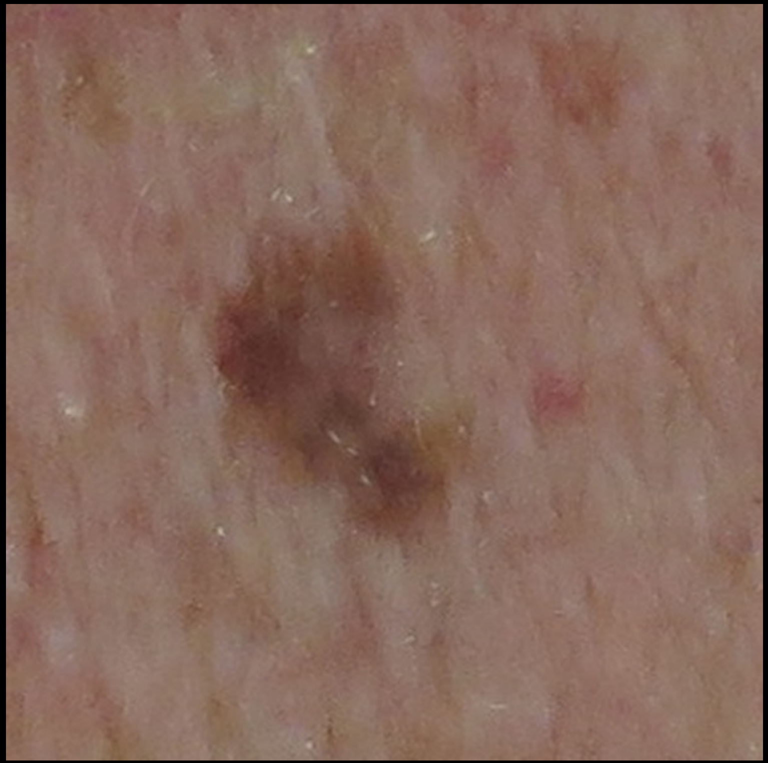

pTis

Case number 1

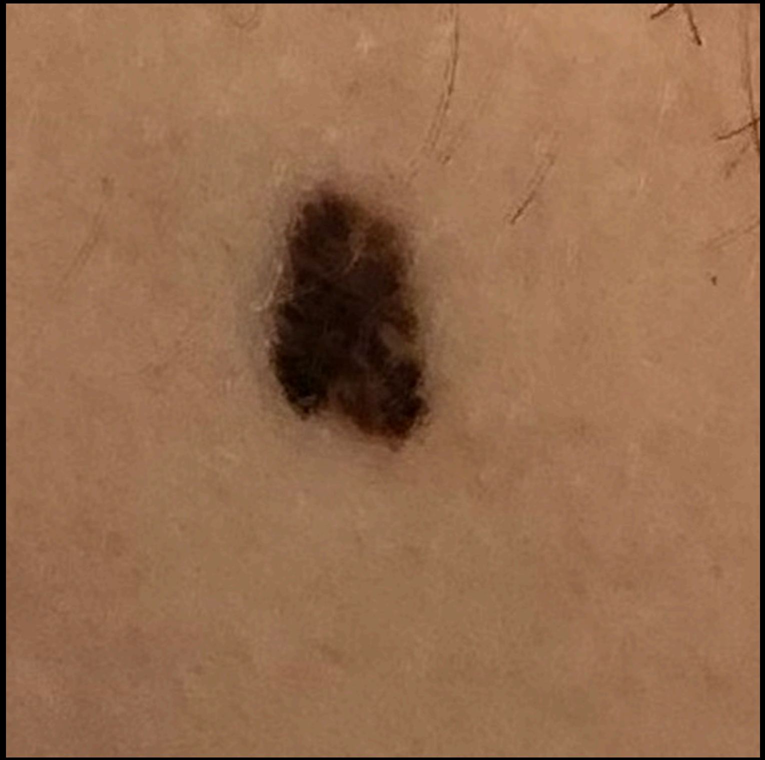

pTis

Case number 2

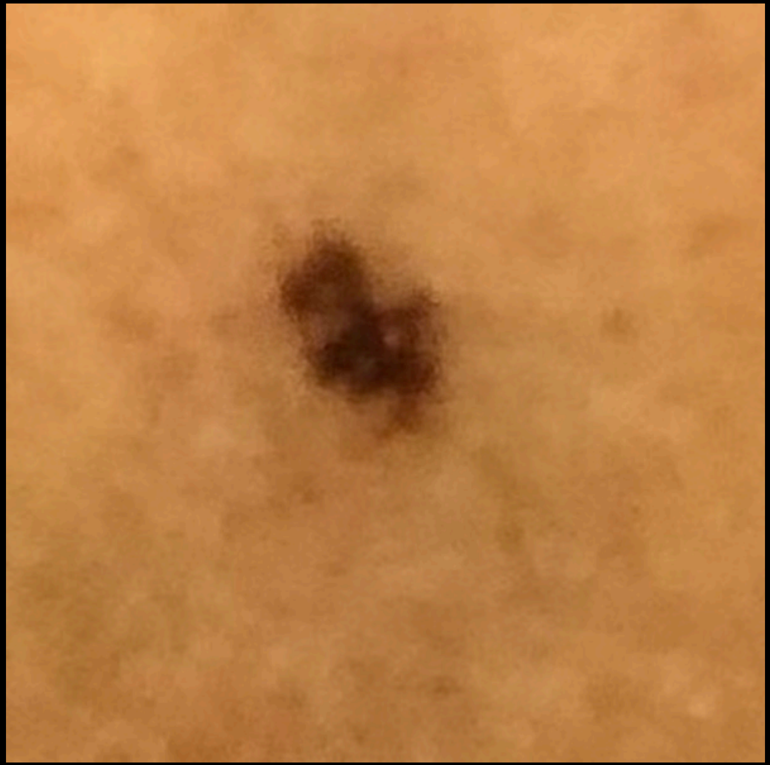

pTis

Case number 3

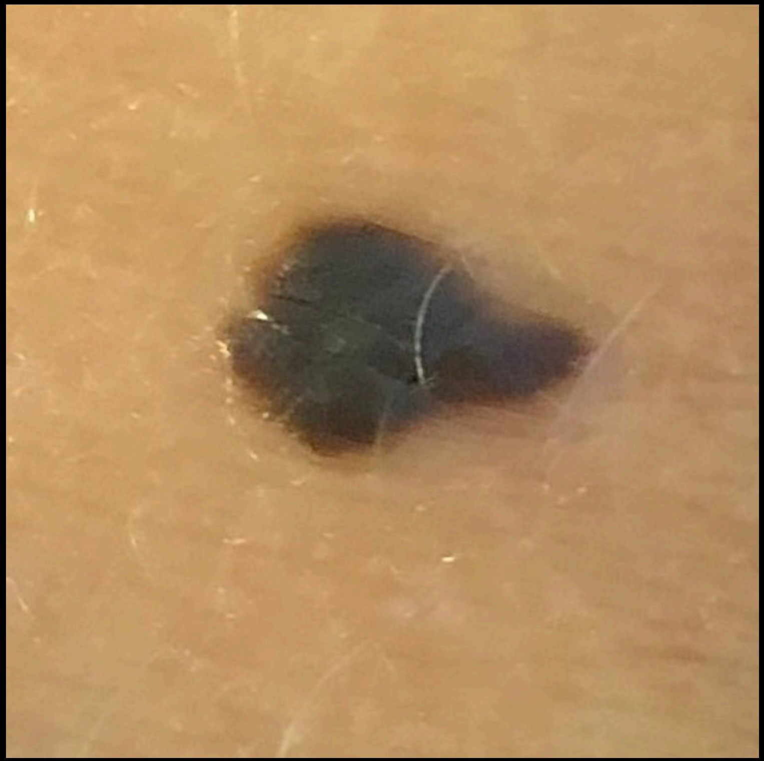

pTis

Case number 4

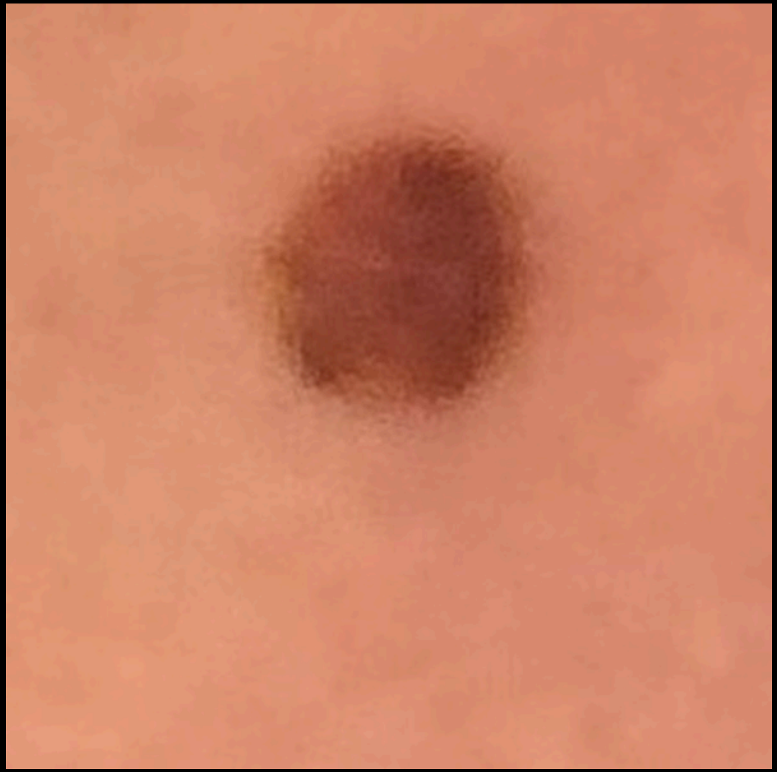

pTis

Case number 5

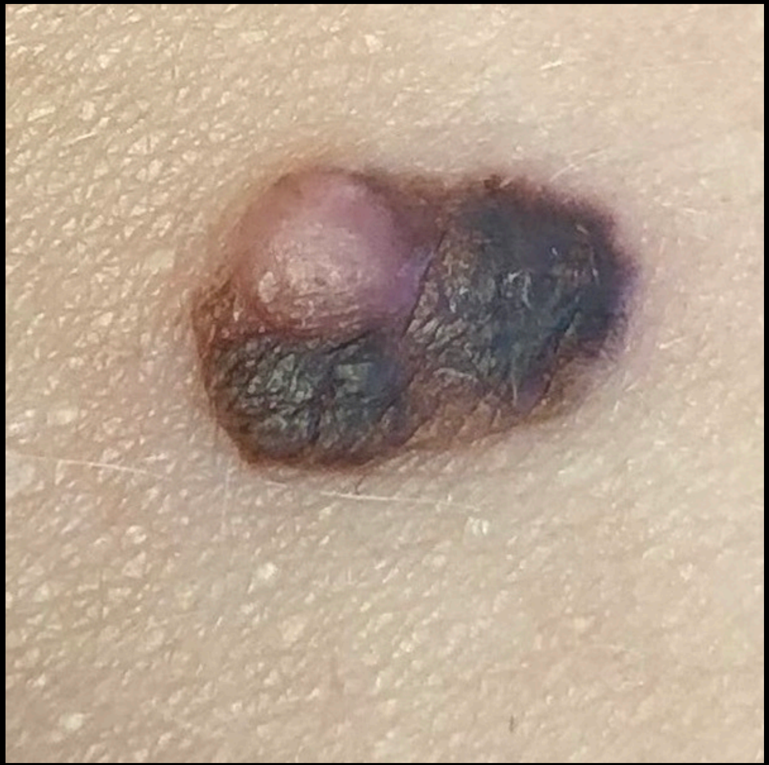

pT2a, 1.4 mm

Case number 6

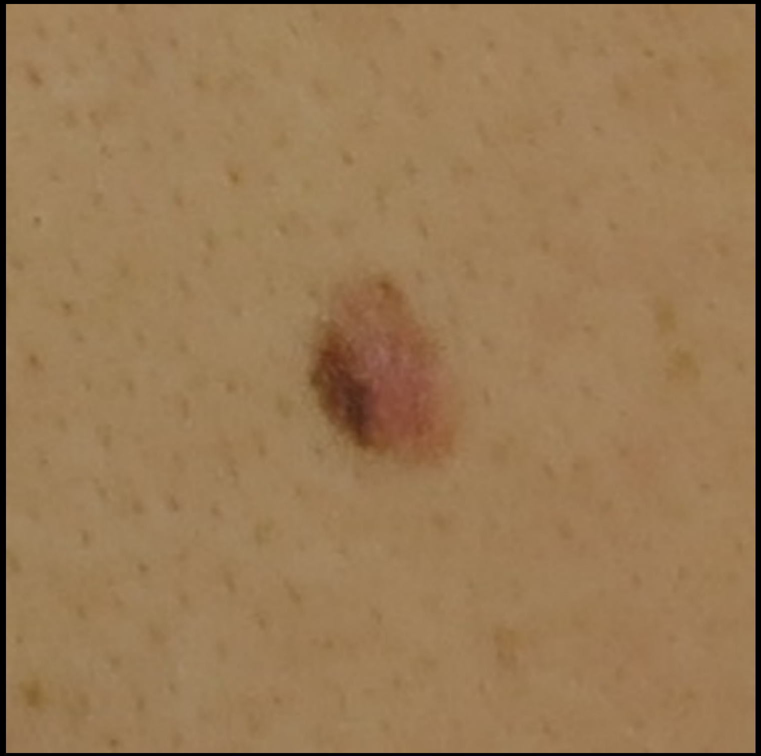

pT1b, 0.9 mm

Case number 7

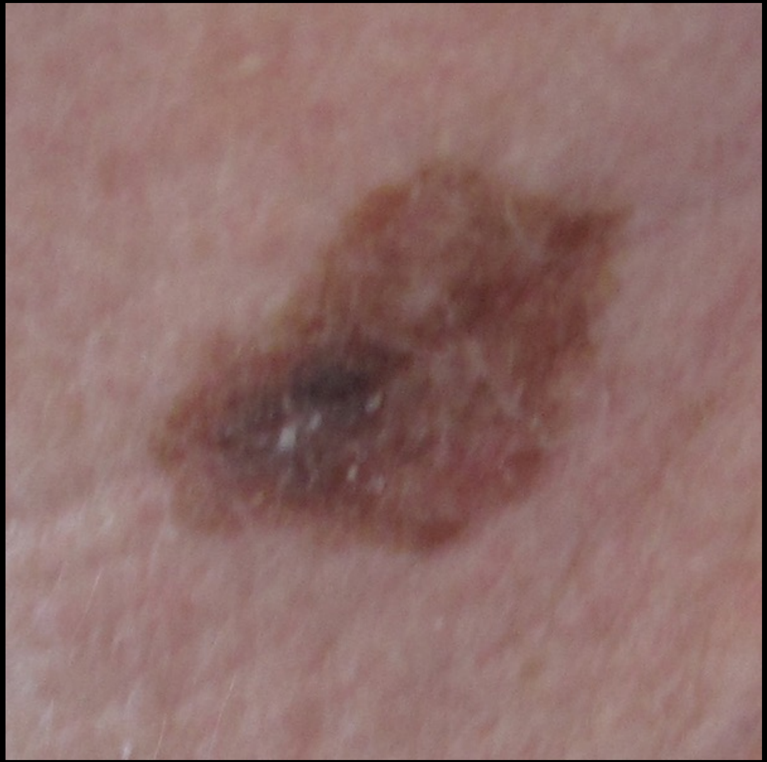

pTis

Case number 8

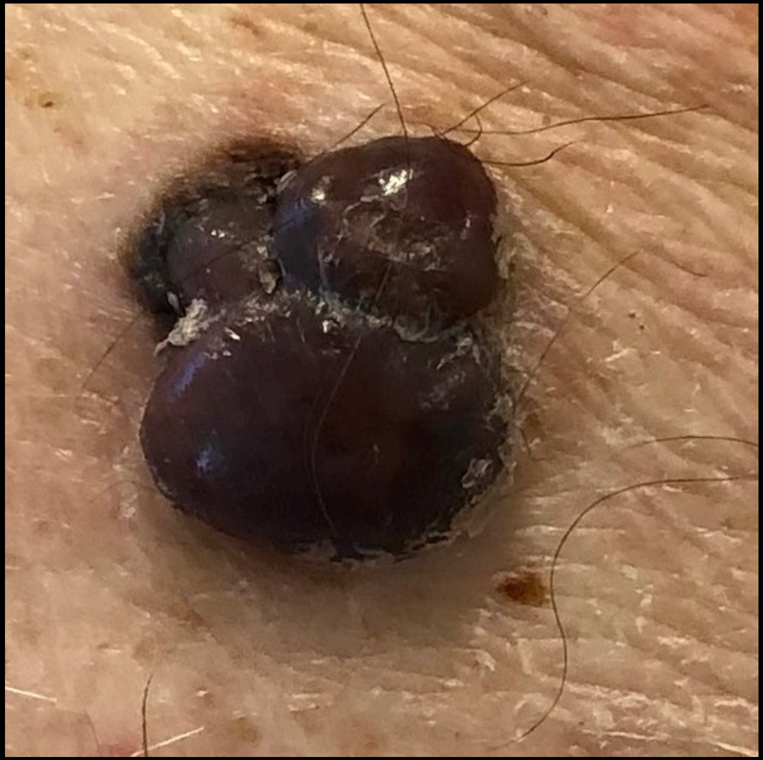

pT4a, 9.0 mm

Case number 9

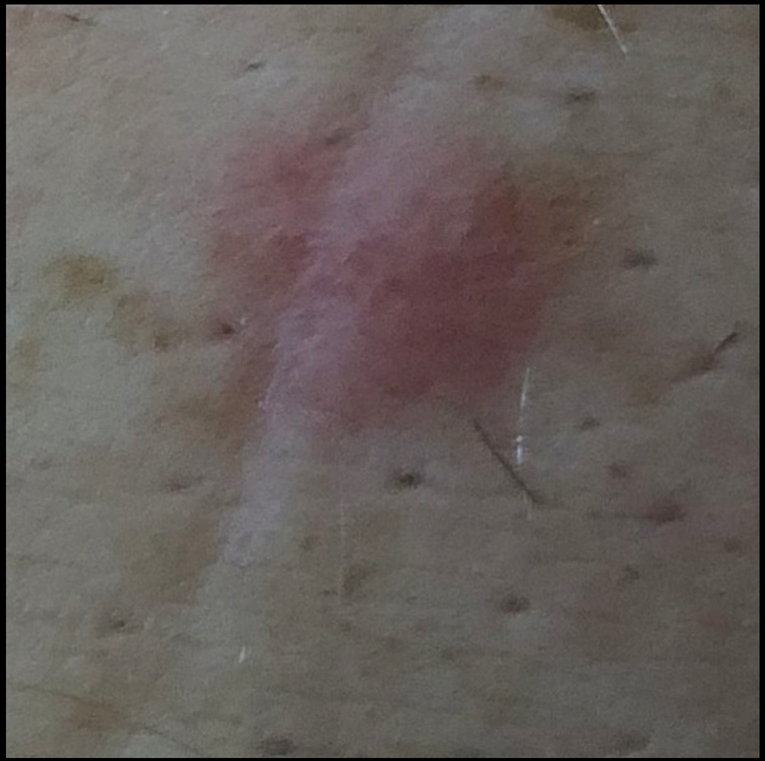

pTis

Case number 10

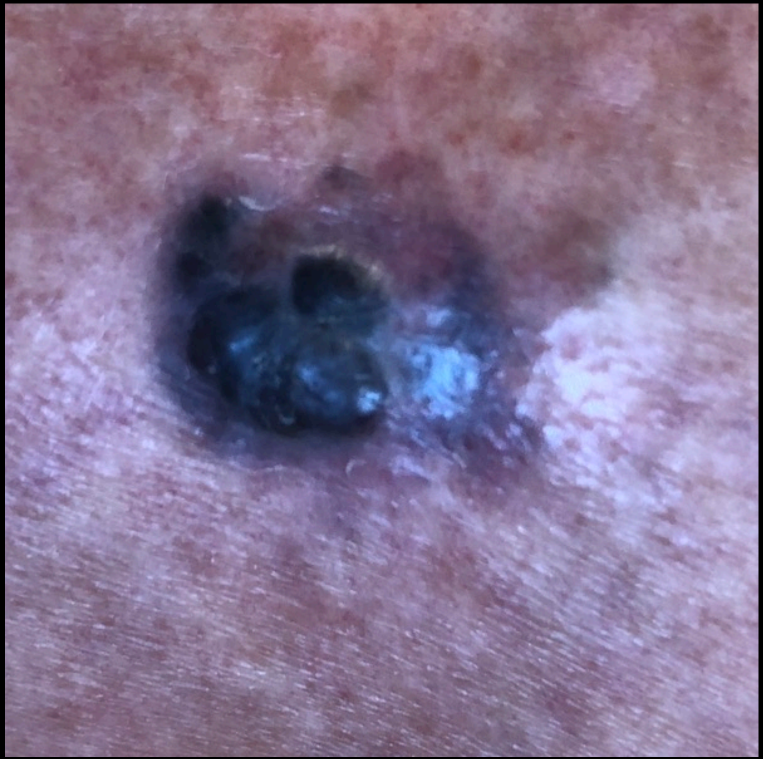

pT3a, 2.5 mm

Case number 11

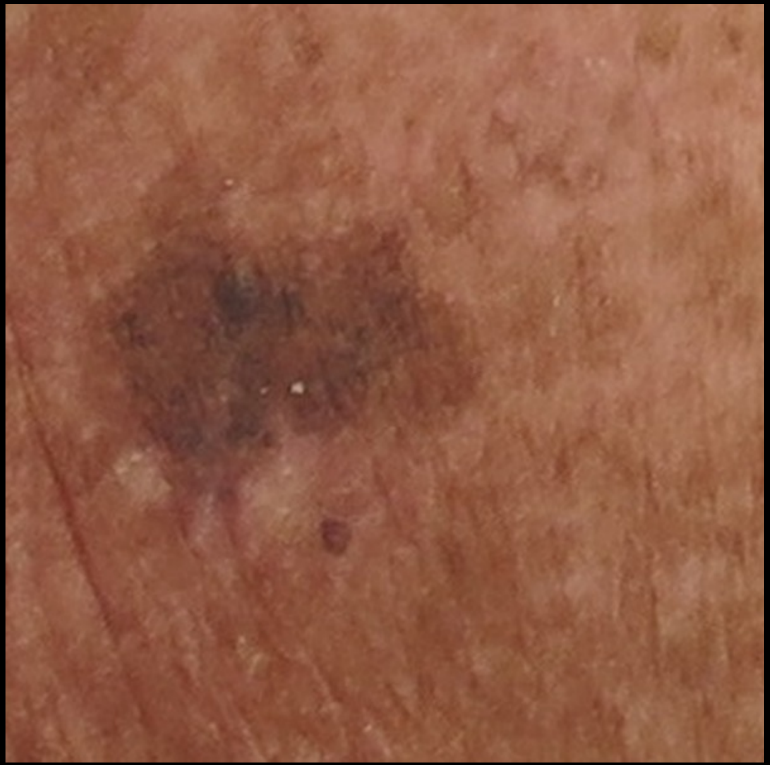

pTis

Case number 12

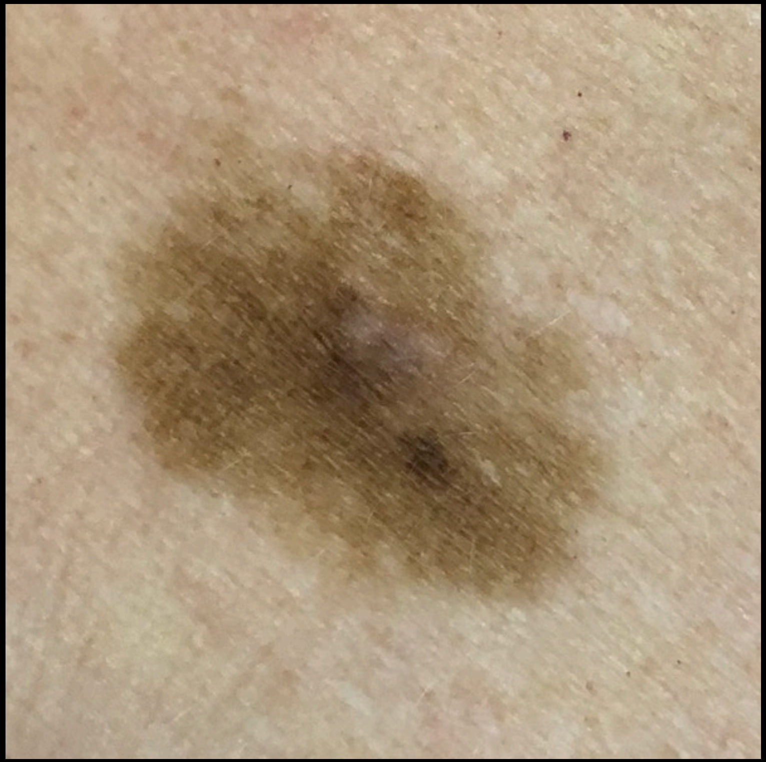

pTis

Case number 13

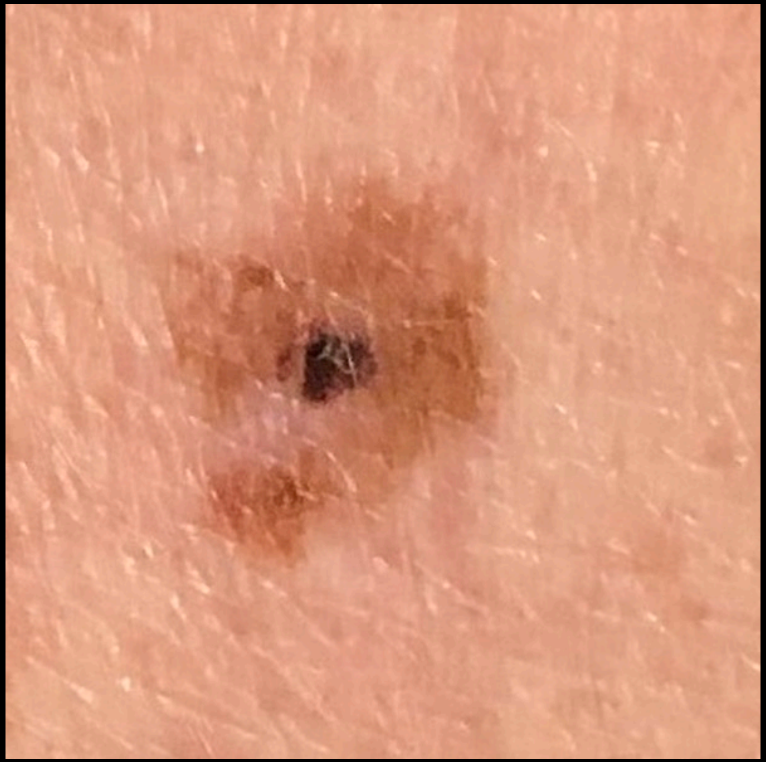

pTis

Case number 14

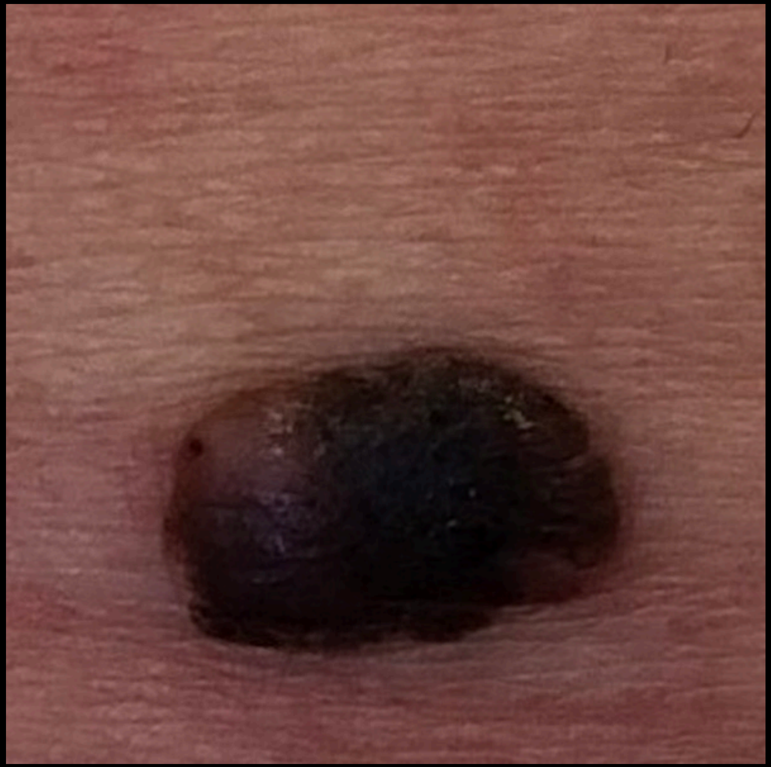

pT2a, 1.5 mm

Case number 15

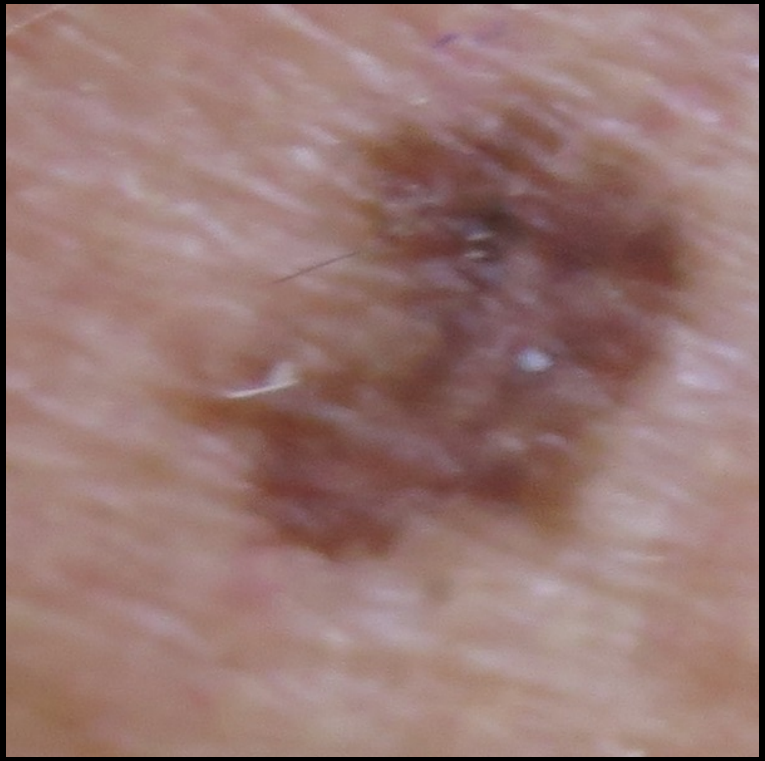

pTis

Case number 16

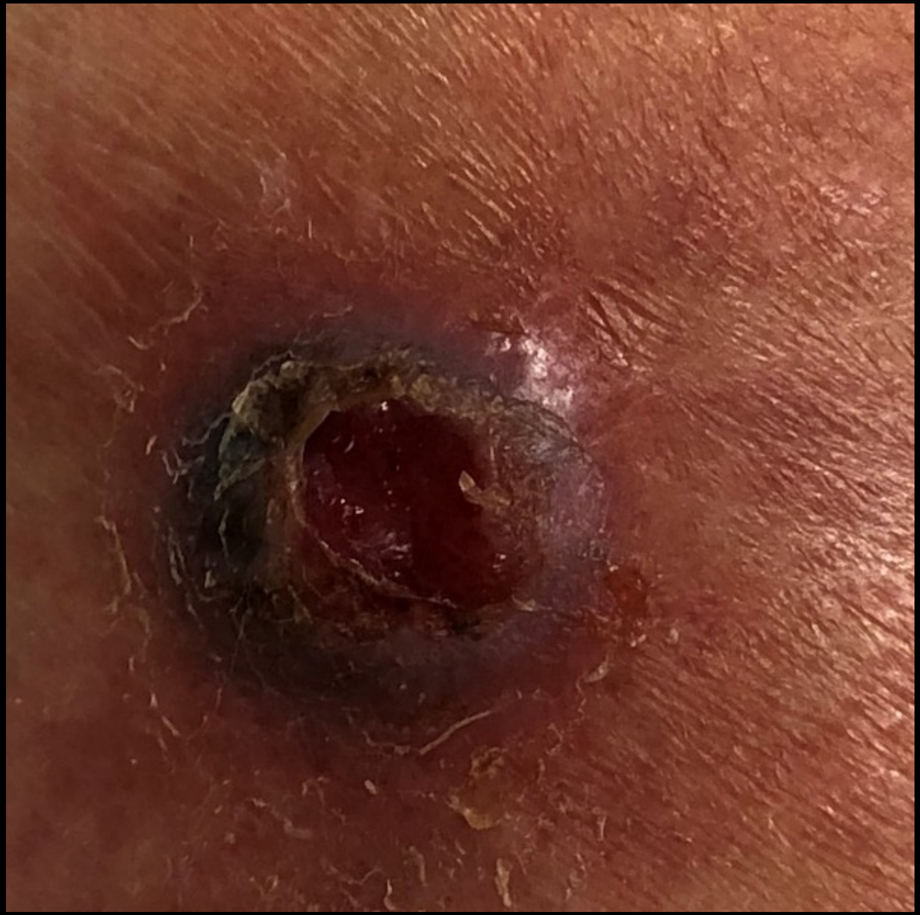

pT3a, 3.2 mm

Case number 17

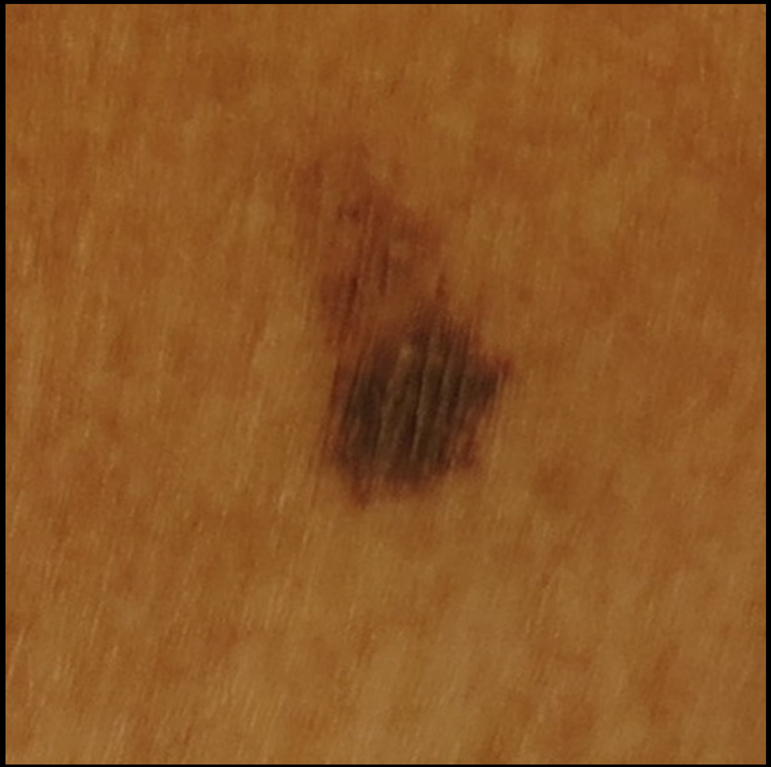

pTis

Case number 18

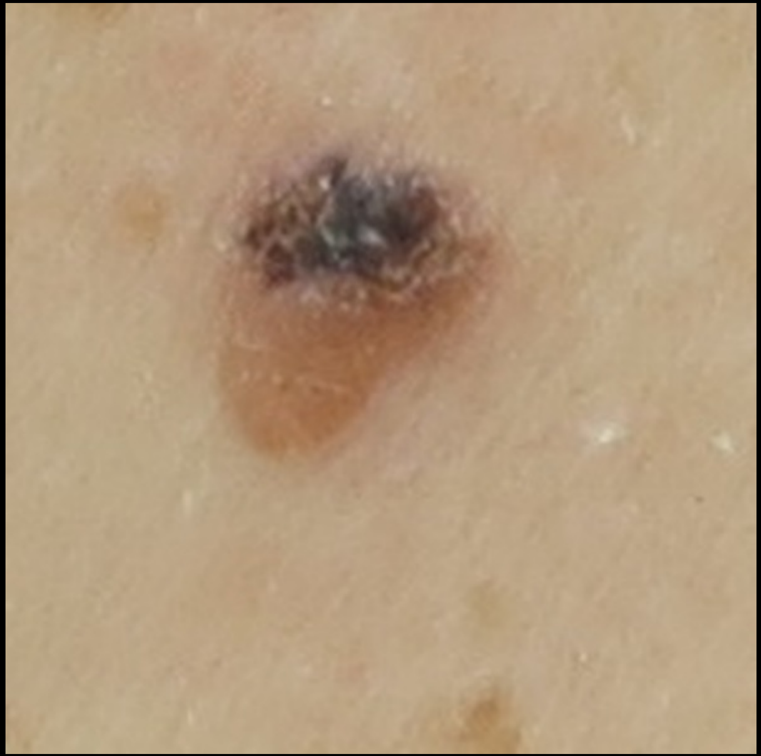

pTis

Case number 19

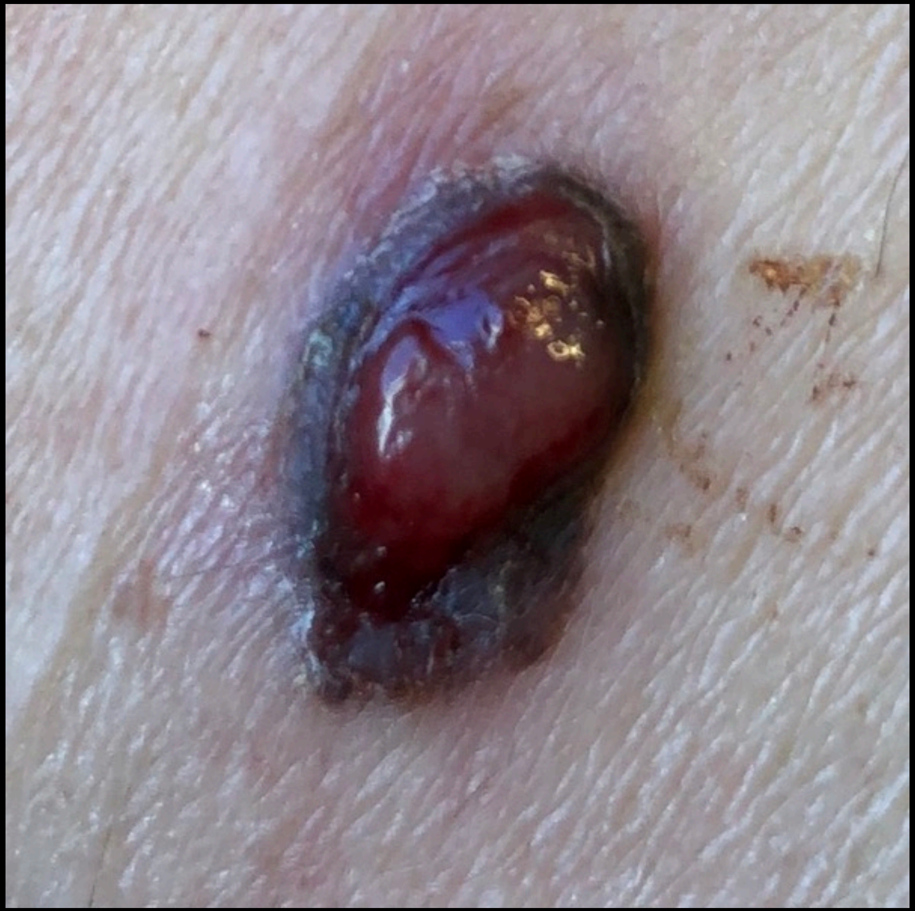

pT4b, 4.5 mm

Case number 20

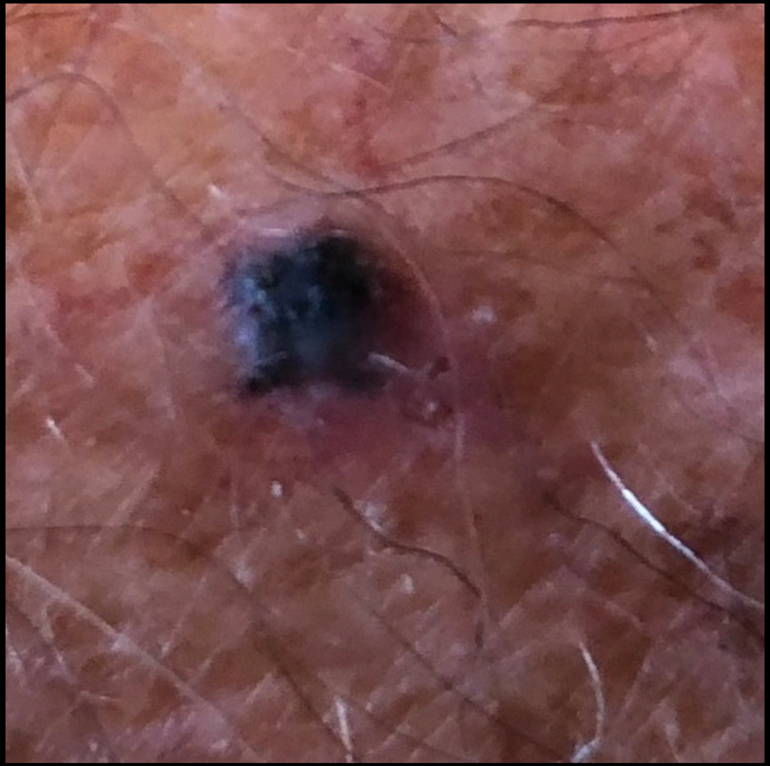

pT1a, 0.7 mm

Case number 21

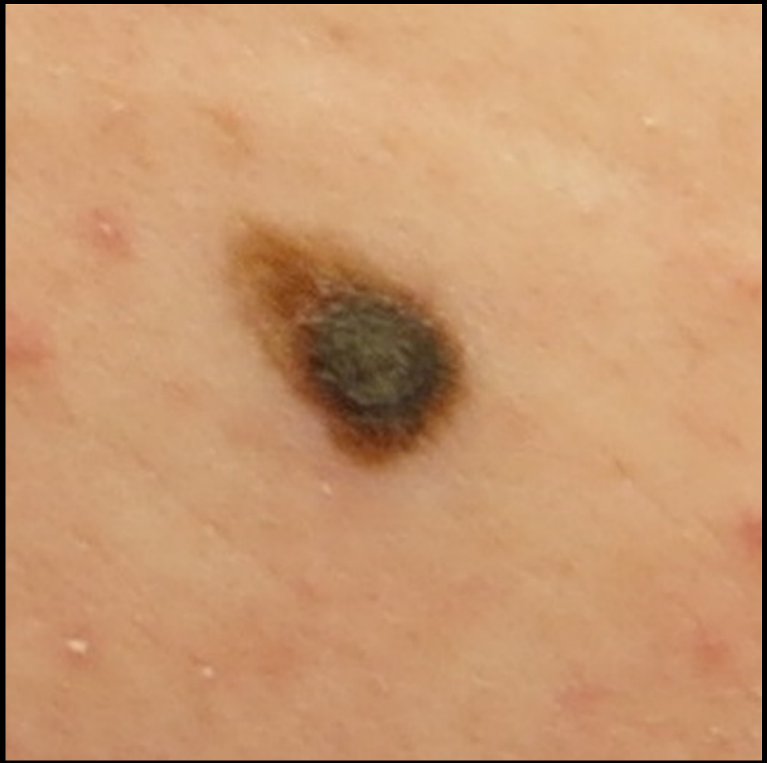

pTis

Case number 22

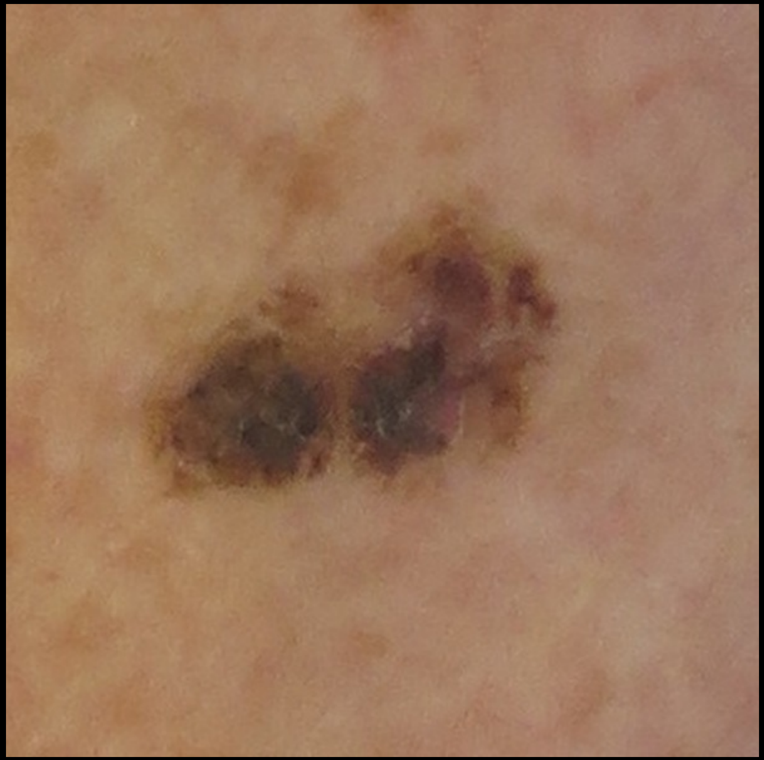

pTis

Case number 23

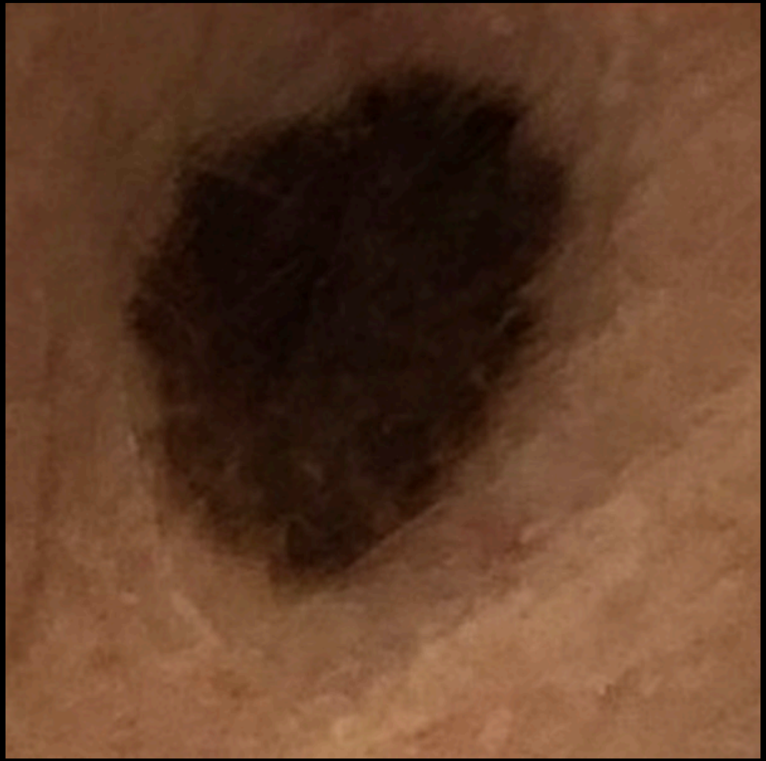

pTis

Case number 24

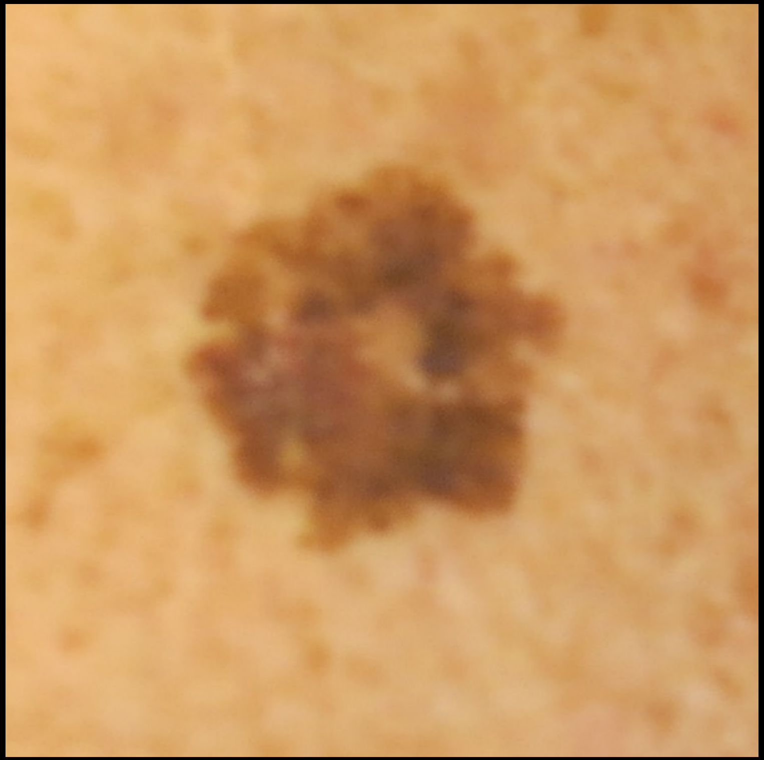

pT1a, 0.4 mm

Case number 25

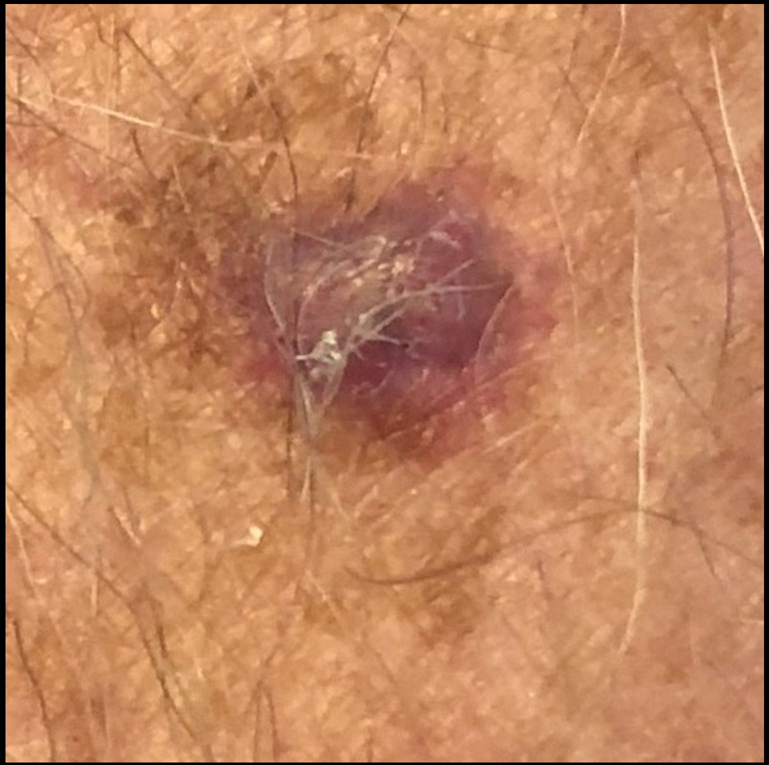

pT2a, 1.6 mm

Case number 26

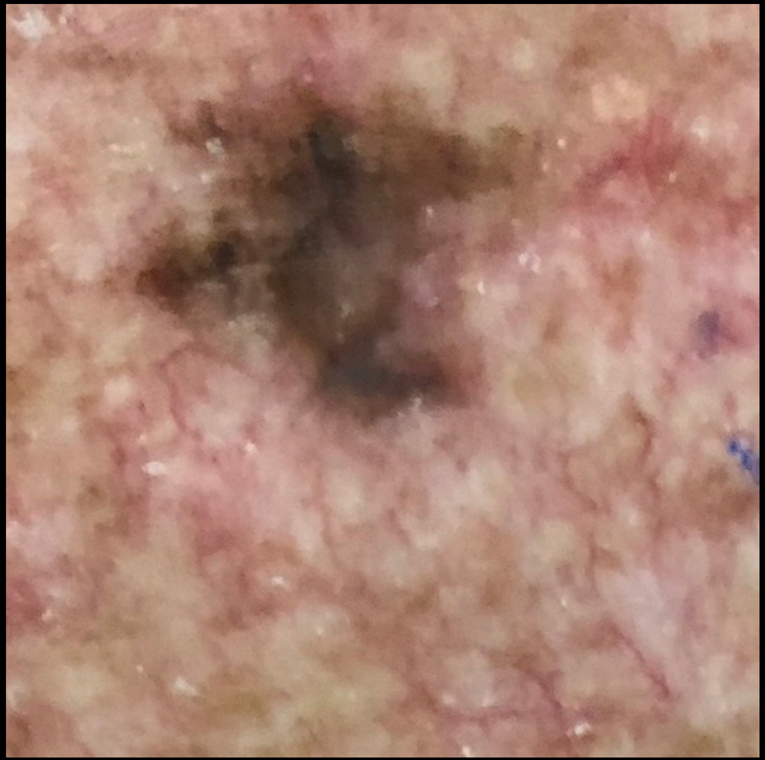

pTis

Case number 27

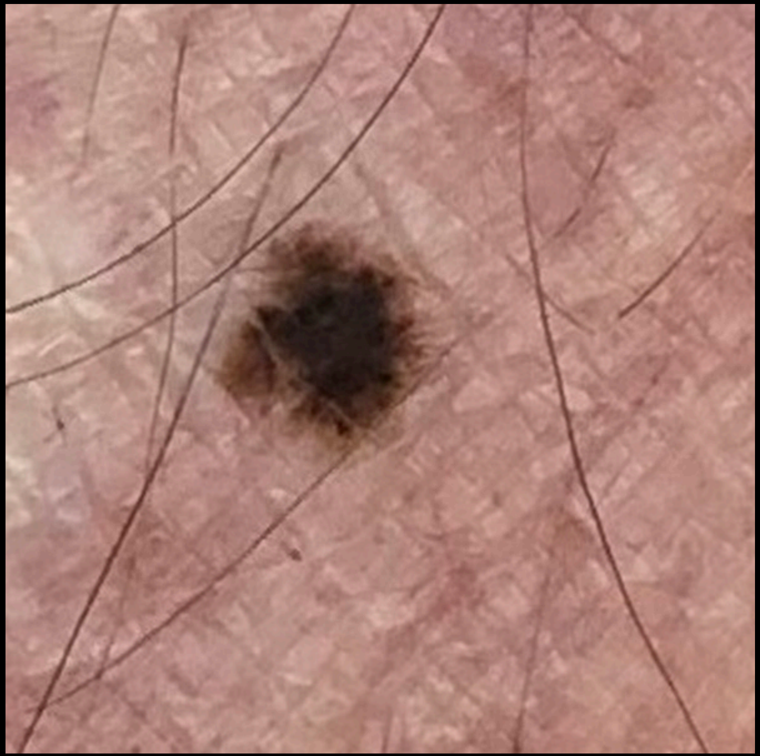

pTis

Case number 28

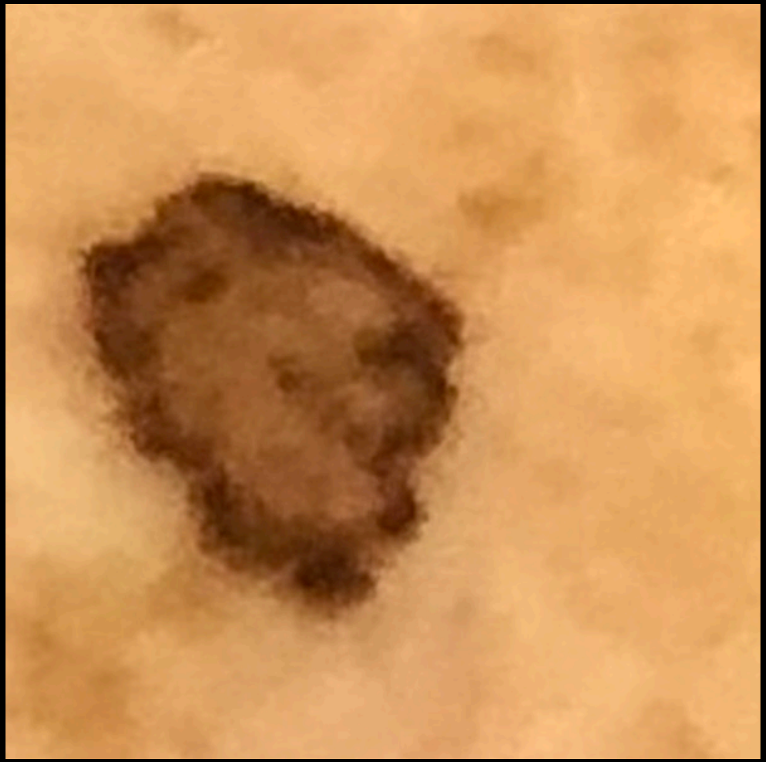

pTis

Case number 29

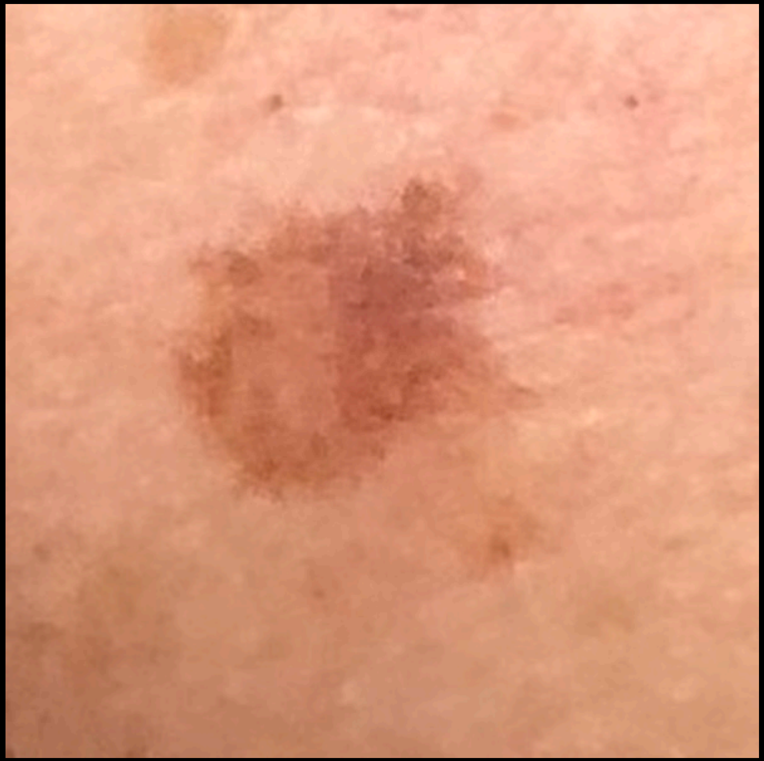

pT1a, 0.4 mm

Case number 30

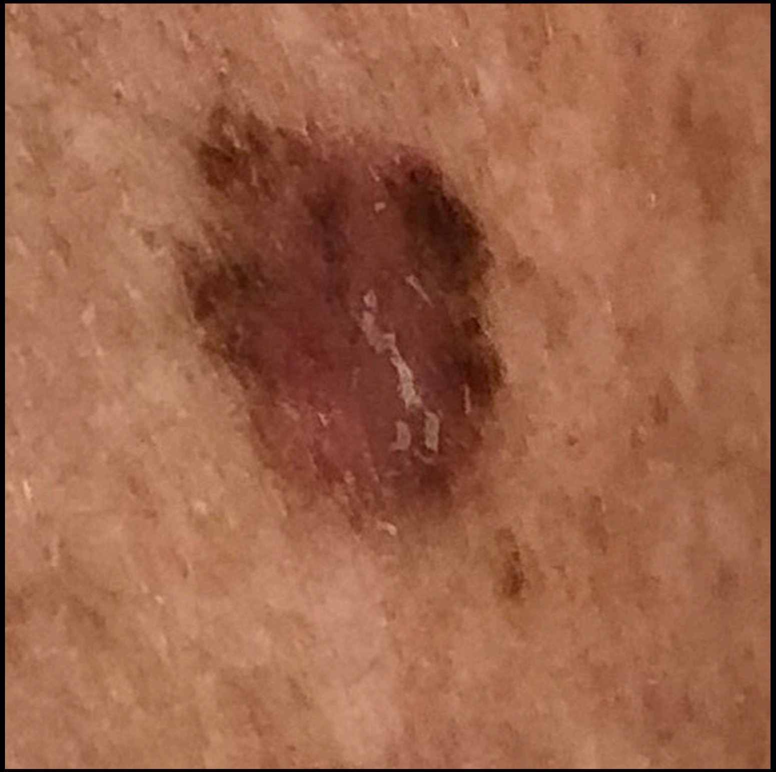

pT1a, 0.7 mm

Case number 31

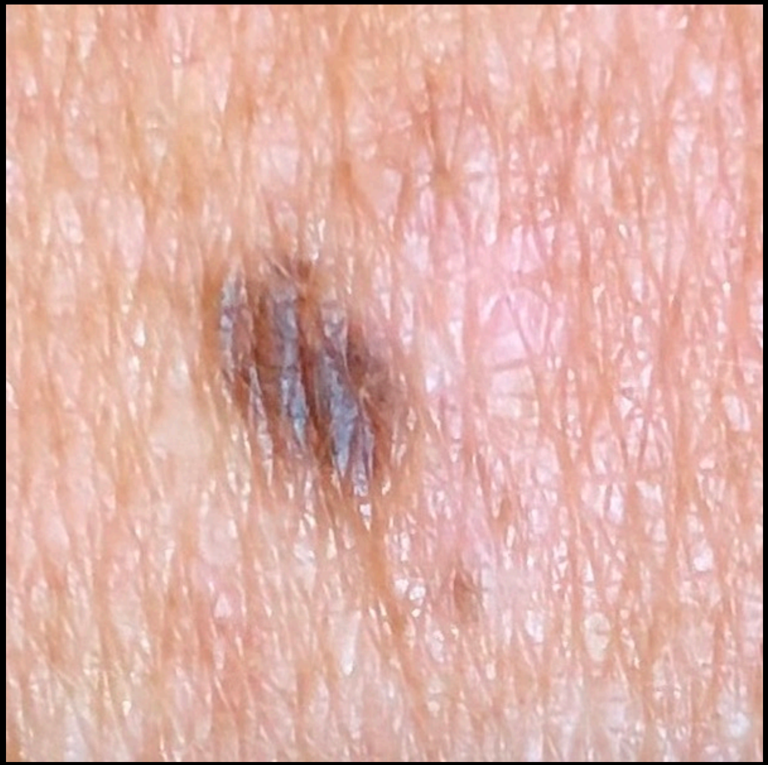

pT2a, 1.6 mm

Case number 32

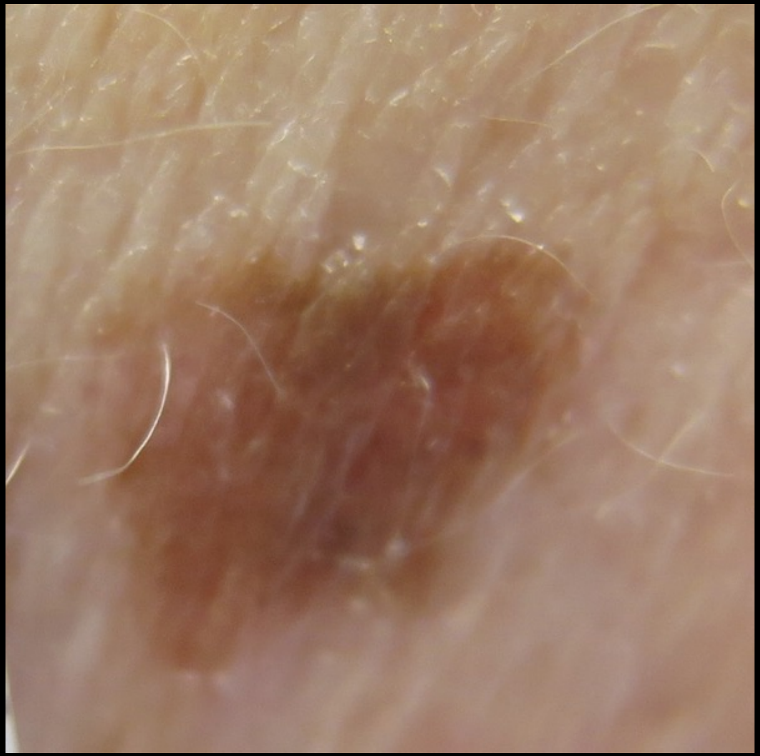

pTis

Case number 33

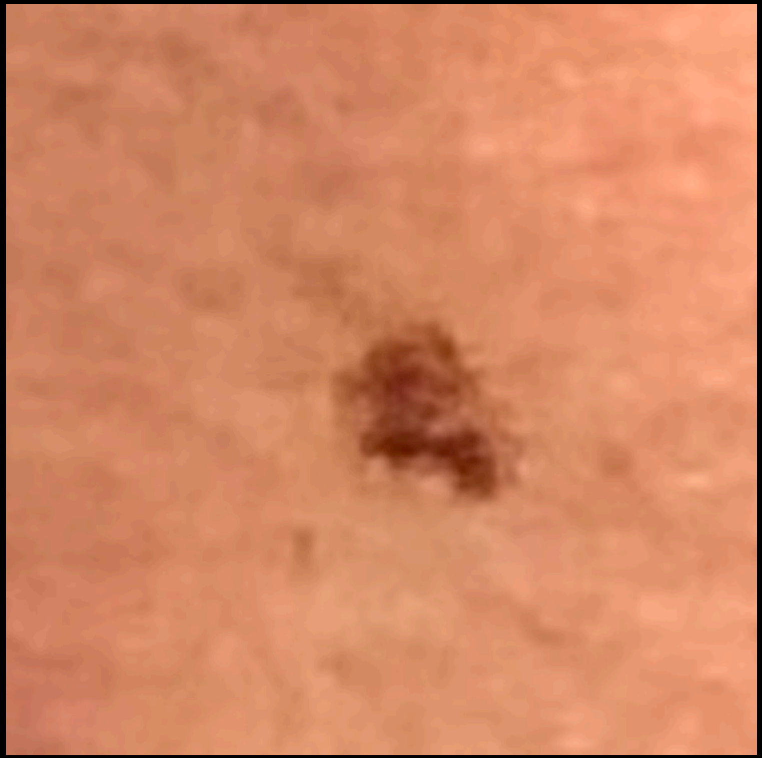

pTis

Case number 34

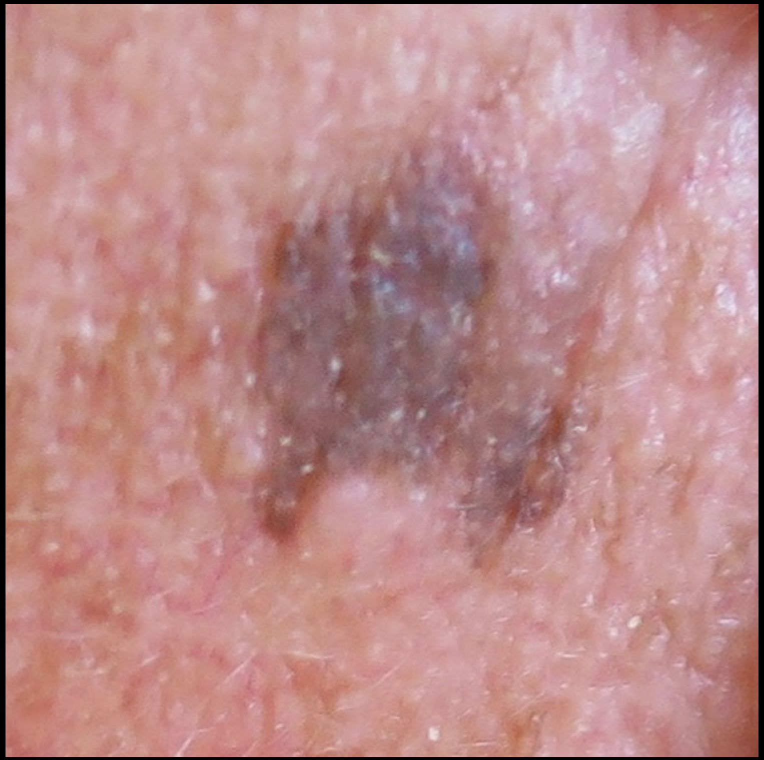

pTis

Case number 35

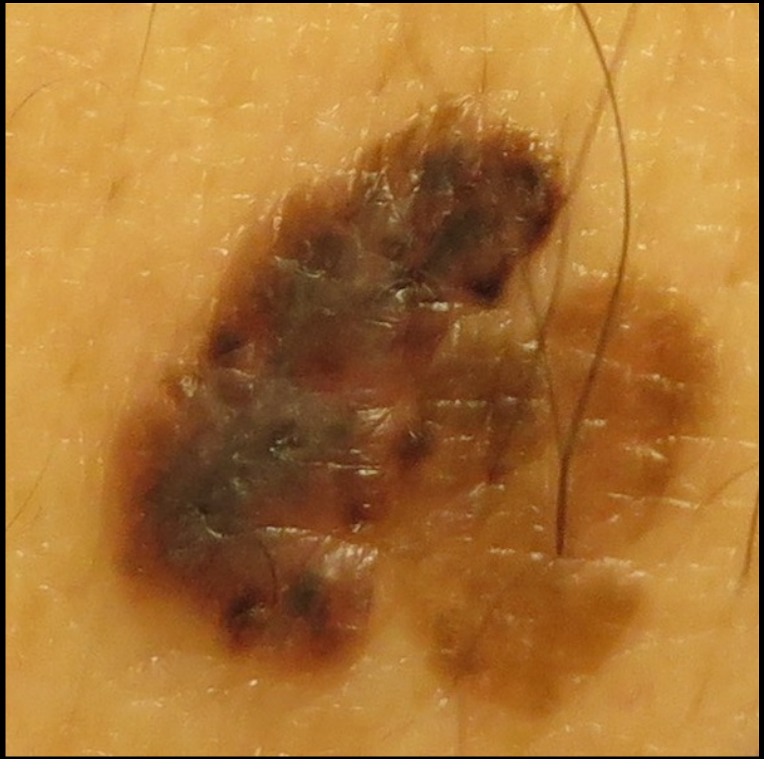

pT1a, 0.5 mm

Case number 36

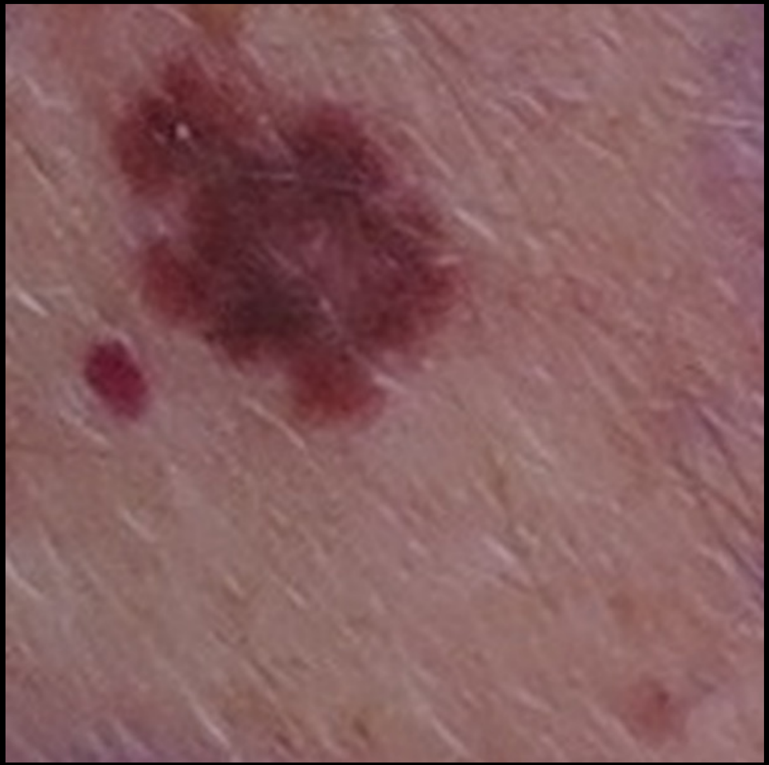

pT1a, 0.3 mm

Case number 37

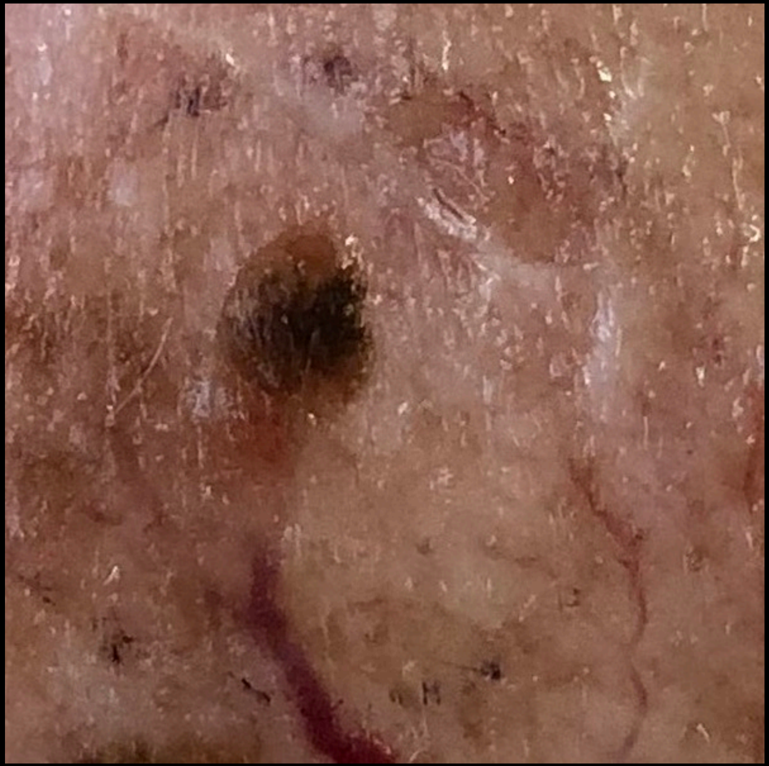

pTis

Case number 38

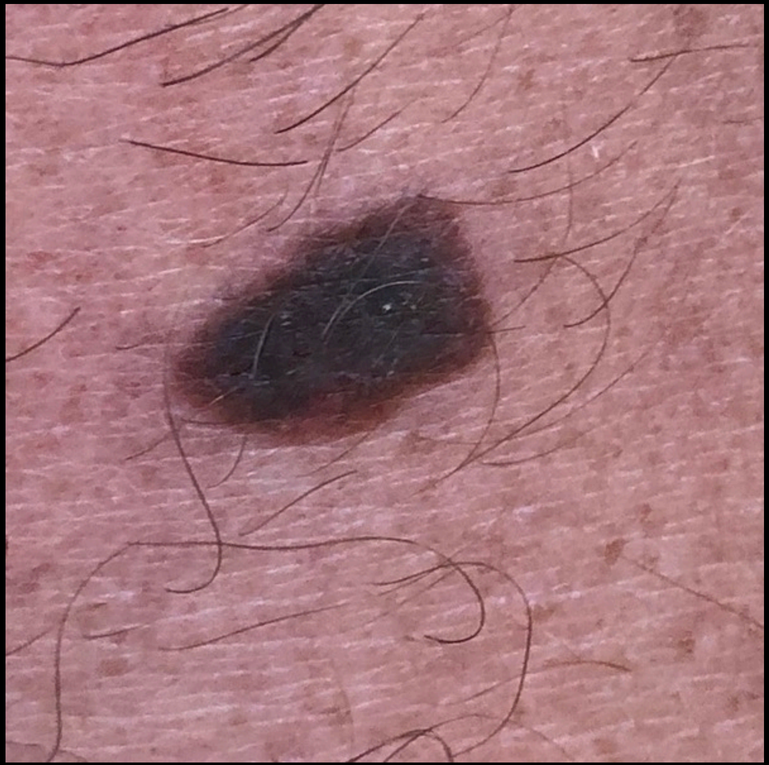

pTis

Case number 39

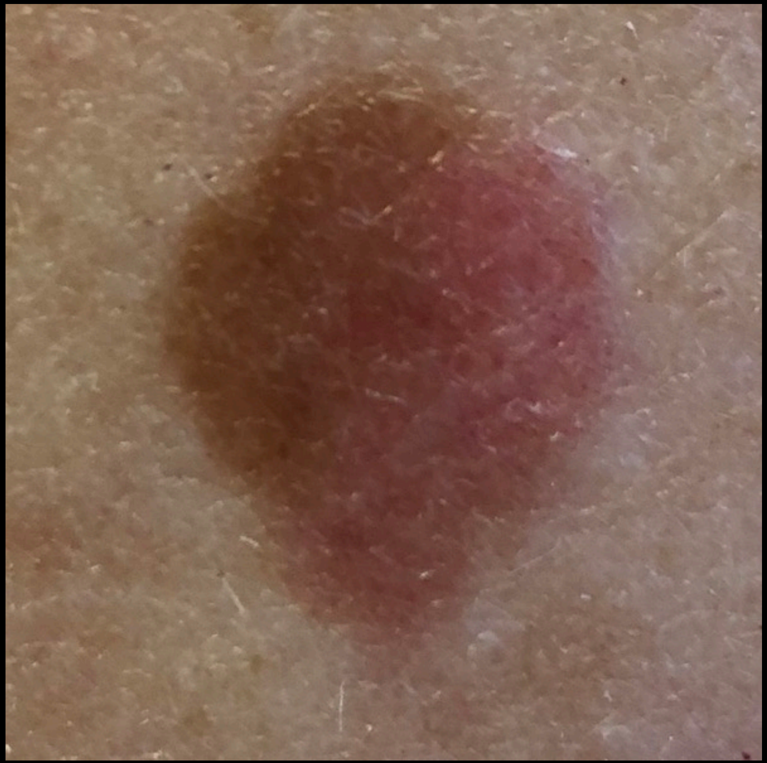

pTis

Case number 40

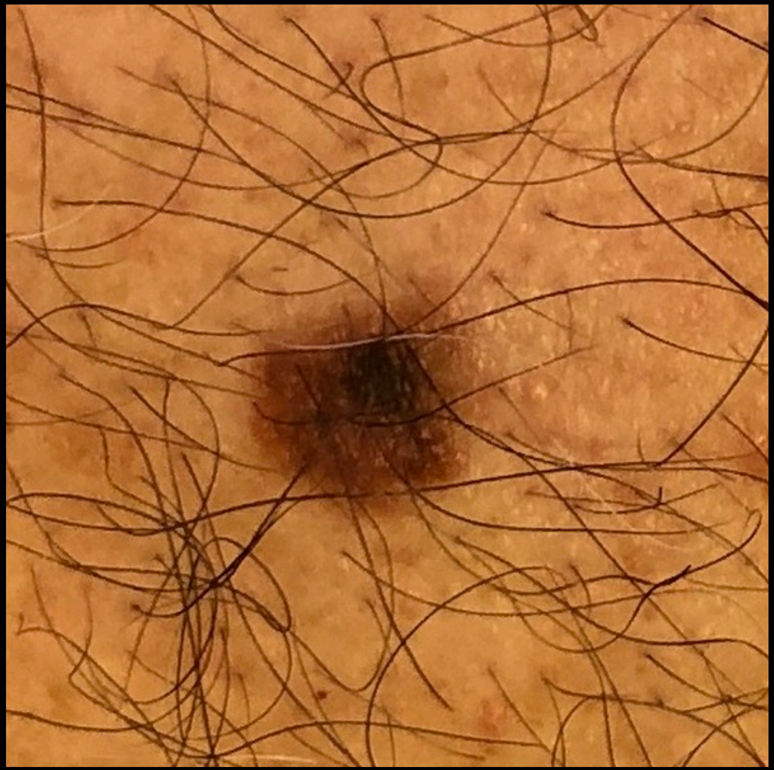

pTis

Case number 41

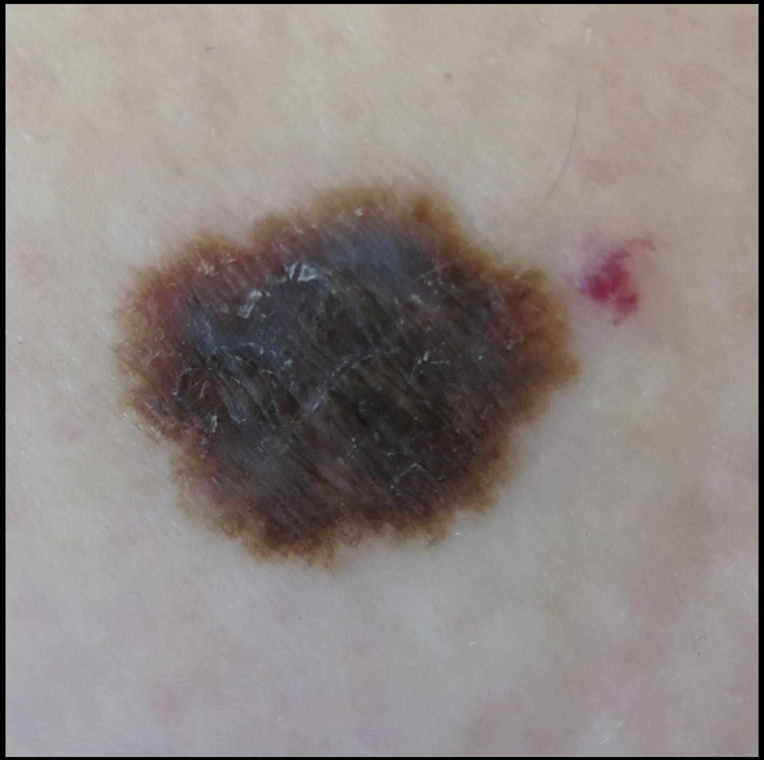

pT1a, 0.9 mm

Case number 42

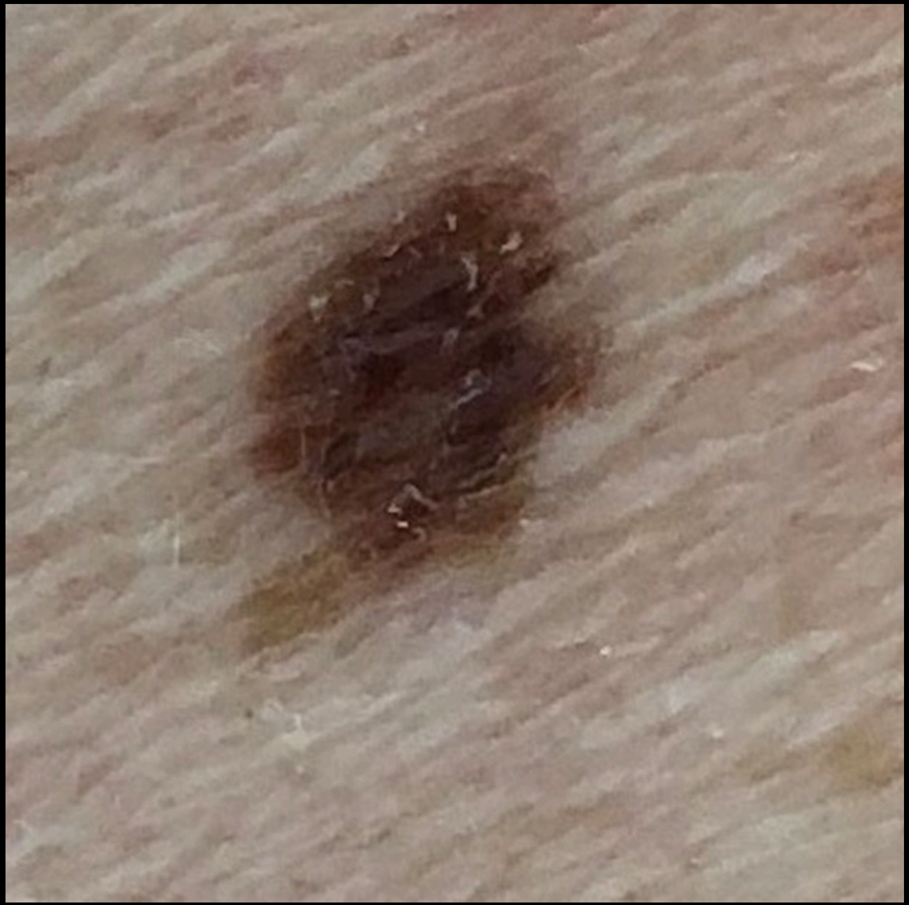

pT1a, 0.4 mm

Case number 43

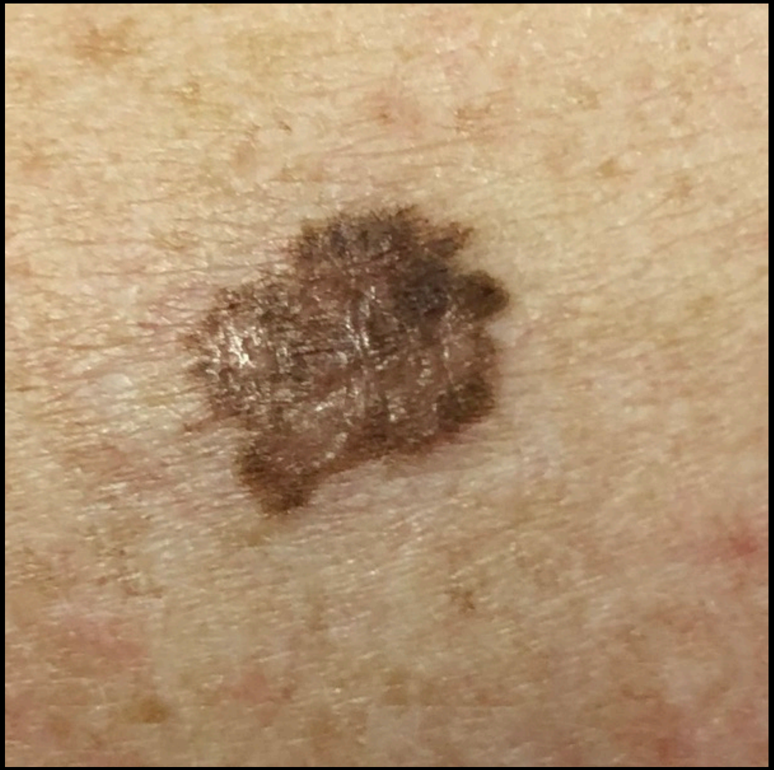

pTis

Case number 44

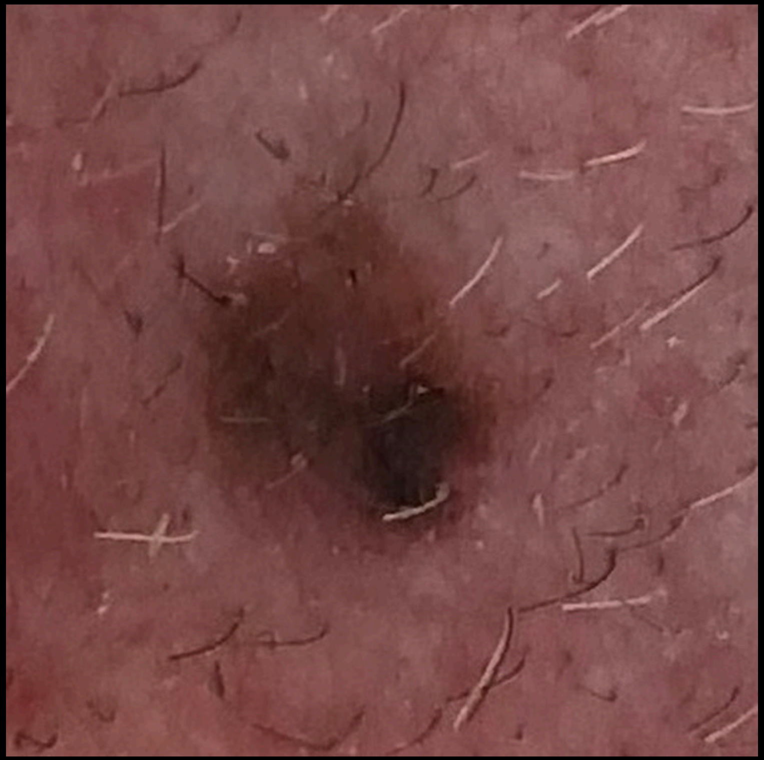

pTis

Case number 45

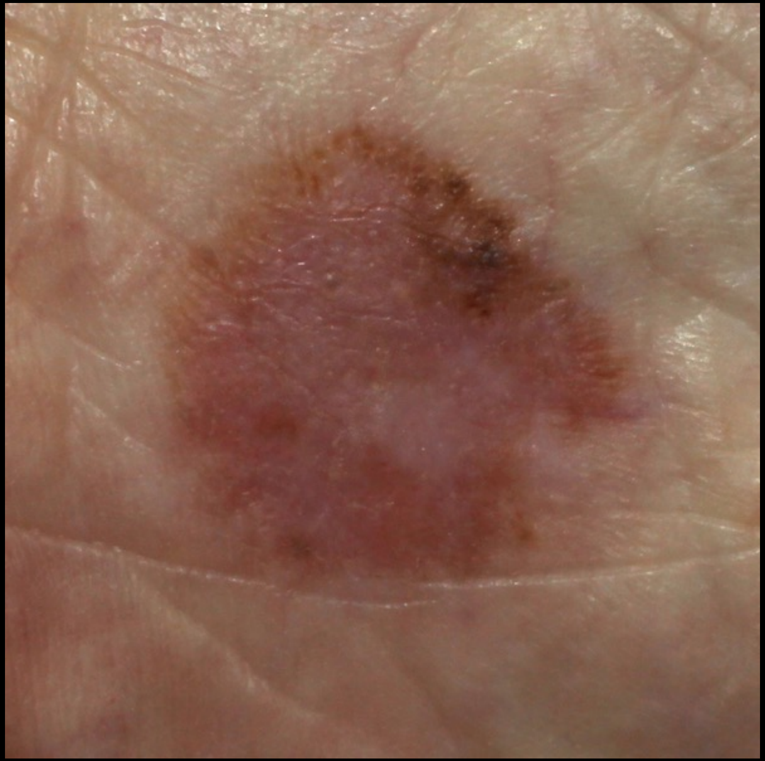

pT1a, 0.7 mm

Case number 46

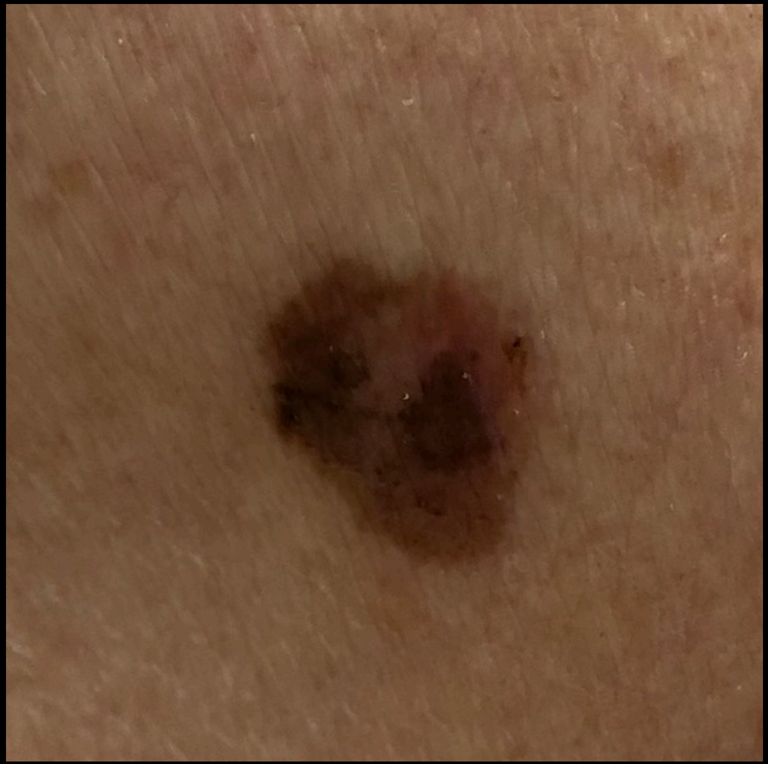

pTis

Case number 47

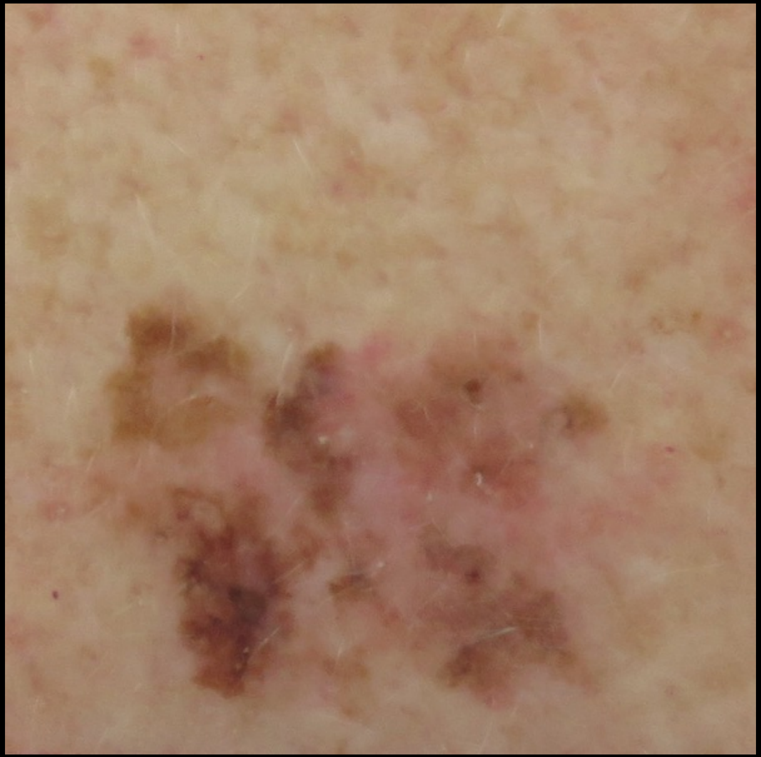

pTis

Case number 48

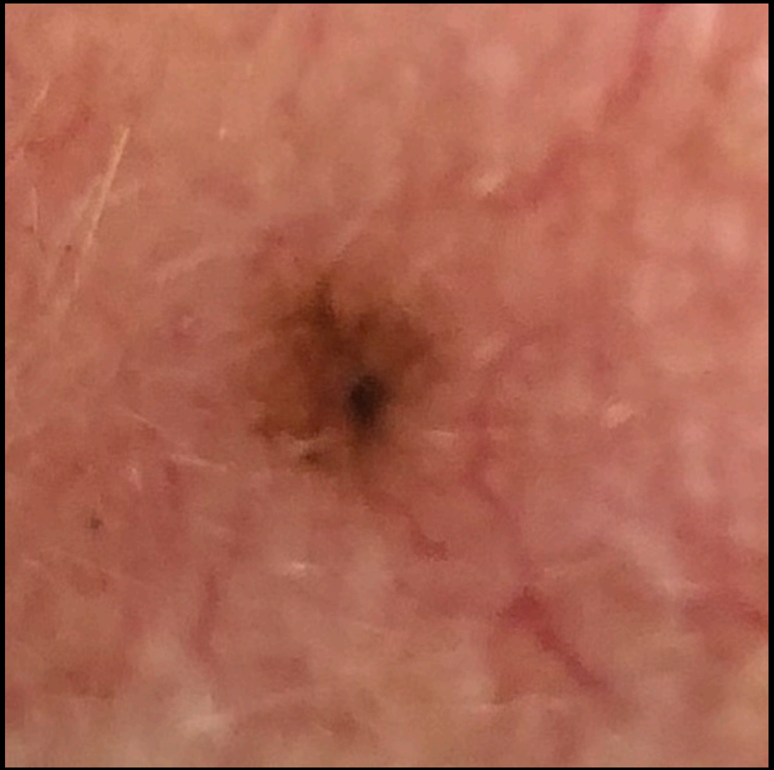

pT1a, 0.4 mm

Case number 49

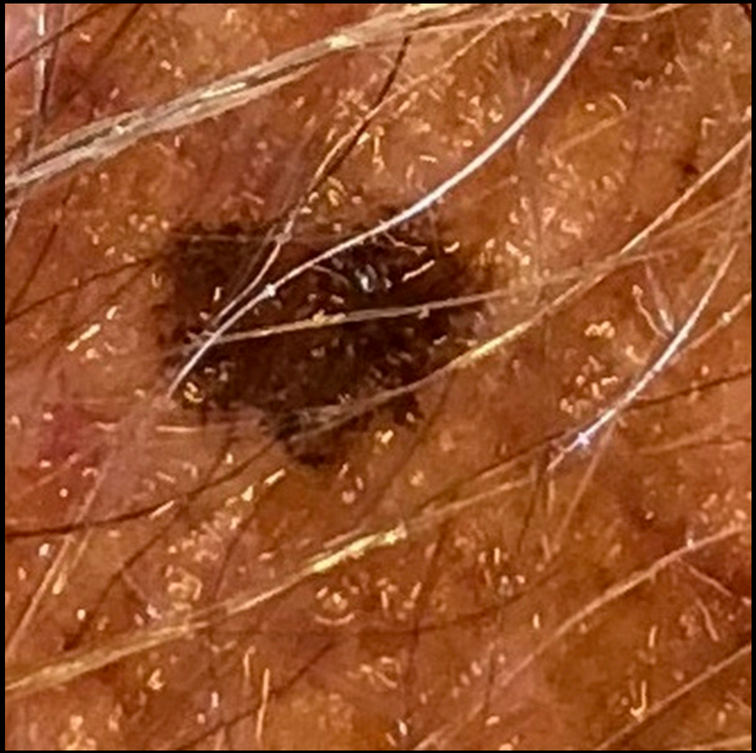

pTis

Case number 50

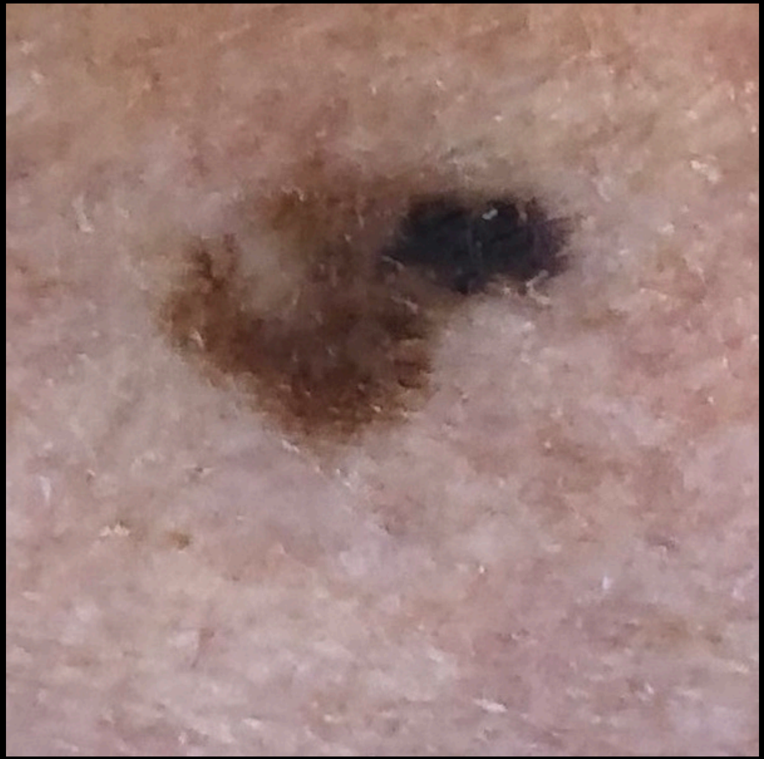

pT1a, 0.4 mm

Case number 51

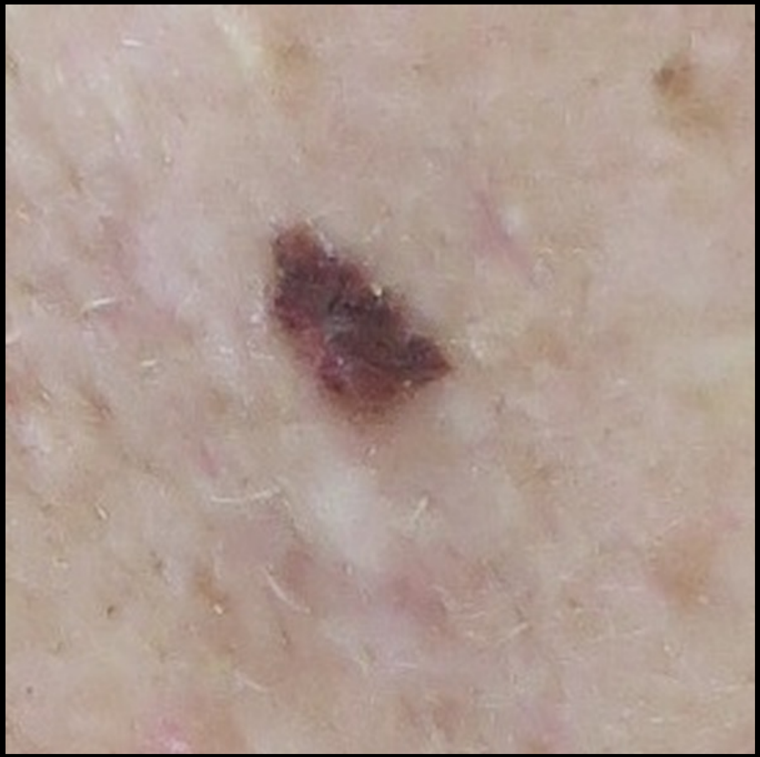

pT1a, 0.2 mm

Case number 52

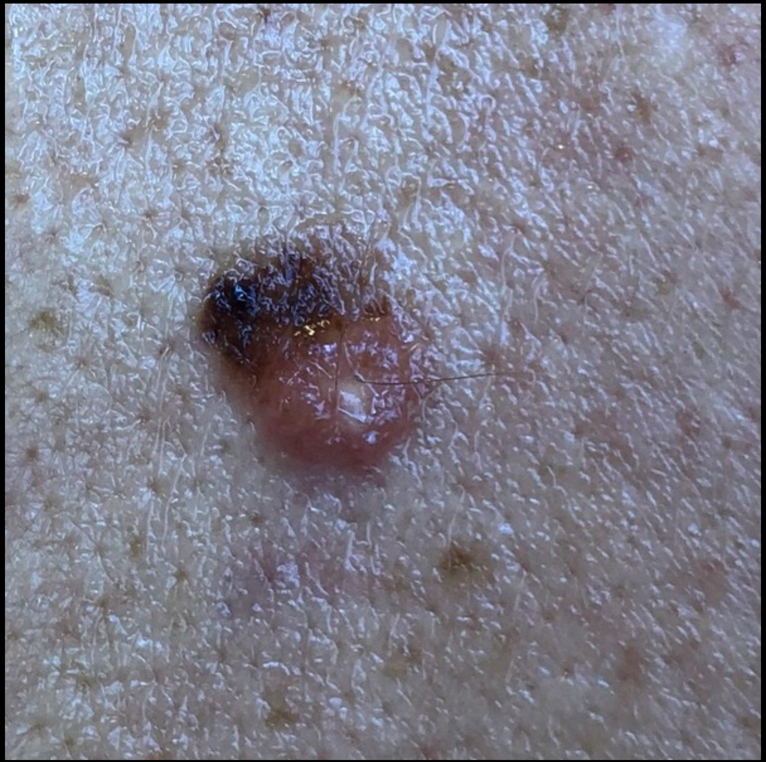

pTis

Case number 53

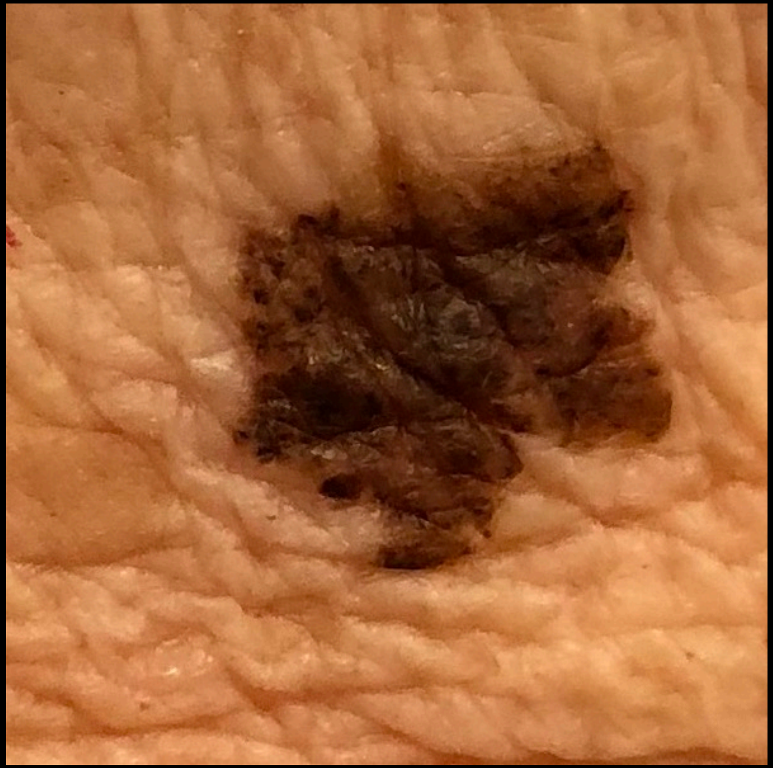

pTis

Case number 54

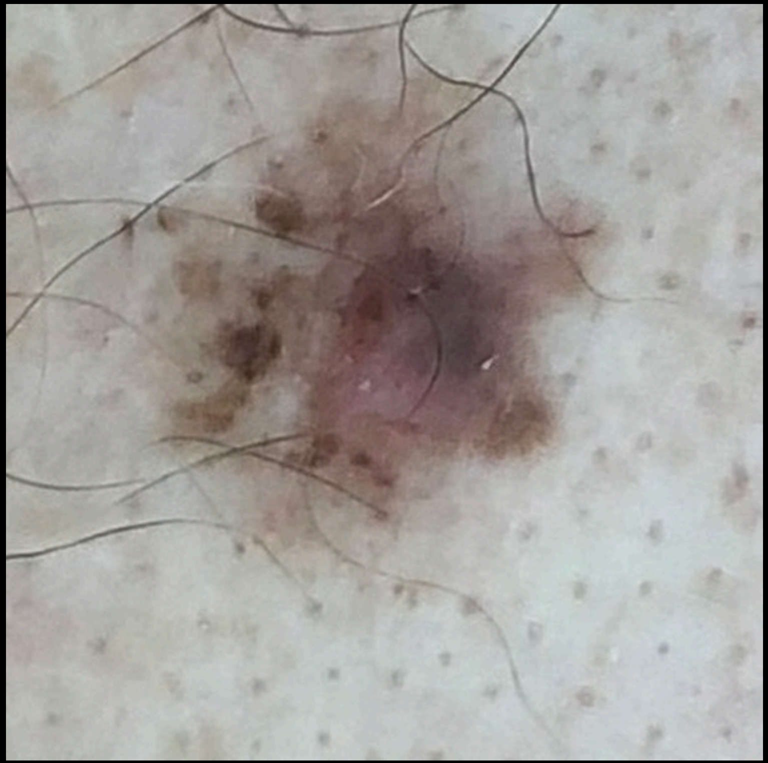

pT1a, 0.7 mm

Case number 55

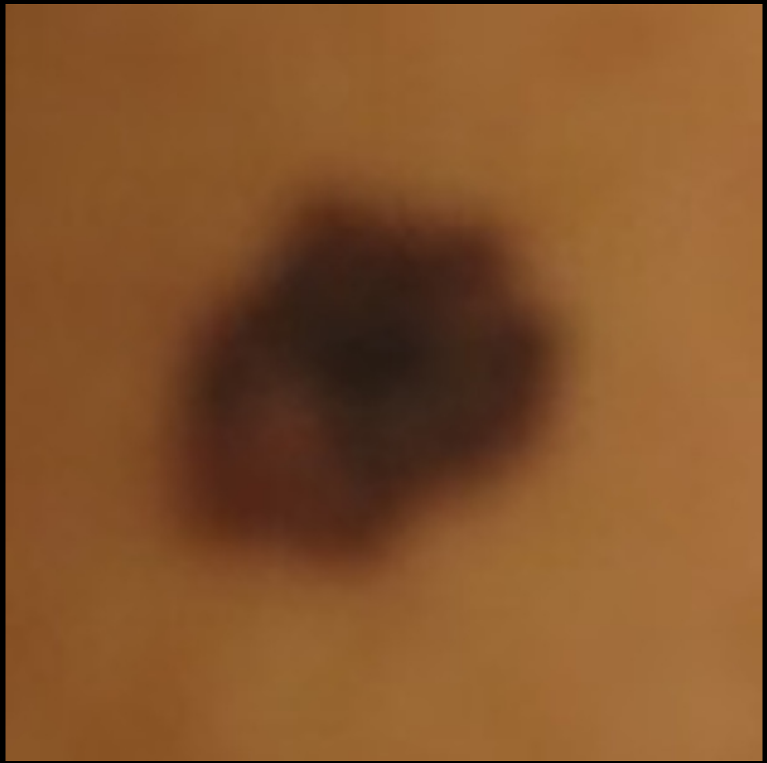

pT1b, 0.5 mm

Case number 56

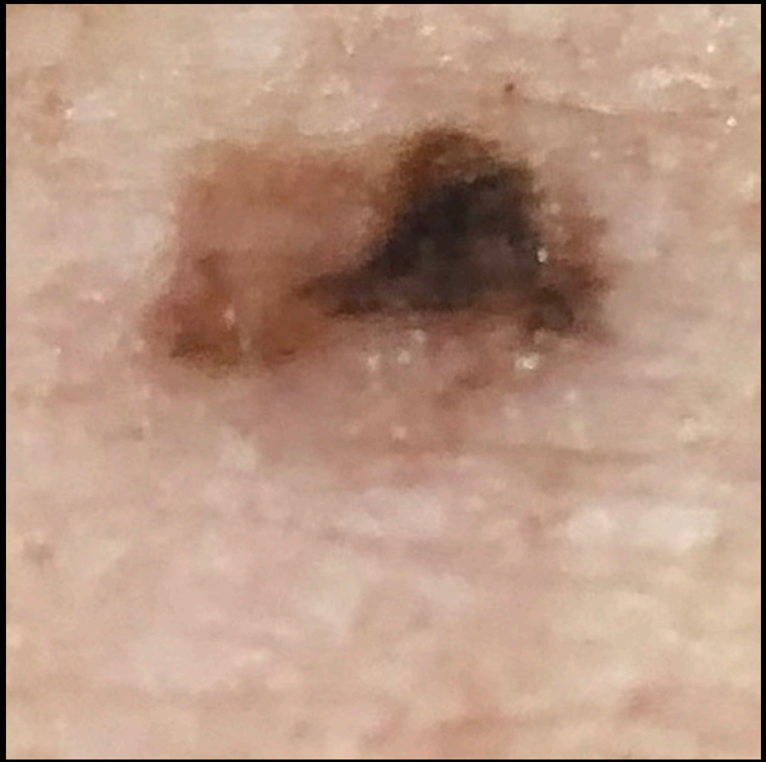

pTis

Case number 57

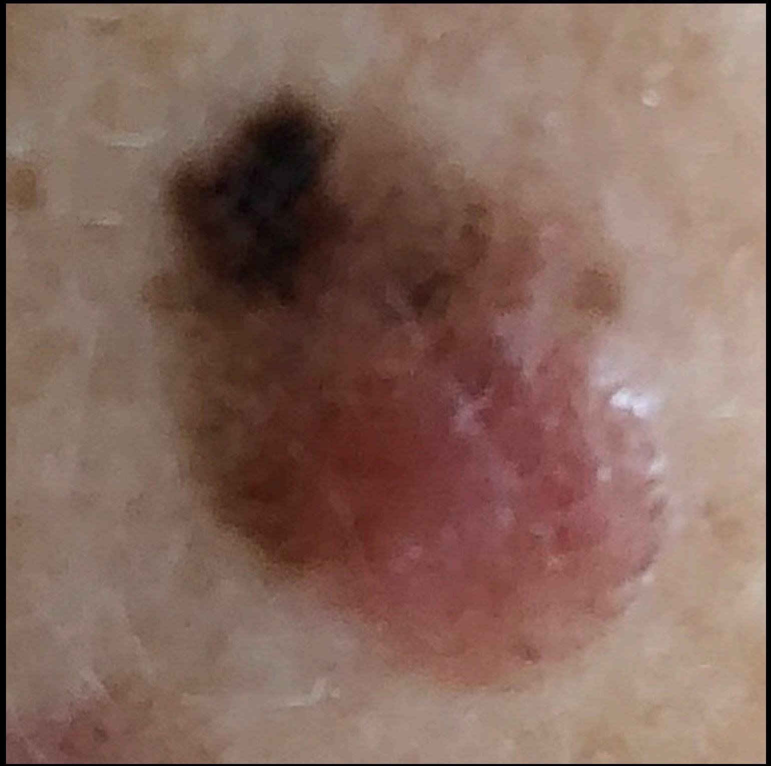

pT3b, 2.7 mm

Case number 58

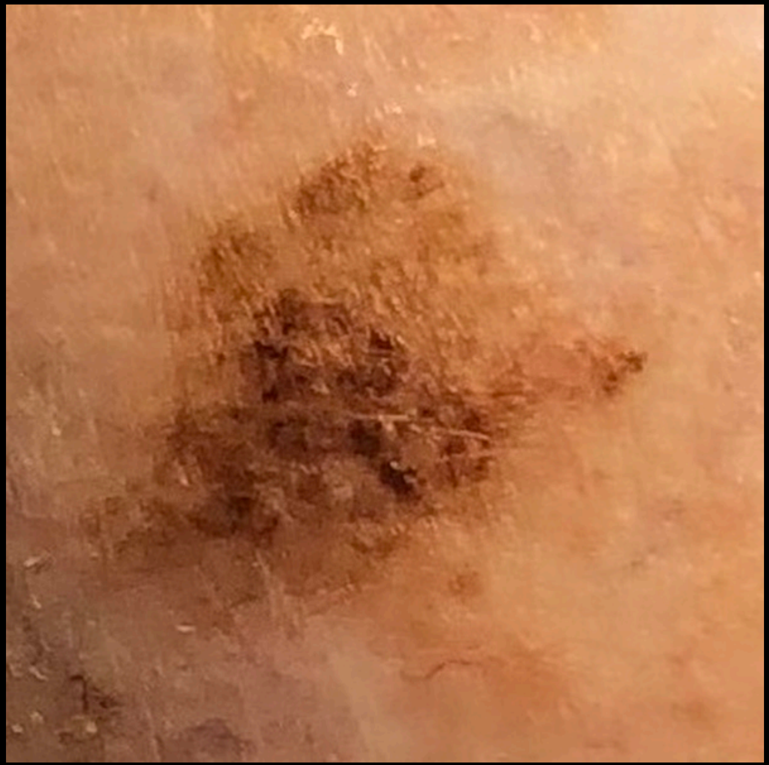

pTis

Case number 59

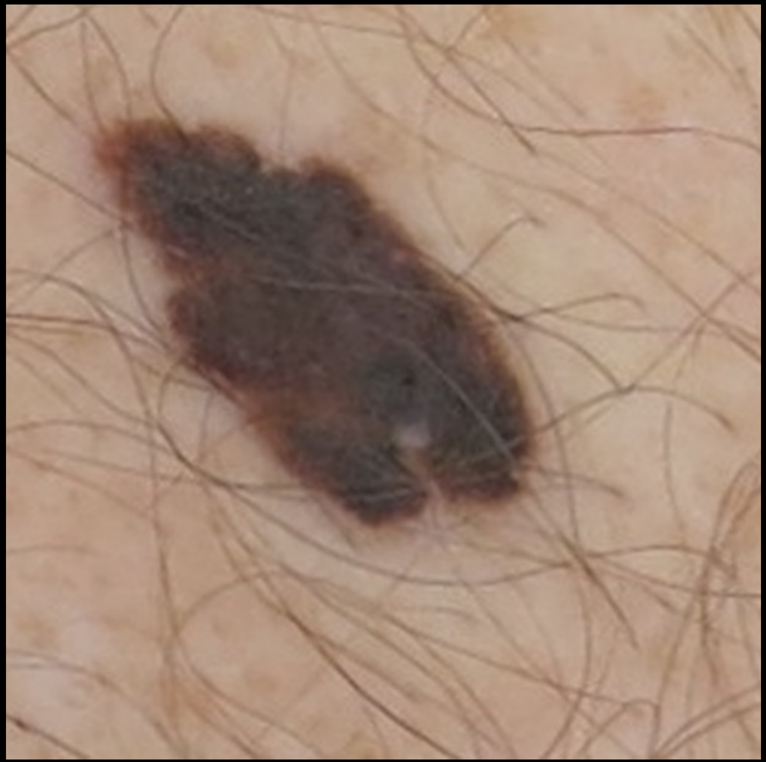

pT1a, 0.6 mm

Case number 60

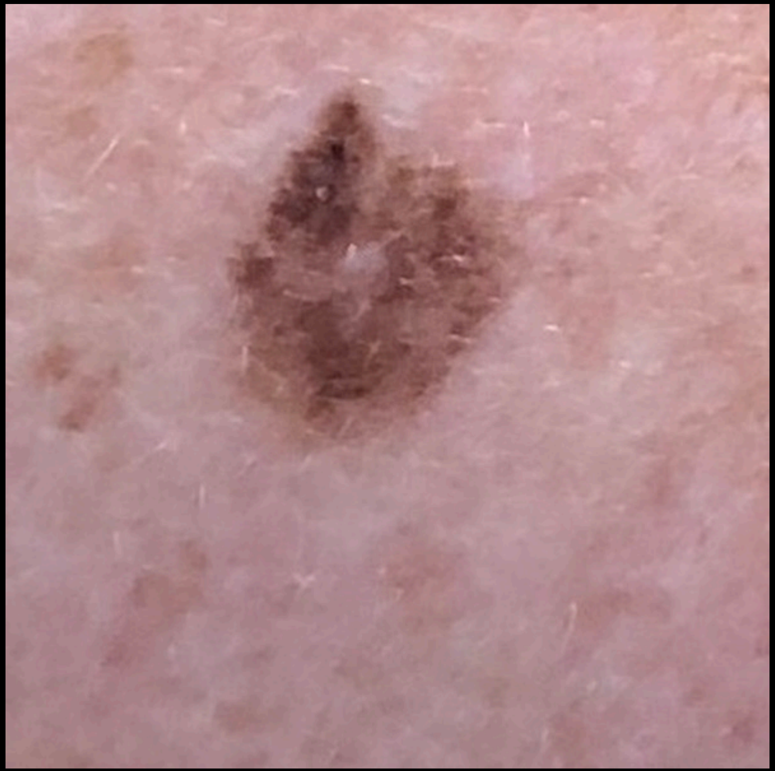

pTis

Case number 61

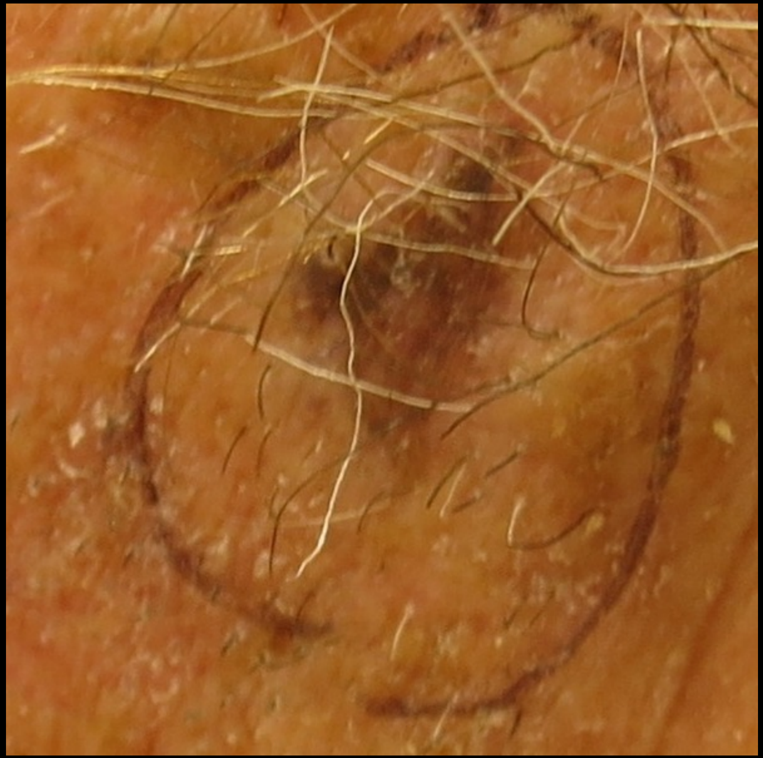

pTis

Case number 62

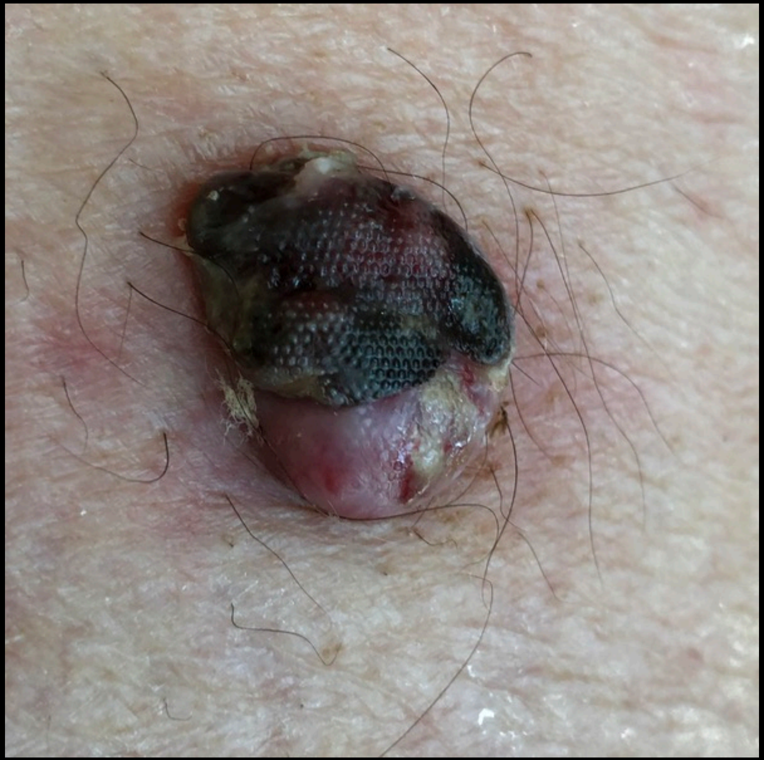

pT4b, 12 mm

Case number 63

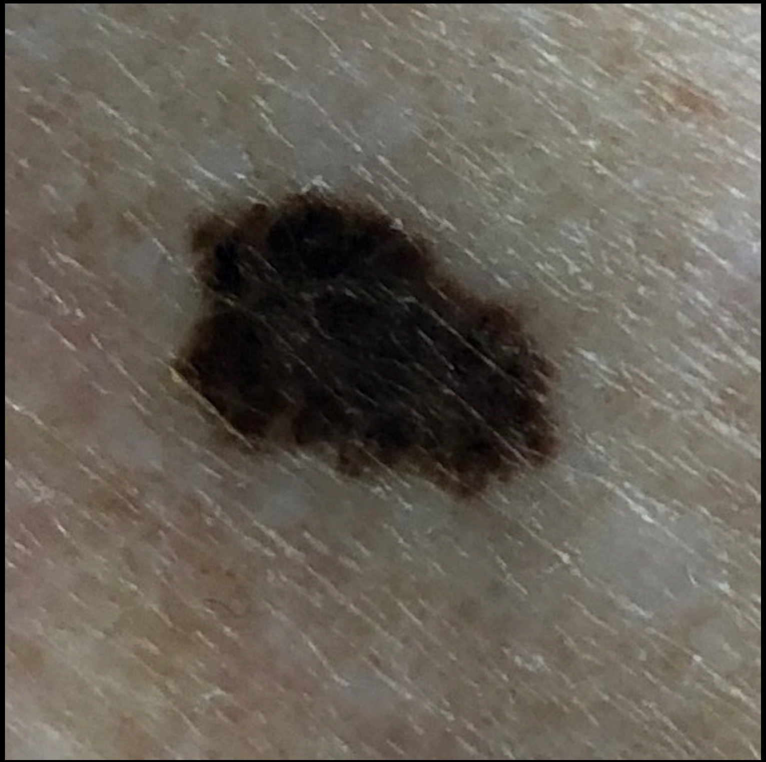

pTis

Case number 64

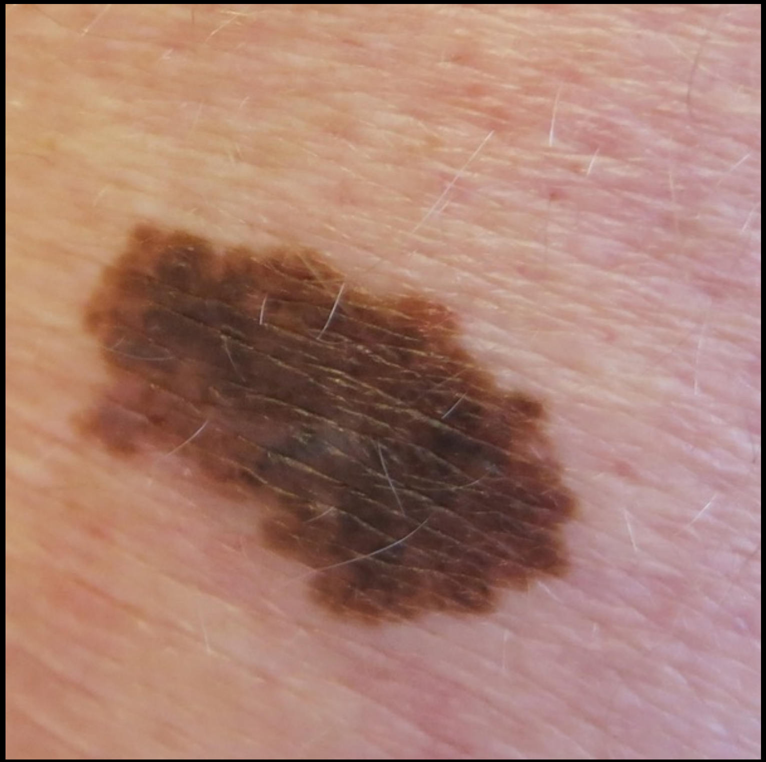

pT1a, 0.3 mm

Case number 65

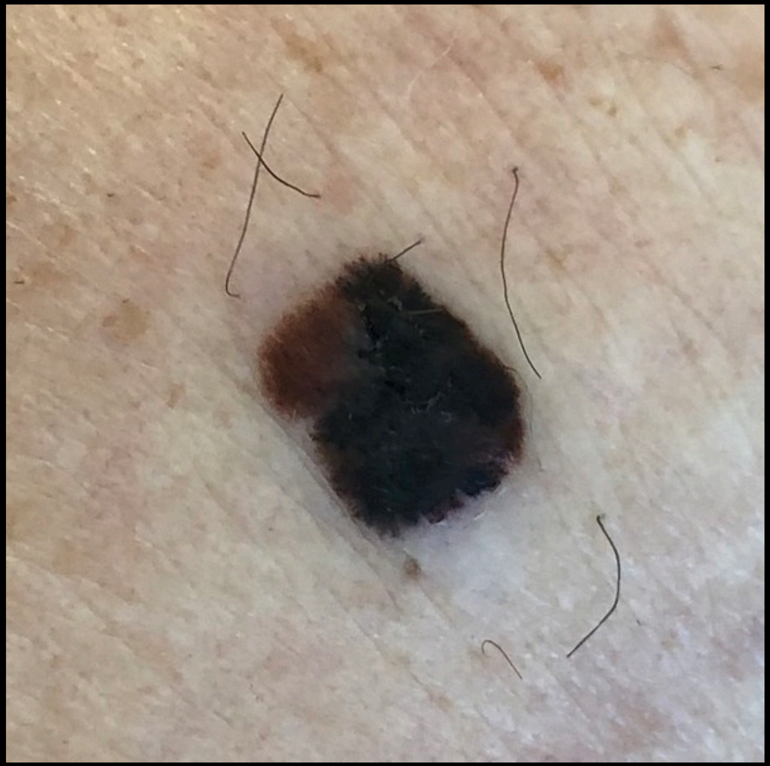

pT2a, 1.2 mm

Case number 66

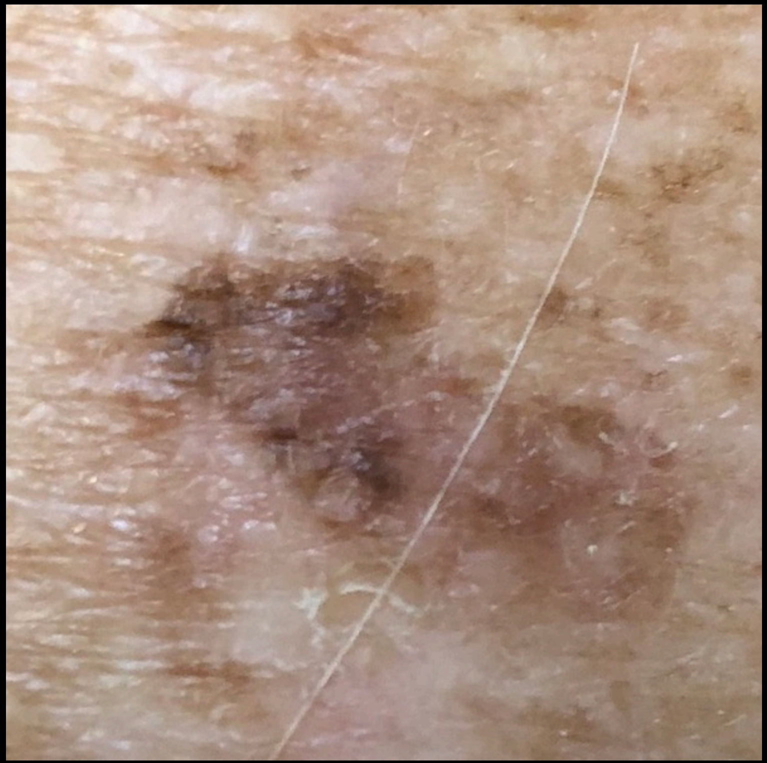

pTis

Case number 67

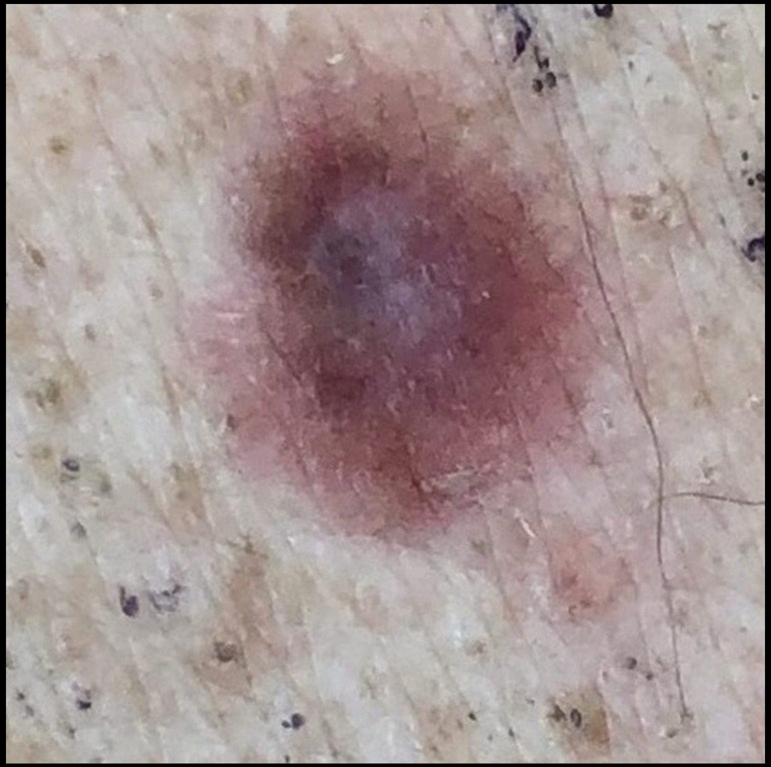

pT1a, 0.6 mm

Case number 68

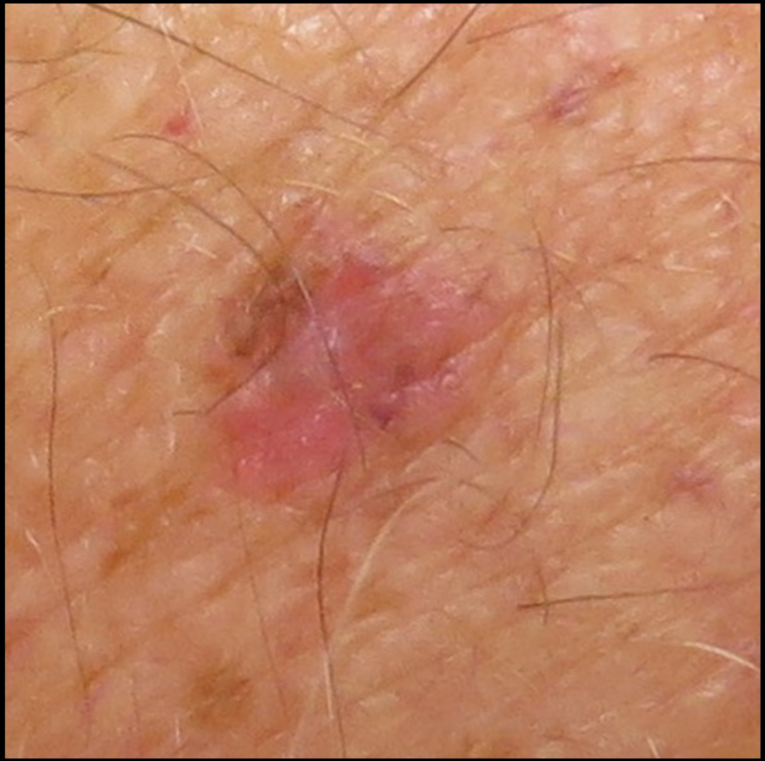

pT1a, 0.5 mm

Case number 69

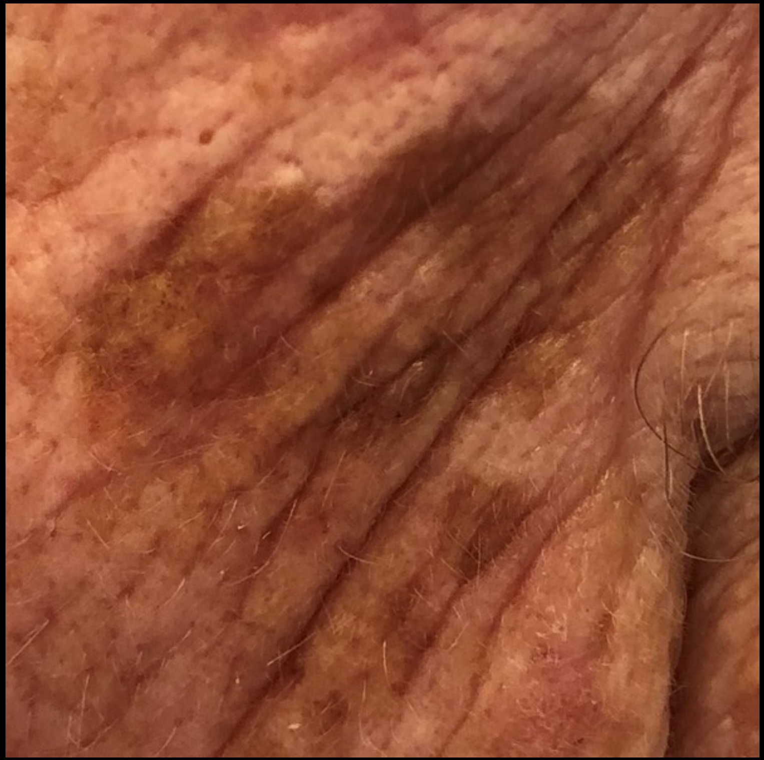

pTis

Case number 70

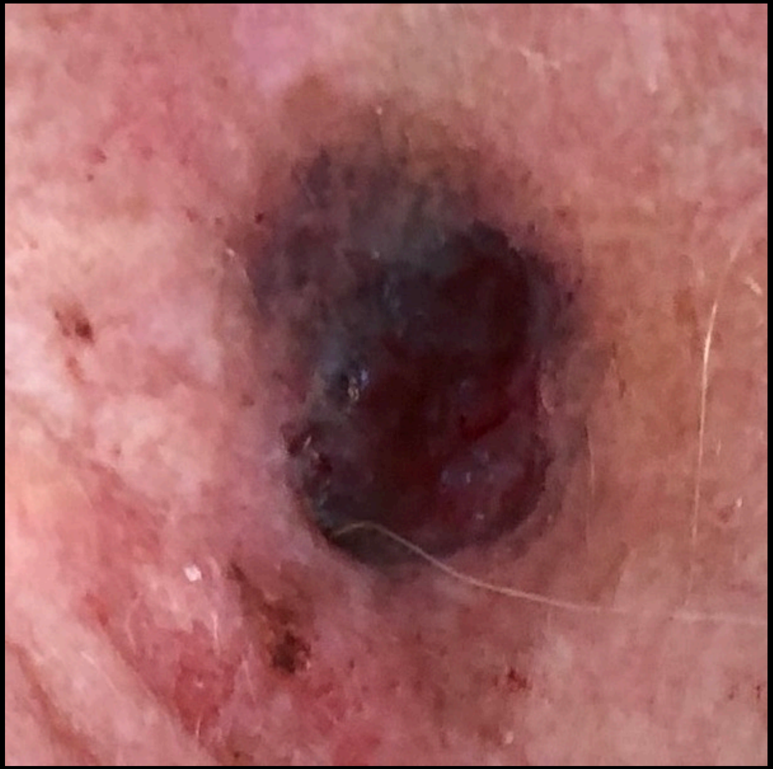

pT3b, 2.1 mm

Case number 71

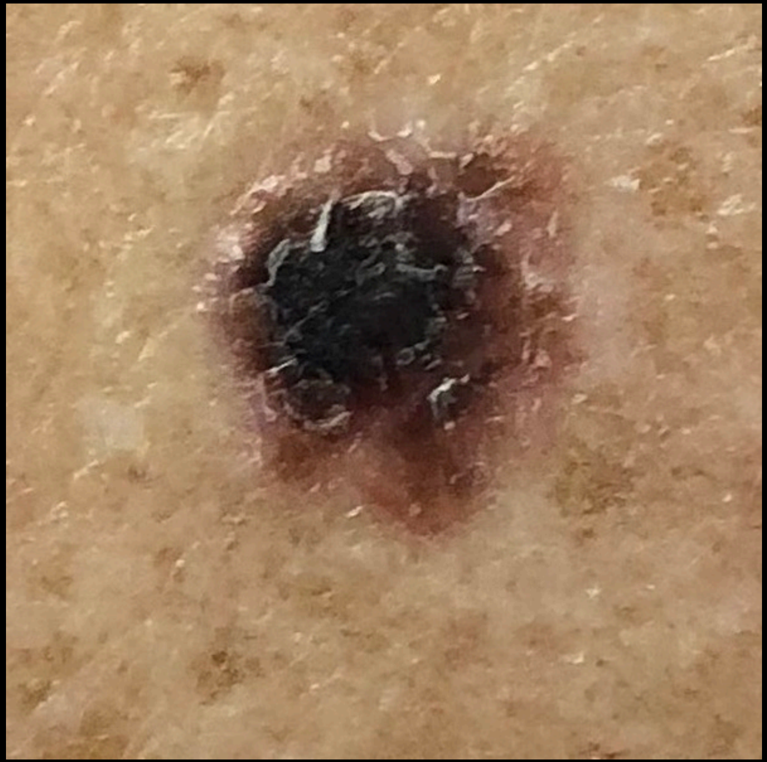

pT2a, 1.5 mm

Case number 72

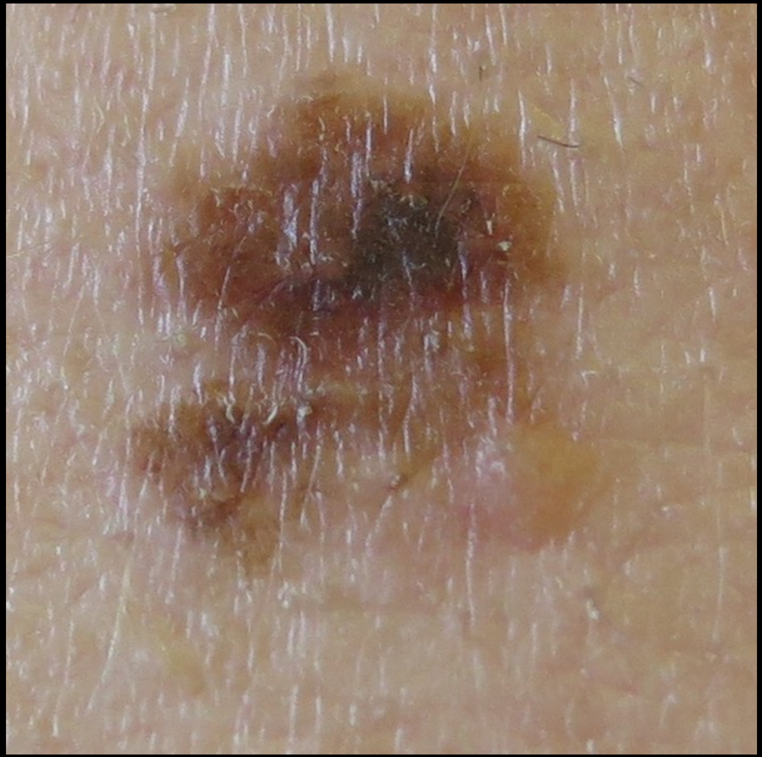

pTis

Case number 73

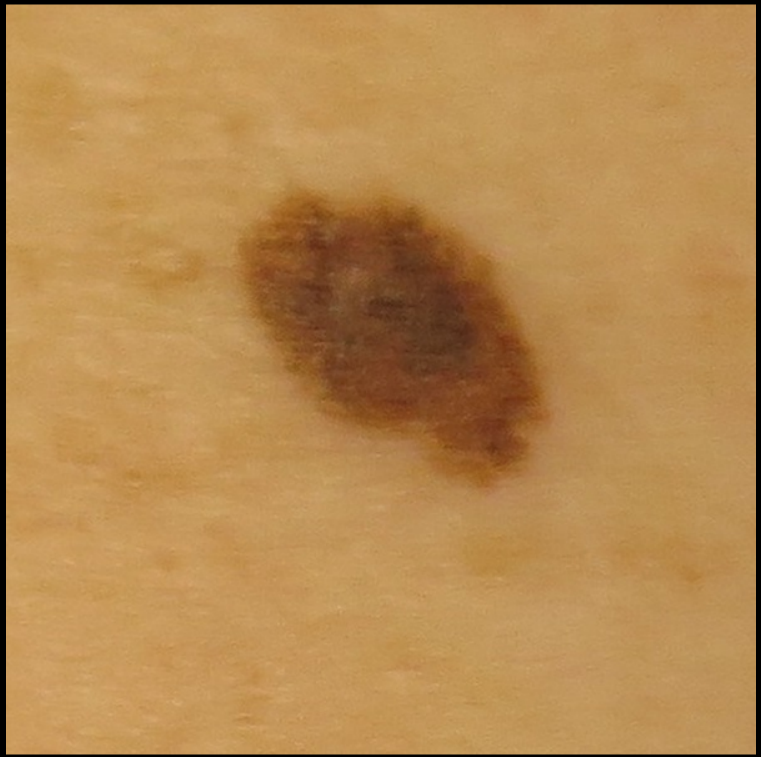

pTis

Case number 74

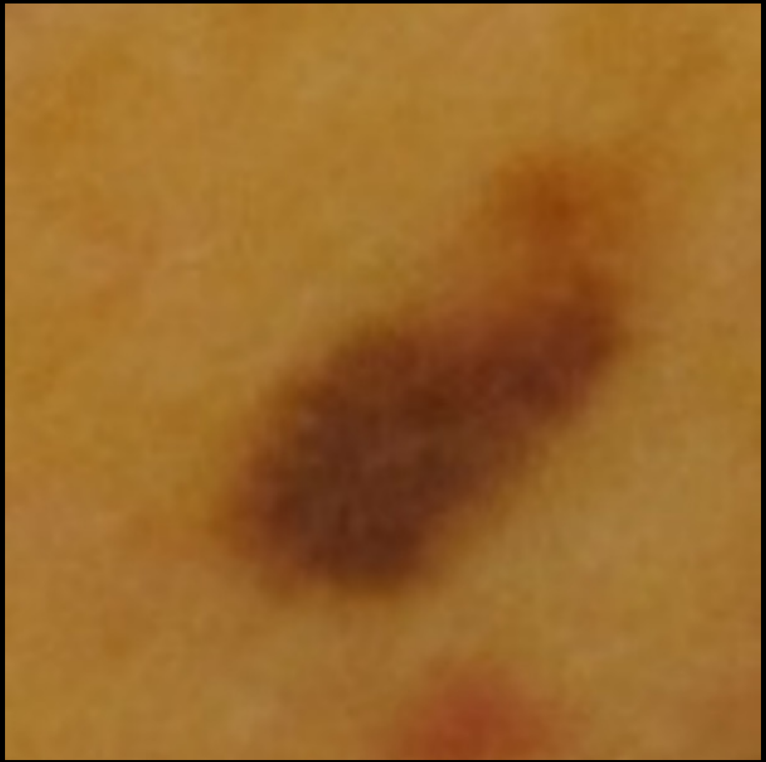

pTis

Case number 75

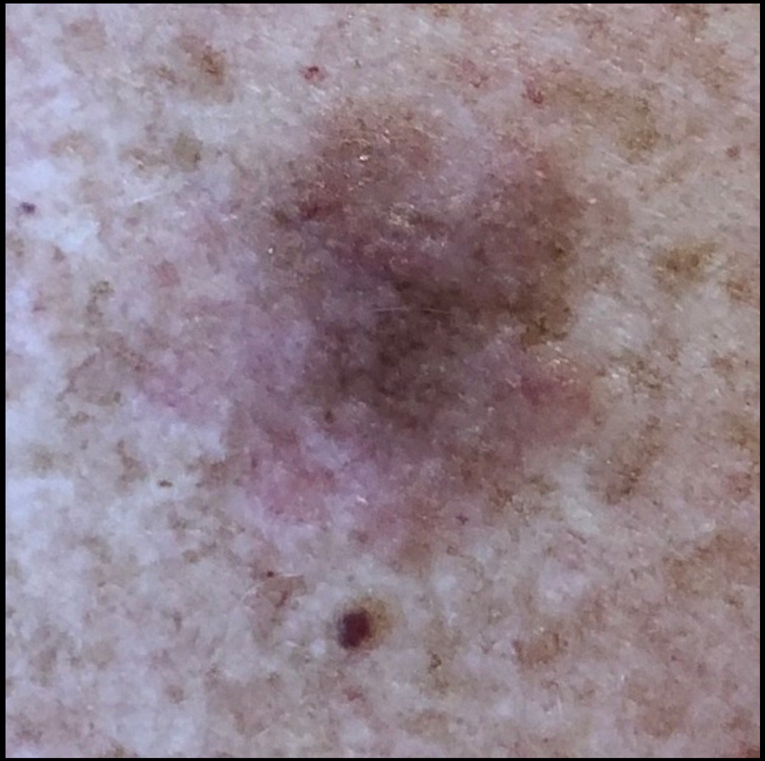

pTis

Case number 76

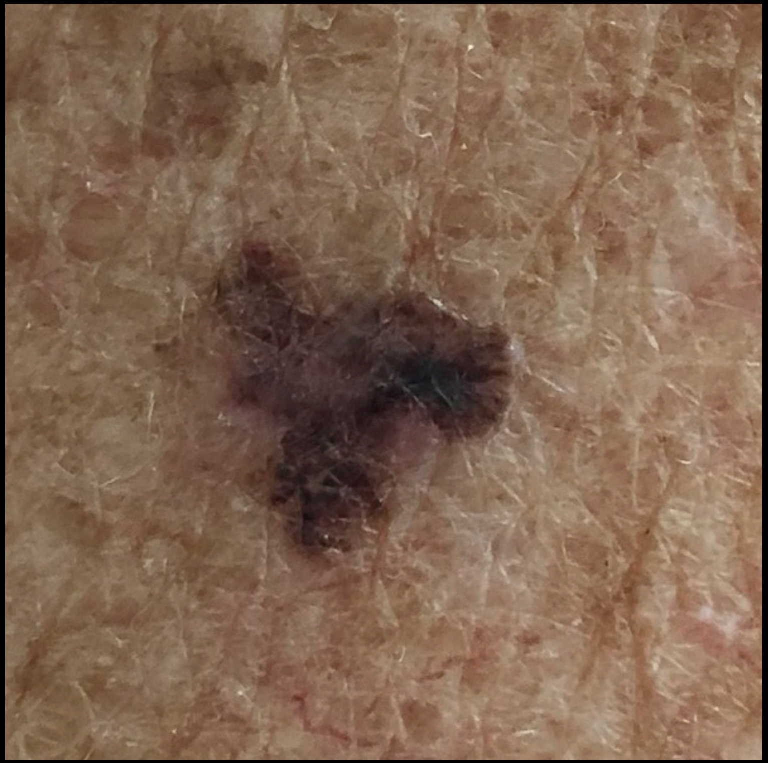

pT1a, 0.3 mm

Case number 77

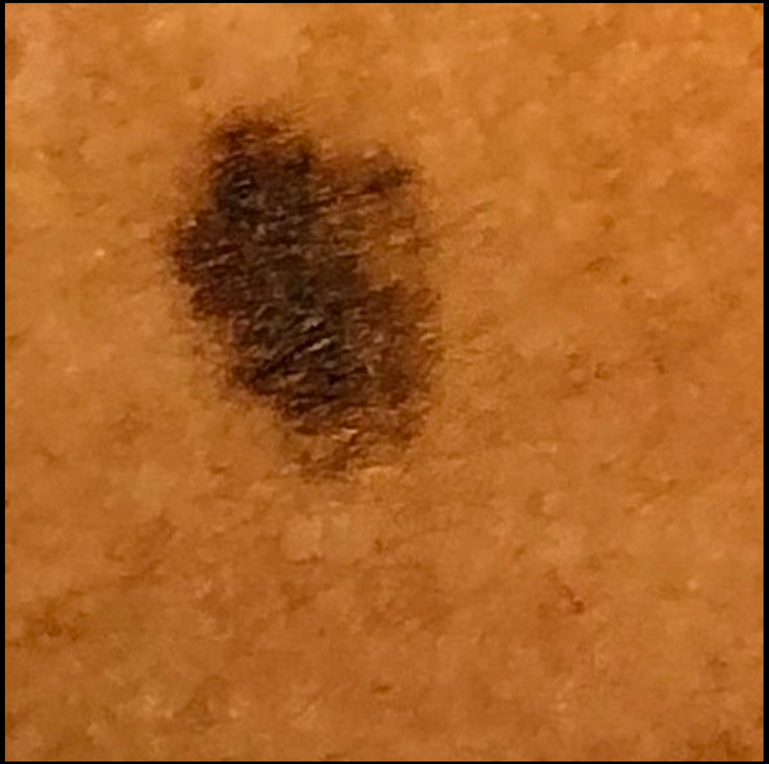

pTis

Case number 78

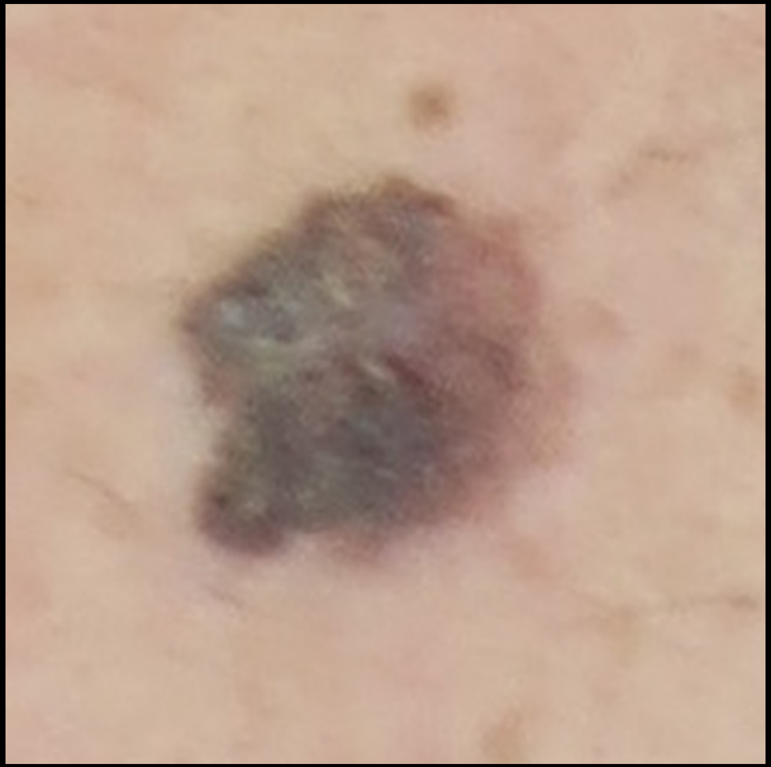

pT1a, 0.5 mm

Case number 79

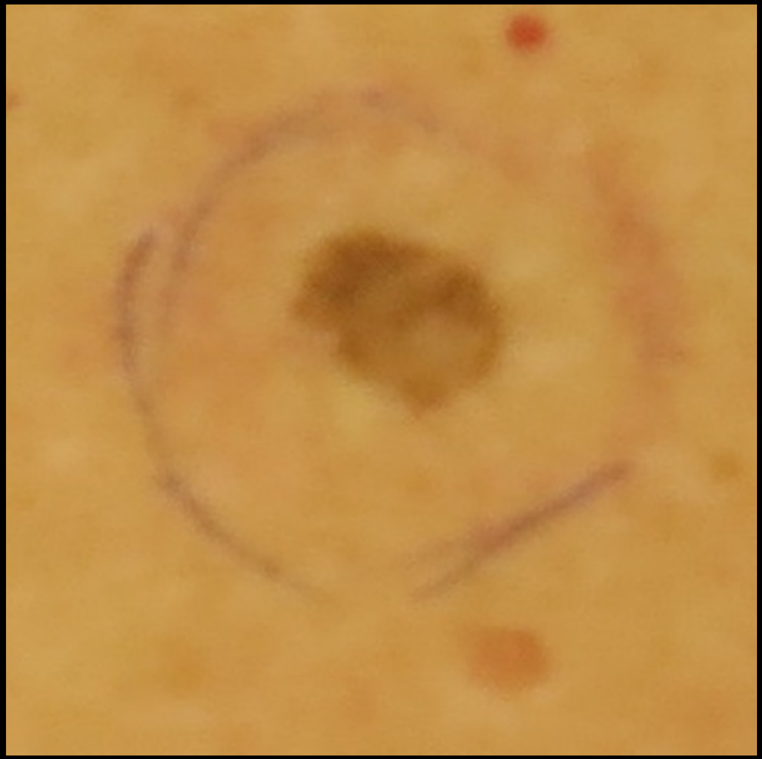

pTis

Case number 80

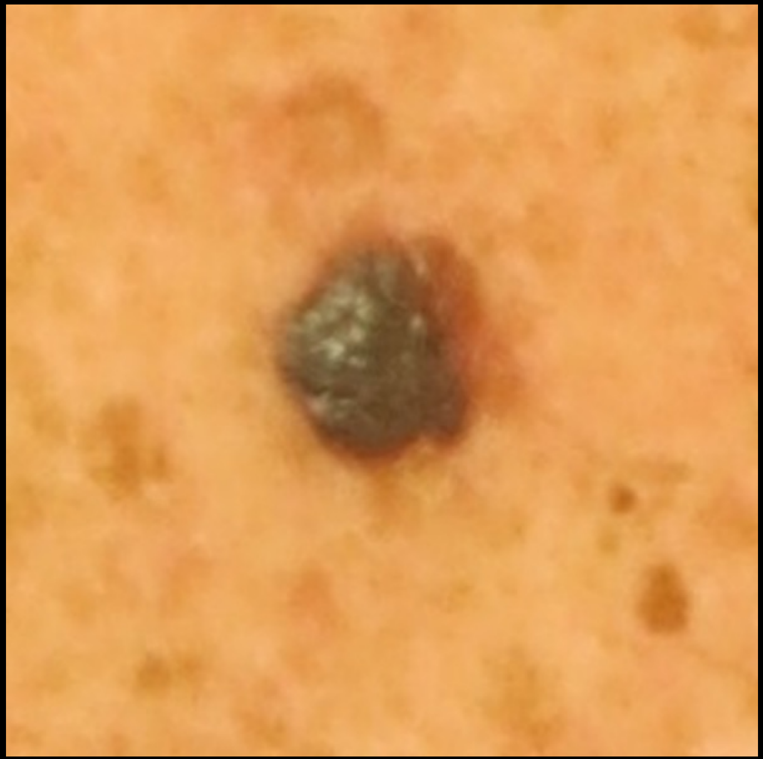

pT2a, 1.8 mm

Case number 81

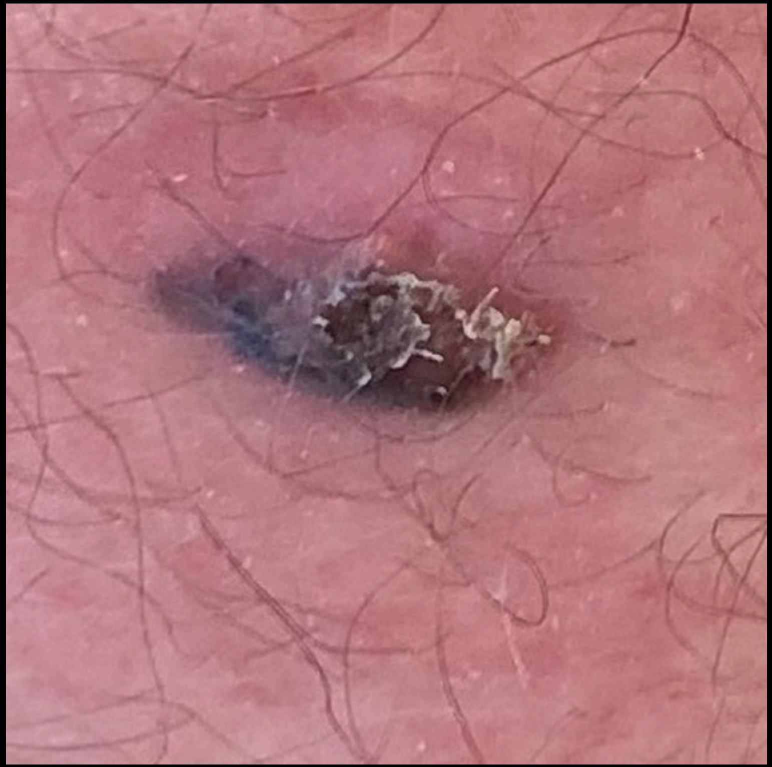

pT3b, 2.2 mm

Case number 82

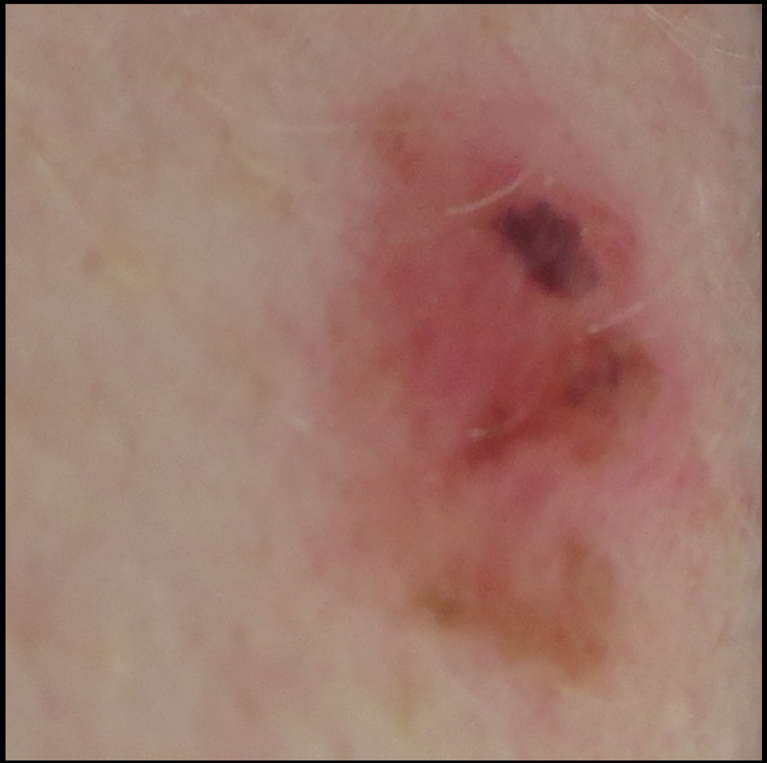

pT1a, 0.6 mm

Case number 83

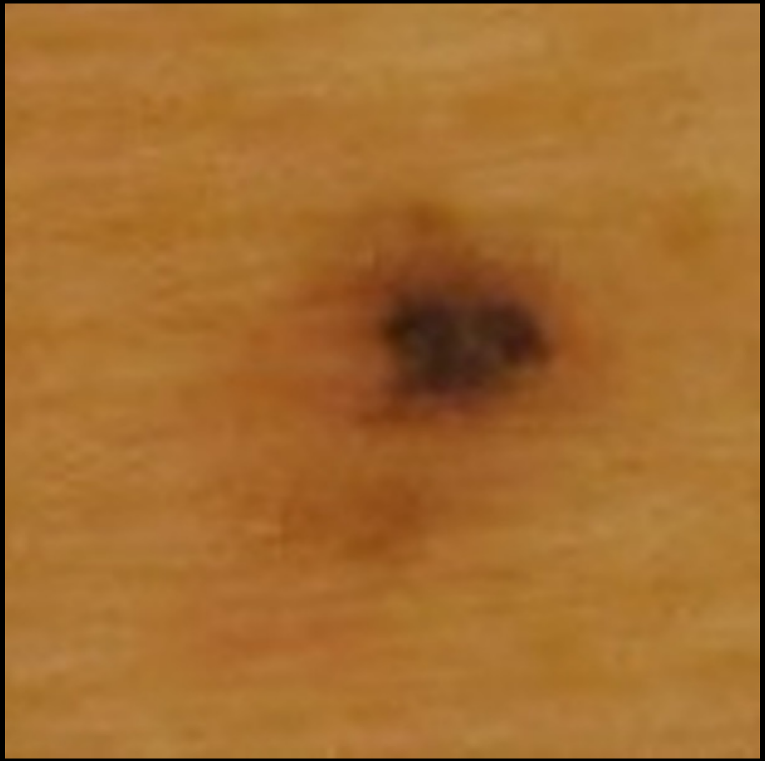

pTis

Case number 84

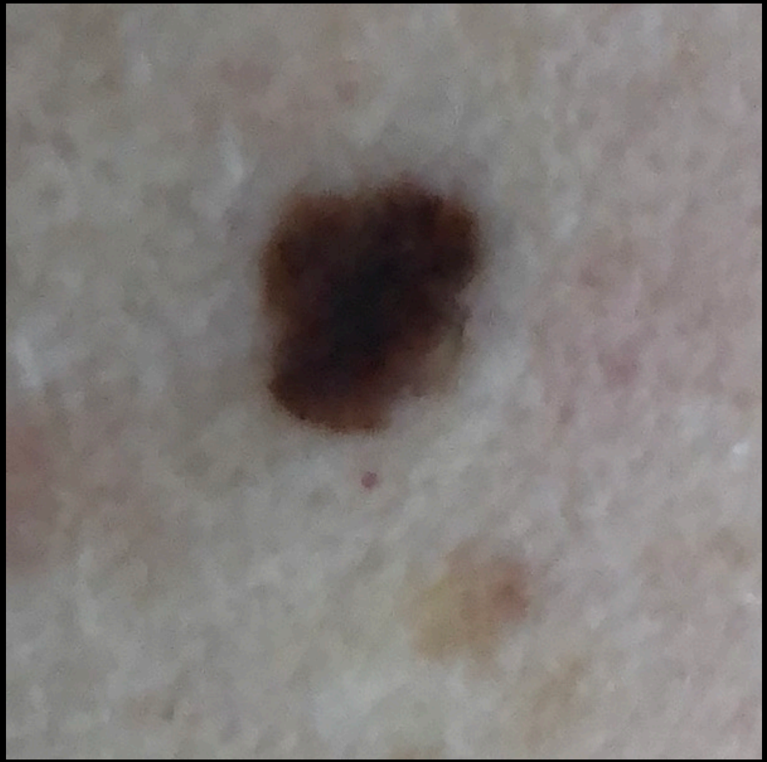

pT1a, 0.7 mm

Case number 85

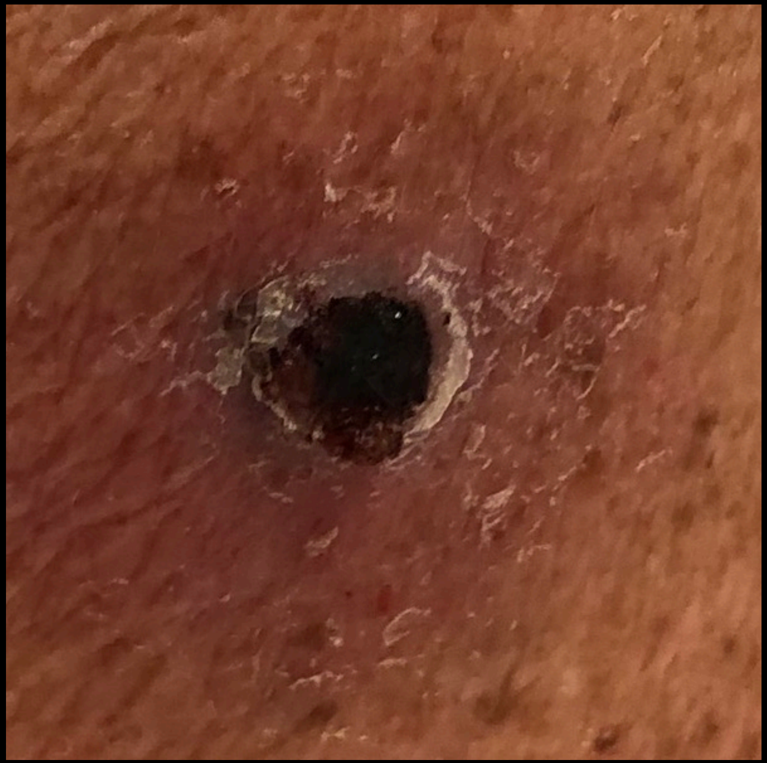

pT2b, 1.8 mm

Case number 86

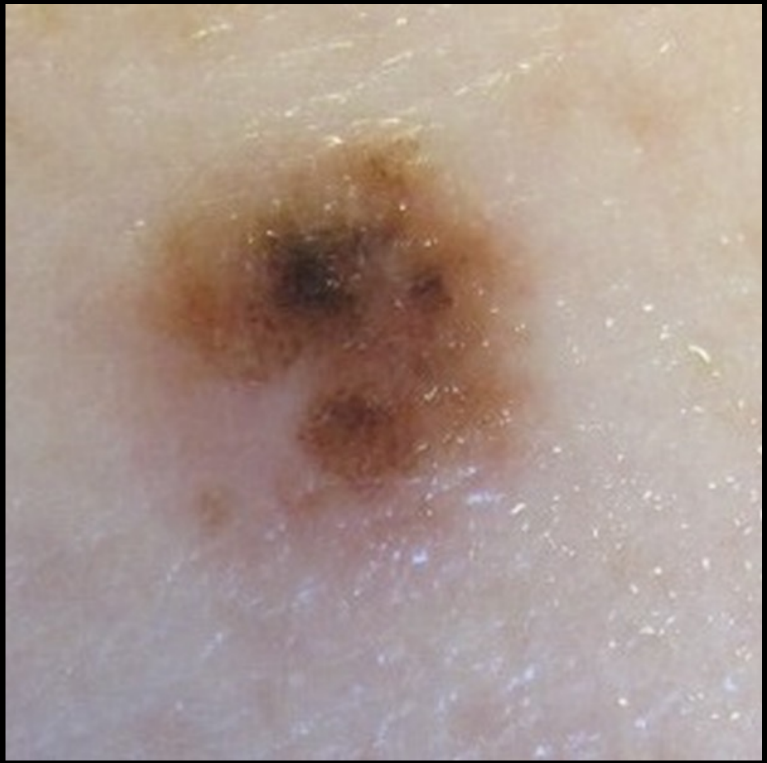

pT1a, 0.6 mm

Case number 87

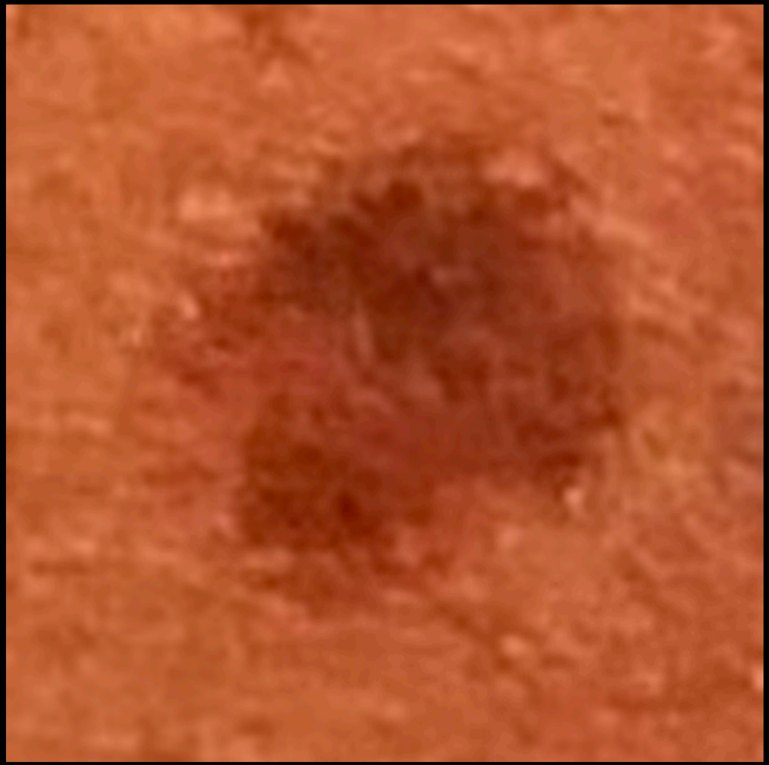

pTis

Case number 88

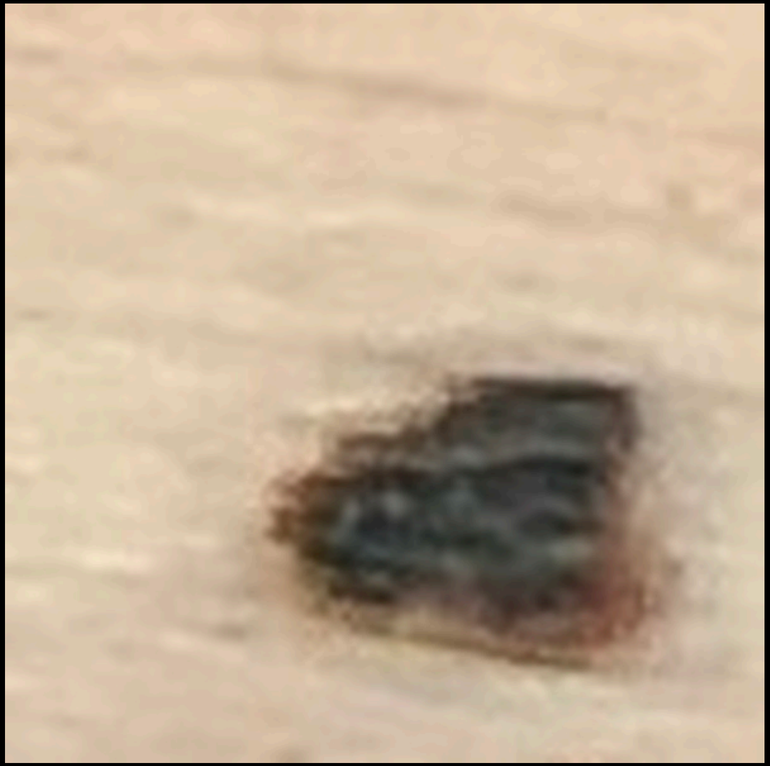

pT1a, 0.6 mm

Case number 89

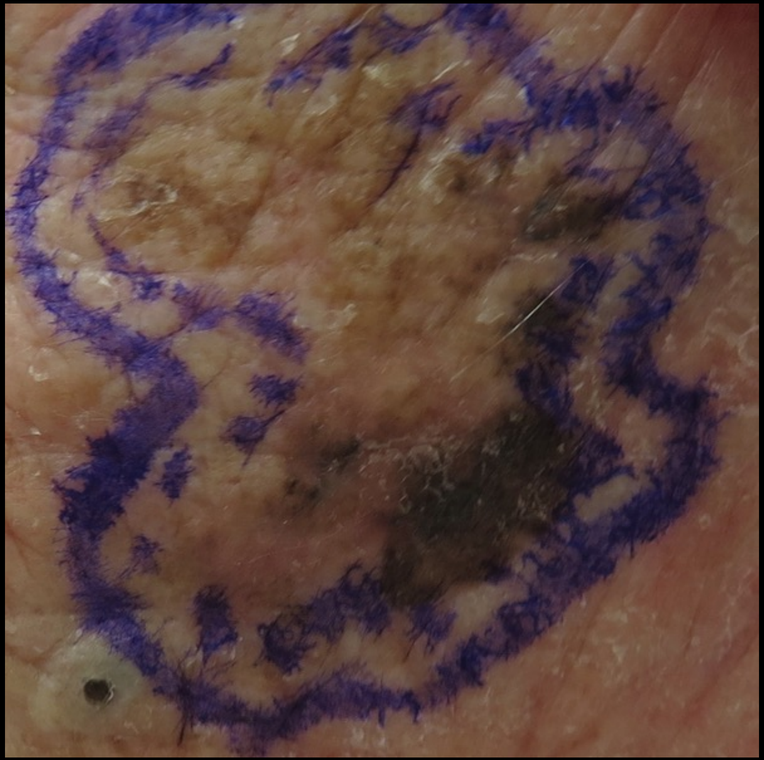

pT2a, 1.8 mm

Case number 90

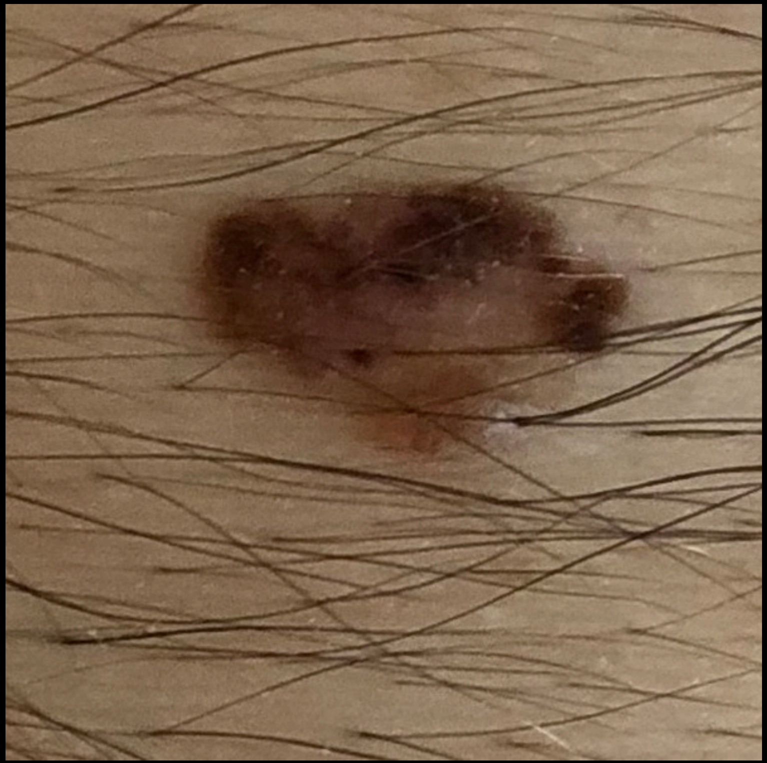

pT1a, 0.3 mm

Case number 91

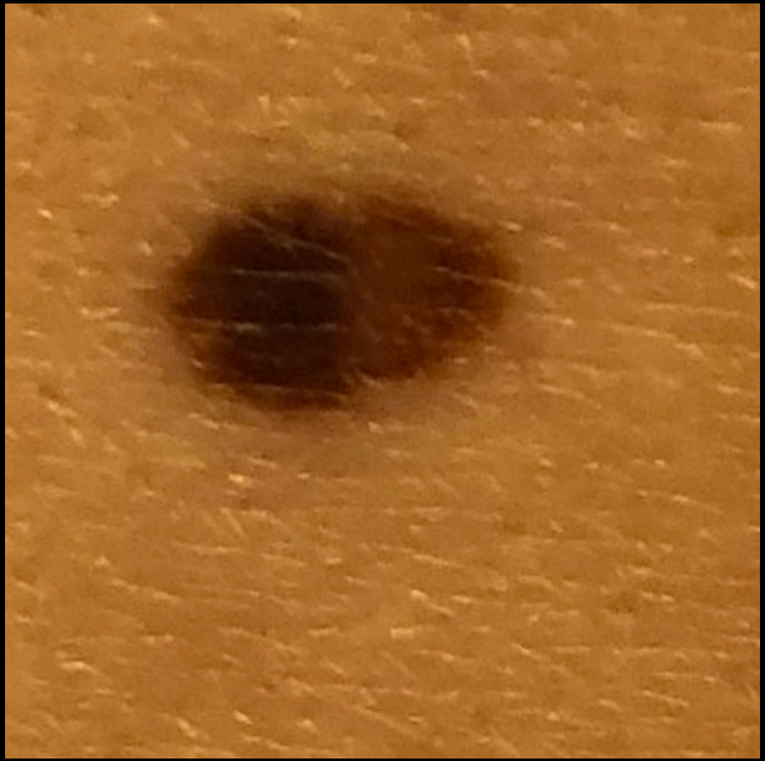

pTis

Case number 92

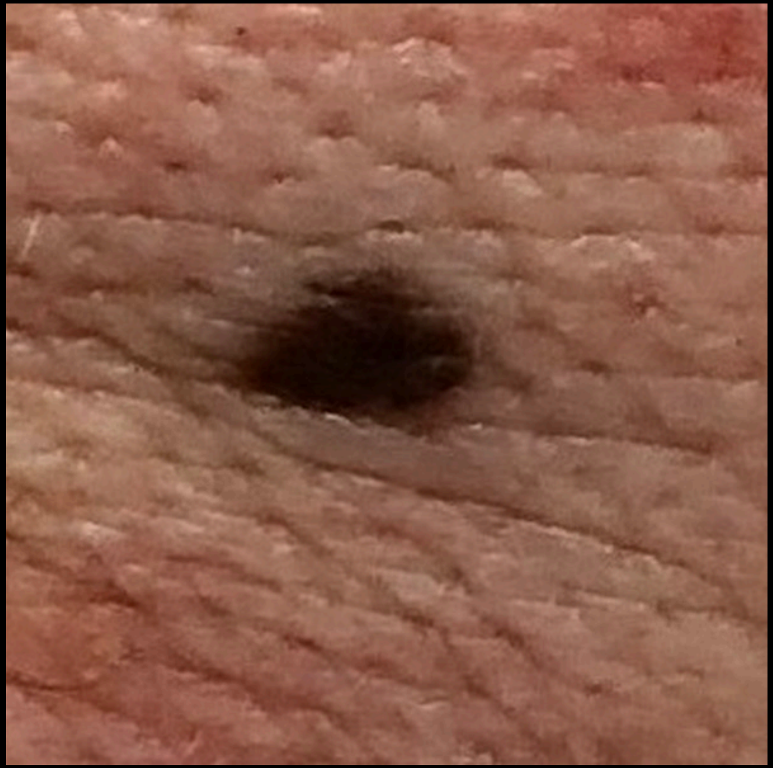

pTis

Case number 93

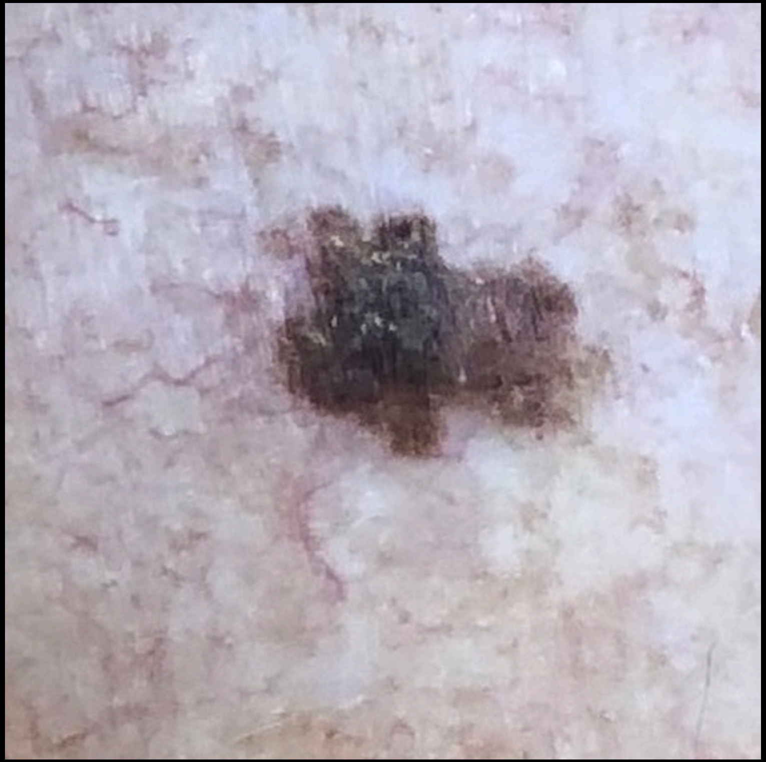

pTis

Case number 94

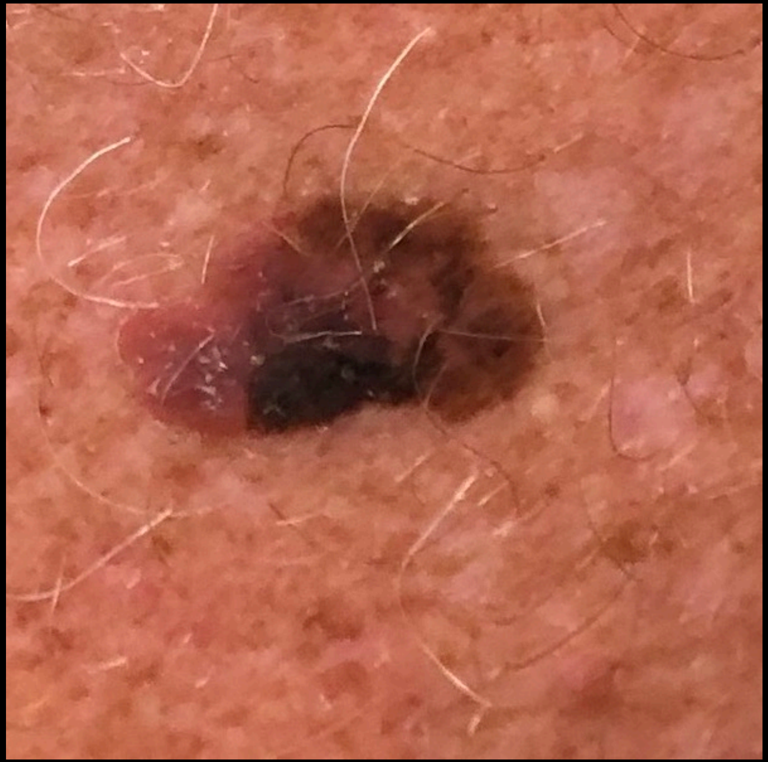

pT1a, 0.5 mm

Case number 95

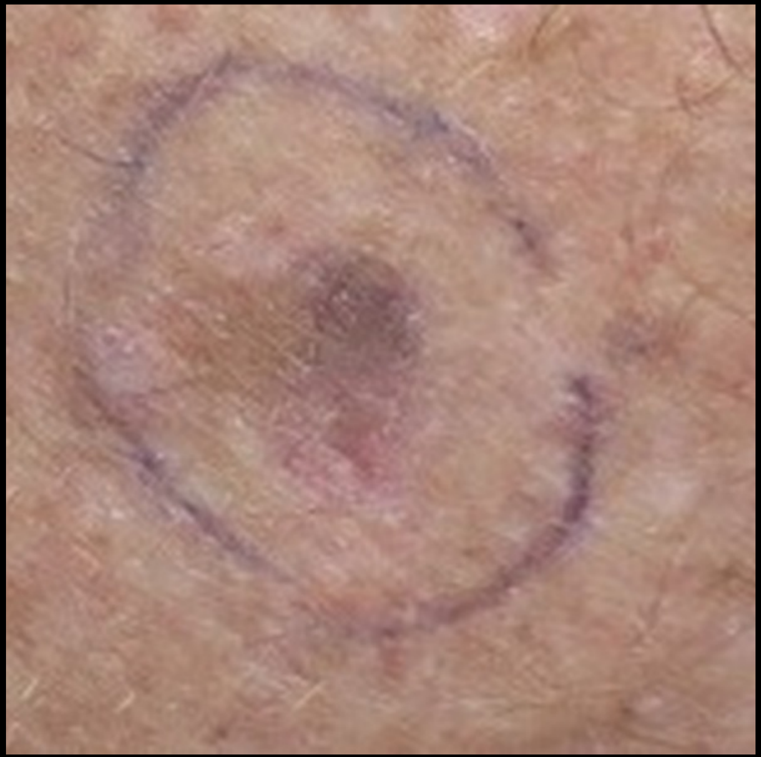

pTis

Case number 96

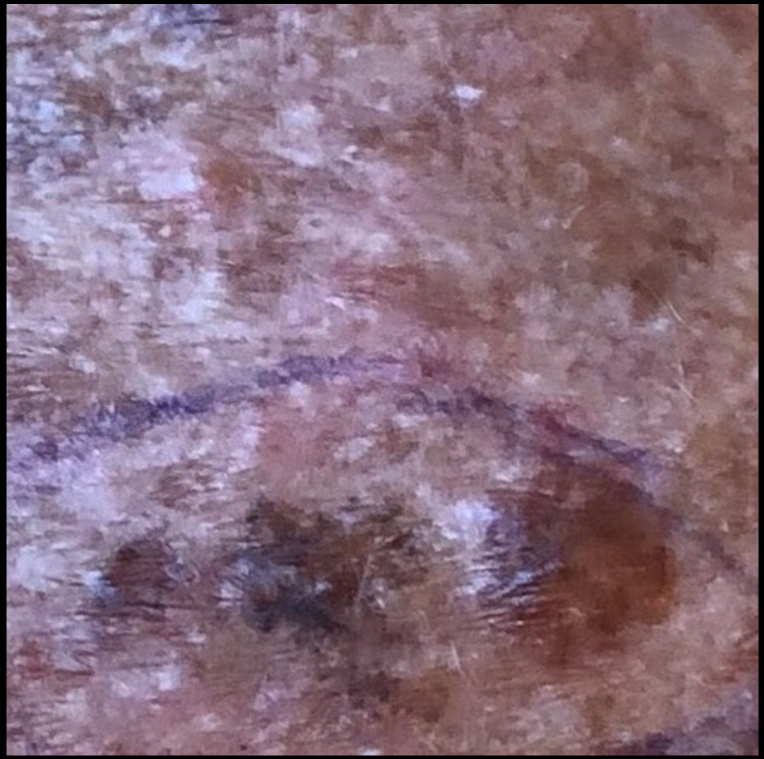

pTis

Case number 97

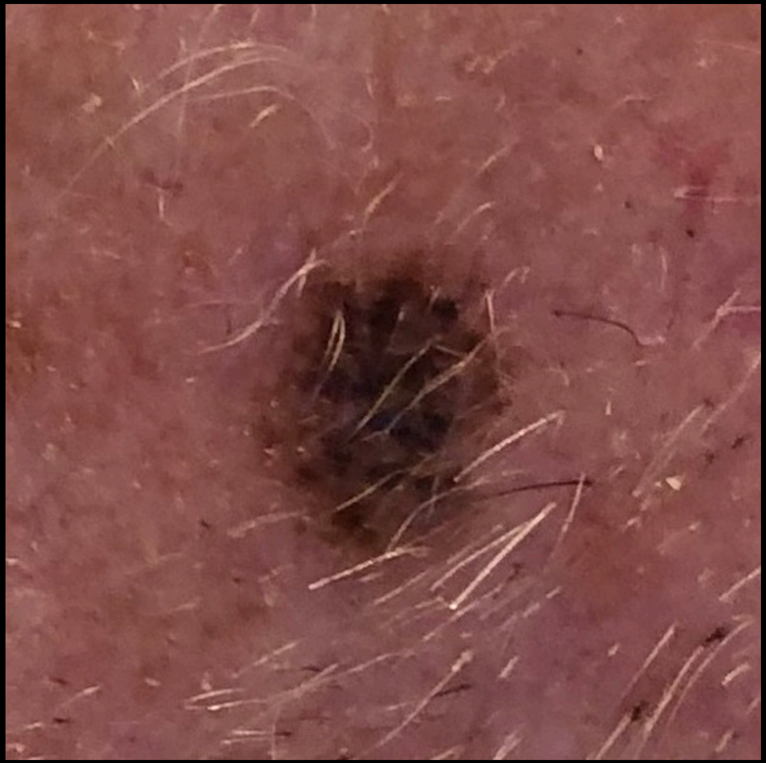

pTis

Case number 98

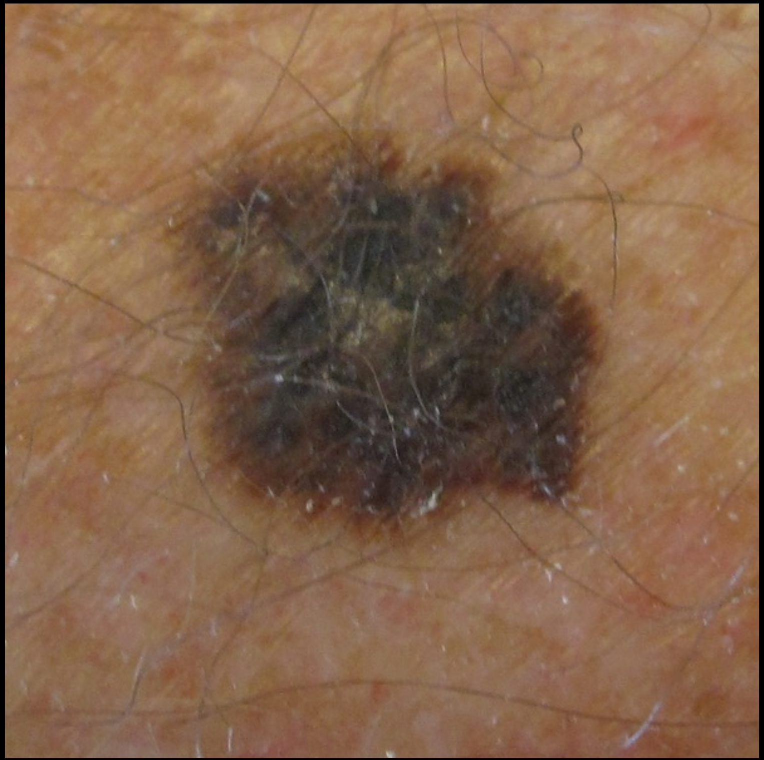

pTis

Case number 99

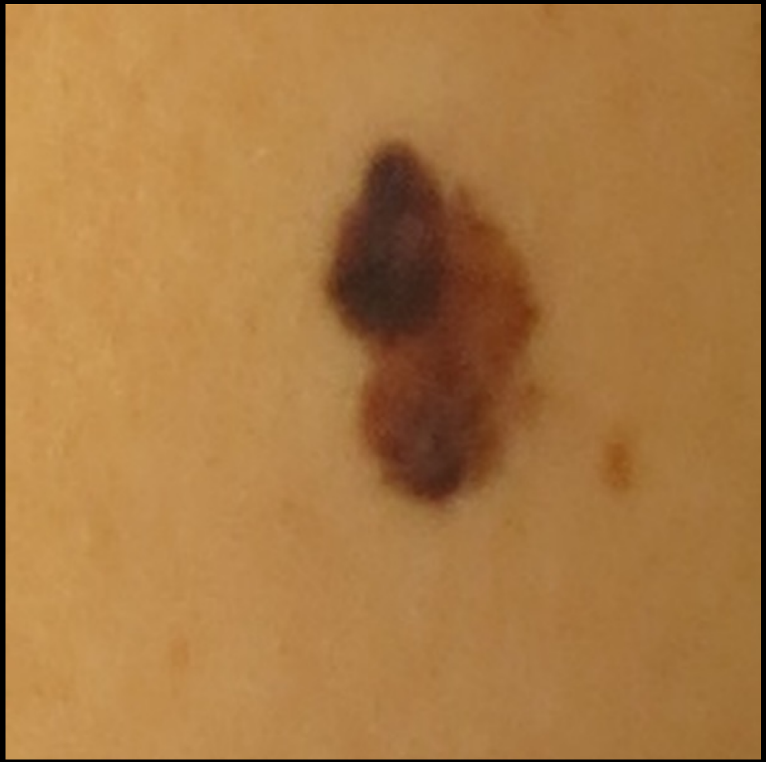

pT1a, 0.6 mm

Case number 100

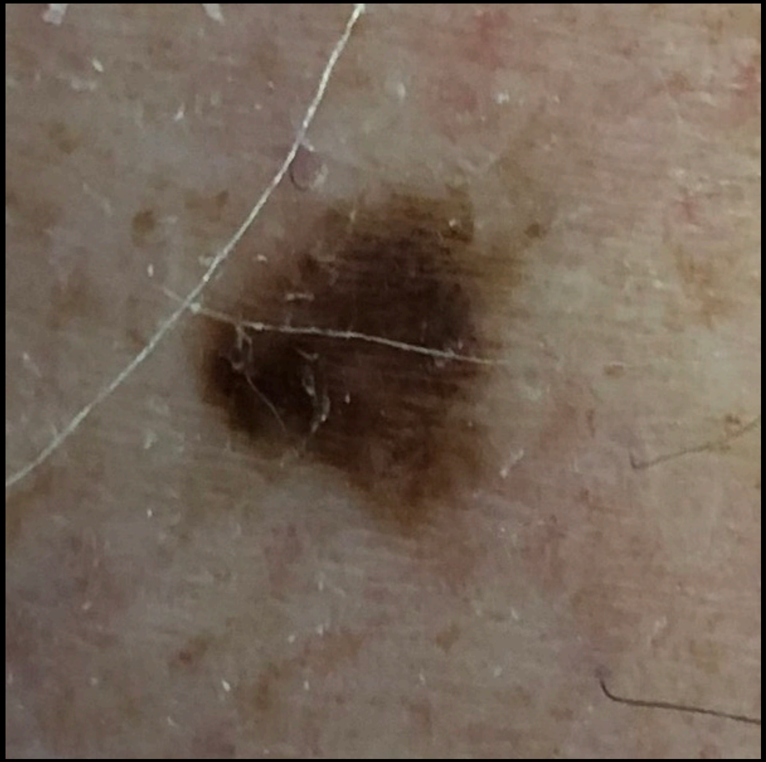

pTis

Case number 101

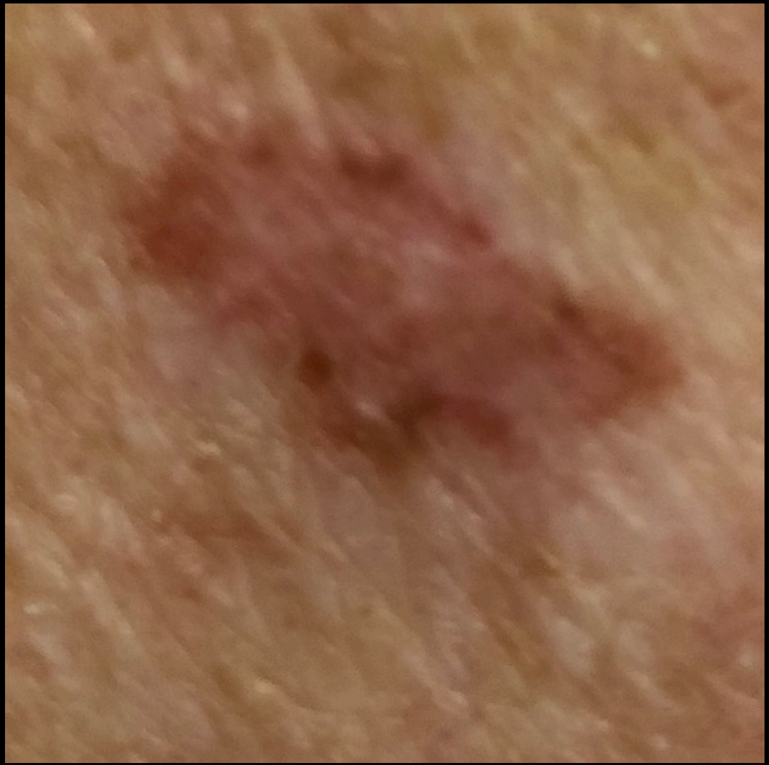

pT1a, 0.3 mm

Case number 102

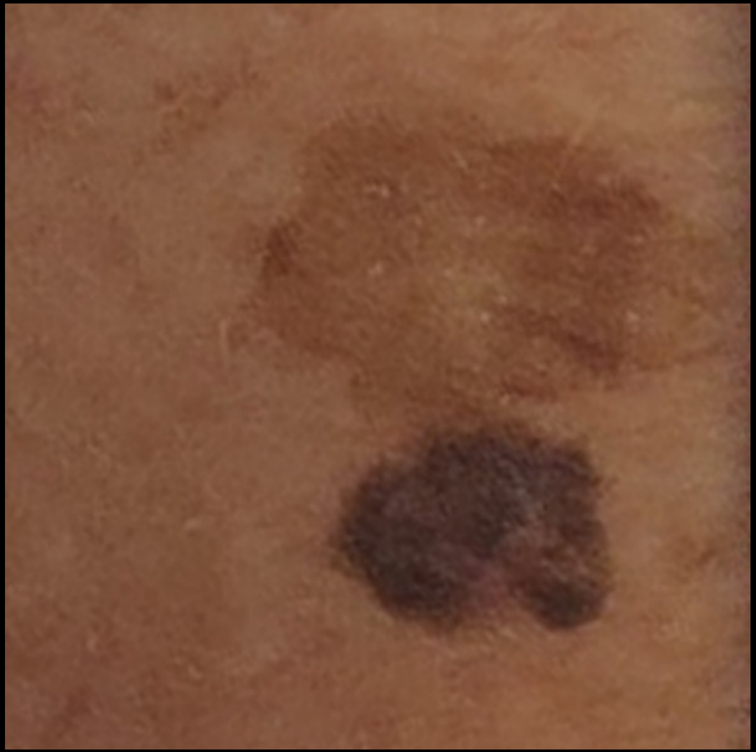

pTis

Case number 103

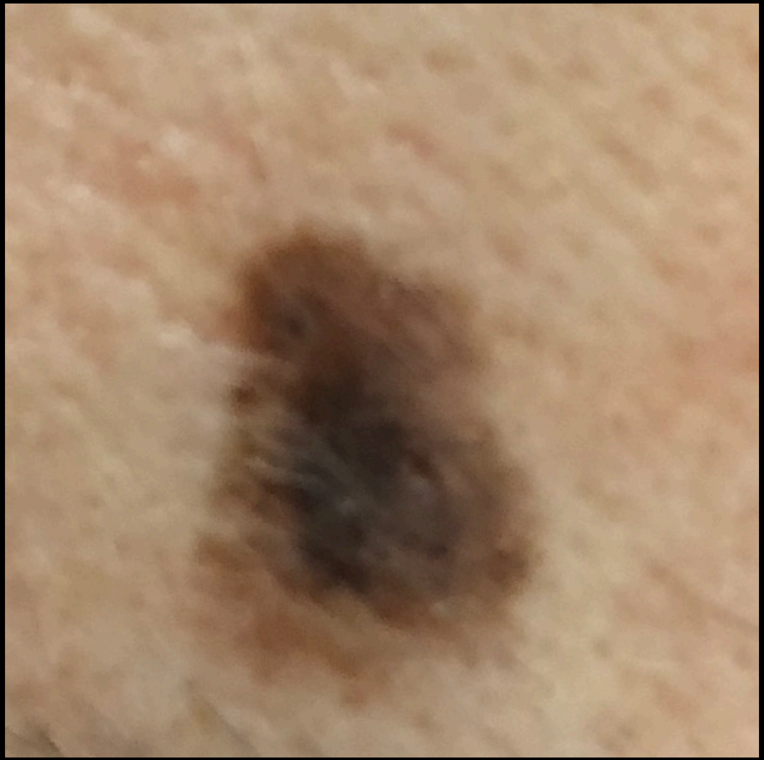

pTis

Case number 104

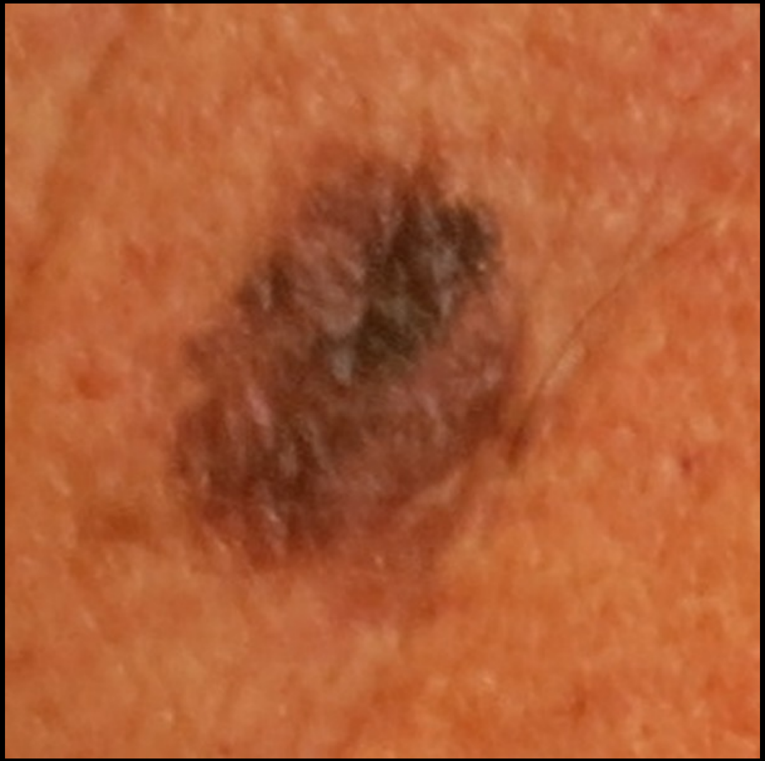

pTis

Case number 105

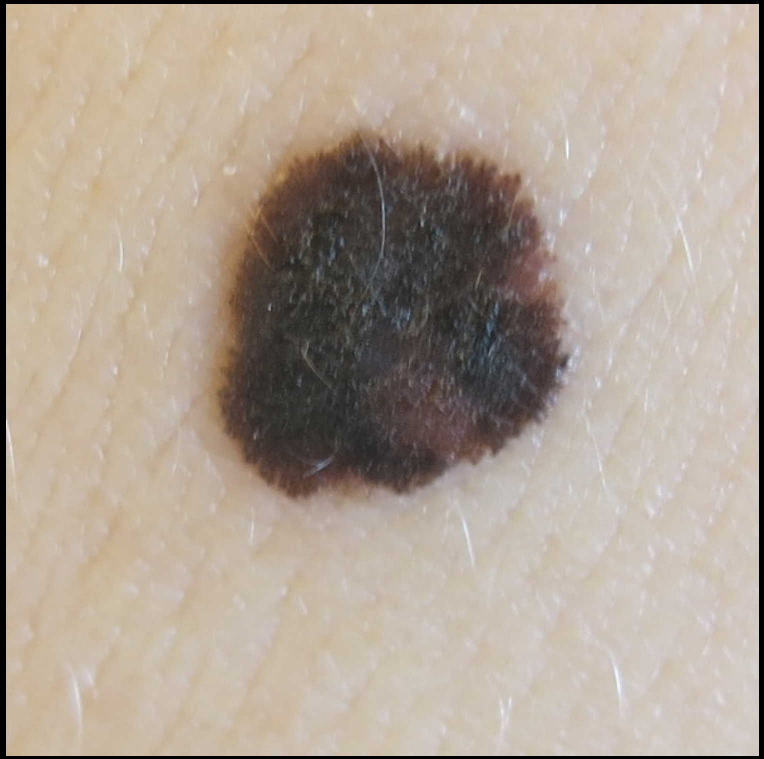

pT1a, 0.7 mm

Case number 106

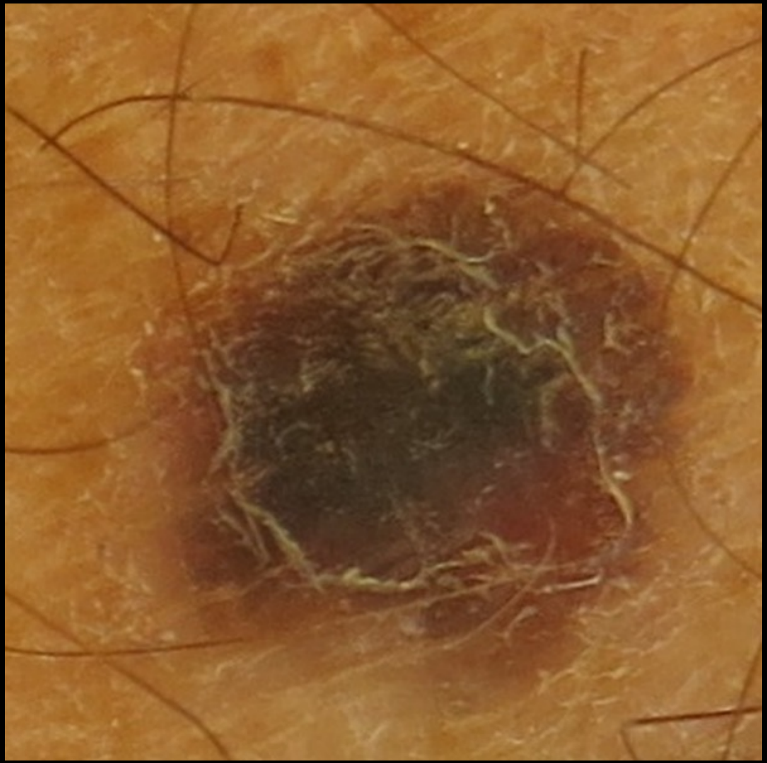

pT1b, 0.8 mm

Case number 107

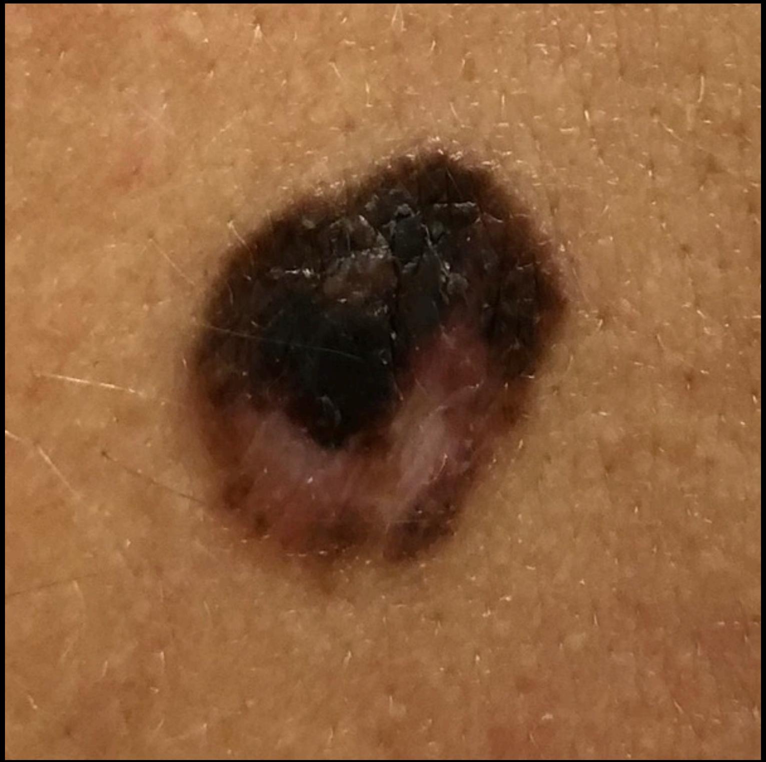

pT2a, 1.3 mm

Case number 108

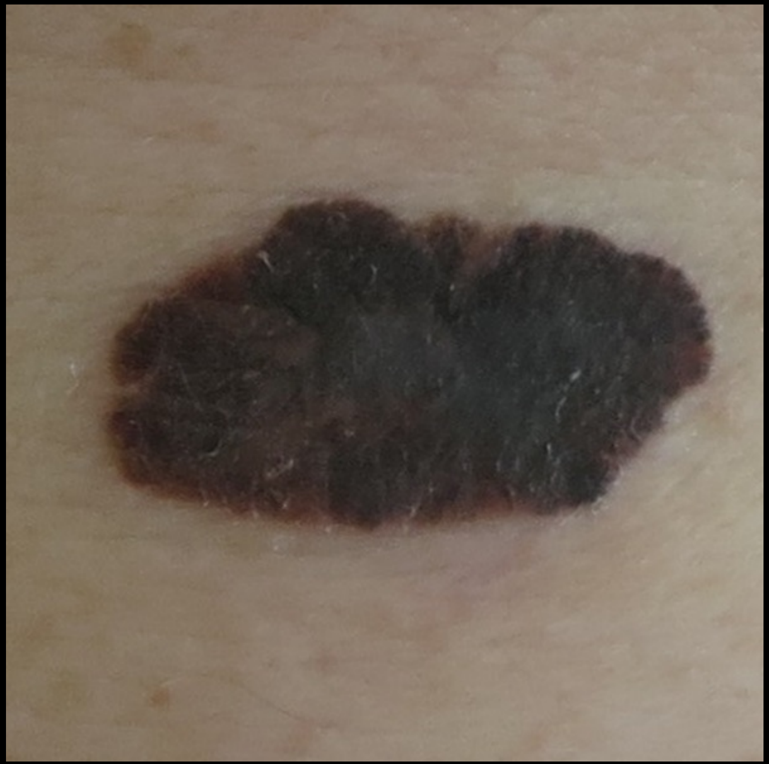

pT1b, 0.9 mm

Case number 109

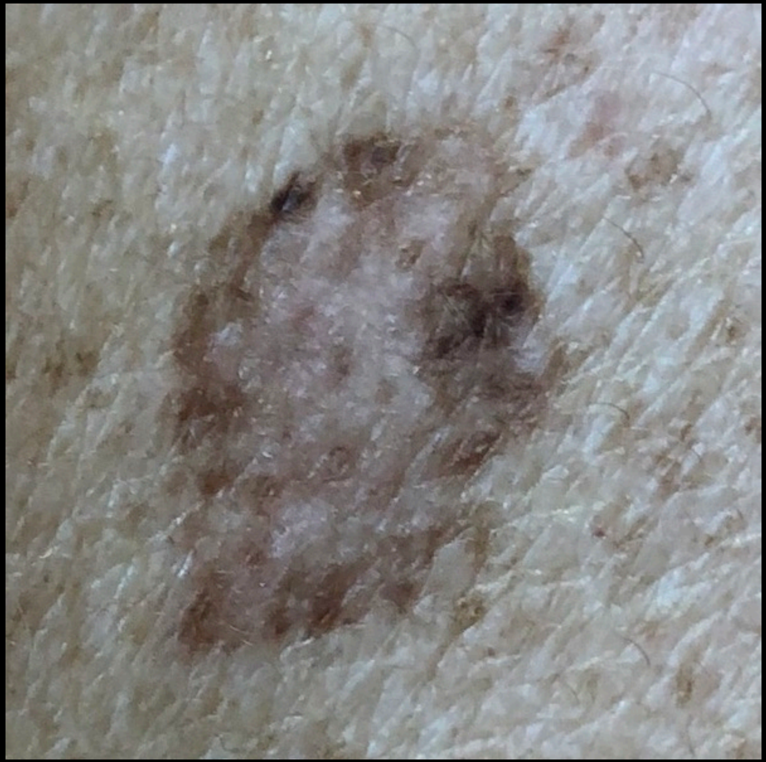

pTis

Case number 110

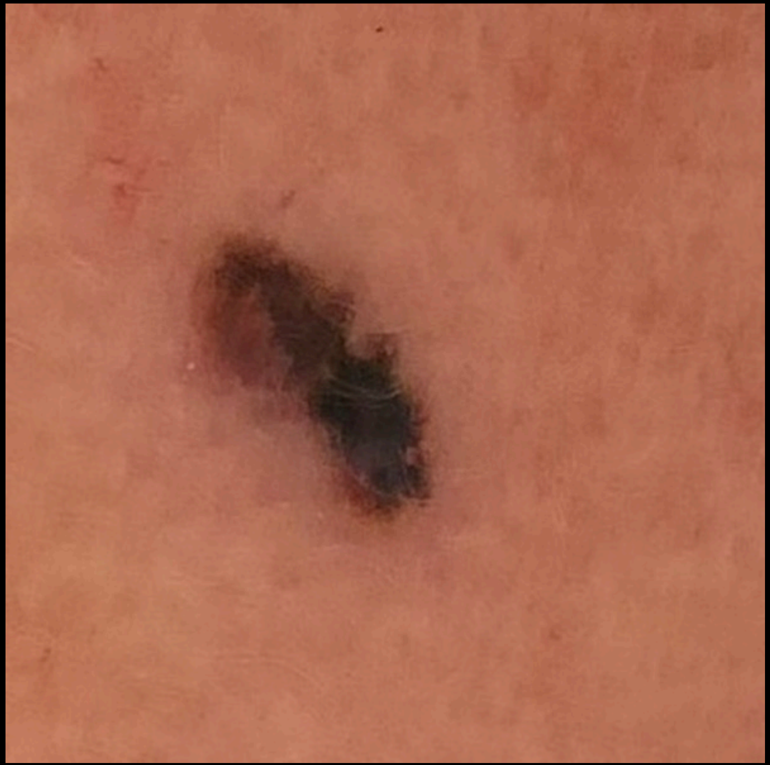

pT1a, 0.4 mm

Case number 111

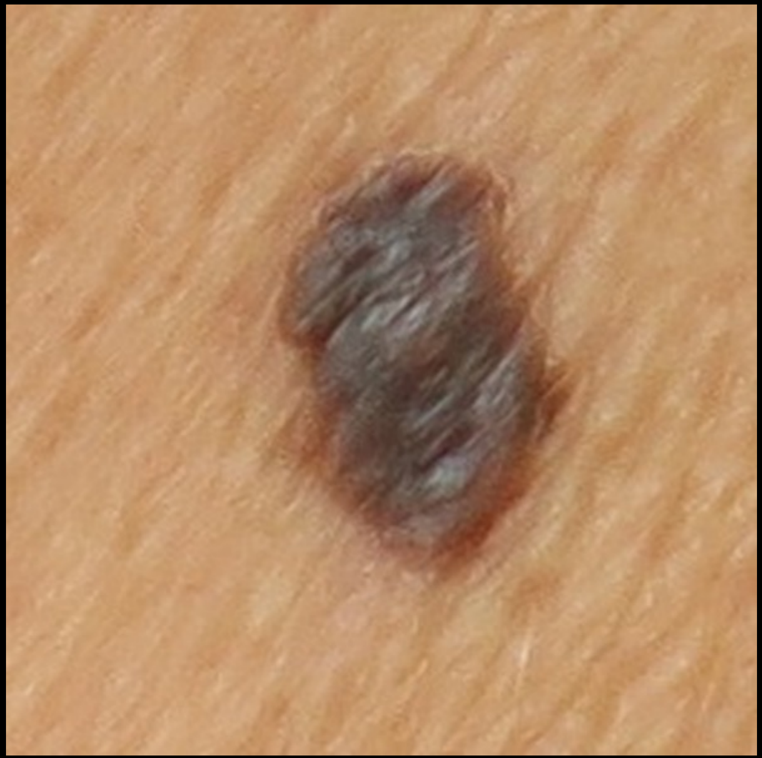

pT2a, 1.1 mm

Case number 112

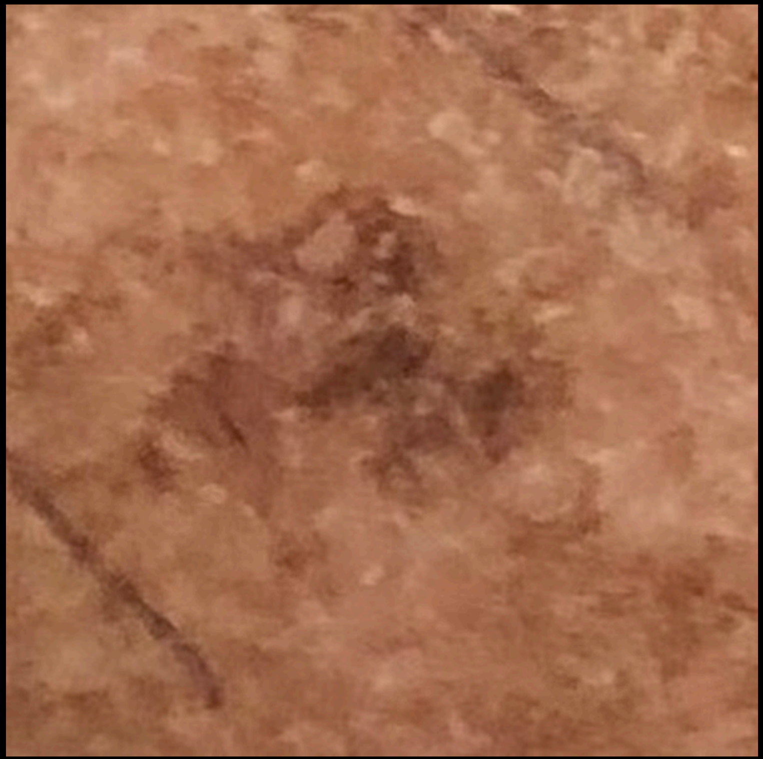

pTis

Case number 113

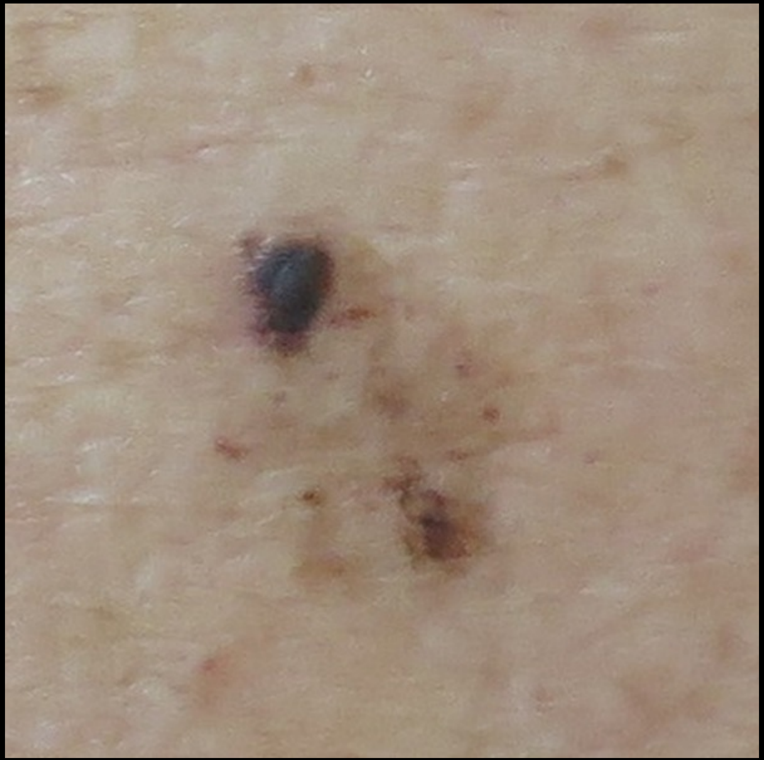

pT2a, 1.3 mm

Case number 114

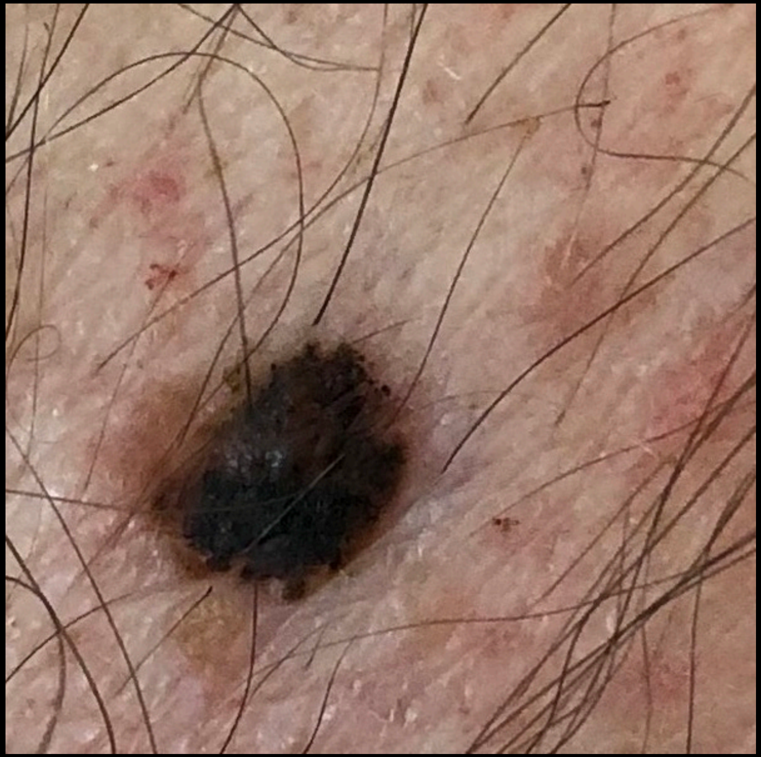

pT1a, 0.7 mm

Case number 115

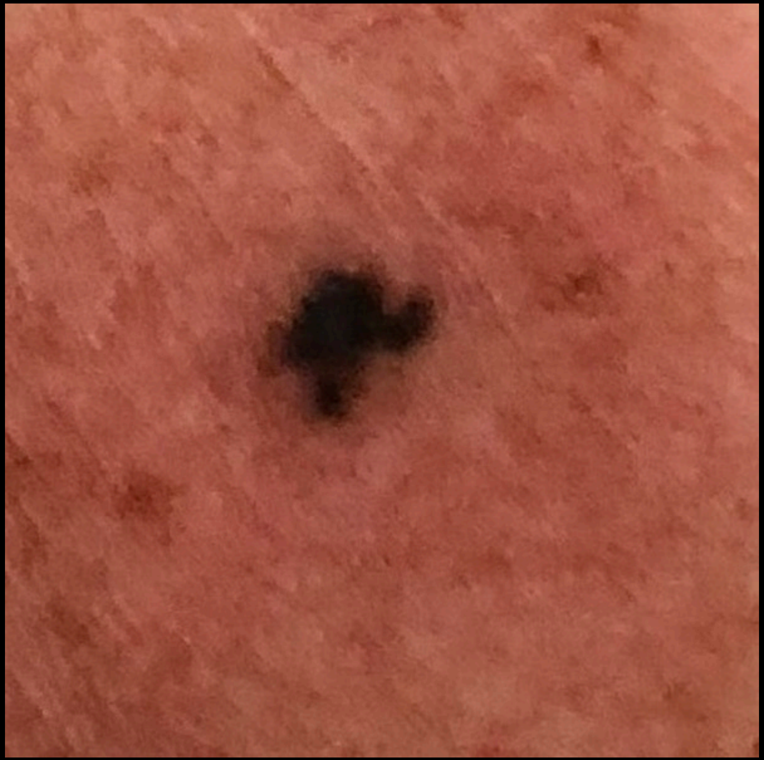

pT1a, 0.5 mm

Case number 116

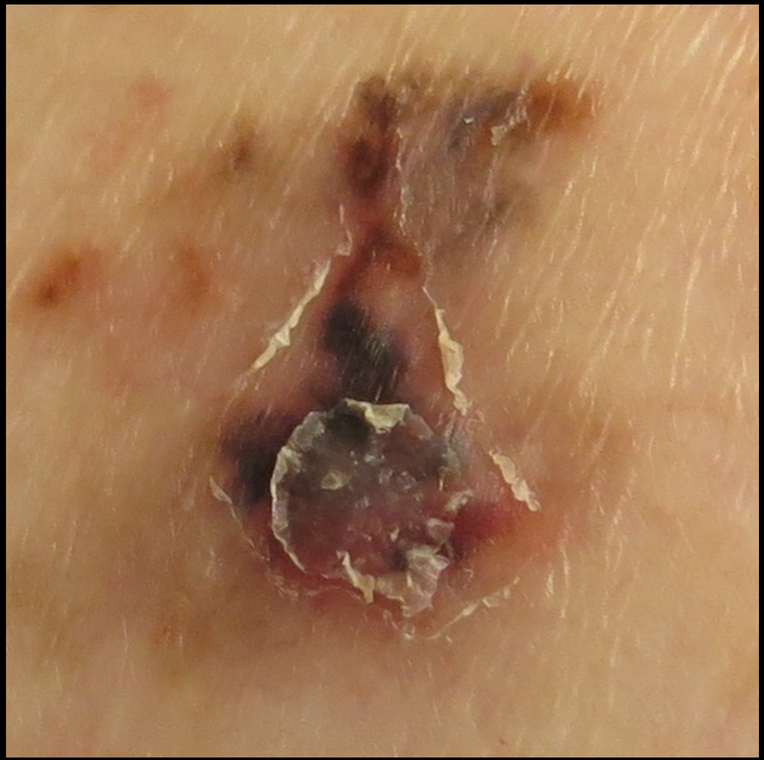

pT2a, 1.1 mm

Case number 117

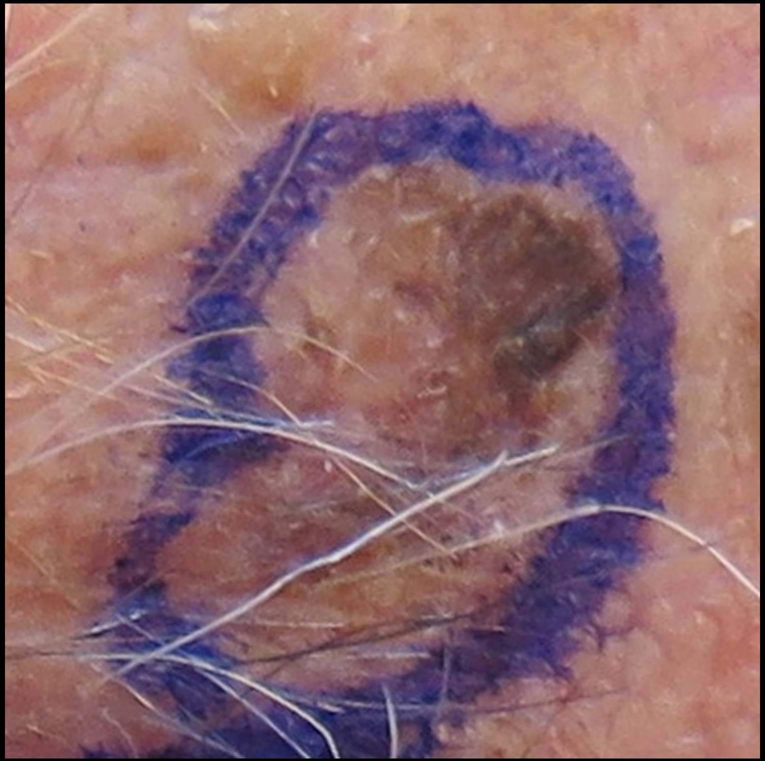

pTis

Case number 118

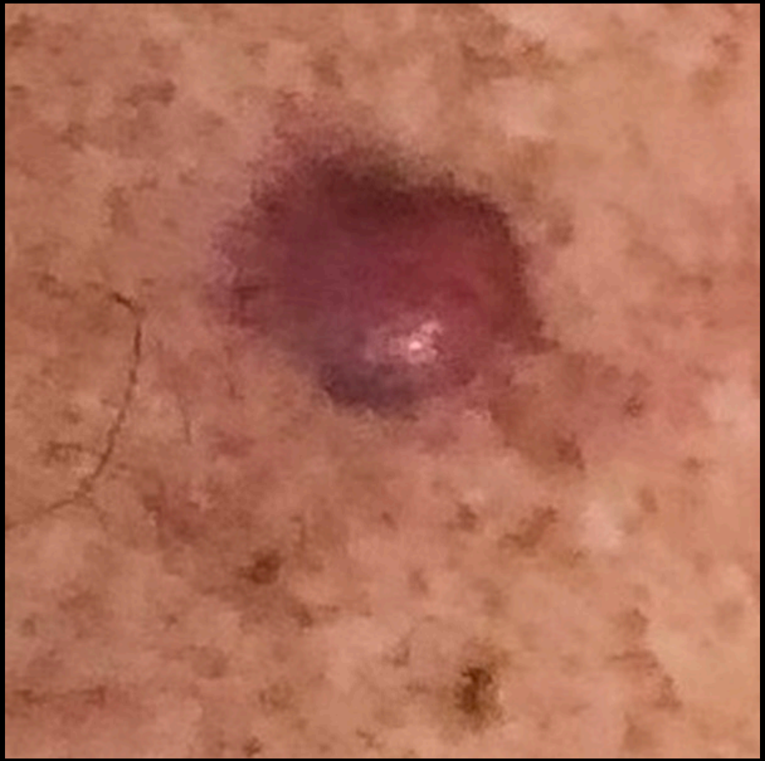

pT4a, 9.0 mm

Case number 119

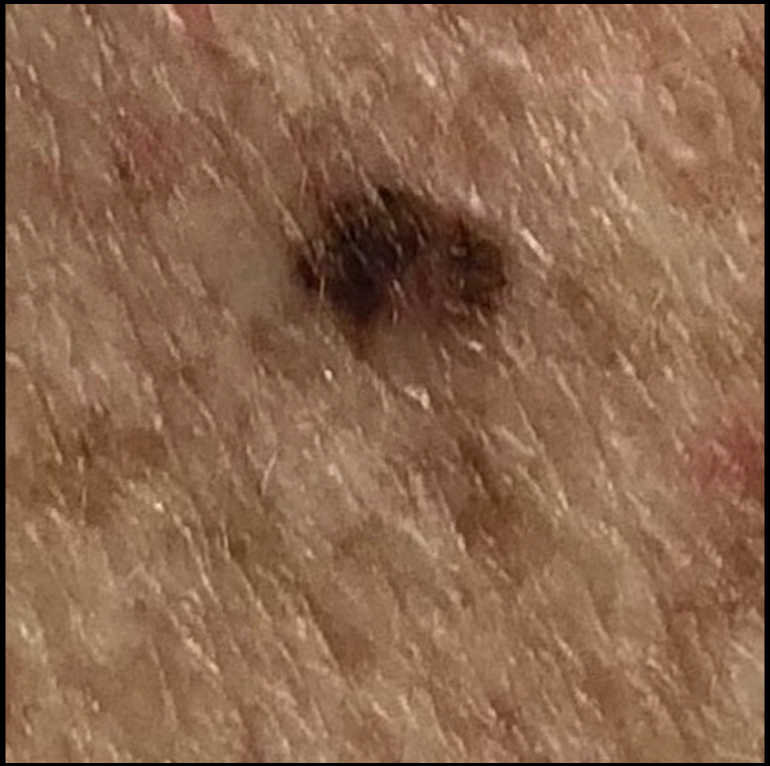

pTis

Case number 120

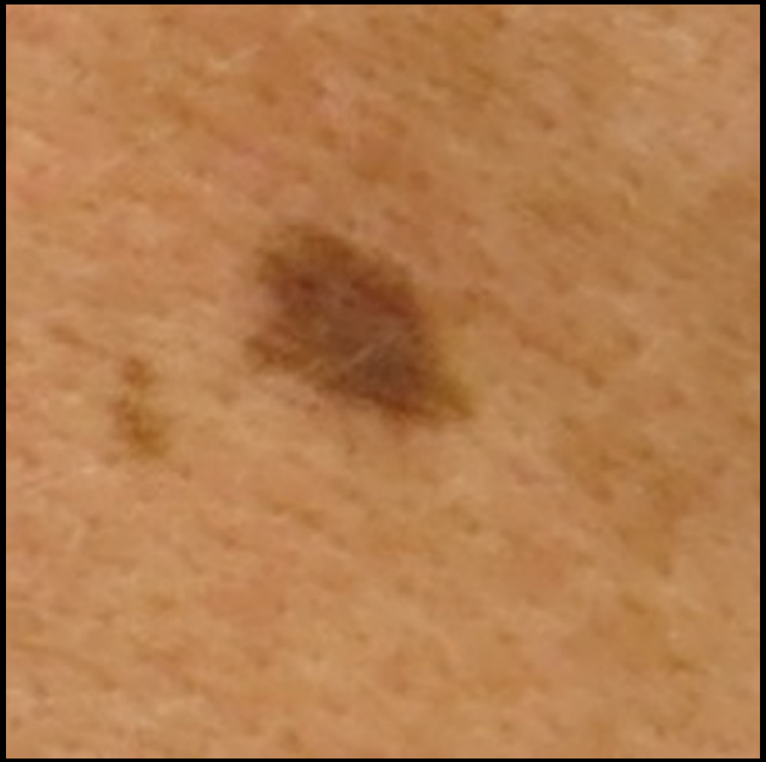

pTis

Case number 121

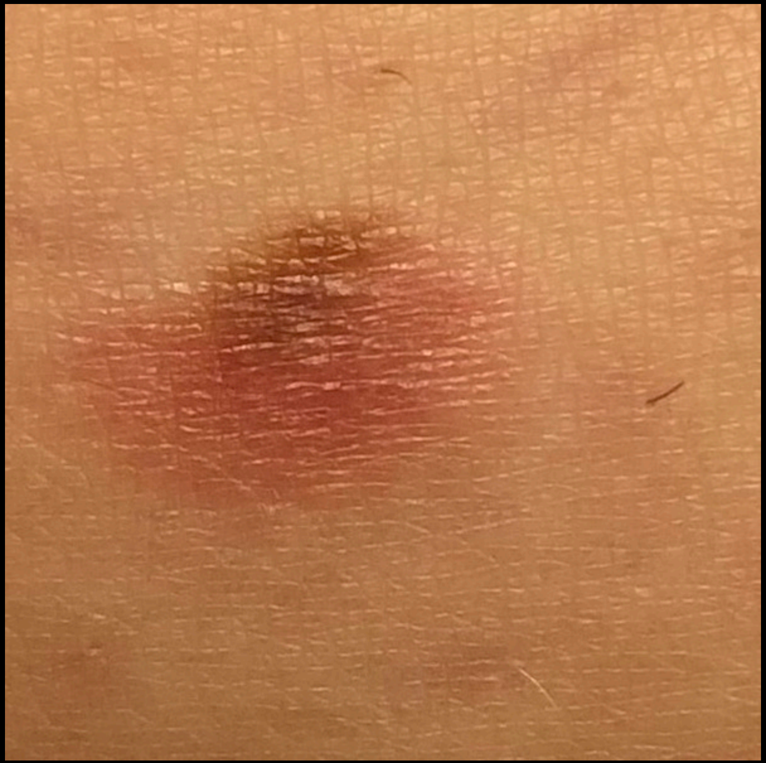

pTis

Case number 122

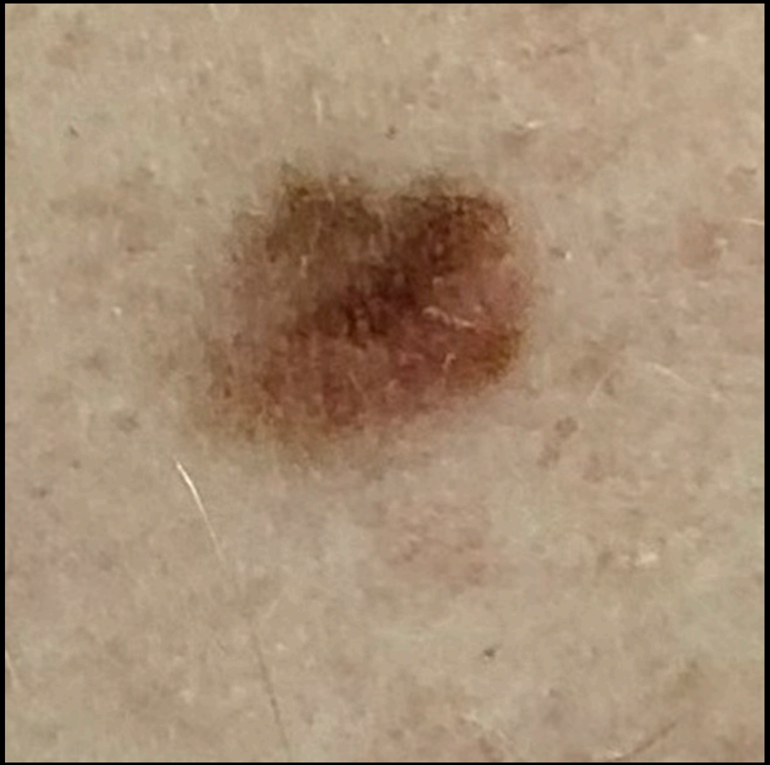

pTis

Case number 123

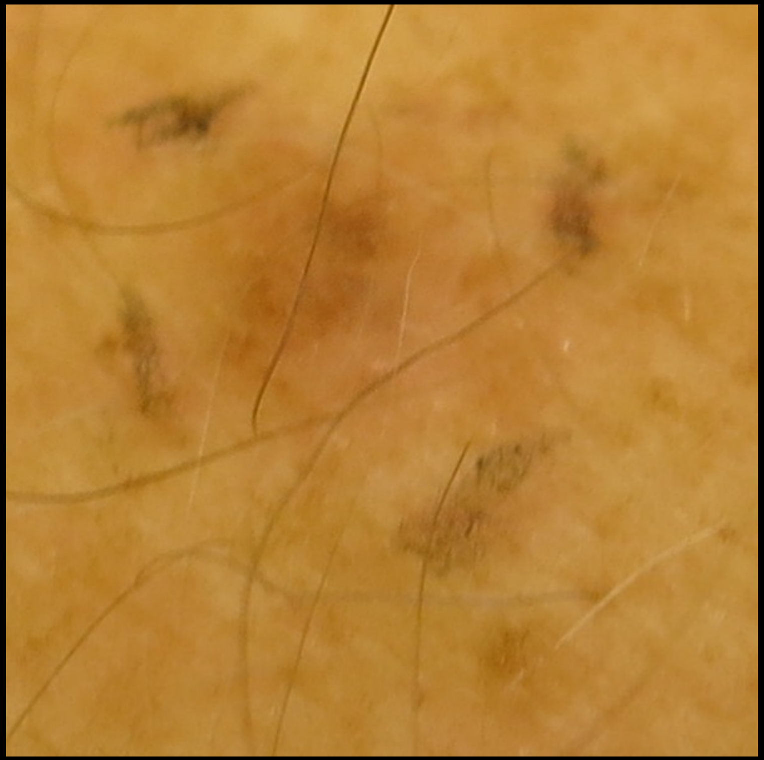

pTis

Case number 124

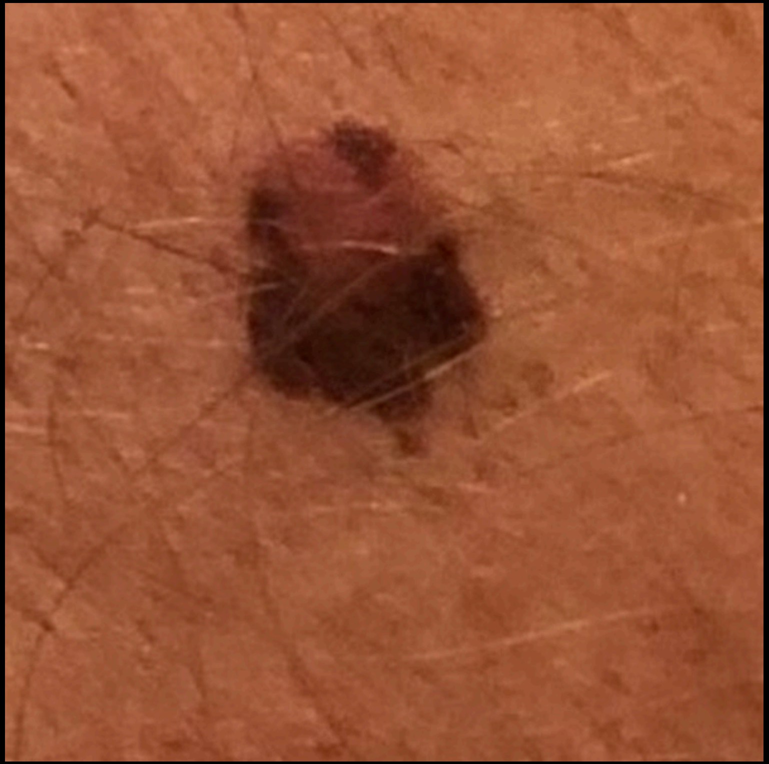

pTis

Case number 125

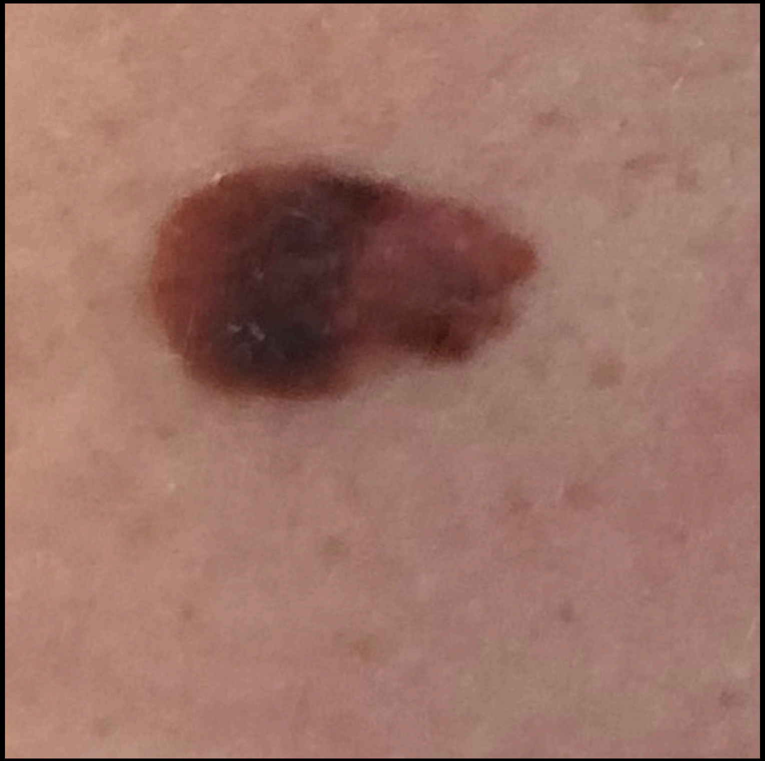

pT1a, 0.6 mm

Case number 126

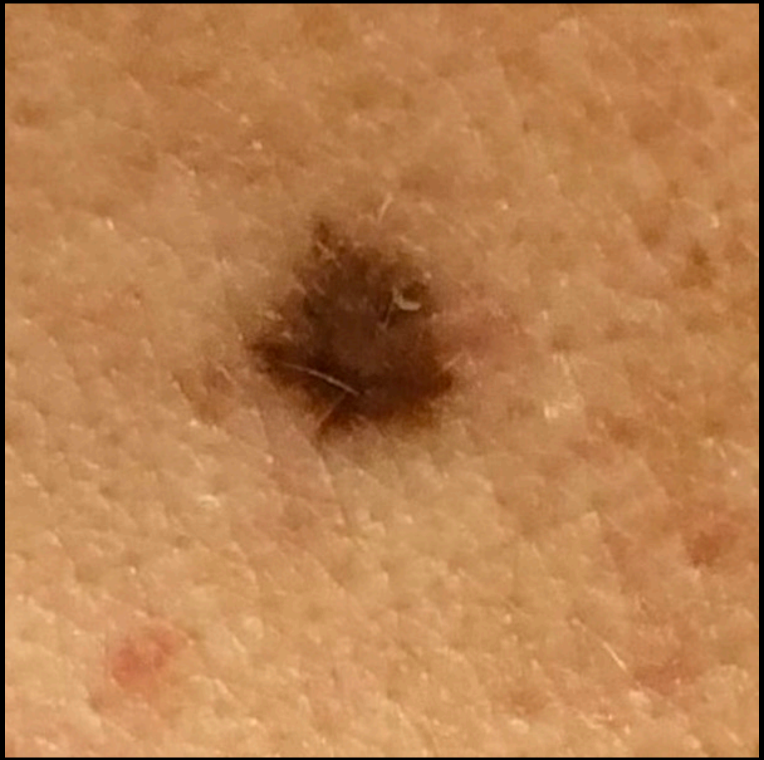

pT1a, 0.5 mm

Case number 127

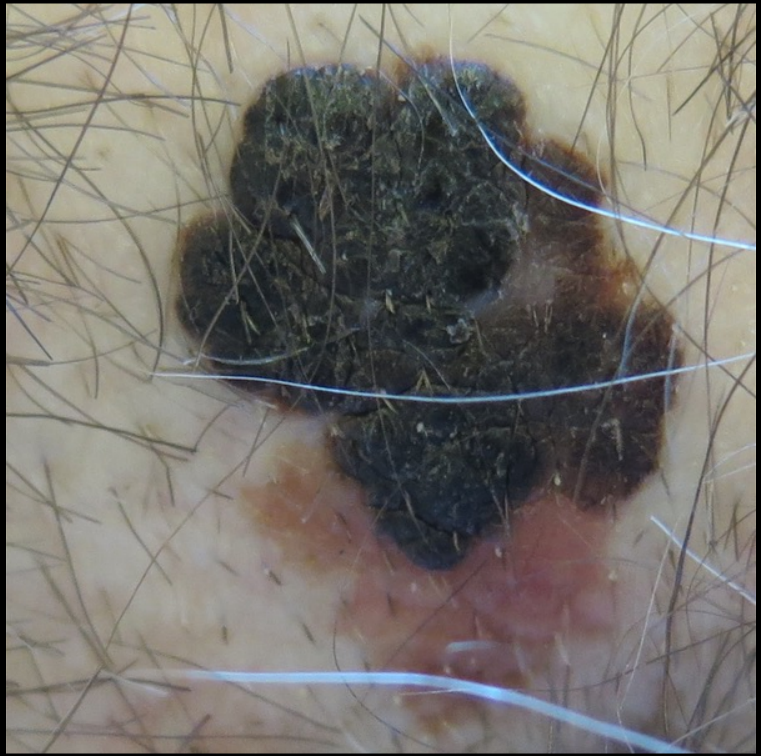

pT1a, 0.8 mm

Case number 128

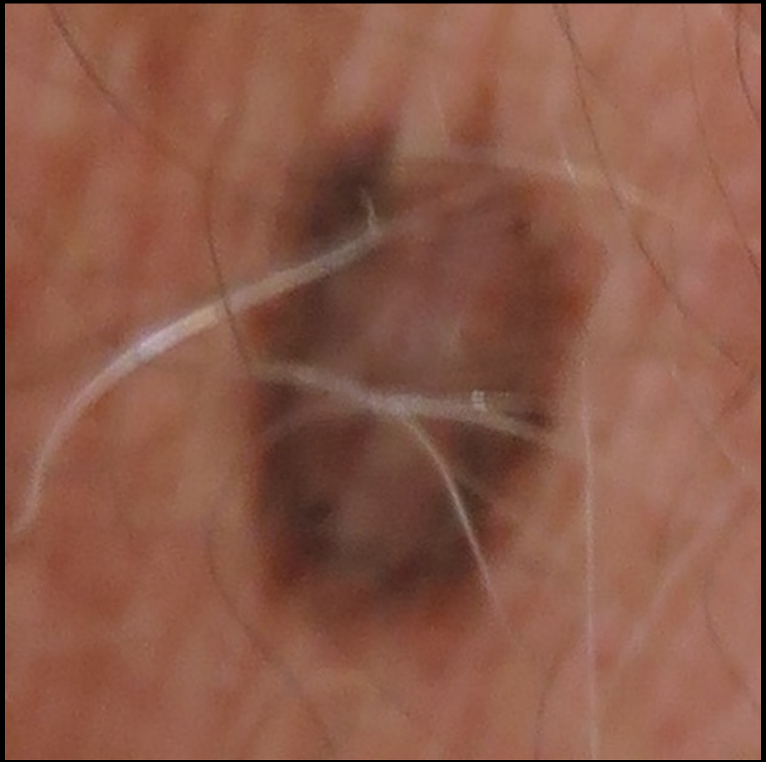

pTis

Case number 129

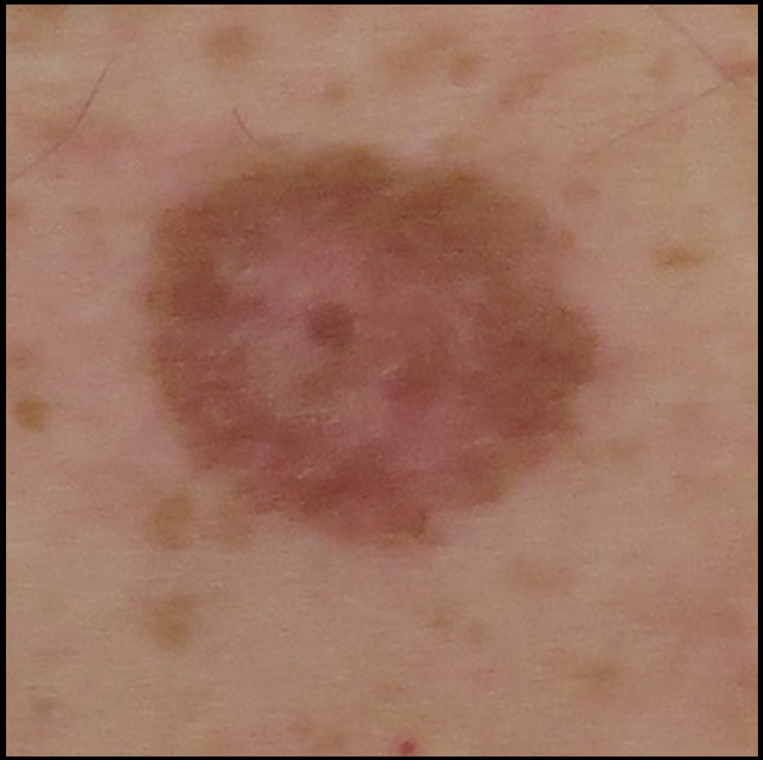

pT1a, 0.3 mm

Case number 130

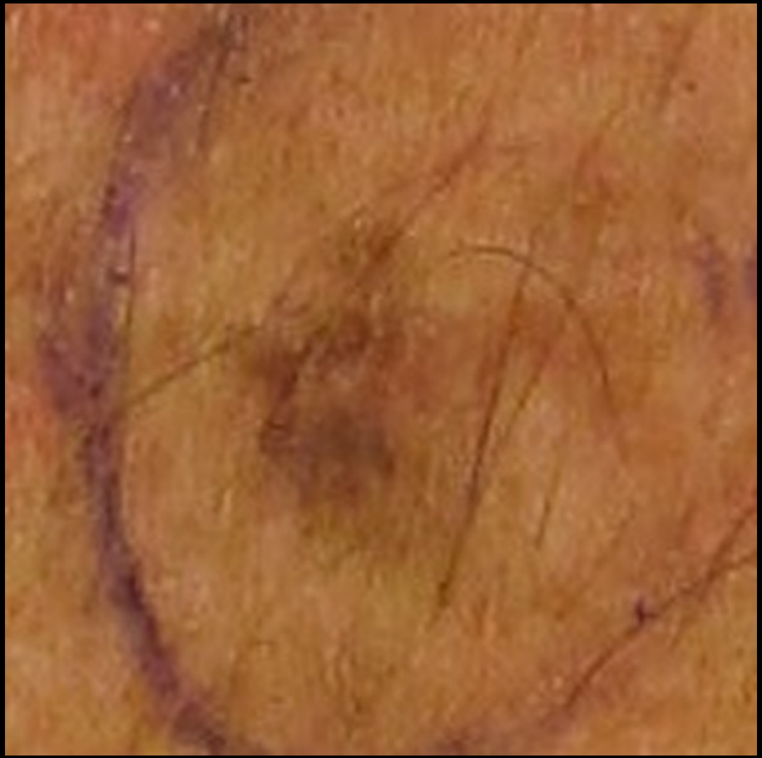

pTis

Case number 131

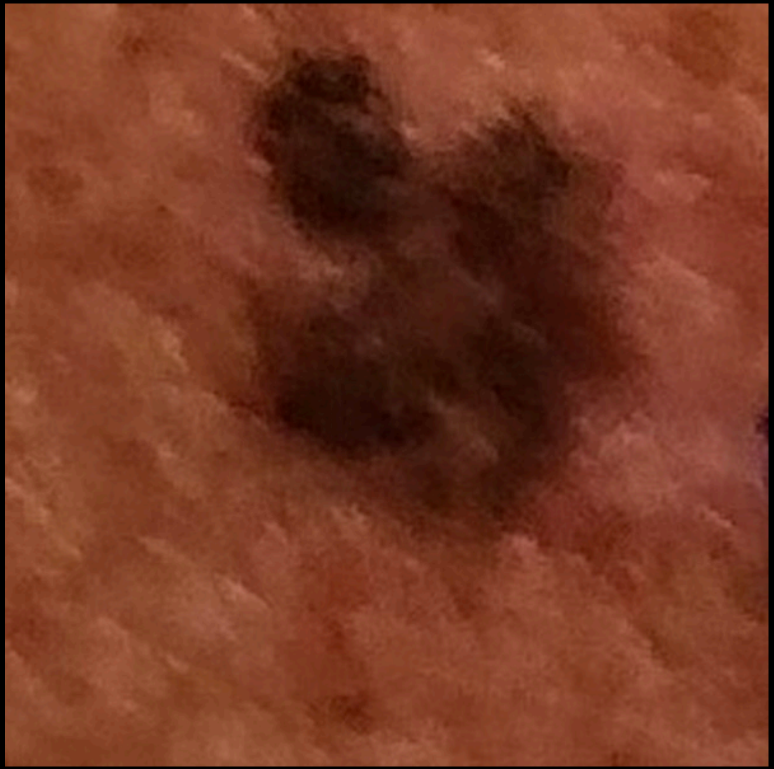

pTis

Case number 132

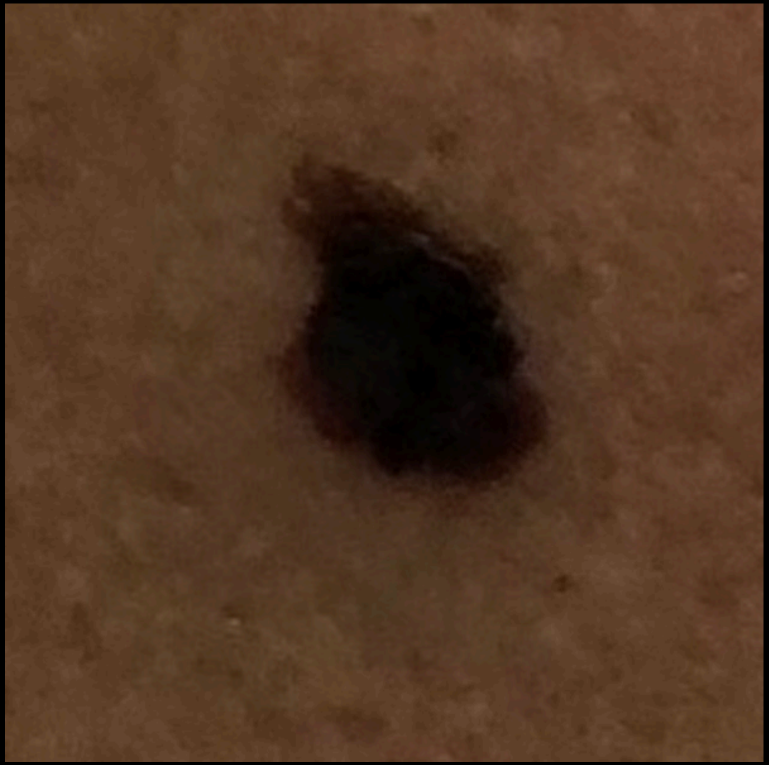

pT2a, 1.3 mm

Case number 133

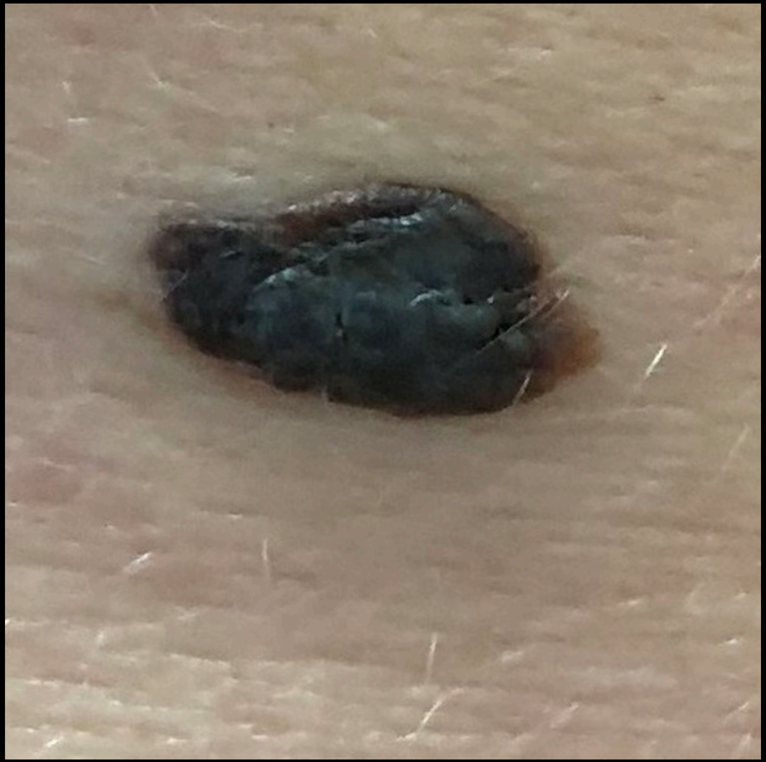

pT2a, 1.1 mm

Case number 134

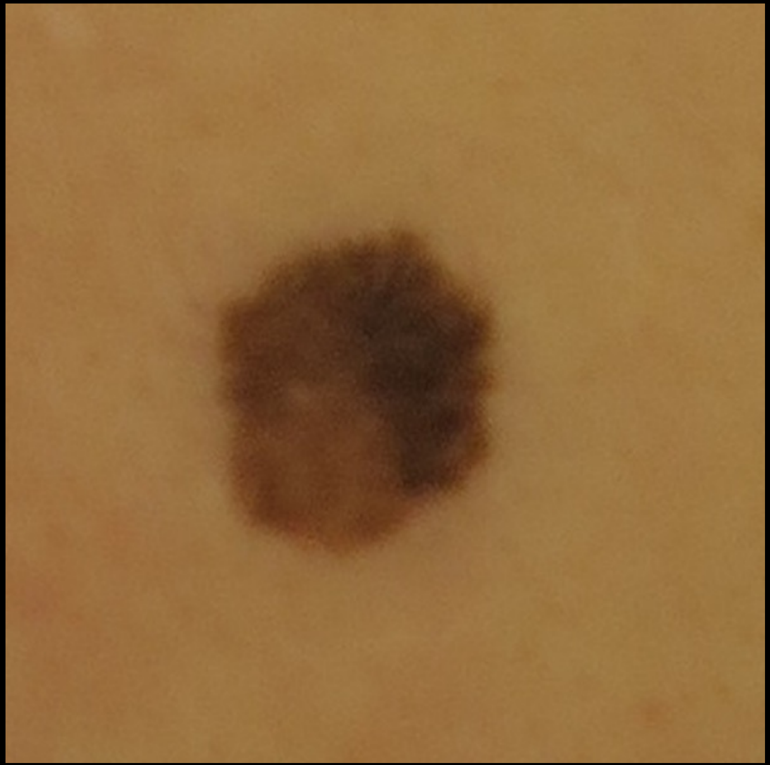

pTis

Case number 135

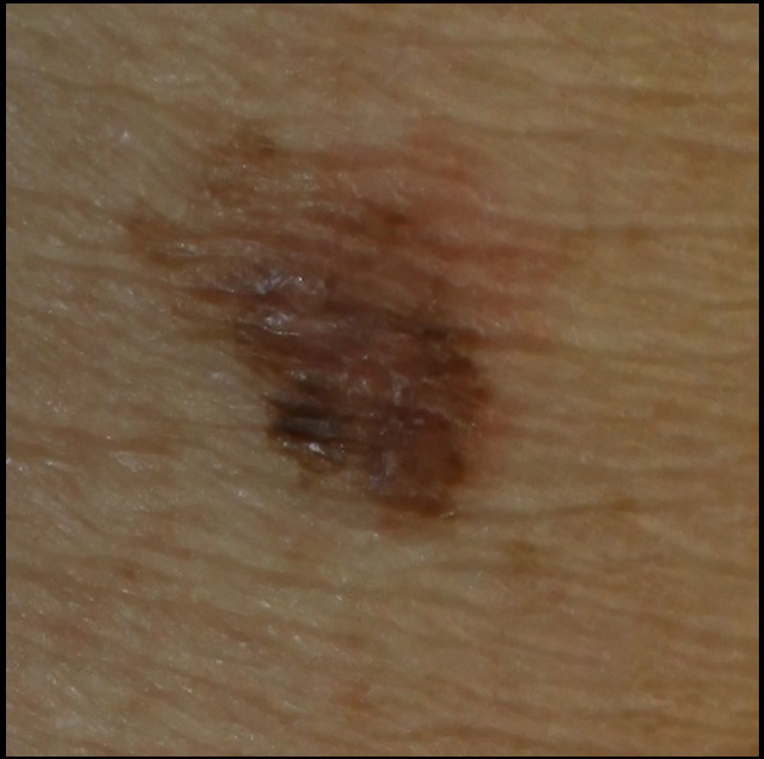

pT1a, 0.9 mm

Case number 136

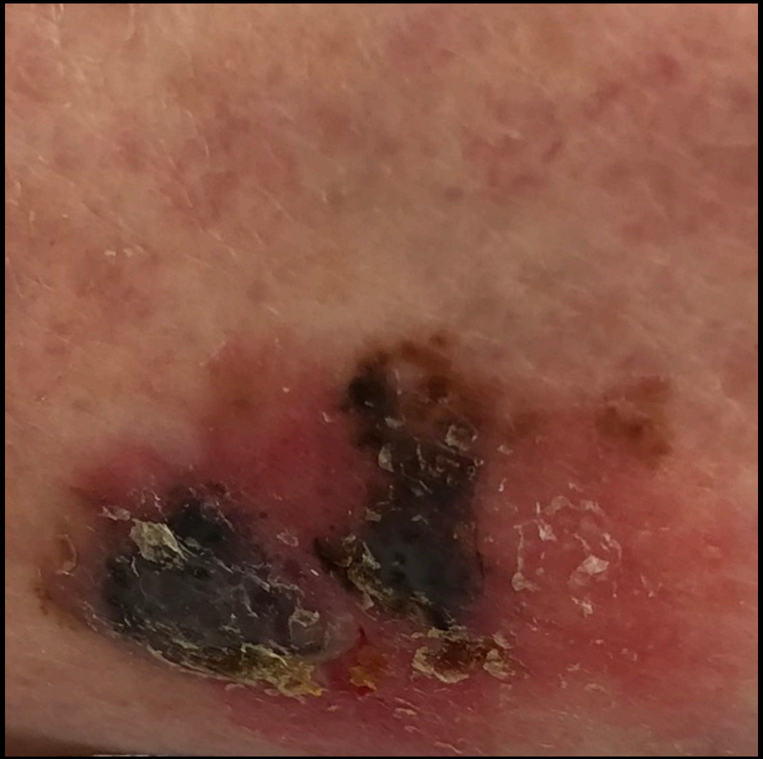

pT3b, 3.9 mm

Case number 137

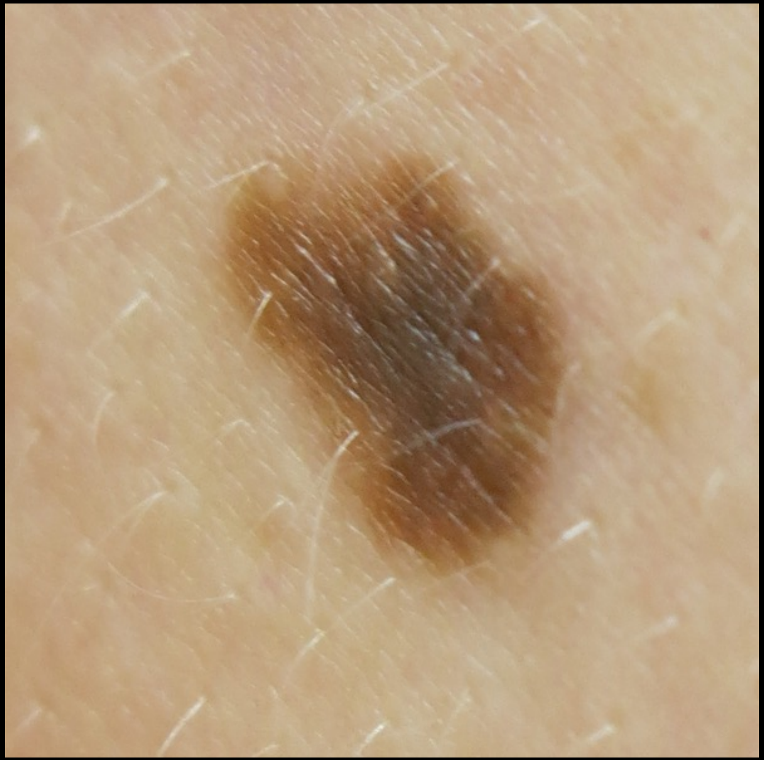

pT1a, 0.4 mm

Case number 138

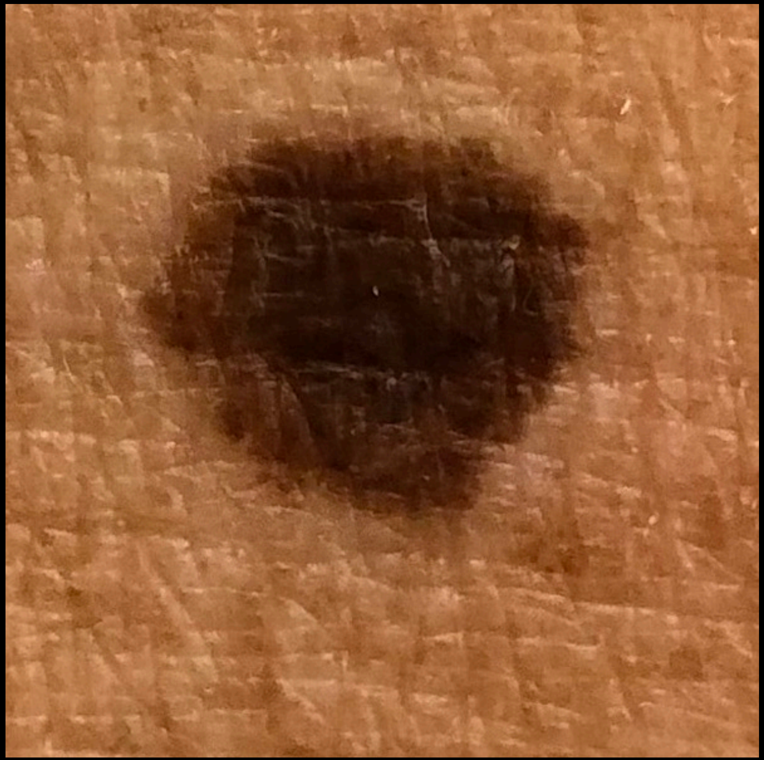

pTis

Case number 139

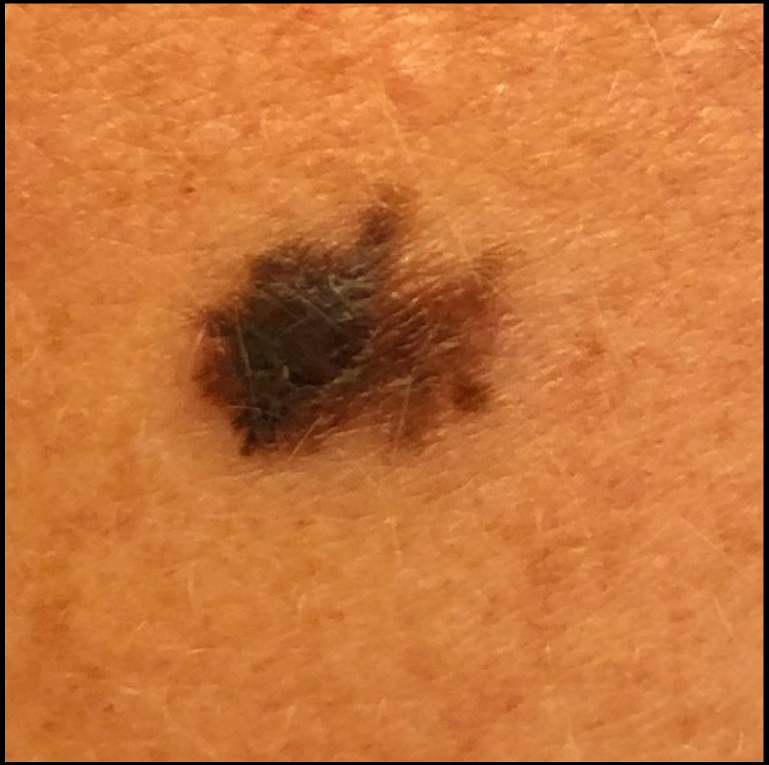

pT1b, 0.8 mm

Case number 140

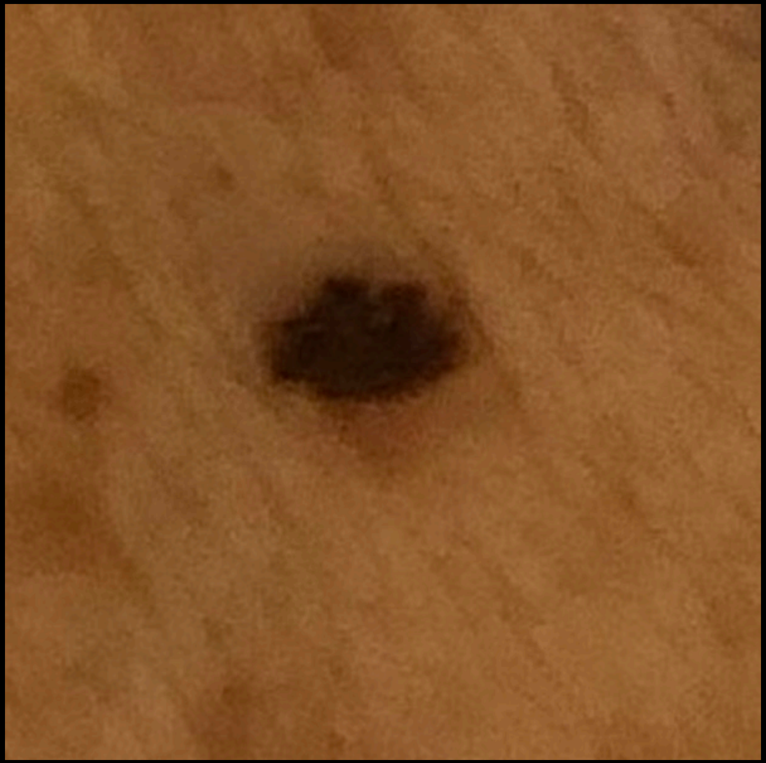

pTis

Case number 141

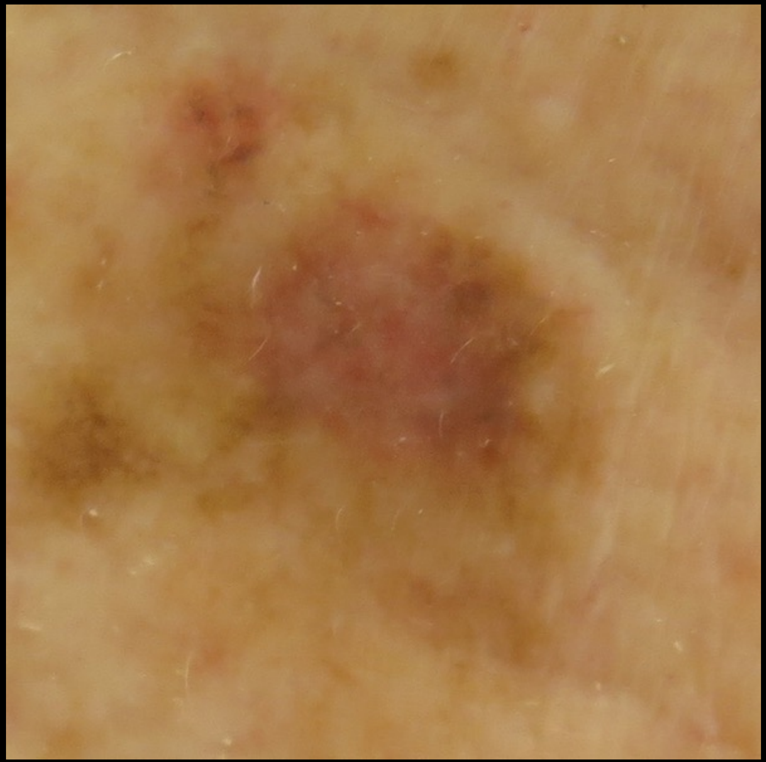

pT1a, 0.5 mm

Case number 142

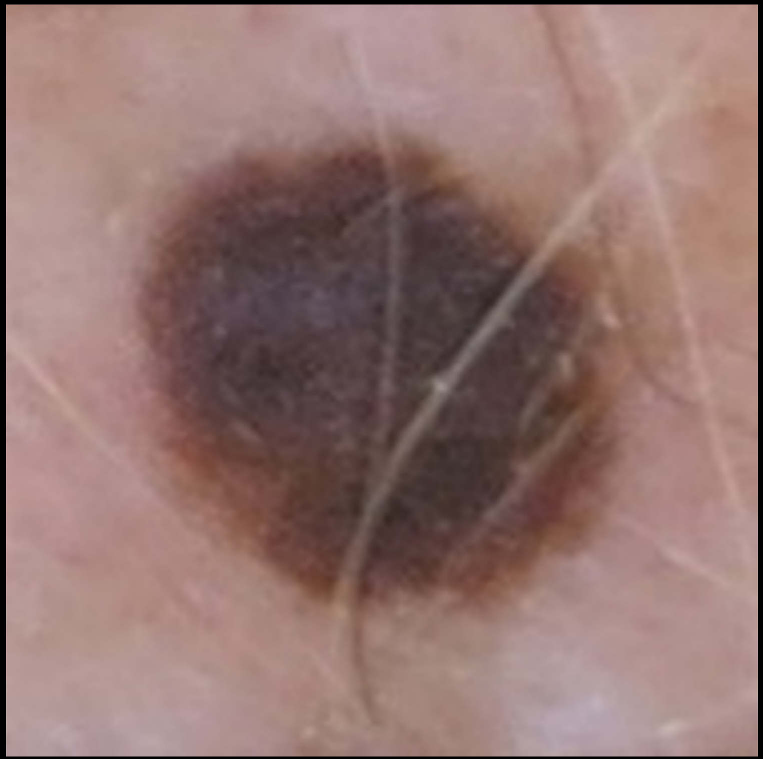

pTis

Case number 143

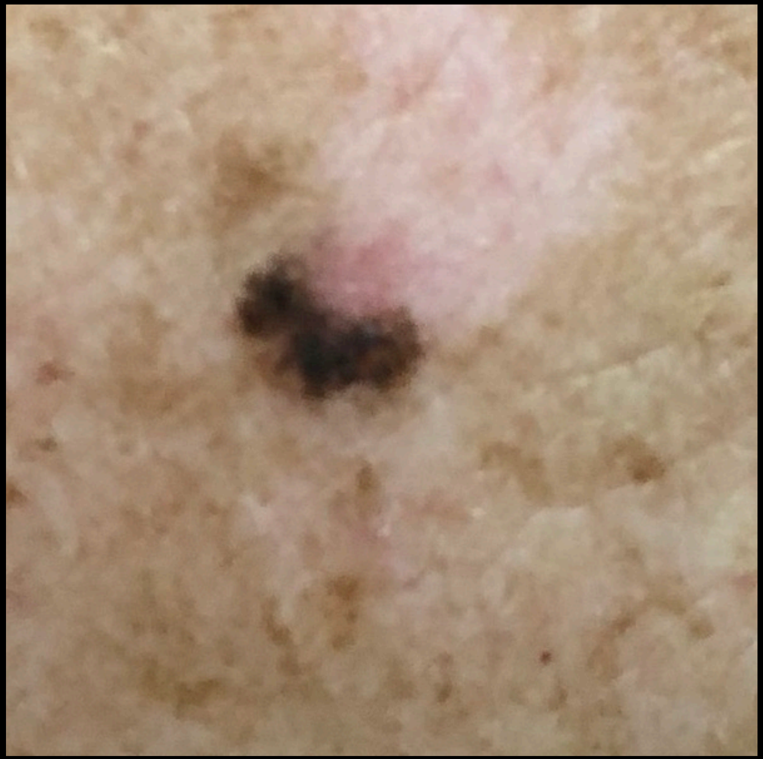

pTis

Case number 144

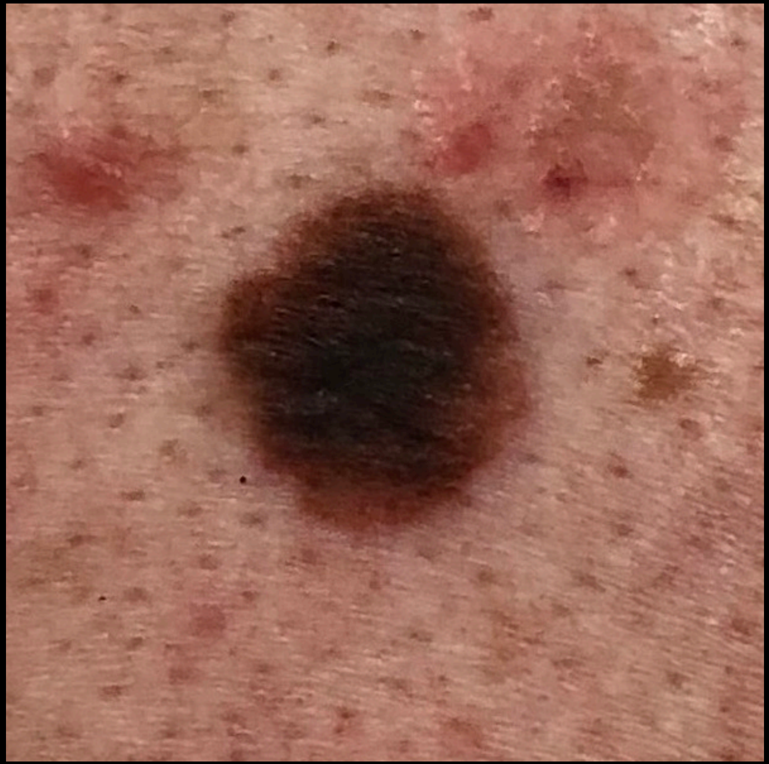

pTis

Case number 145

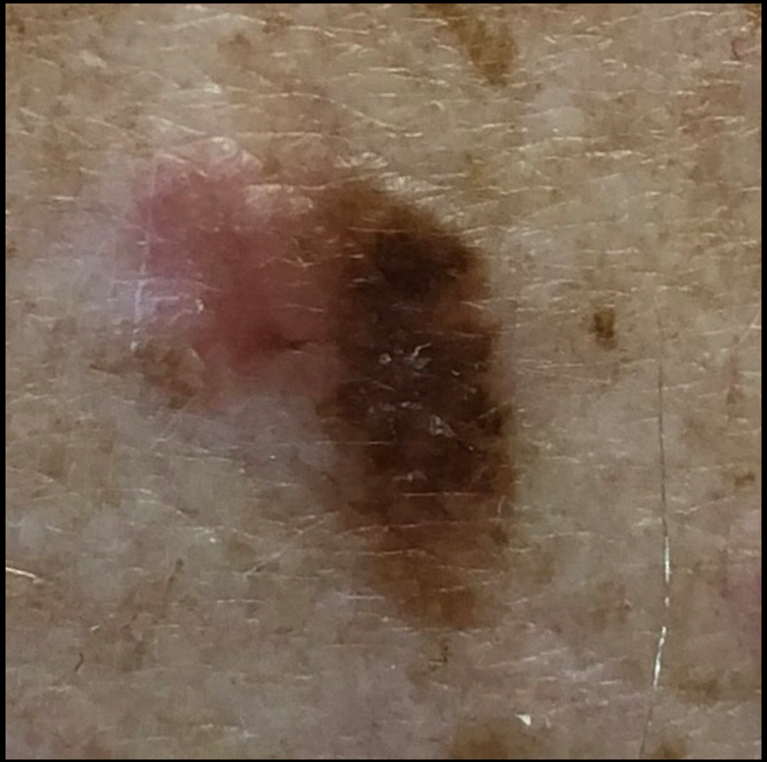

pT1a, 0.8 mm

Case number 146

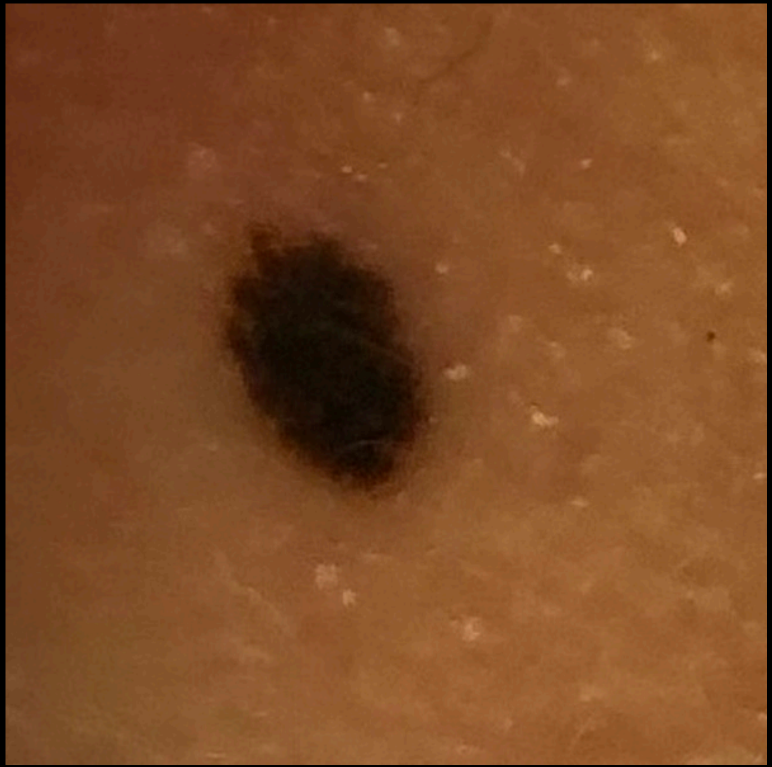

pTis

Case number 147

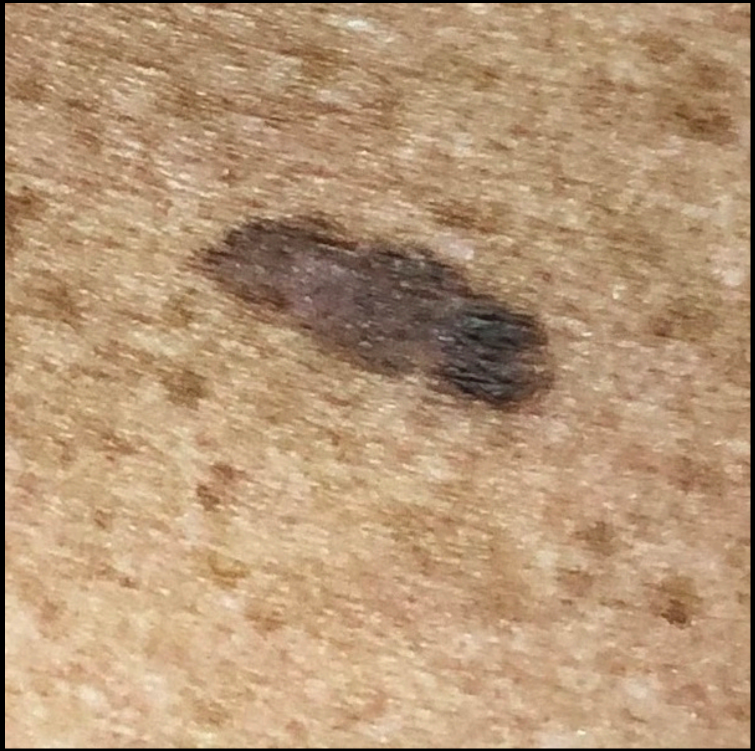

pT1a, 0.4 mm

Case number 148

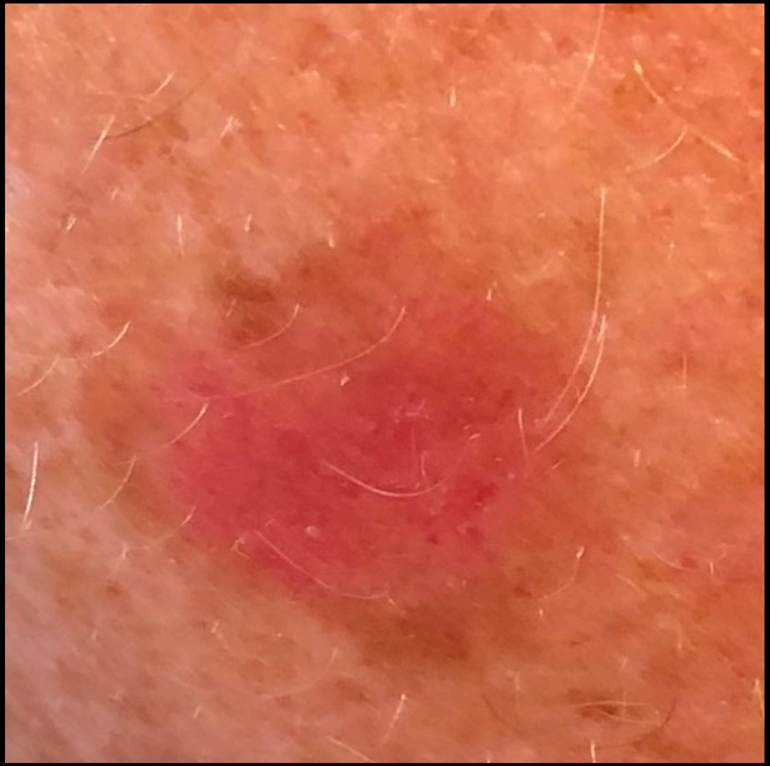

pTis

Case number 149

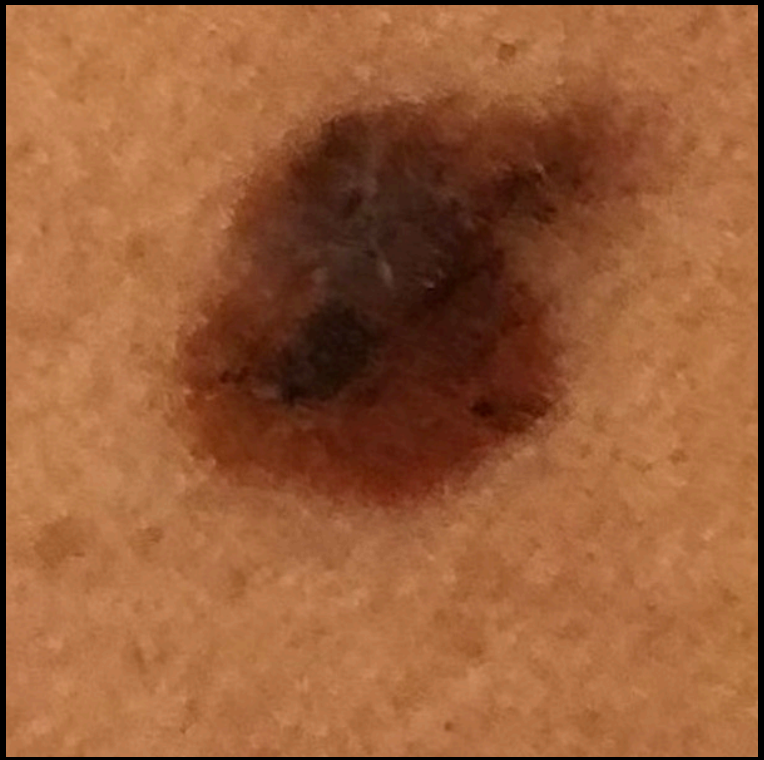

pTis

Case number 150

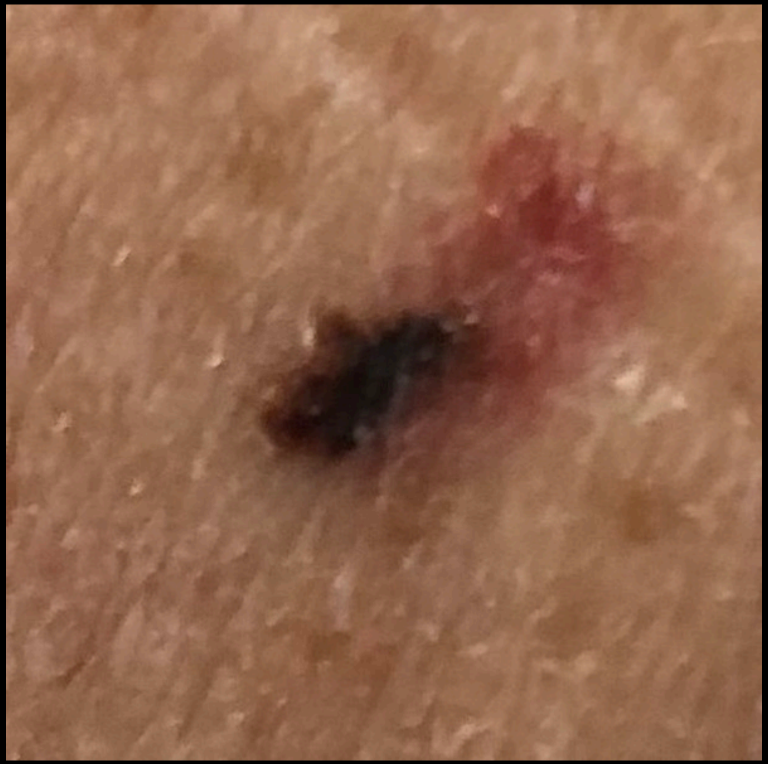

pT1a, 0.6 mm

Case number 151

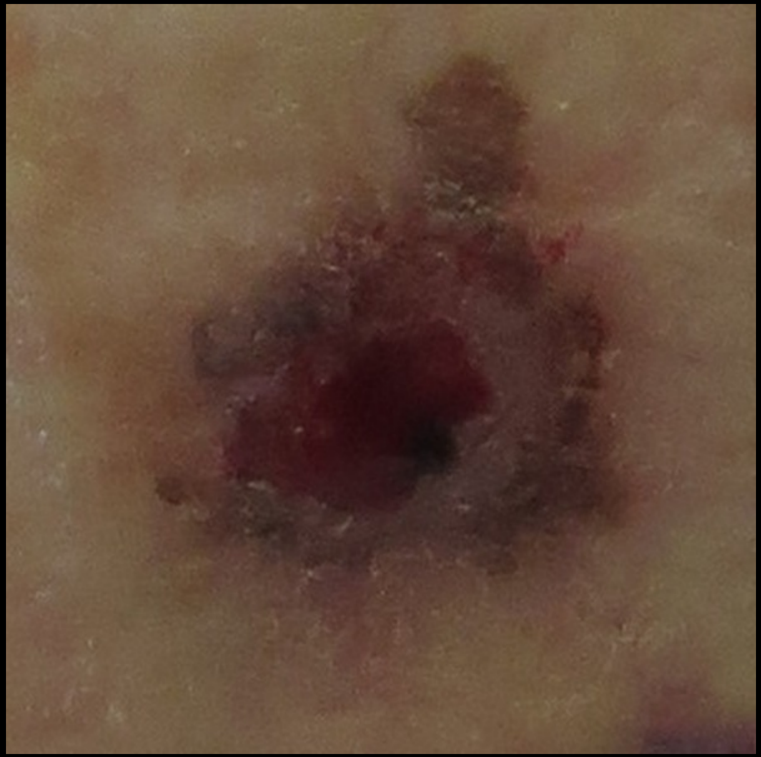

pT2a, 1.3 mm

Case number 152

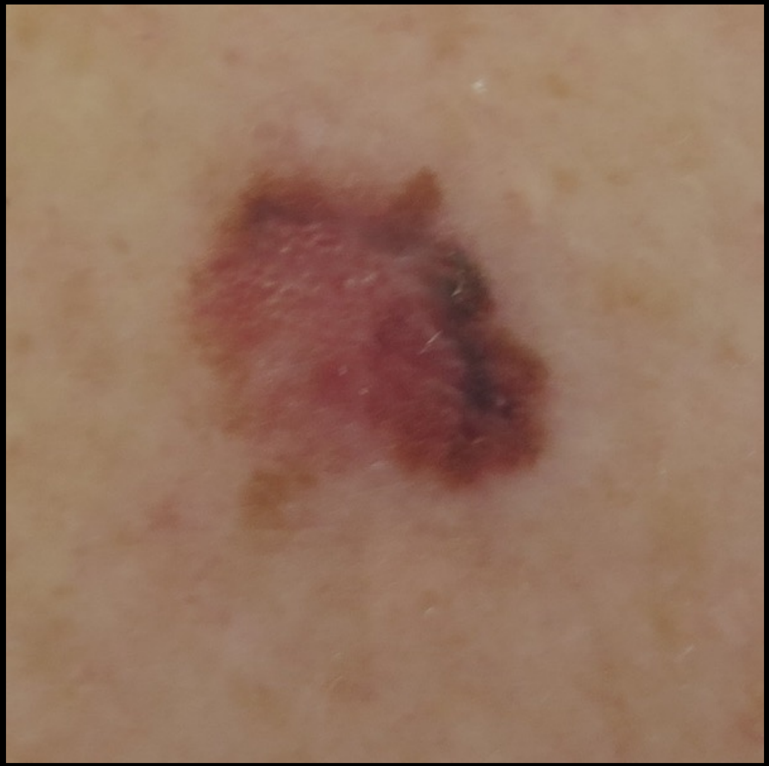

pT1a, 0.6 mm

Case number 153

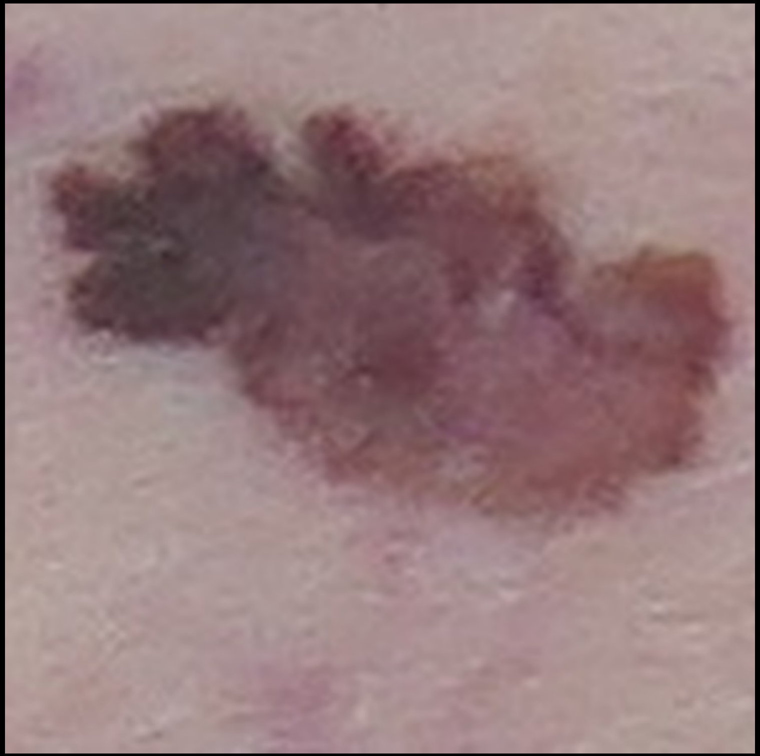

pT1a, 0.3 mm

Case number 154

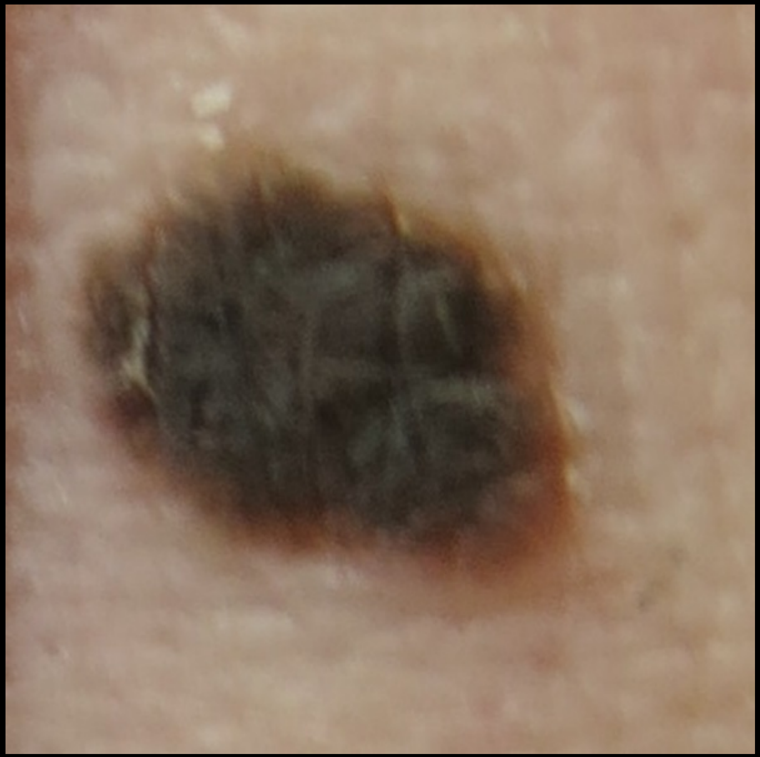

pTis

Case number 155

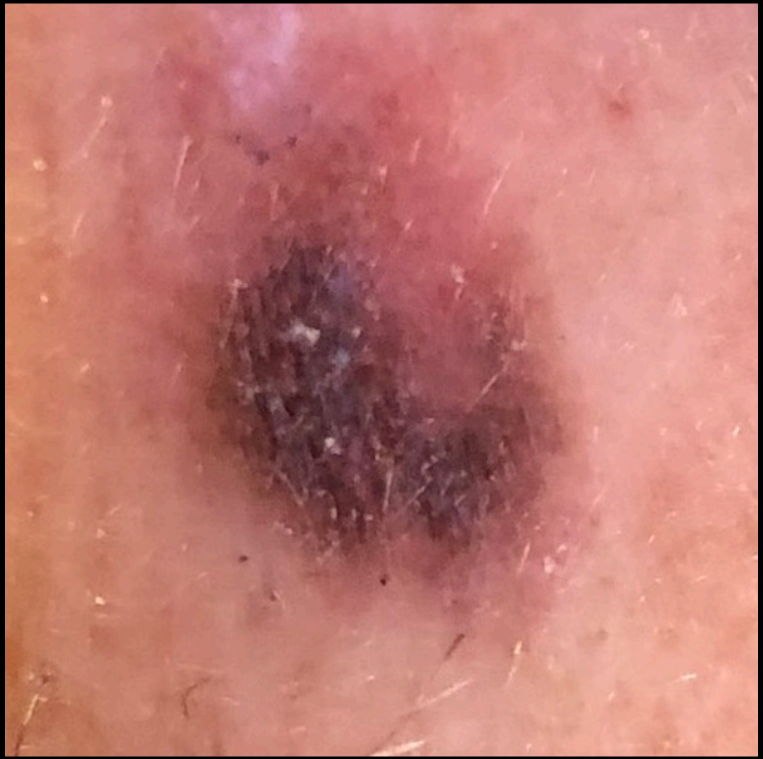

pTis

Case number 156

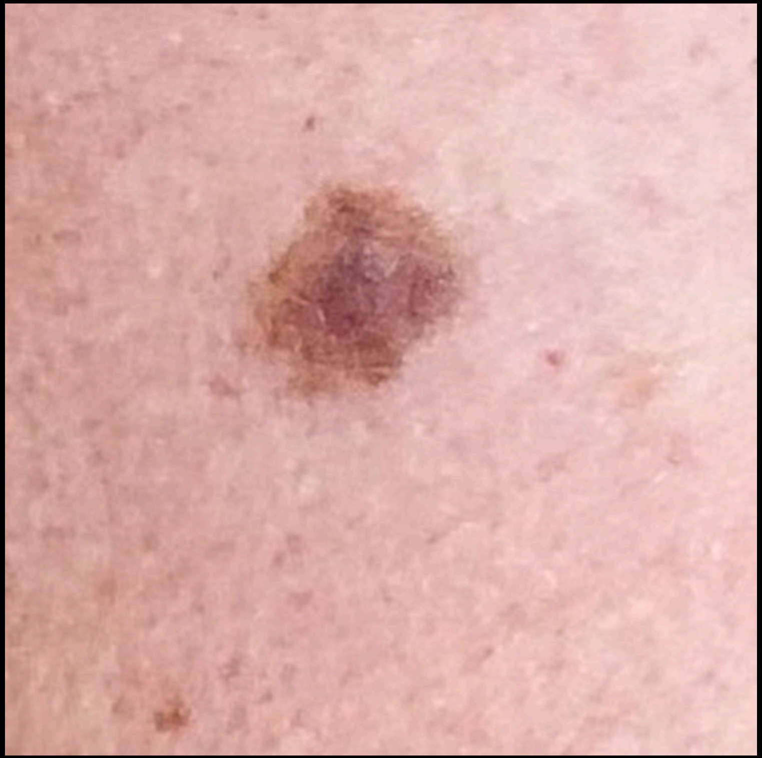

pT1a, 0.5 mm

Case number 157

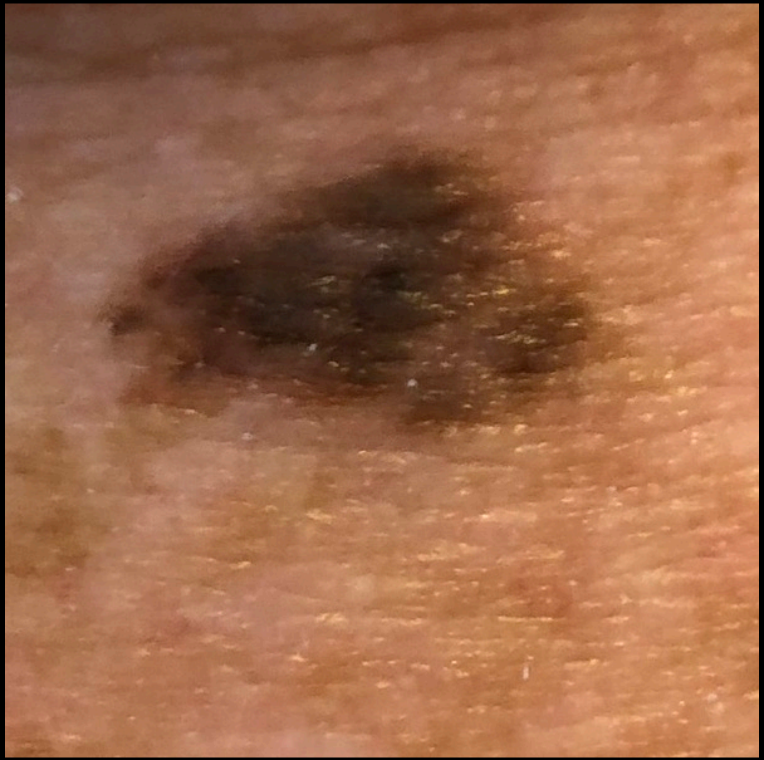

pT1a, 0.6 mm

Case number 158

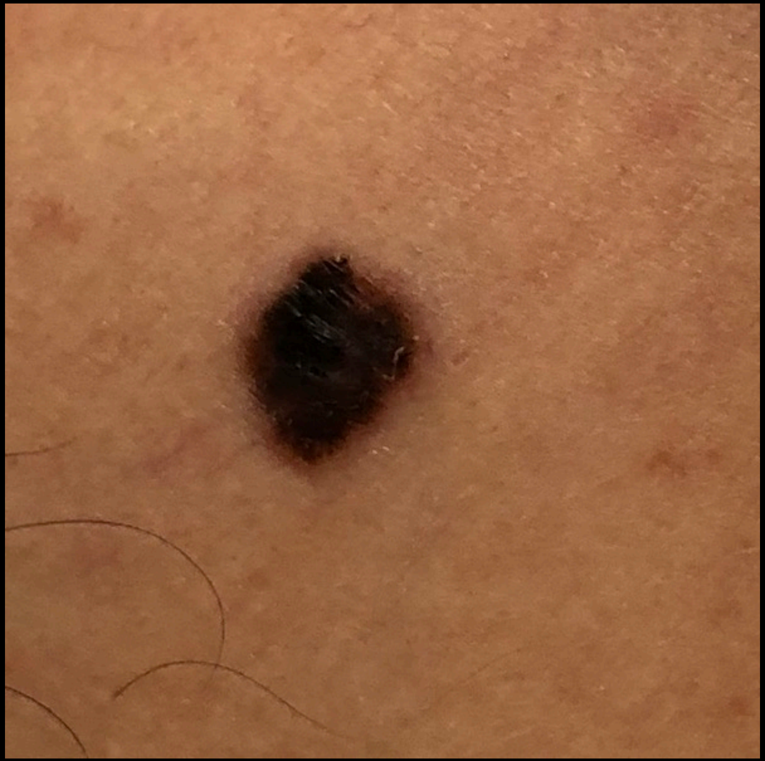

pT1a, 0.8 mm

Case number 159

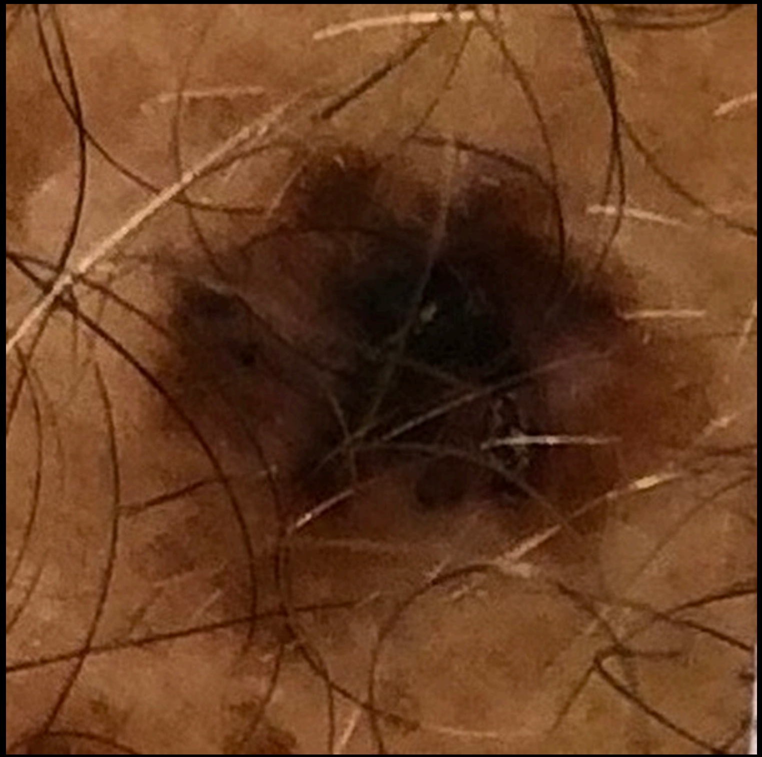

pT1b, 0.8 mm

Case number 160

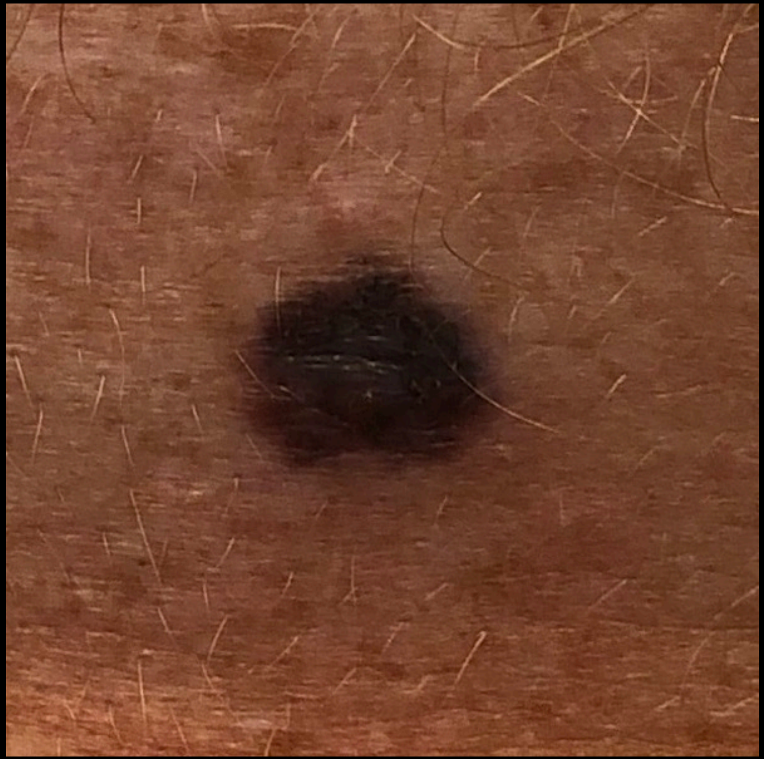

pT2a, 1.2 mm

Case number 161

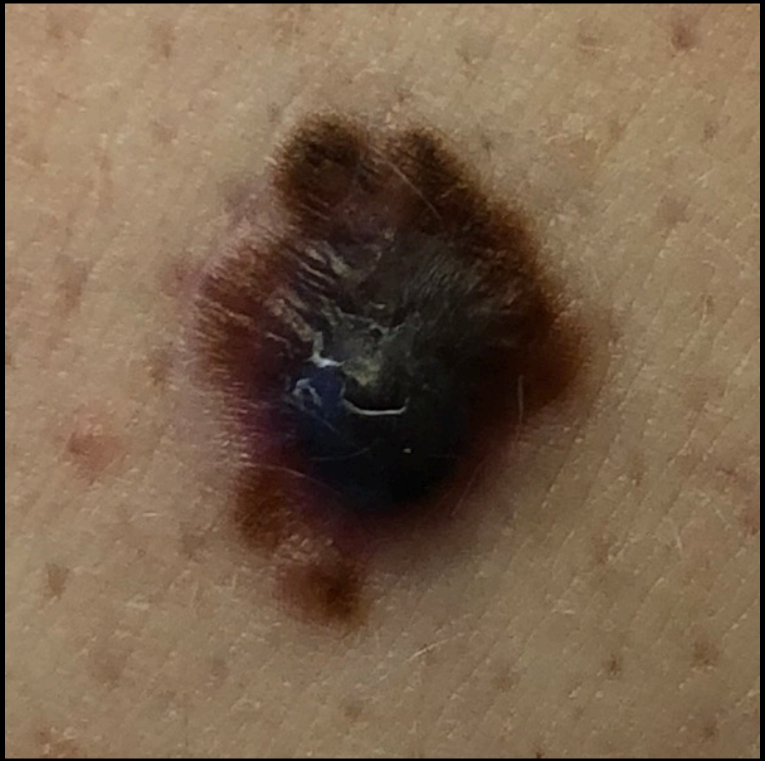

pT3a, 2.5 mm

Case number 162

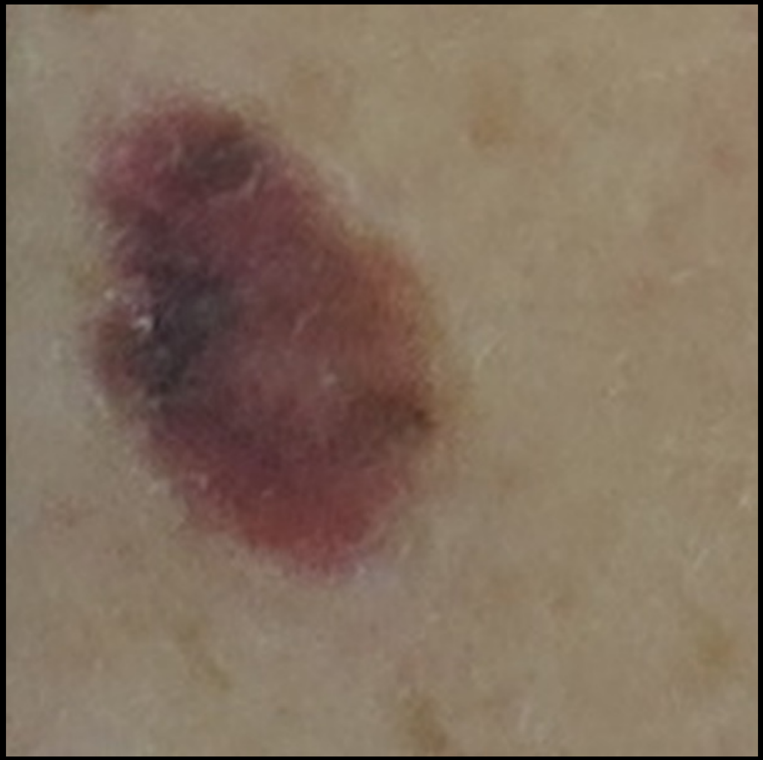

pT1a, 0.9 mm

Case number 163

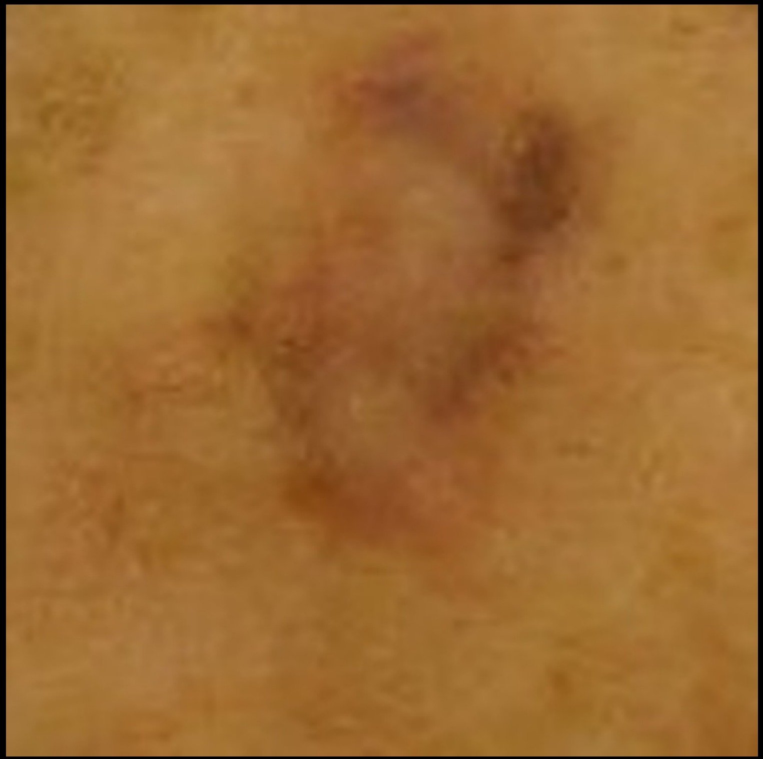

pTis

Case number 164

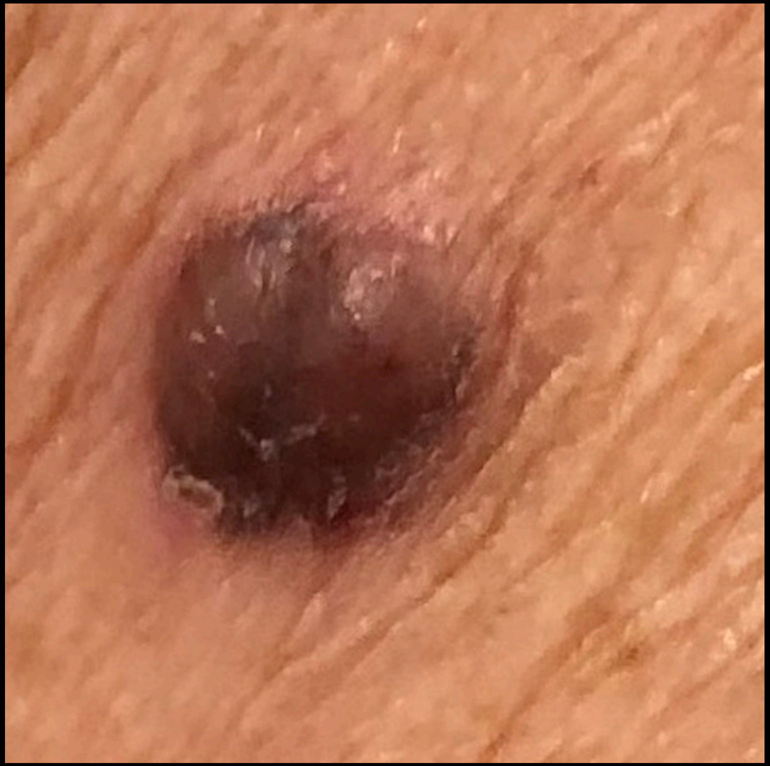

pT4a, 8.5 mm

Case number 165

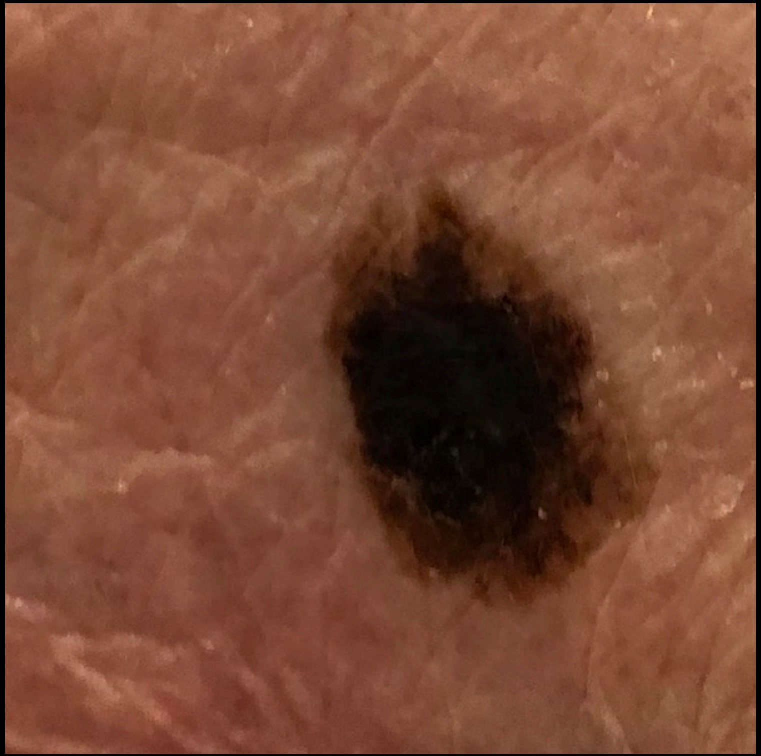

pT1a, 0.4 mm

Case number 166

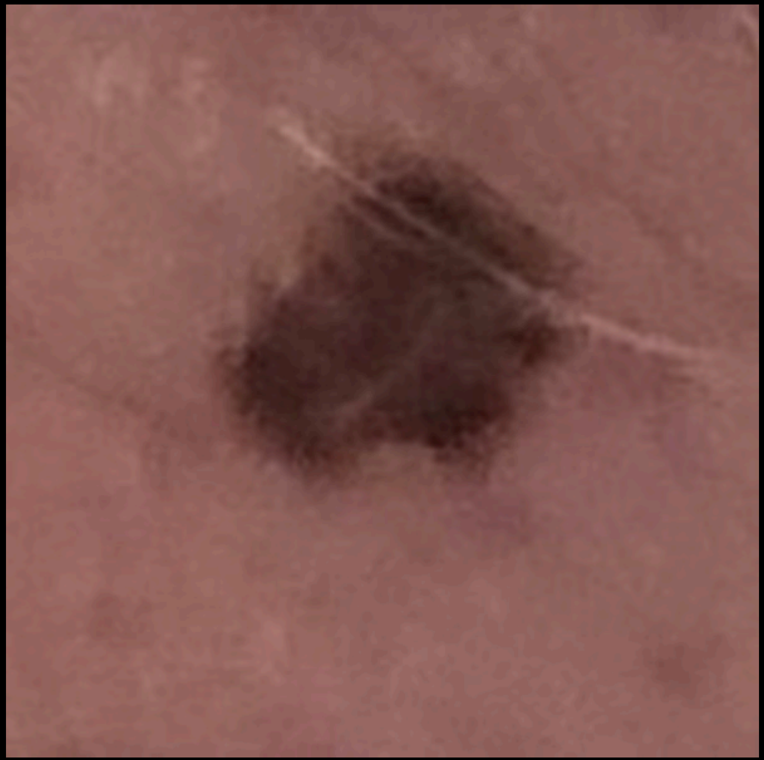

pTis

Case number 167

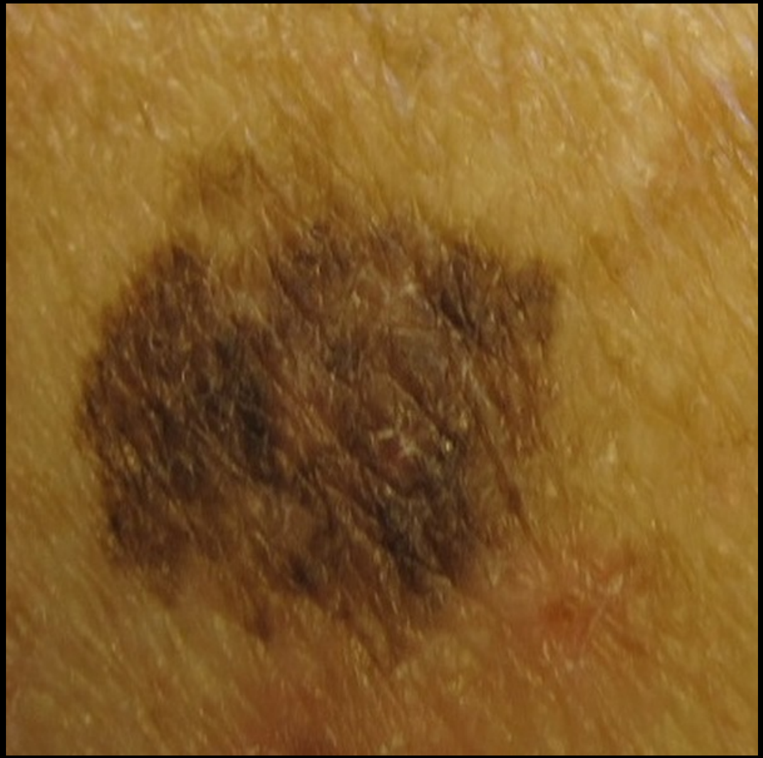

pTis

Case number 168

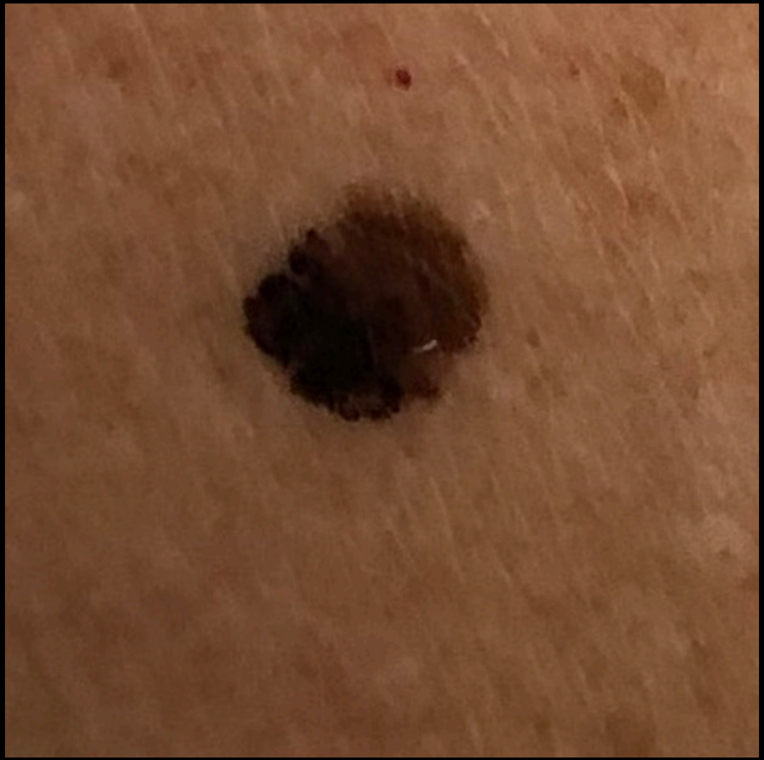

pTis

Case number 169

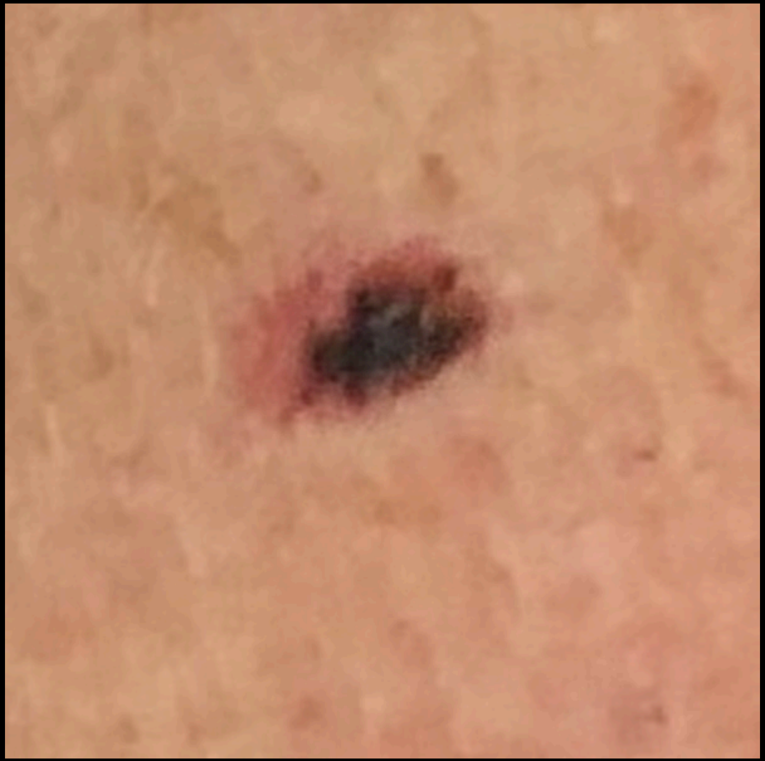

pT1a, 0.7 mm

Case number 170

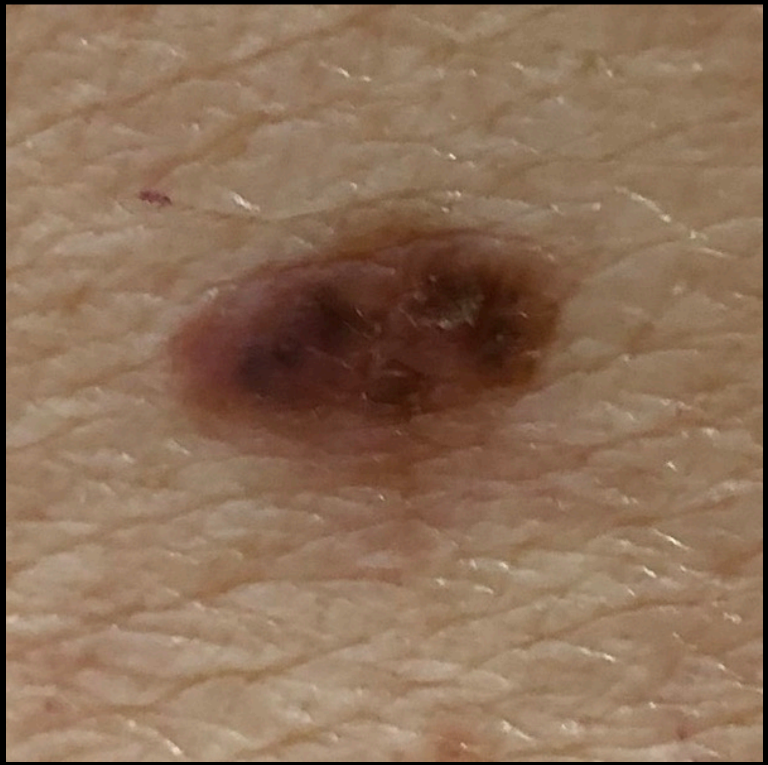

pT1a, 0.6 mm

Case number 171

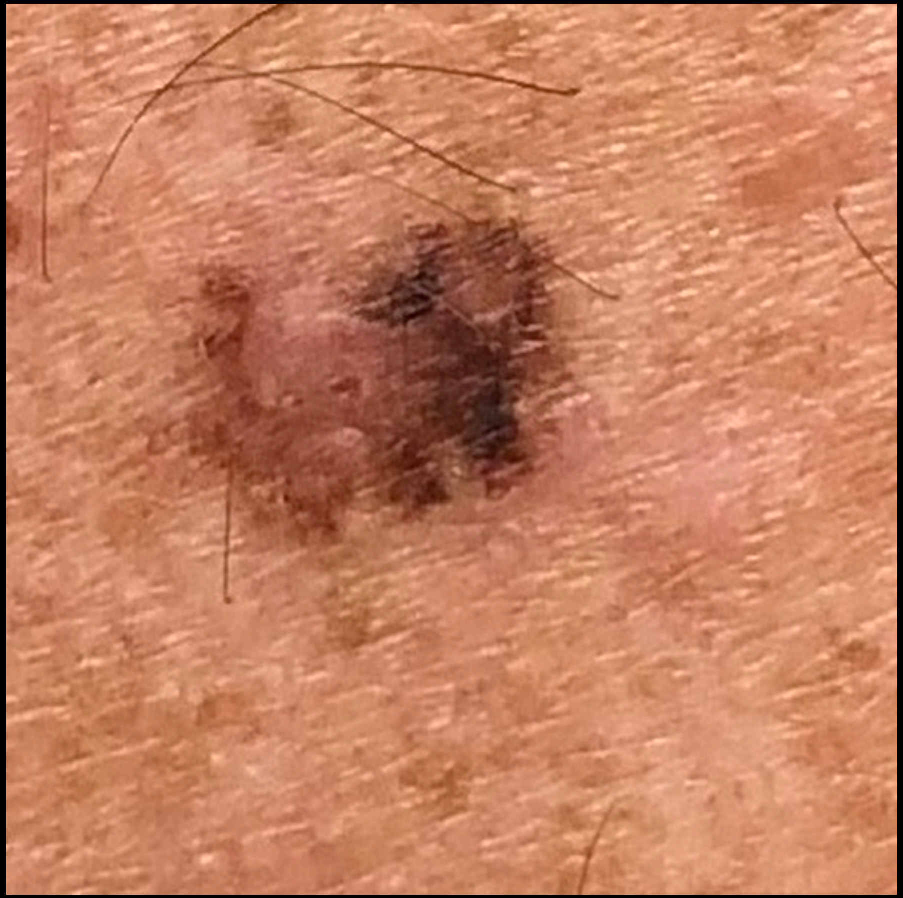

pTis

Case number 172

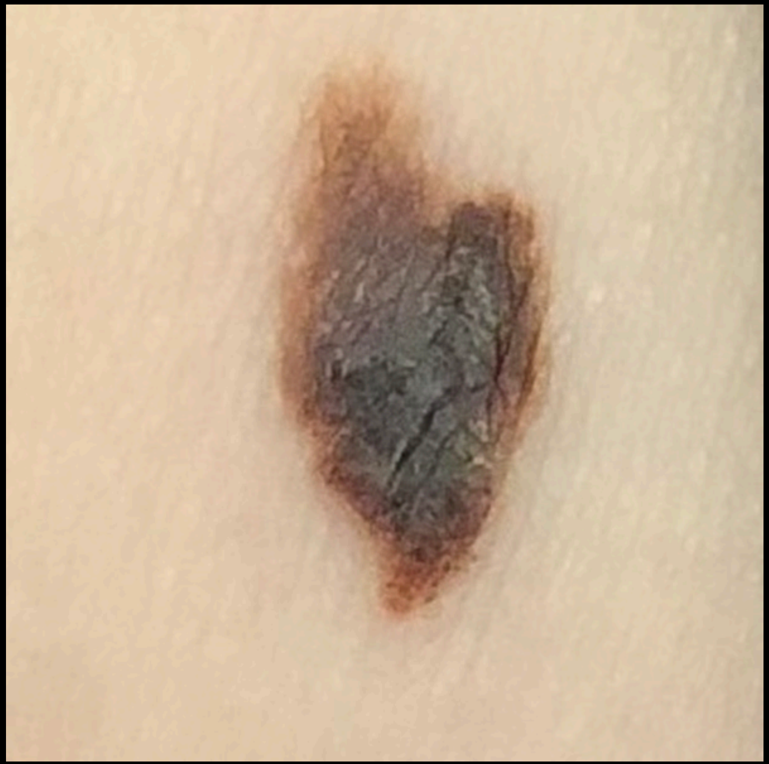

pT1a, 0.5 mm

Case number 173

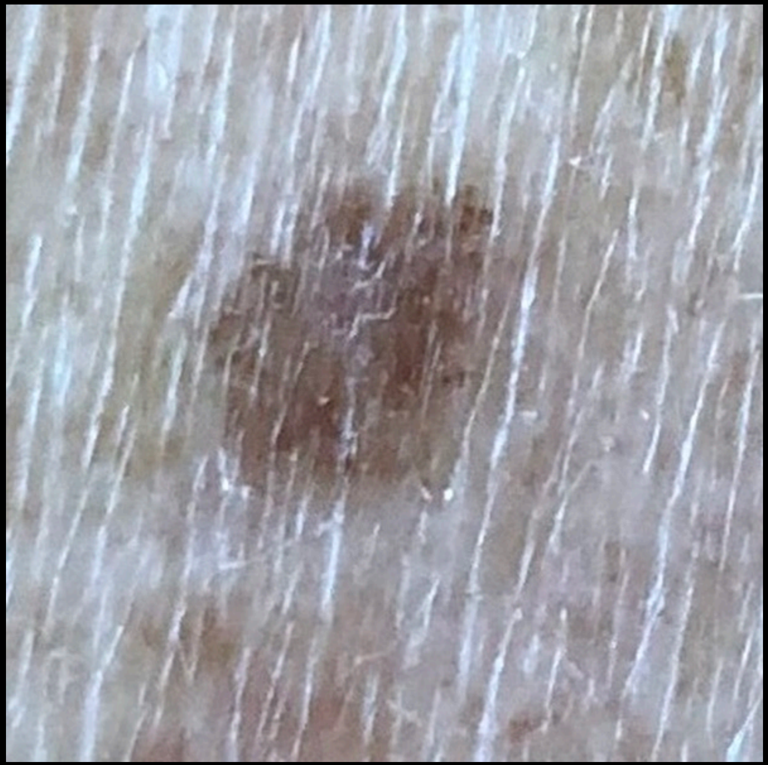

pTis

Case number 174

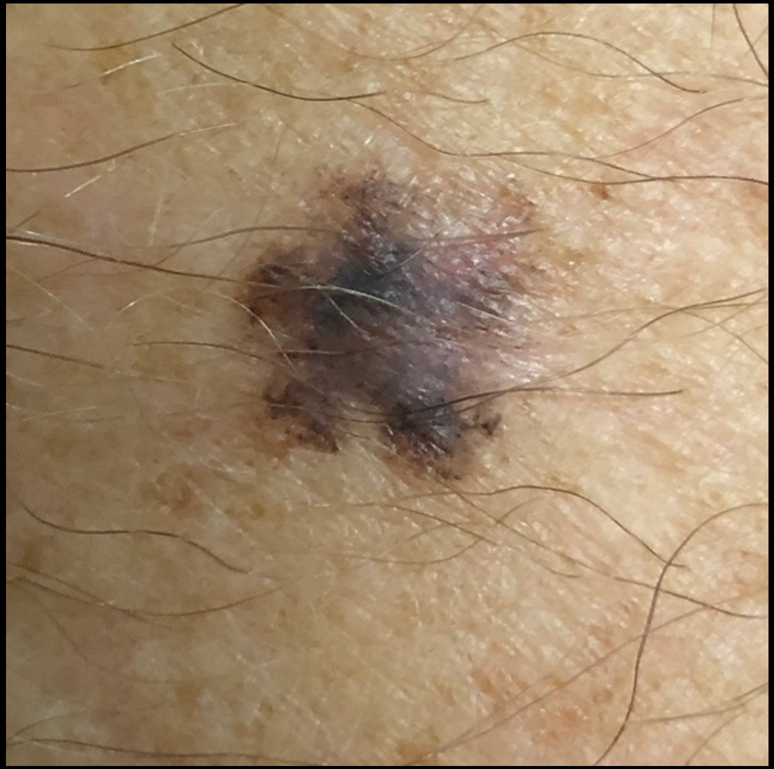

pTis

Case number 175

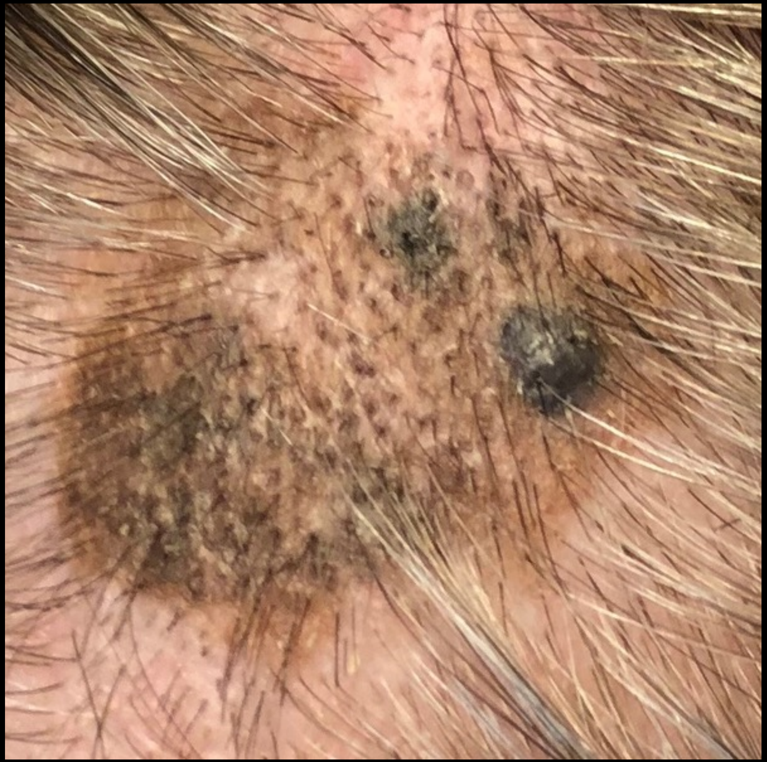

pT2a, 1.4 mm

Case number 176

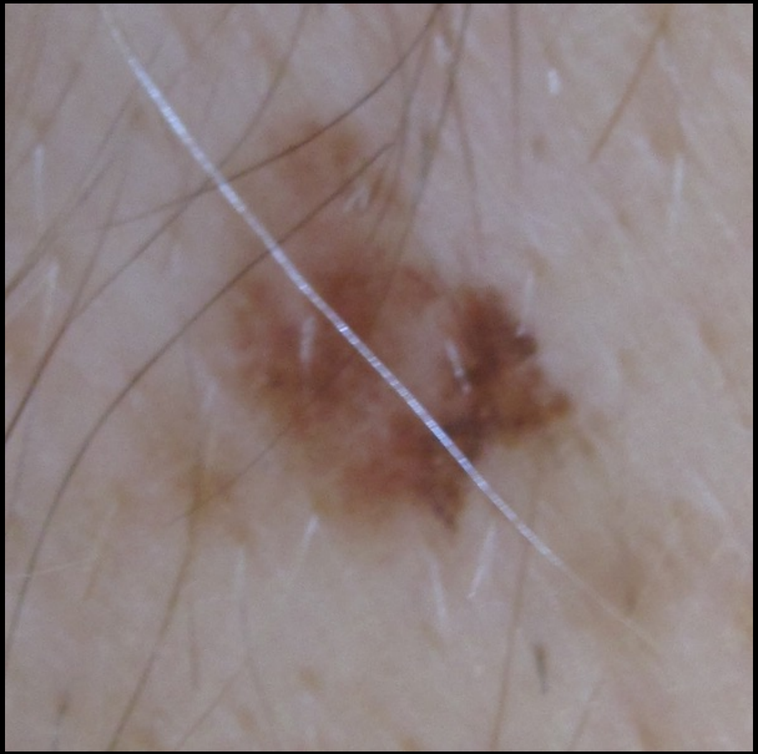

pTis

Case number 177

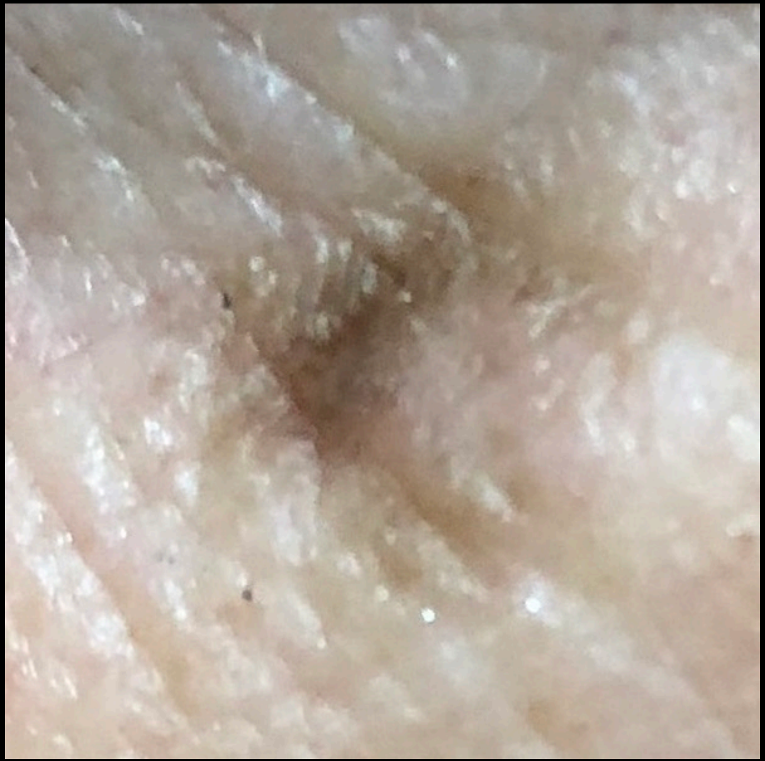

pTis

Case number 178

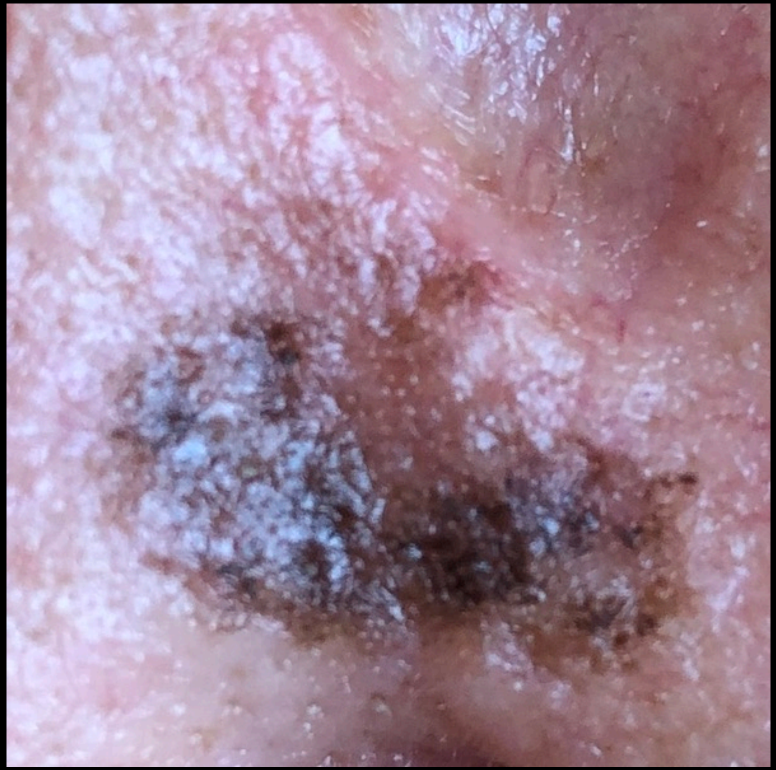

pTis

Case number 179

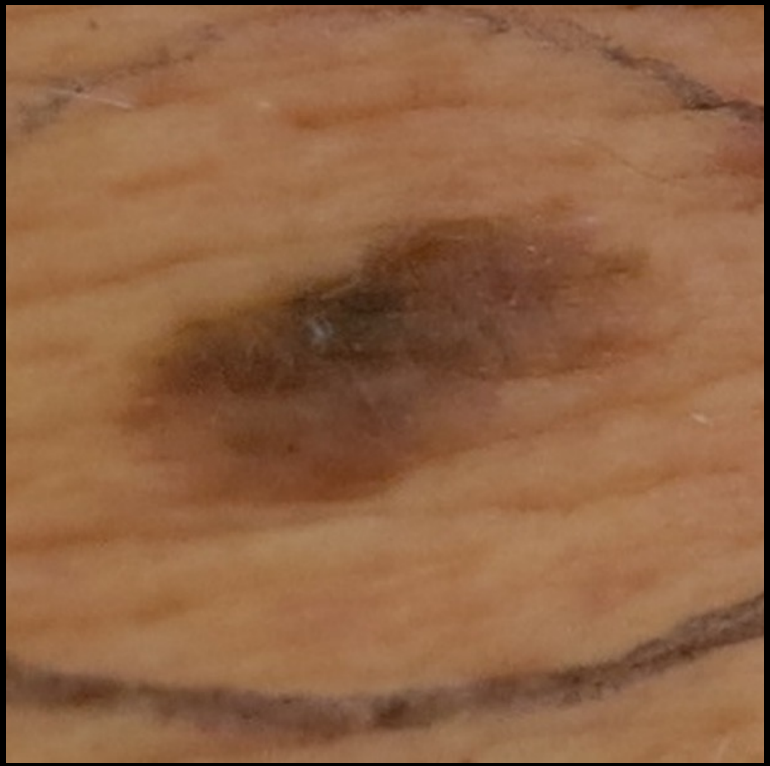

pT1a, 0.4 mm

Case number 180

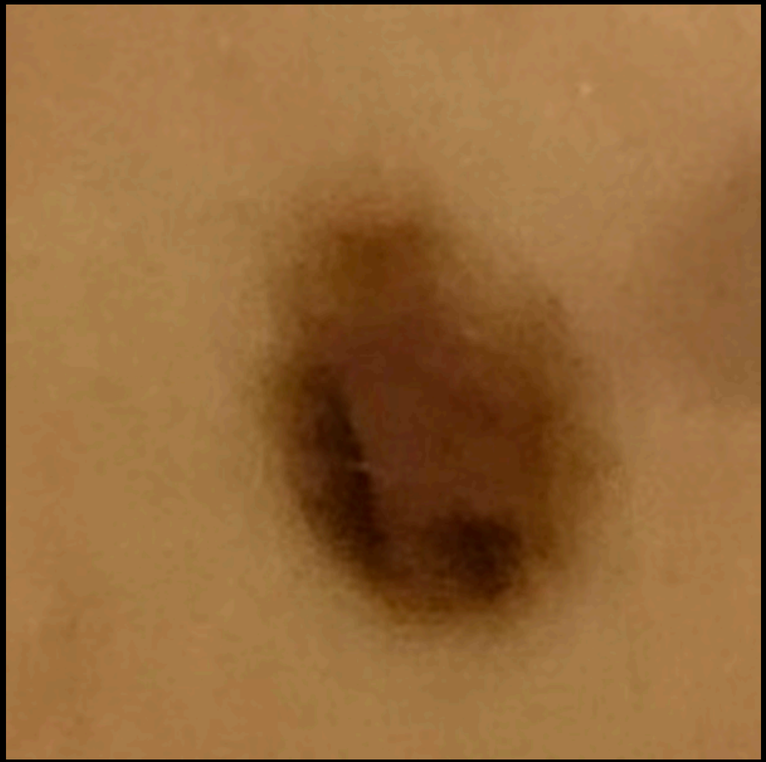

pTis

Case number 181

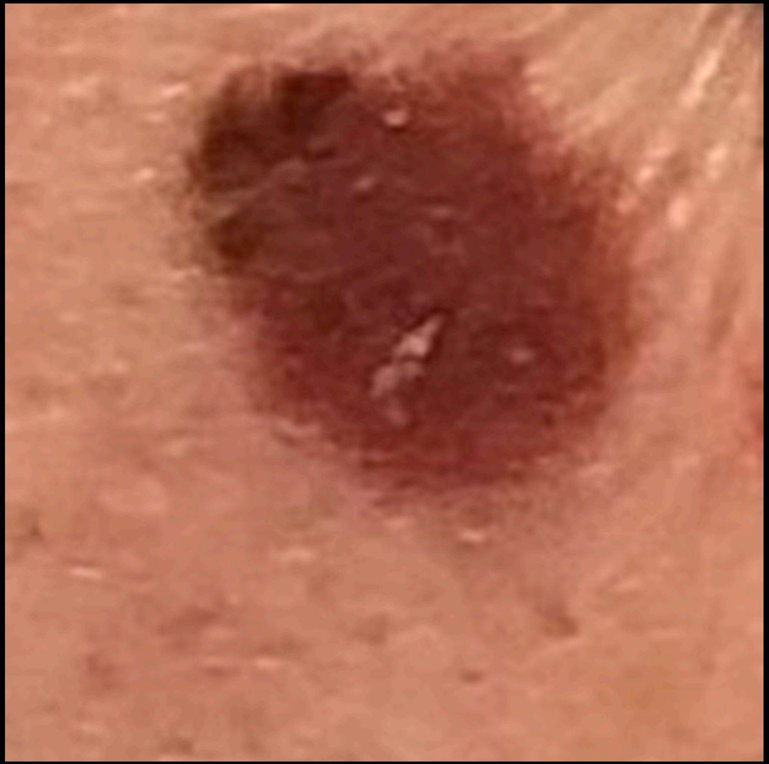

pTis

Case number 182

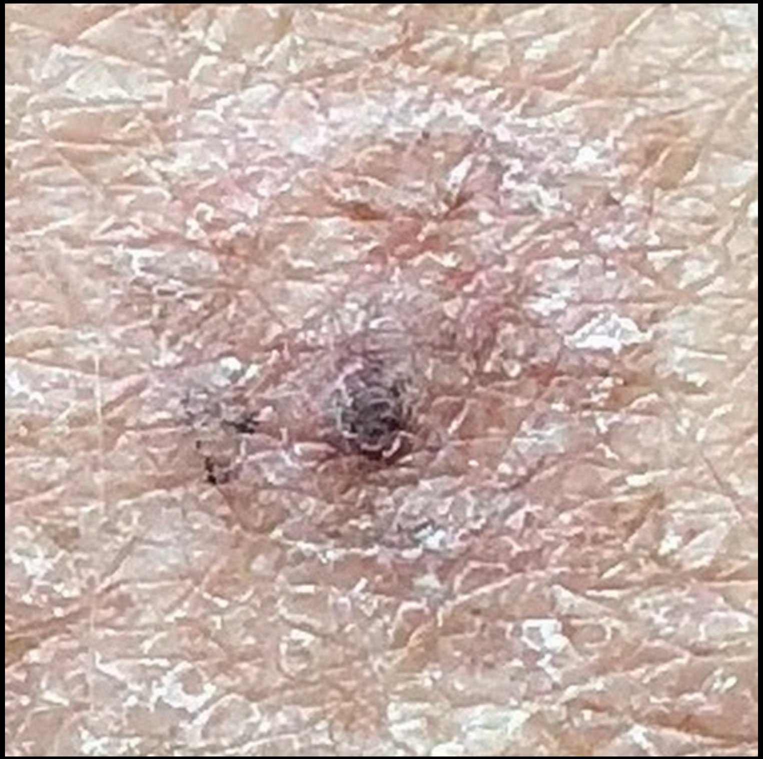

pTis

Case number 183

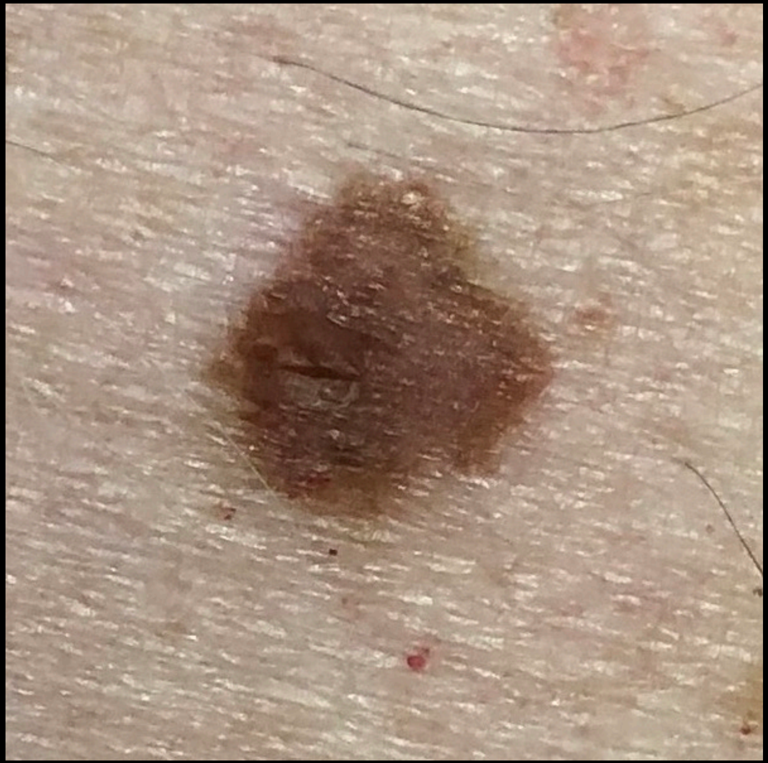

pTis

Case number 184

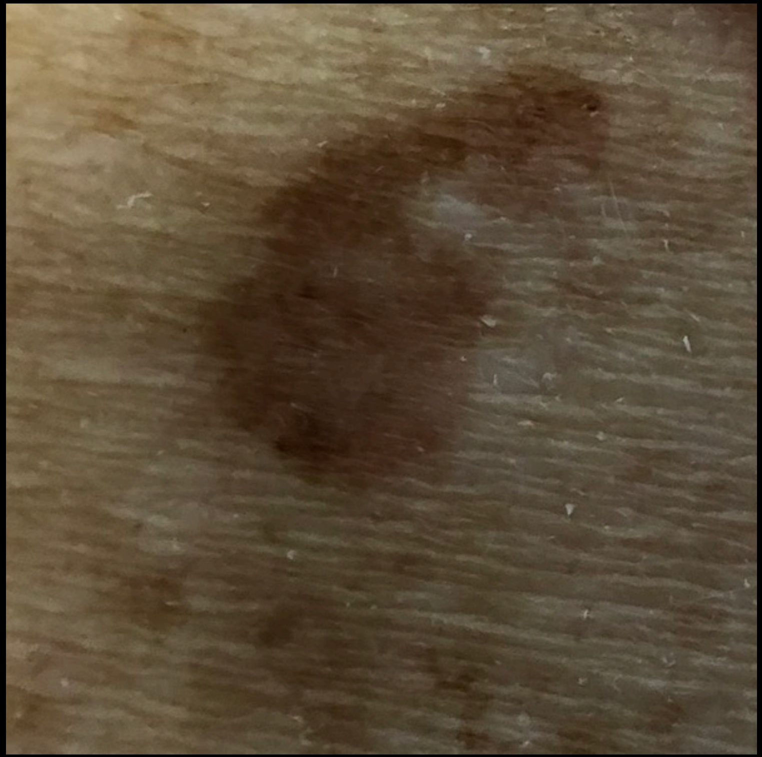

pTis

Case number 185

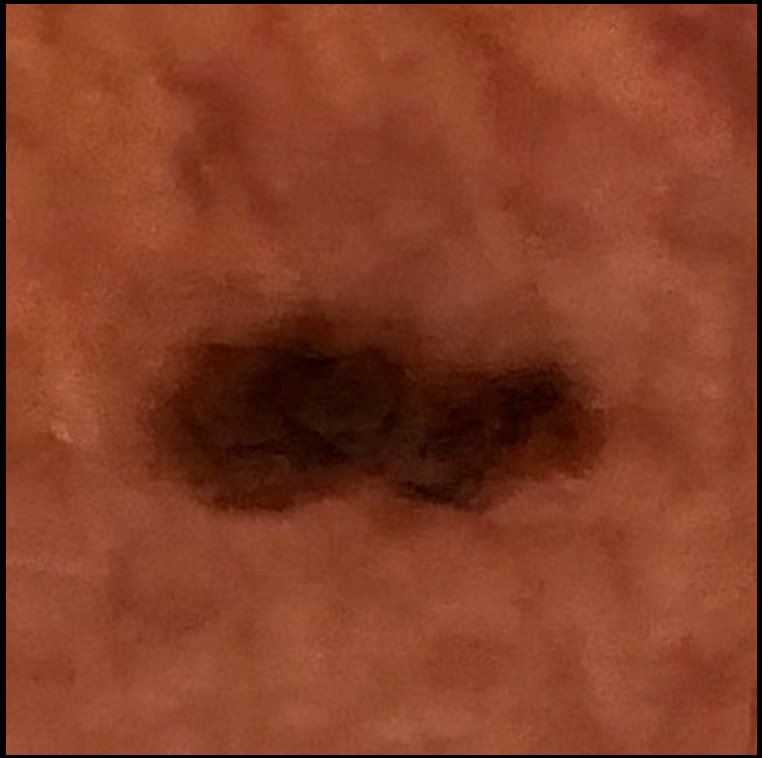

pTis

Case number 186

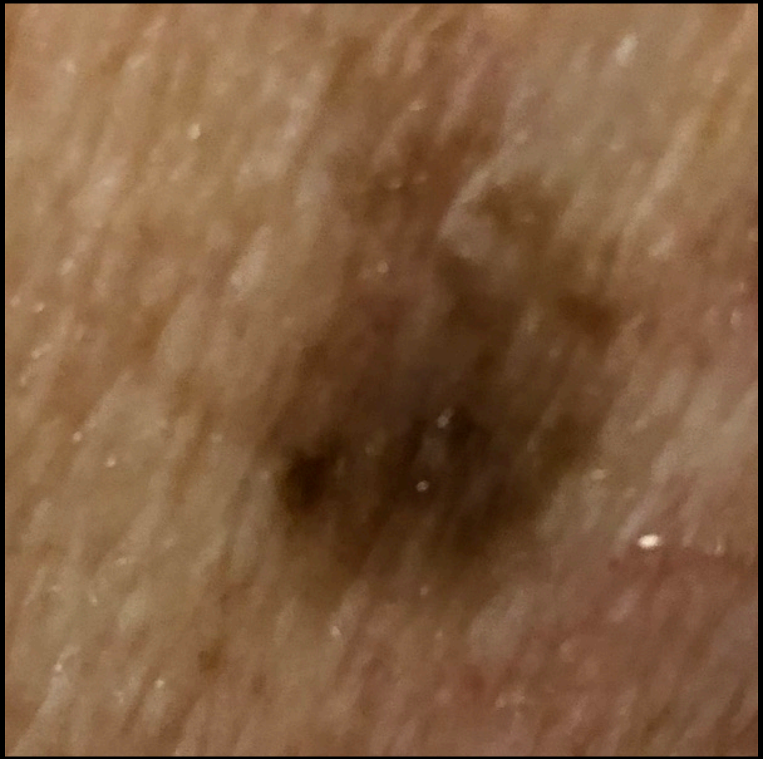

pTis

Case number 187

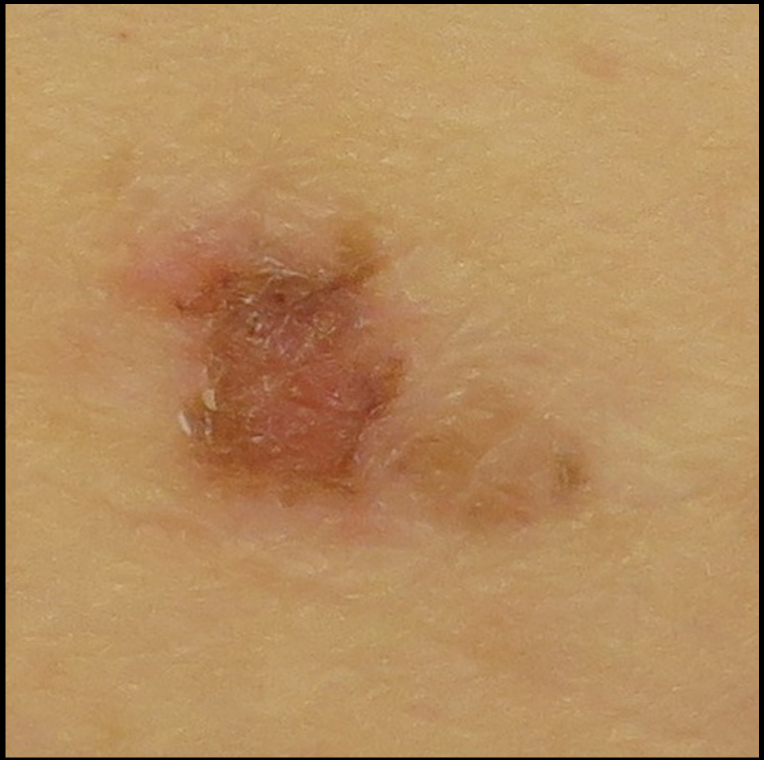

pT1a, 0.5 mm

Case number 188

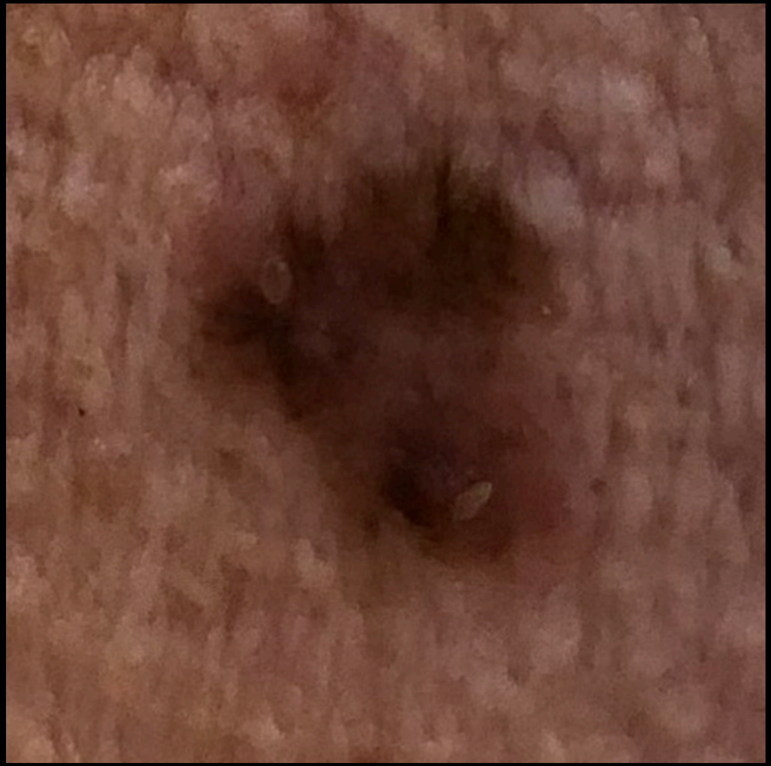

pT1a, 0.5 mm

Case number 189

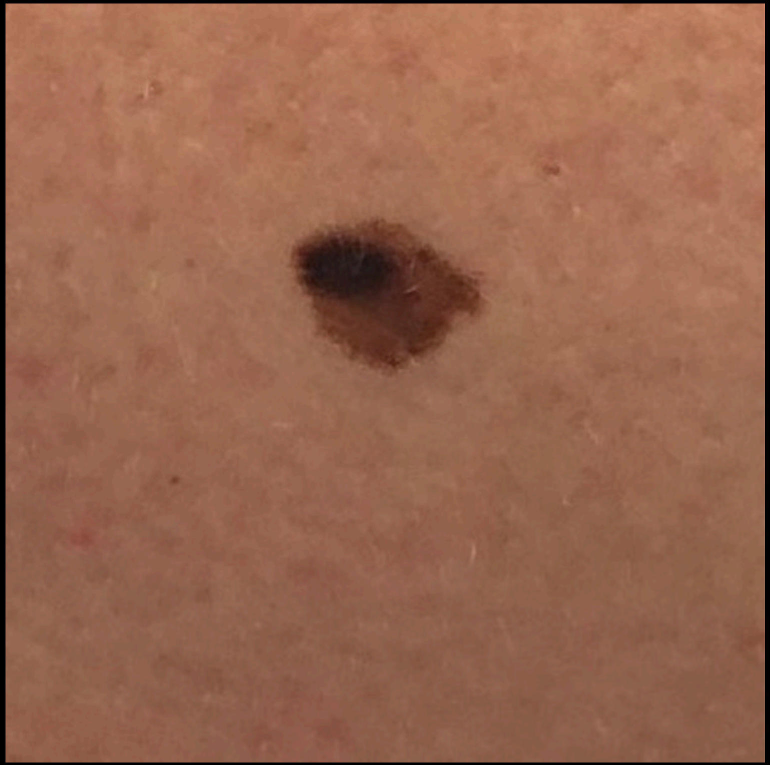

pTis

Case number 190

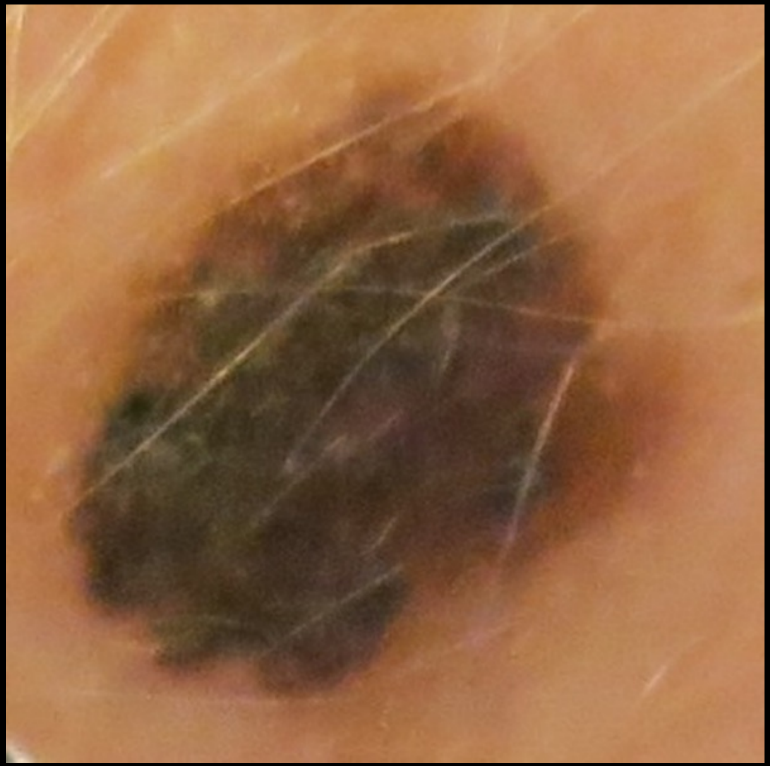

pTis

Case number 191

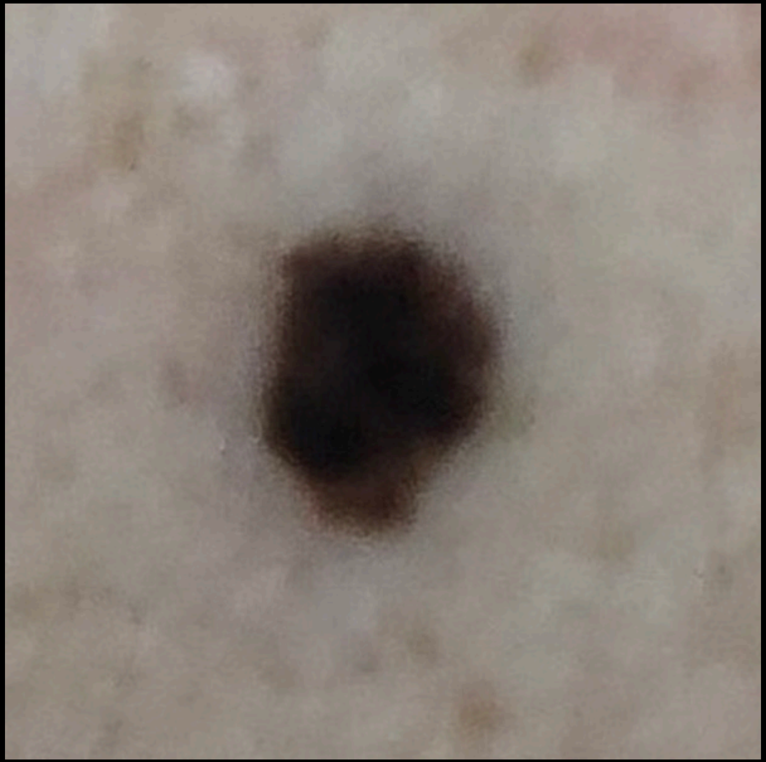

pTis

Case number 192

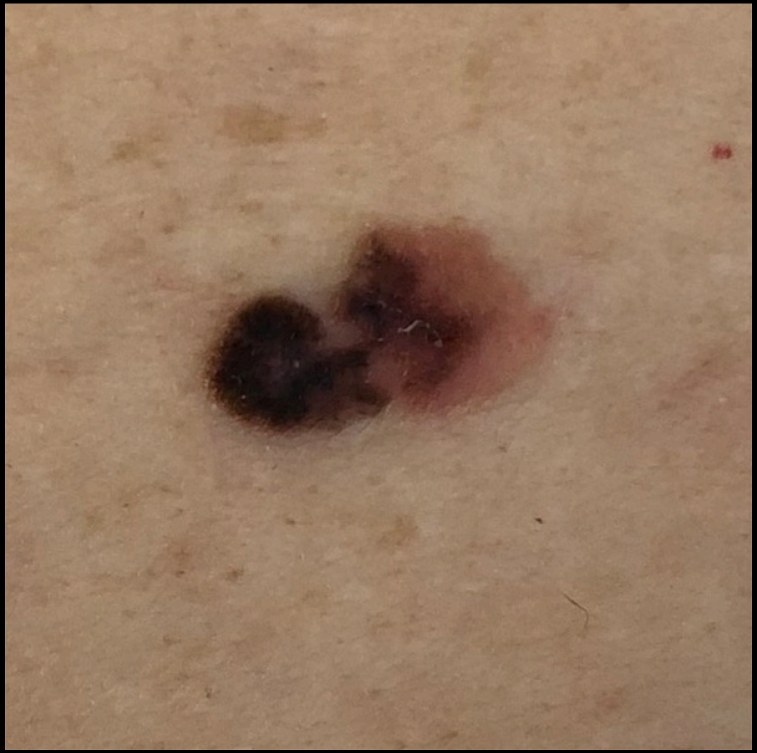

pT1a, 0.5 mm

Case number 193

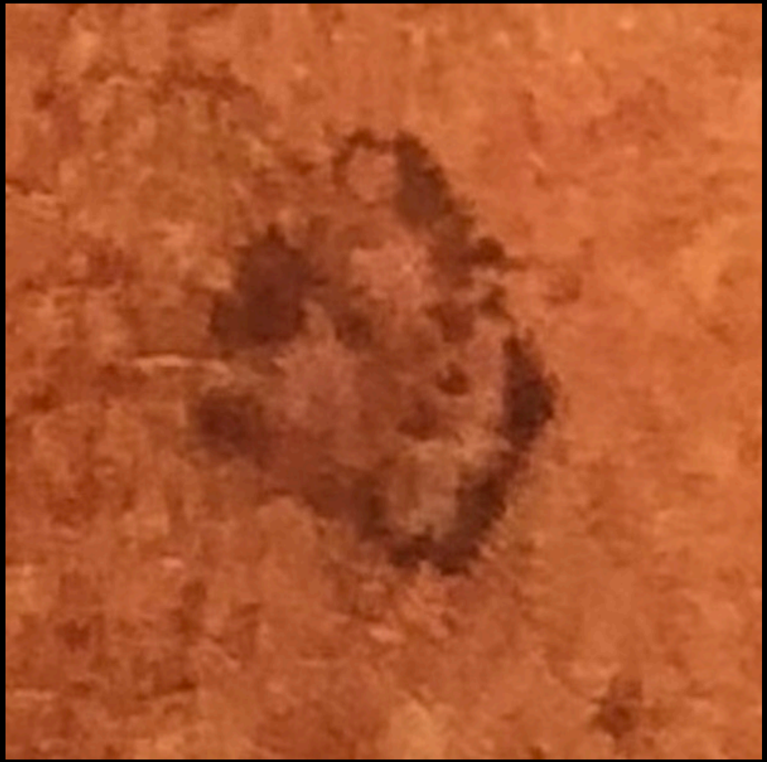

pTis

Case number 194

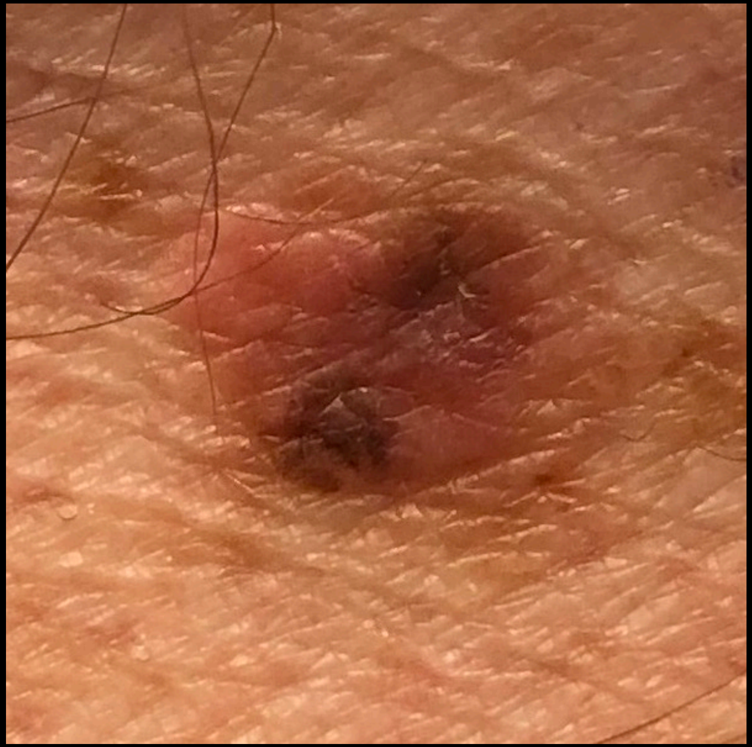

pT1a, 0.6 mm

Case number 195

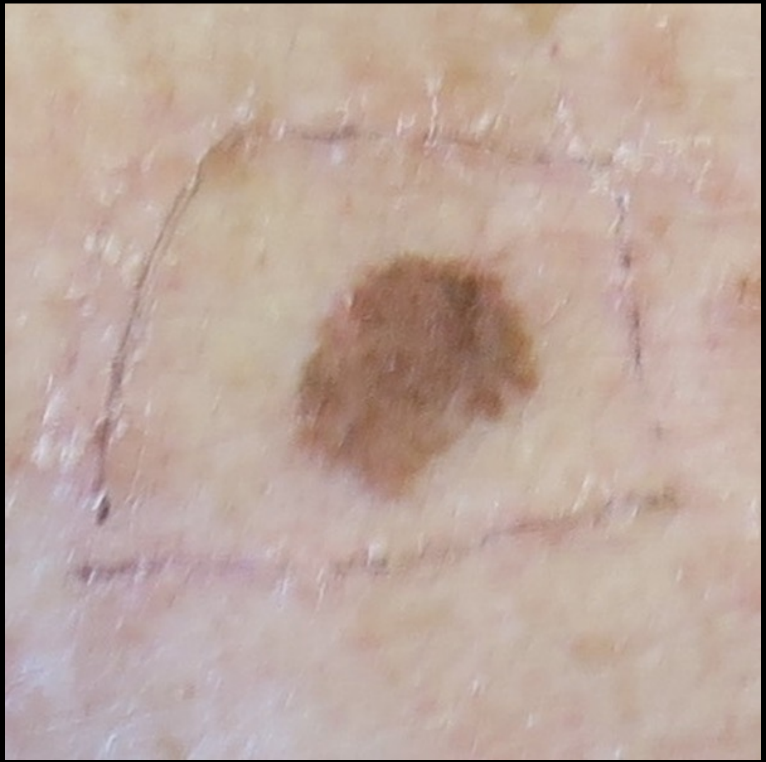

pTis

Case number 196

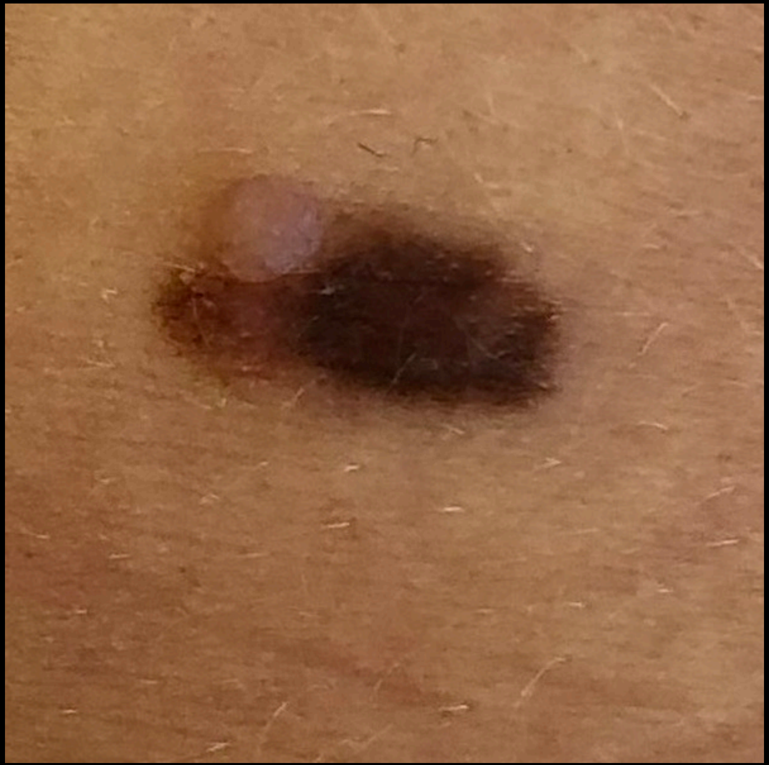

pTis

Case number 197

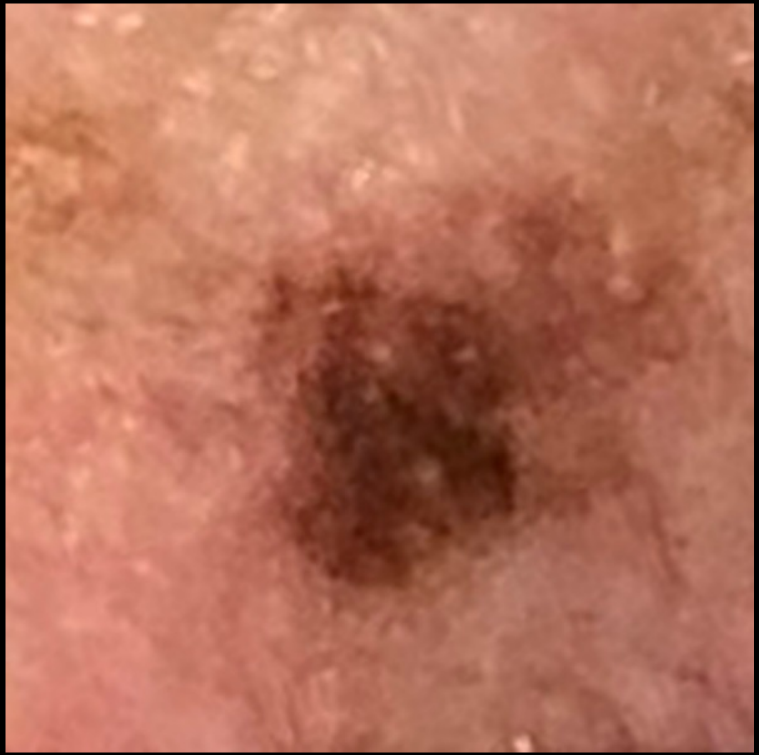

pTis

Case number 198

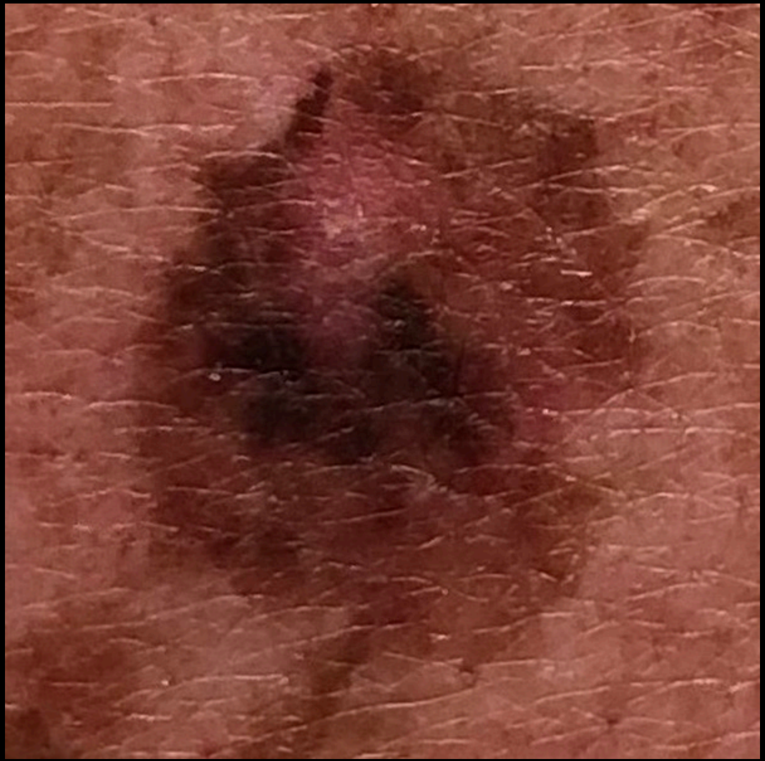

pT1a, 0.7 mm

Case number 199

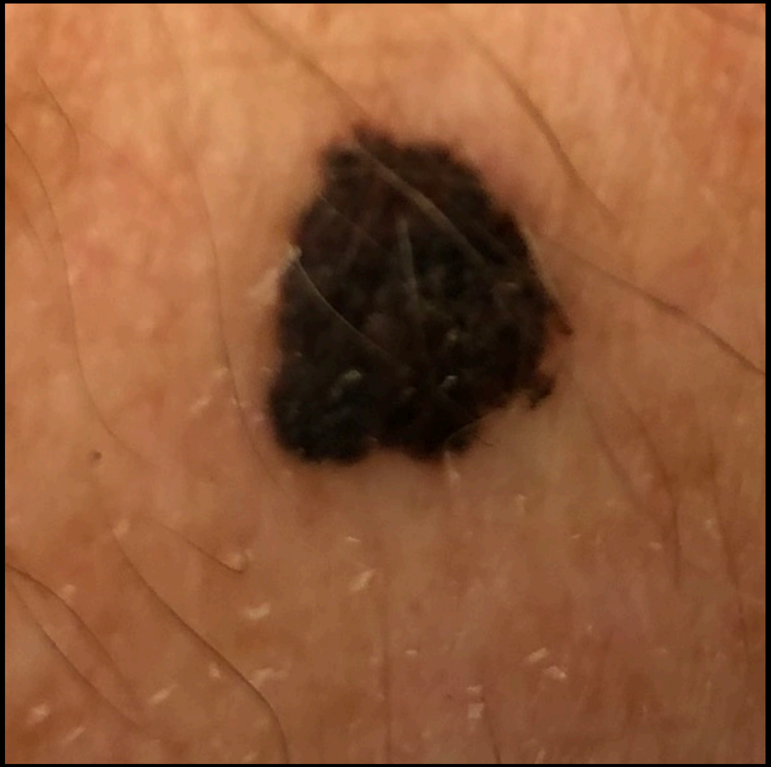

pTis

Case number 200

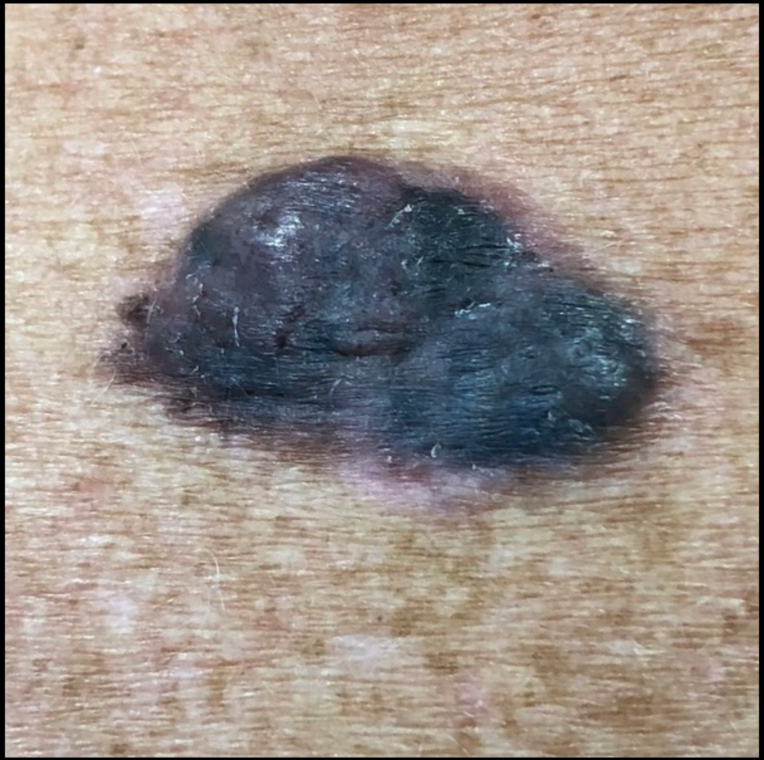

pT3a, 3.3 mm

Case number 201

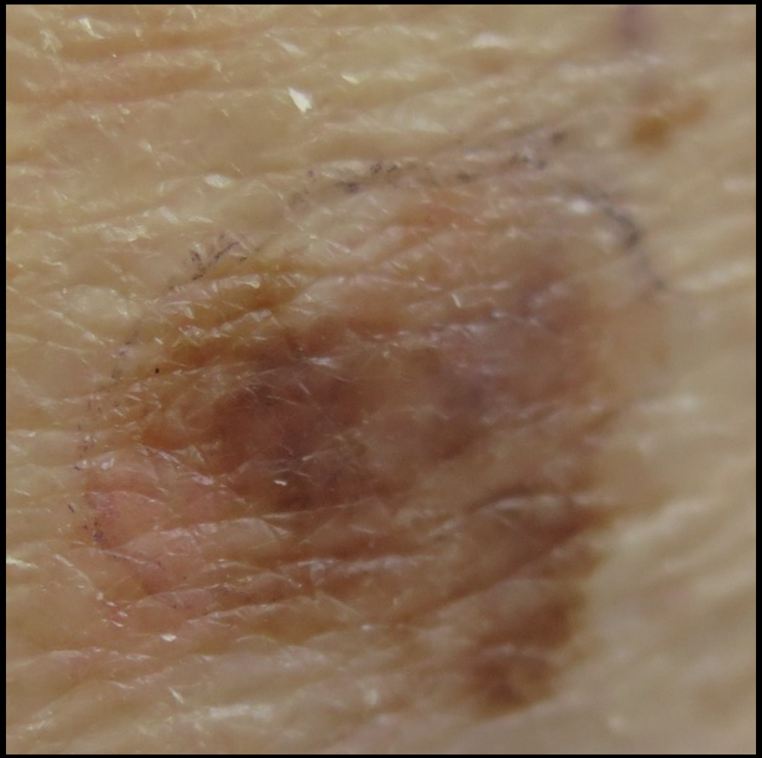

pTis

Case number 202

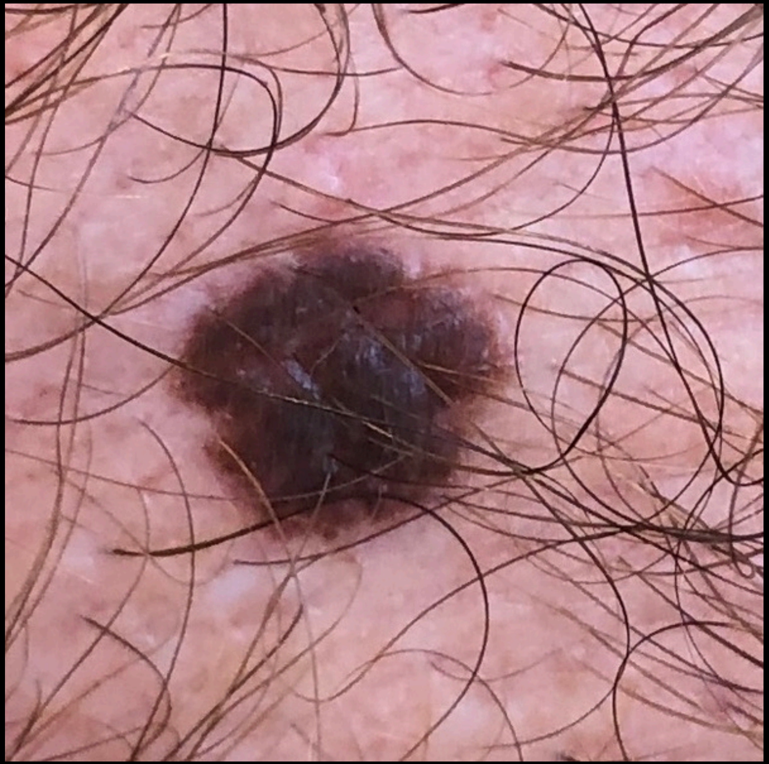

pT1a, 0.3 mm

Case number 203

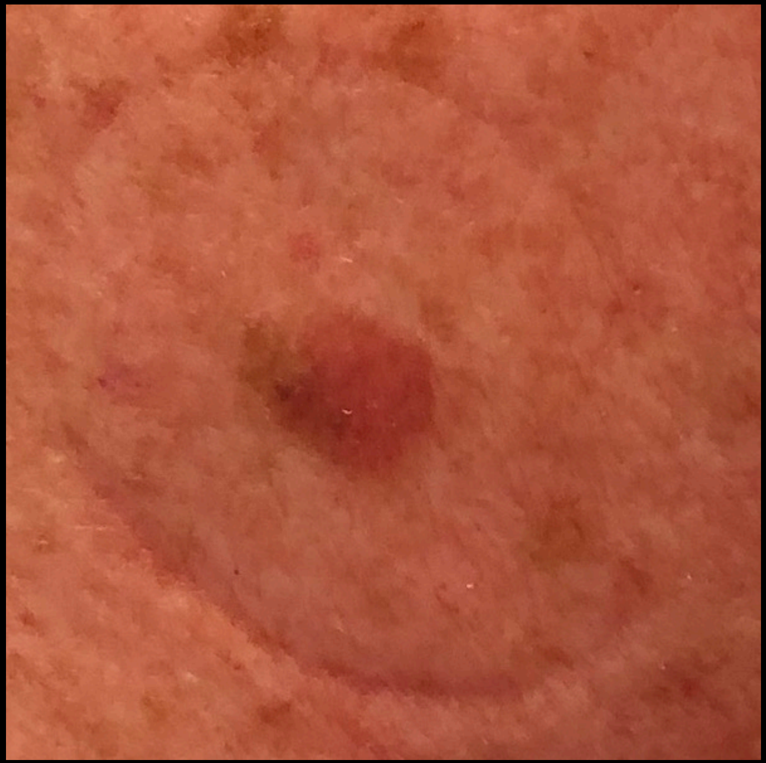

pTis

Case number 204

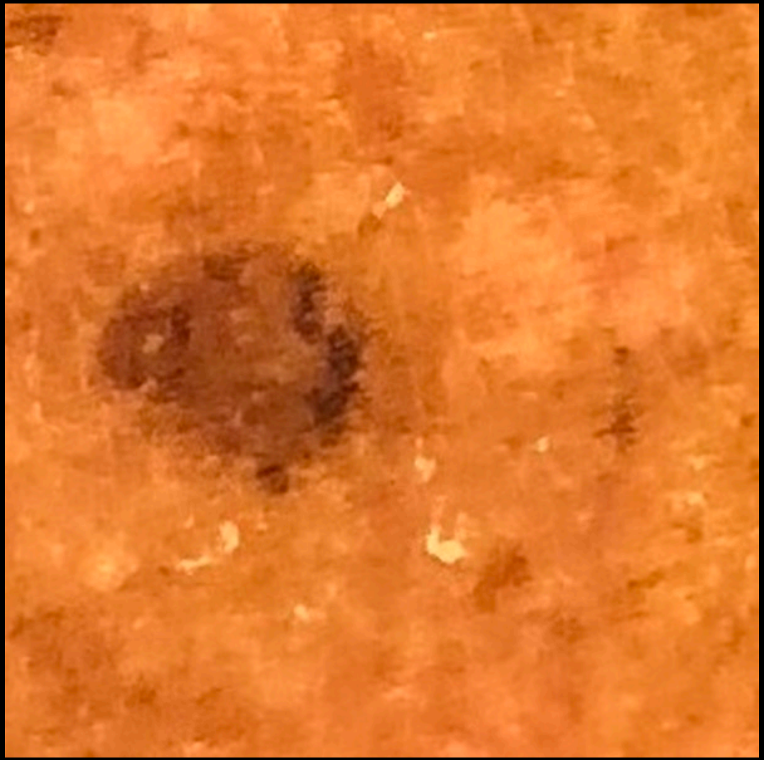

pTis

Case number 205

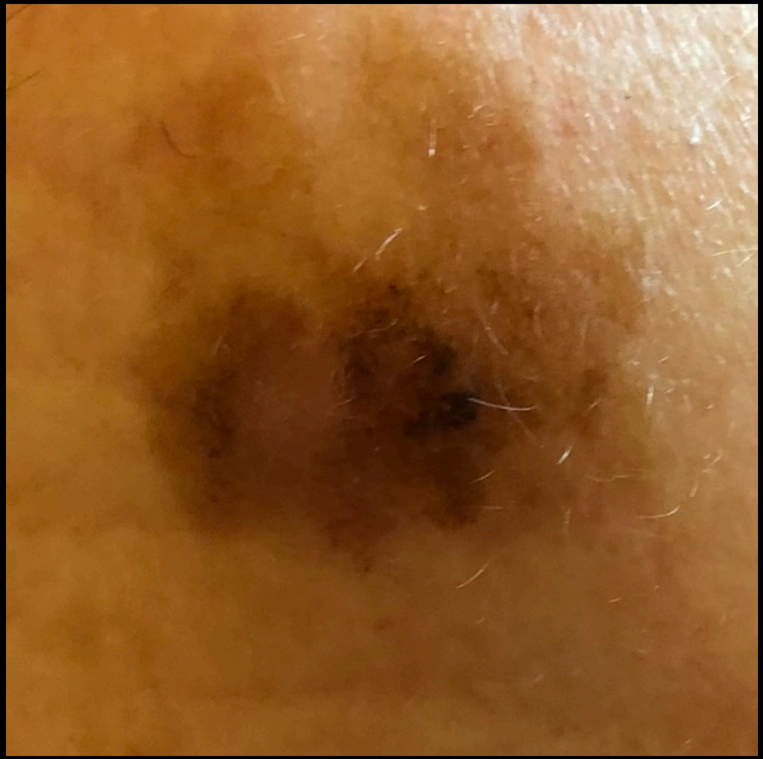

pT2a, 1.6 mm

Case number 206

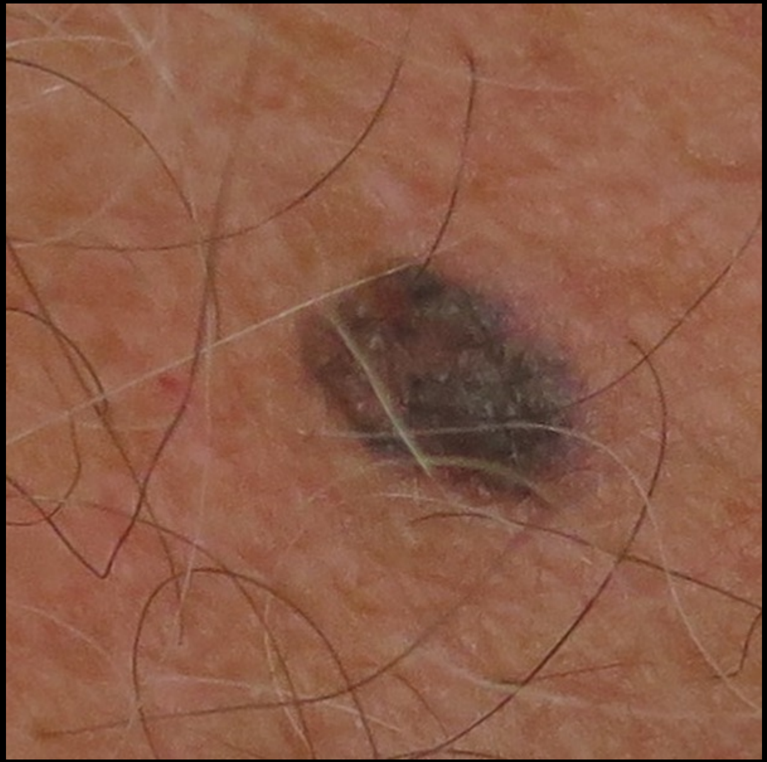

pT1a, 0.5 mm

Case number 207

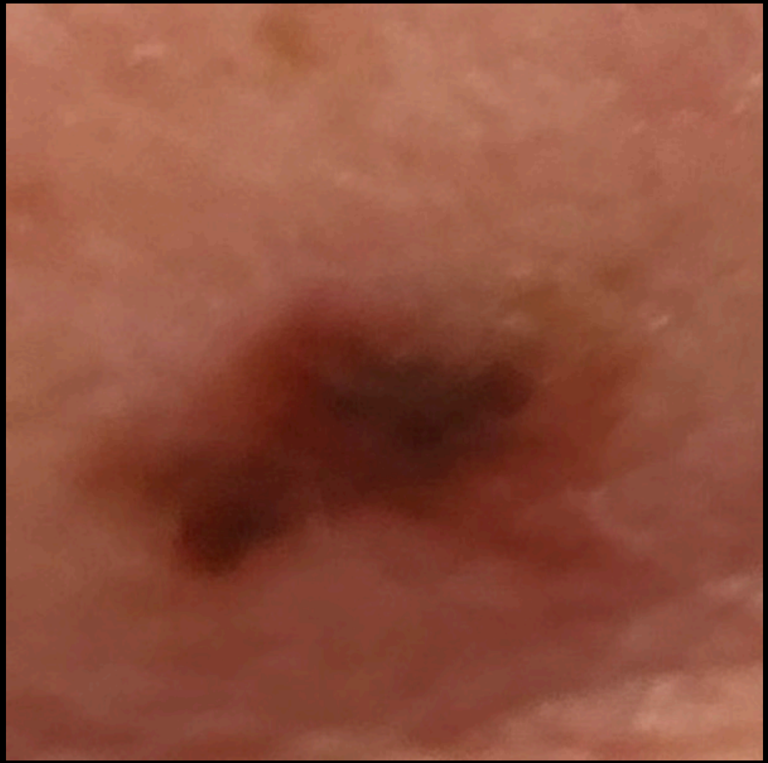

pT1a, 0.7 mm

Case number 208

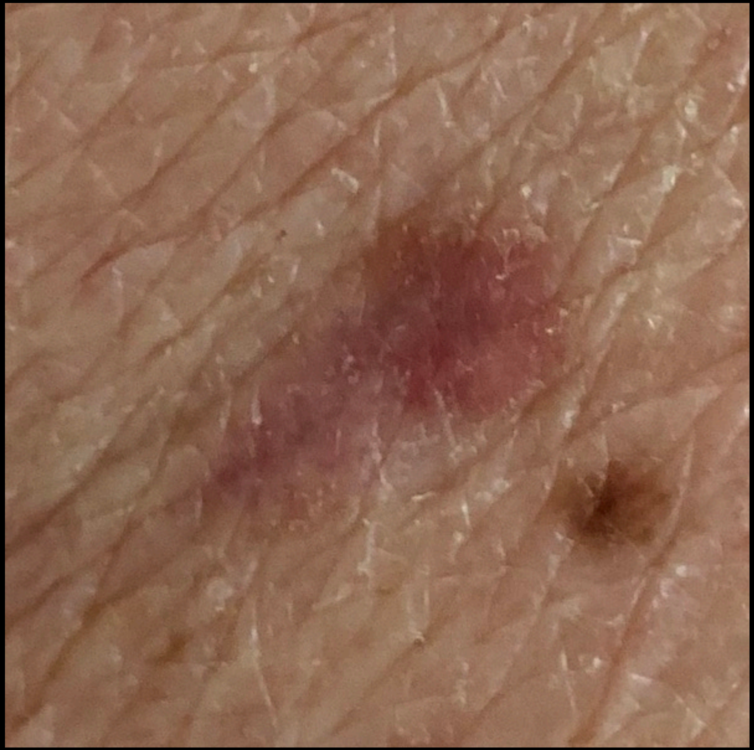

pTis

Case number 209

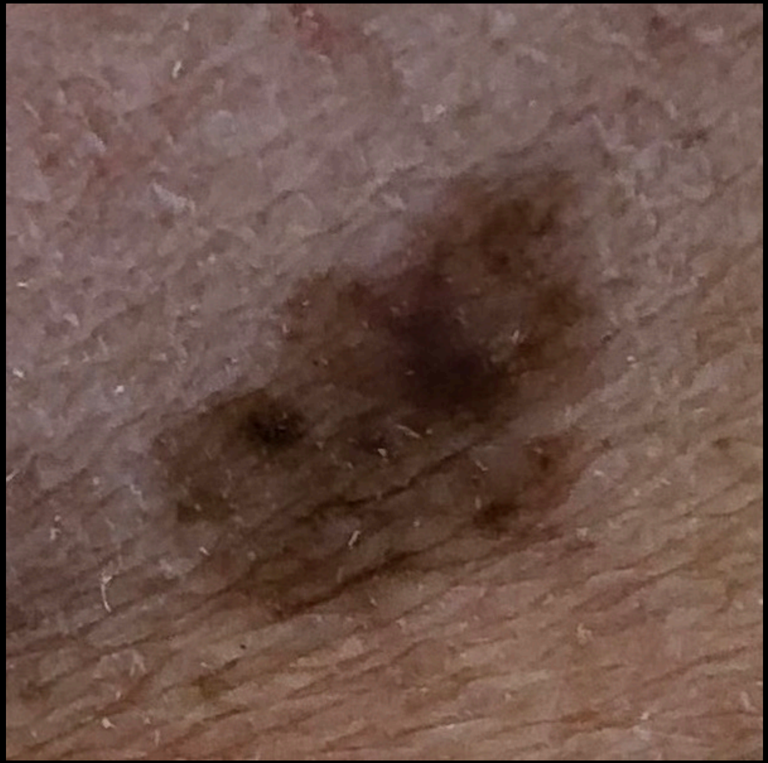

pT1a, 0.5 mm

Case number 210

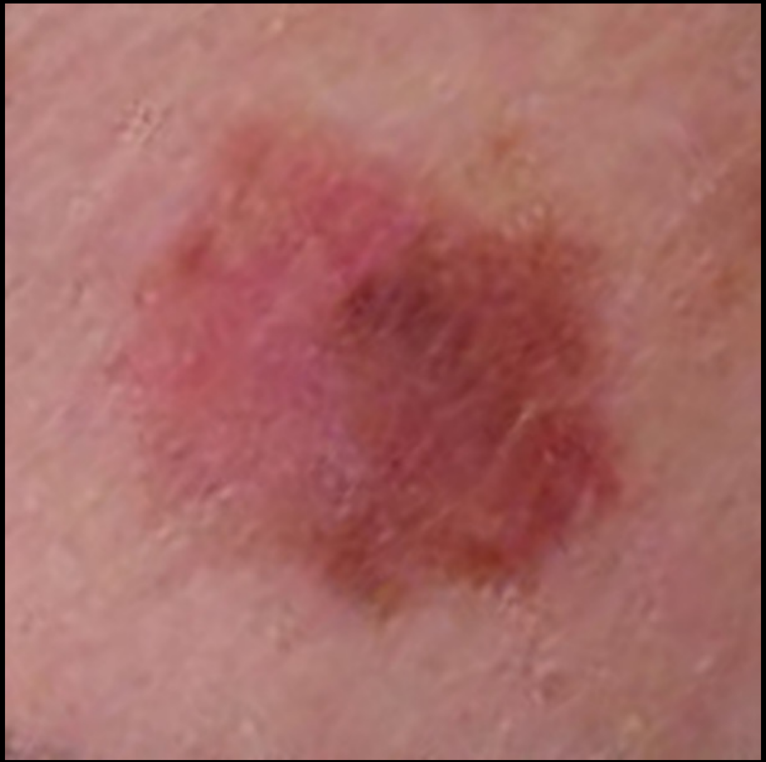

pTis

Case number 211

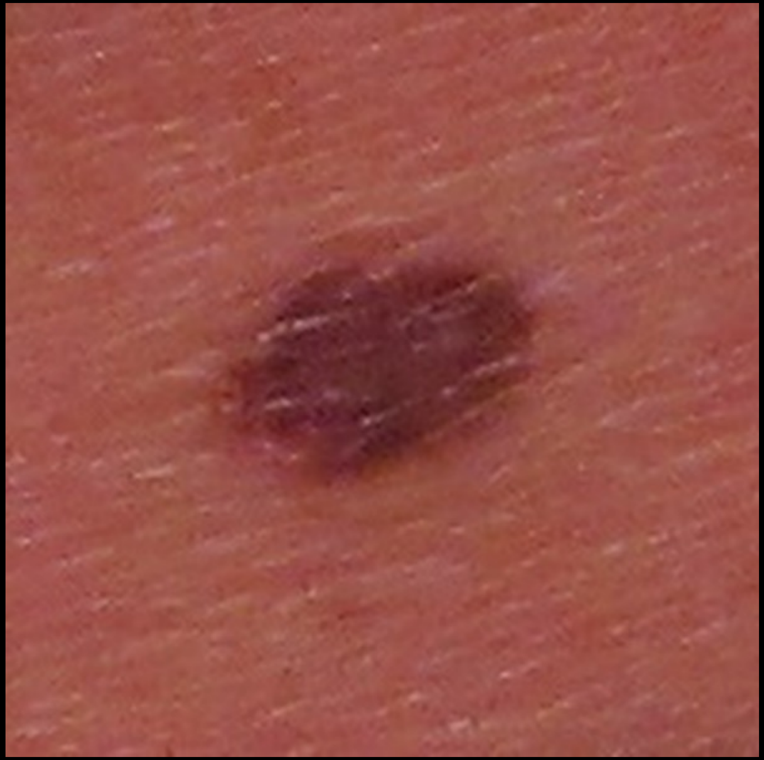

pTis

Case number 212

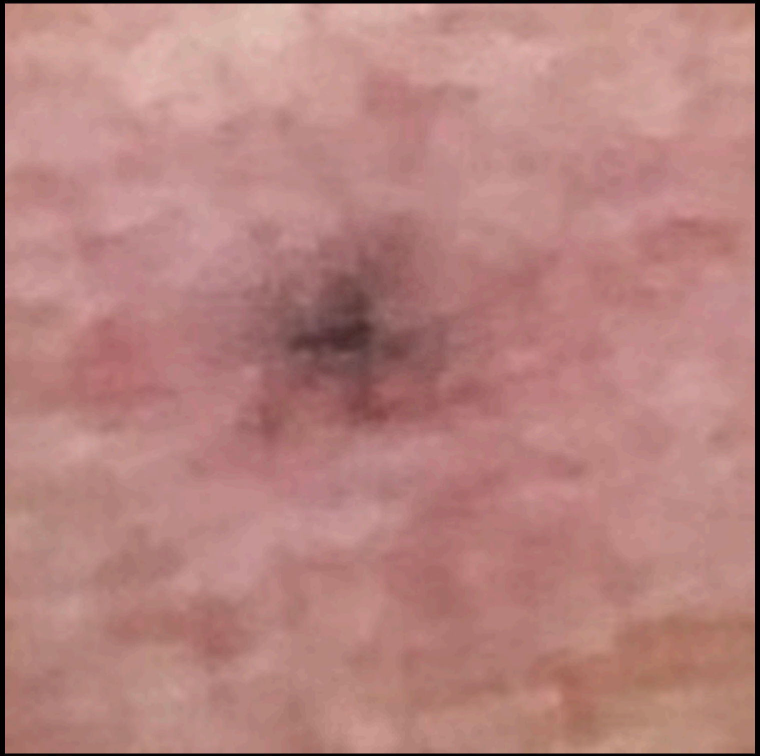

pTis

Case number 213

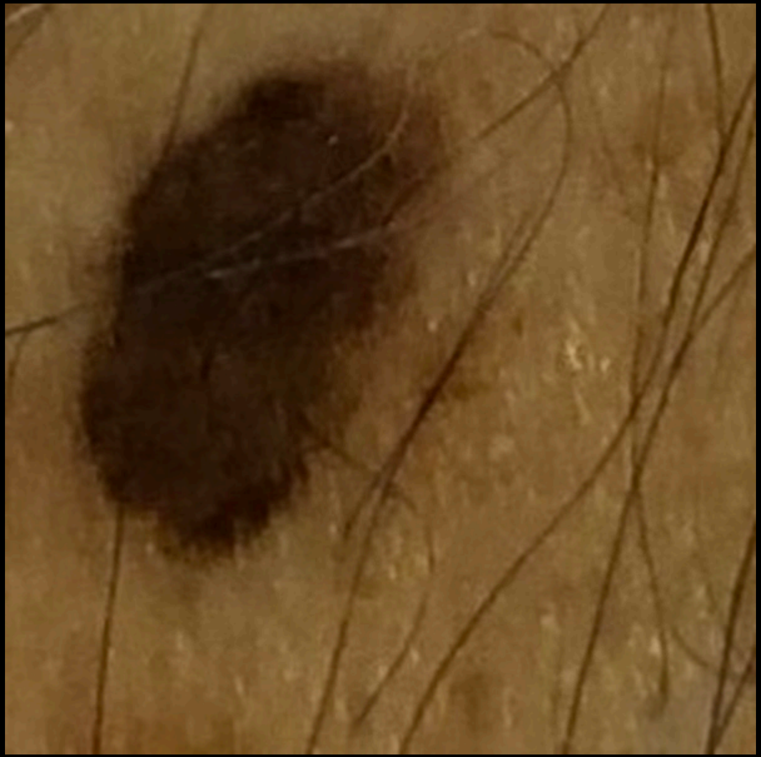

pTis

Case number 214

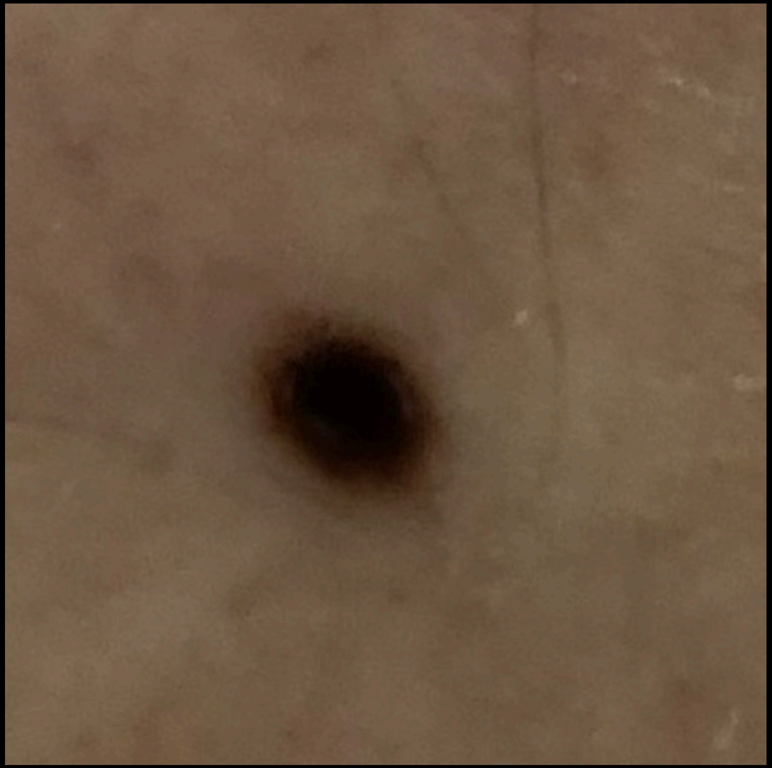

pTis

Case number 215

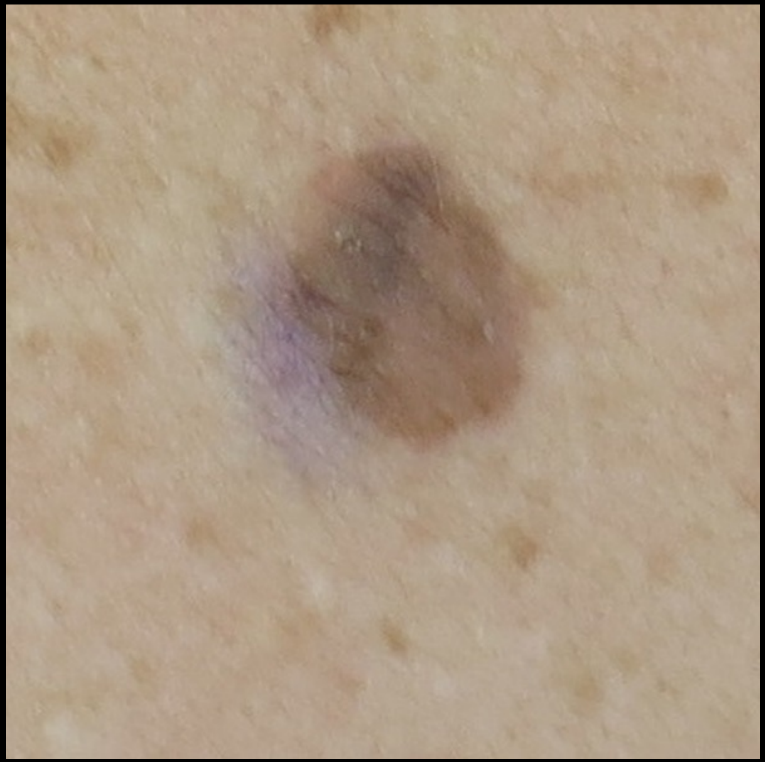

pTis

Case number 216

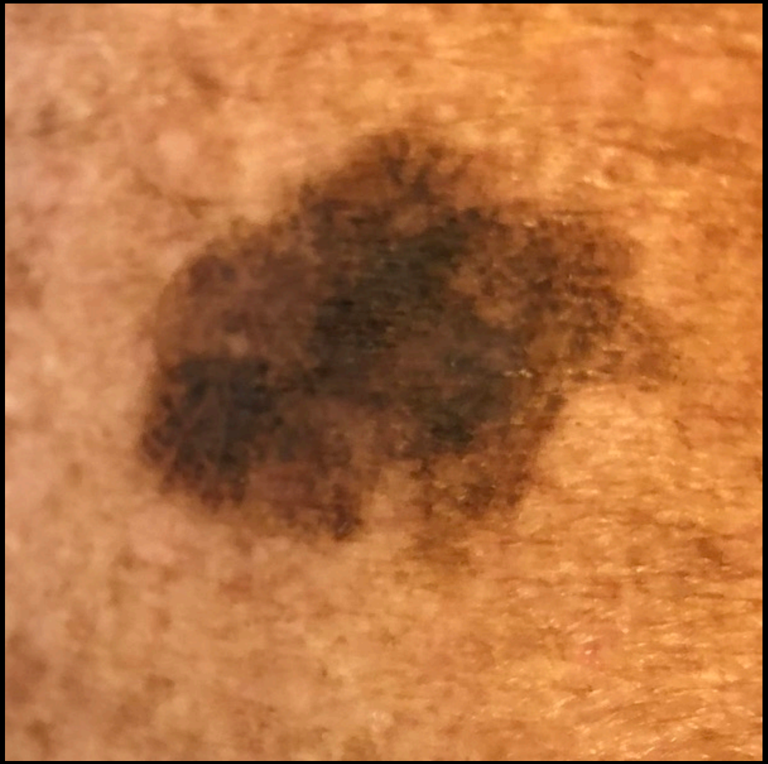

pT1a, 0.3 mm

Case number 217

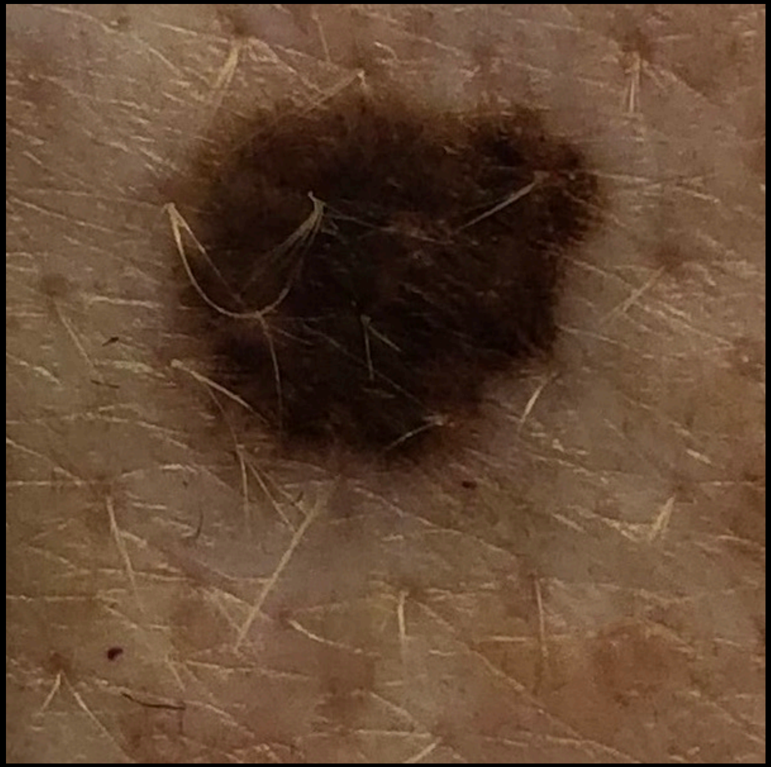

pTis

Case number 218

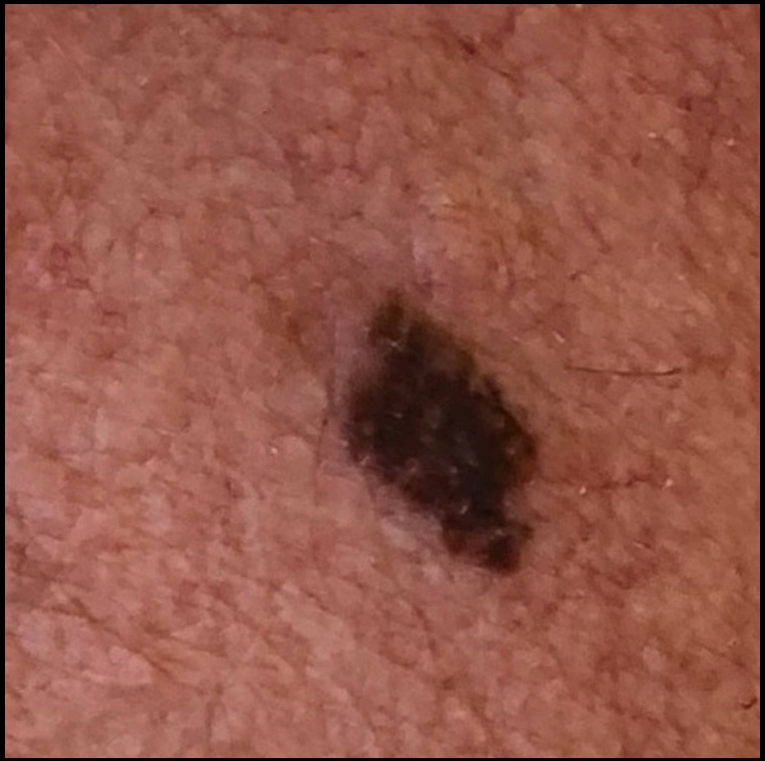

pTis

Case number 219

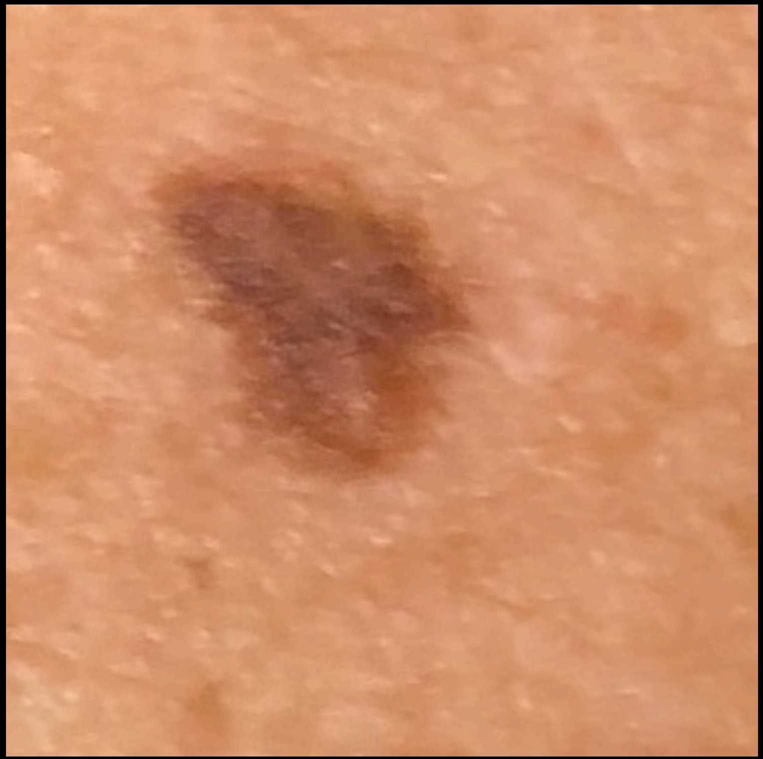

pTis

Case number 220

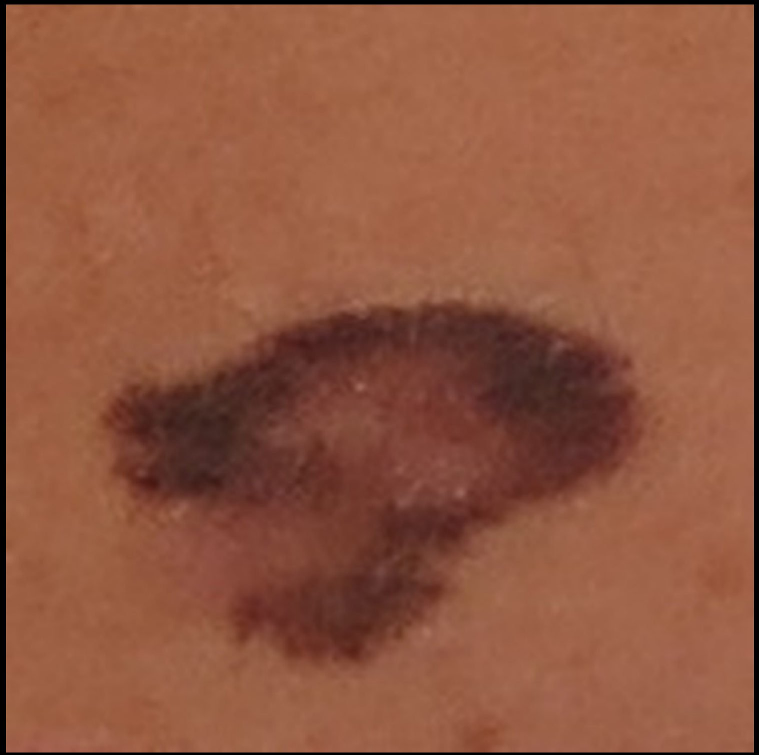

pTis

Case number 221

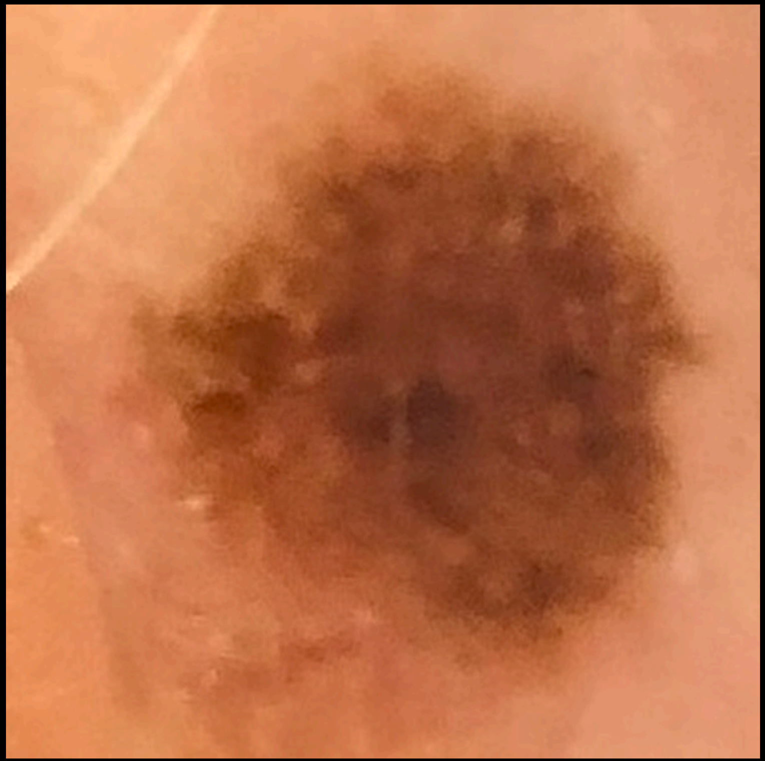

pTis

Case number 222

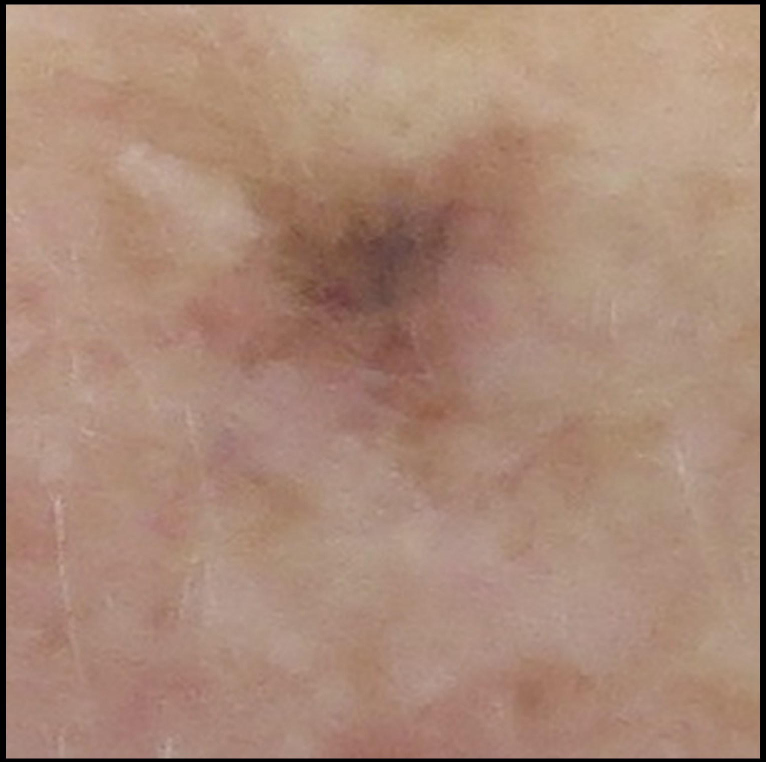

pT1a, 0.5 mm

Case number 223

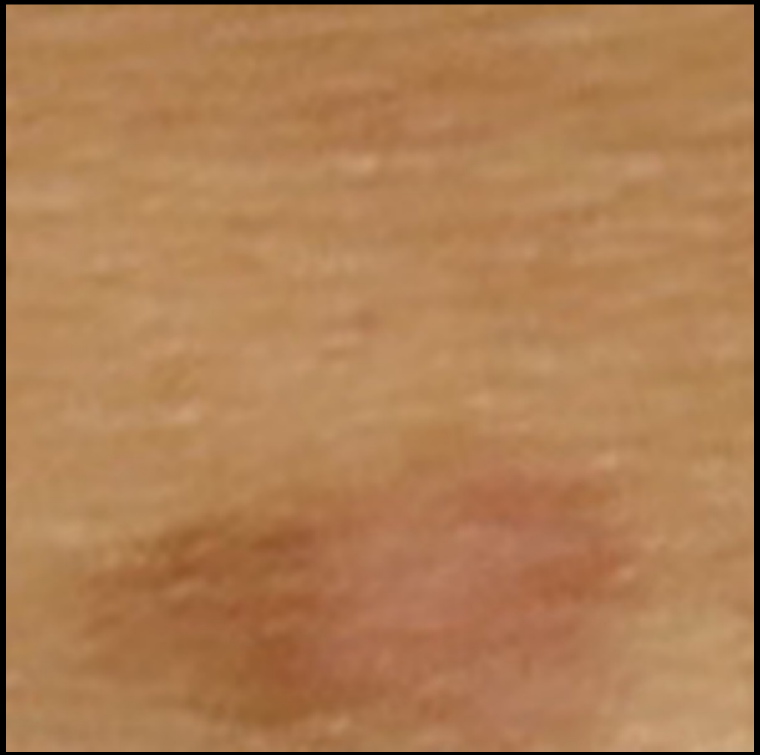

pTis

Case number 224

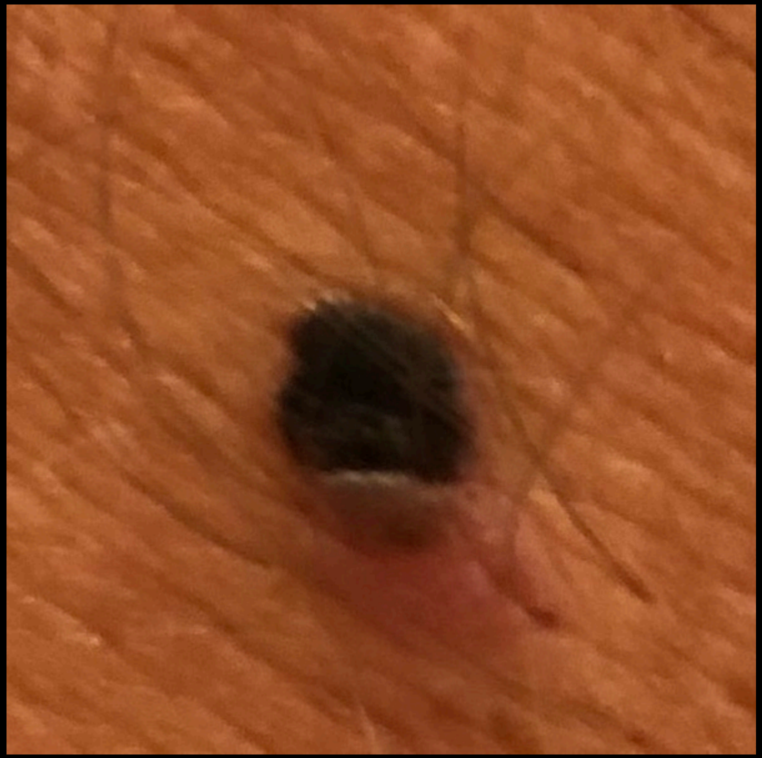

pT1a, 0.7 mm

Case number 225

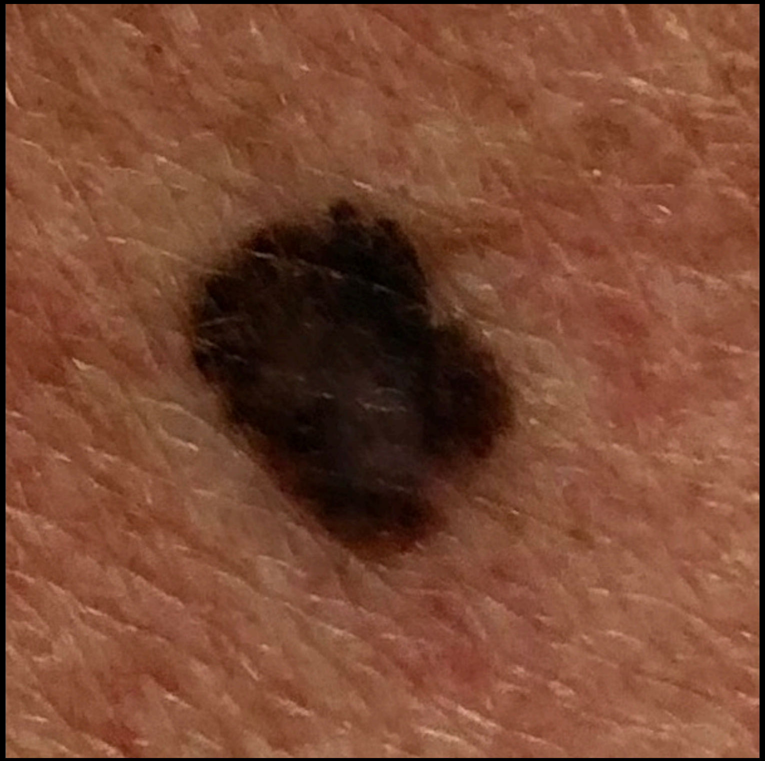

pT1a, 0.8 mm

Case number 226

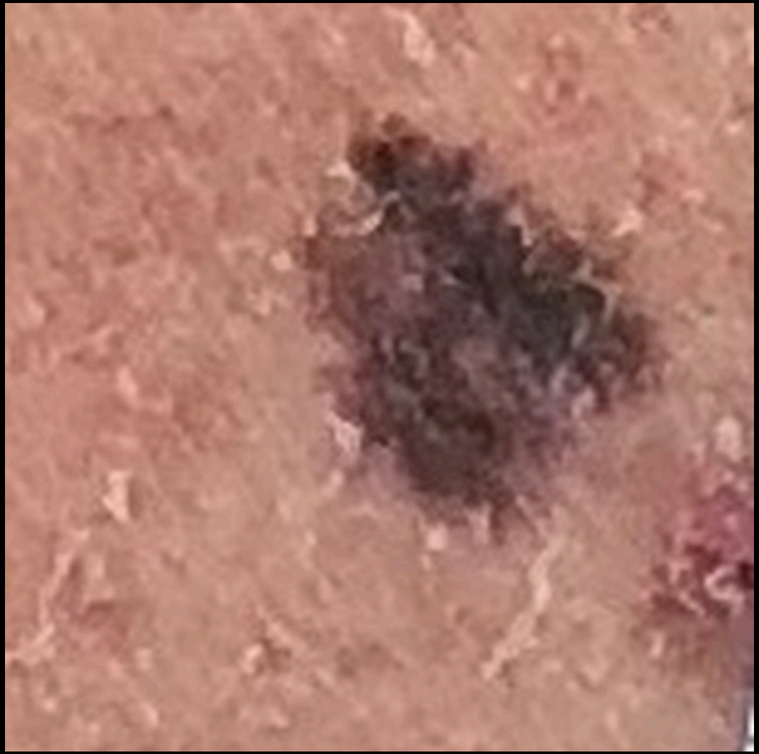

pT1a, 0.3 mm

Case number 227

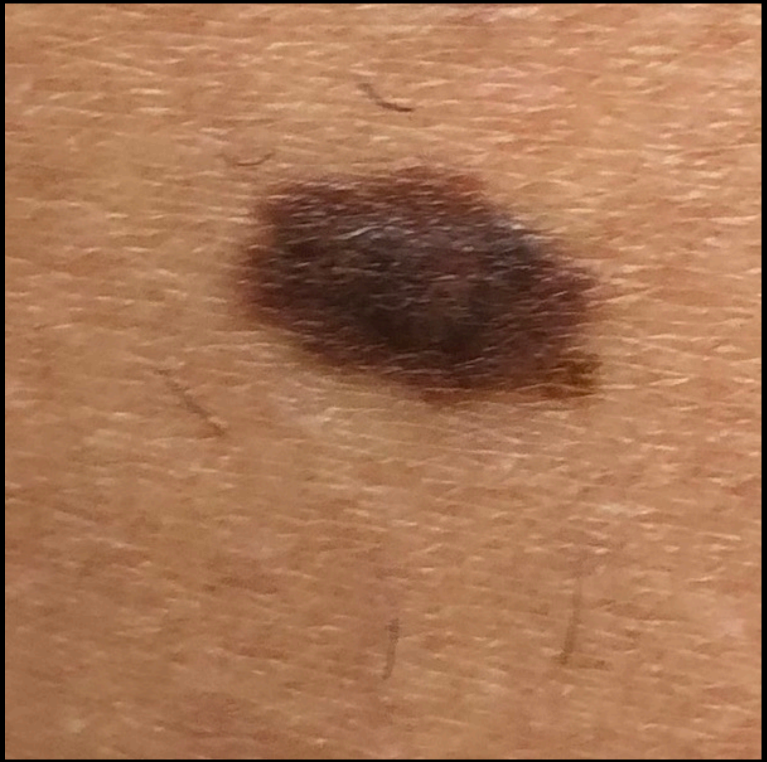

pTis

Case number 228

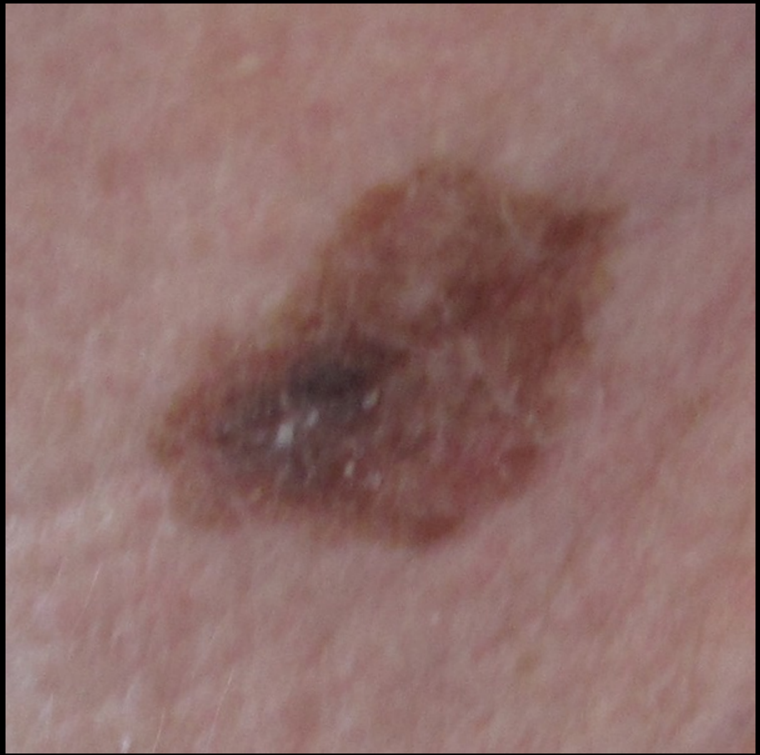

pTis

Case number 229

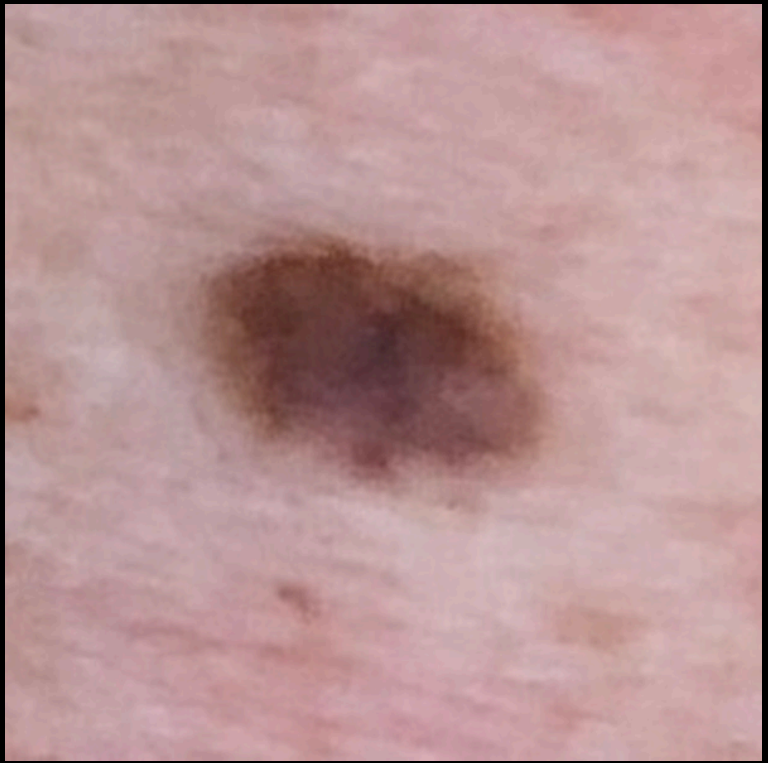

pTis

Case number 230

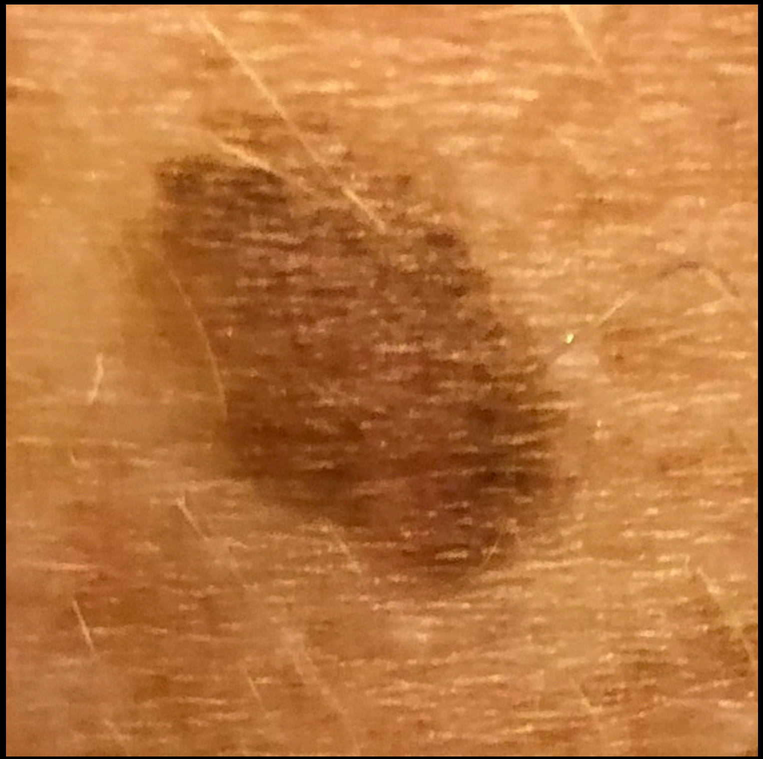

pTis

Case number 231

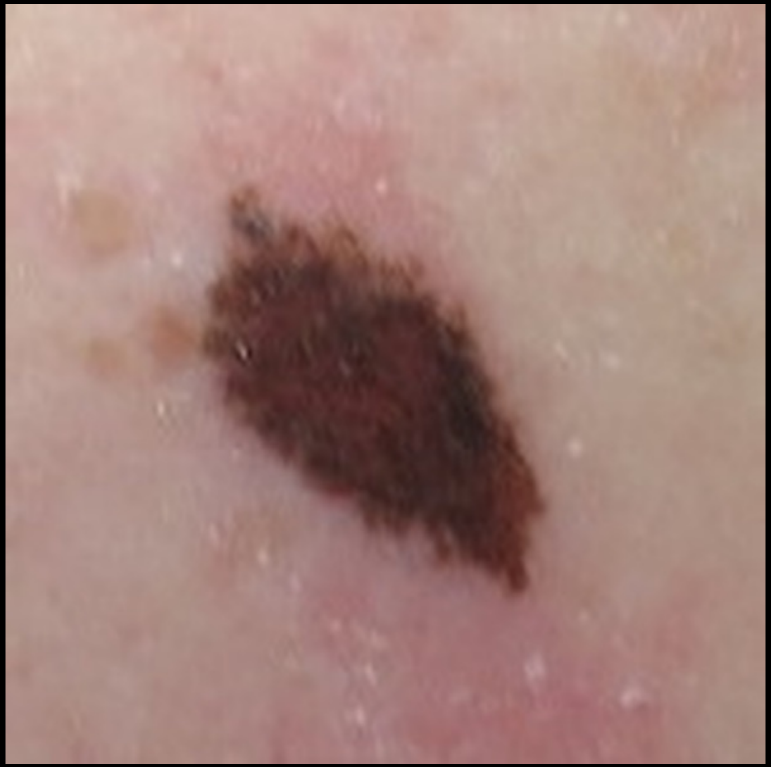

pTis

Case number 232

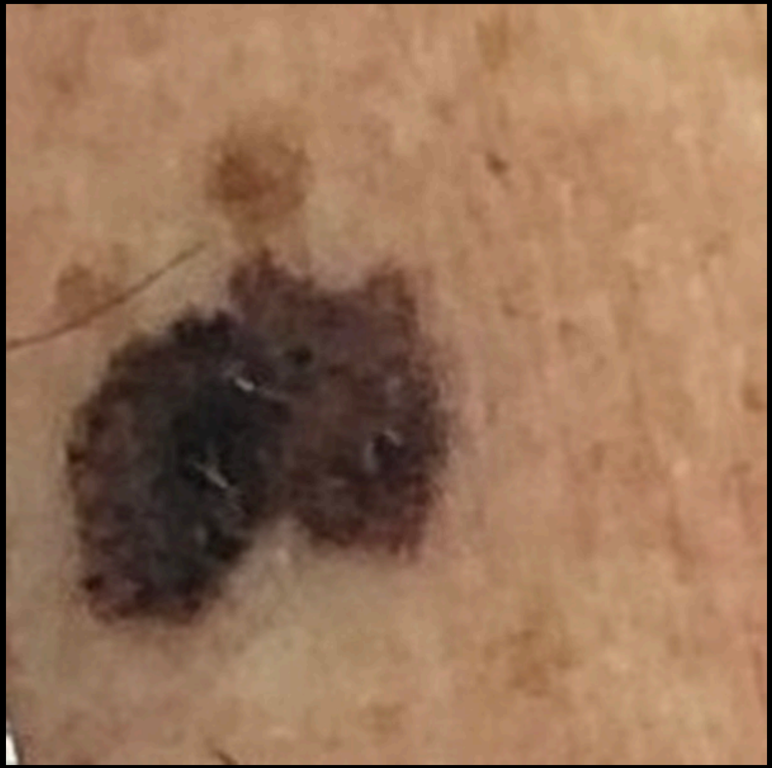

pTis

Case number 233

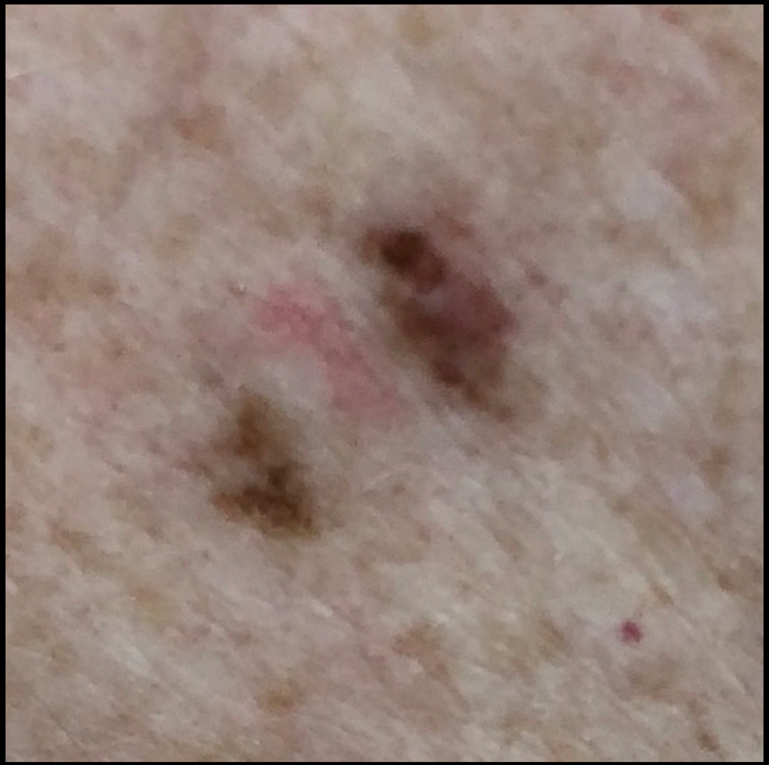

pTis

Case number 234

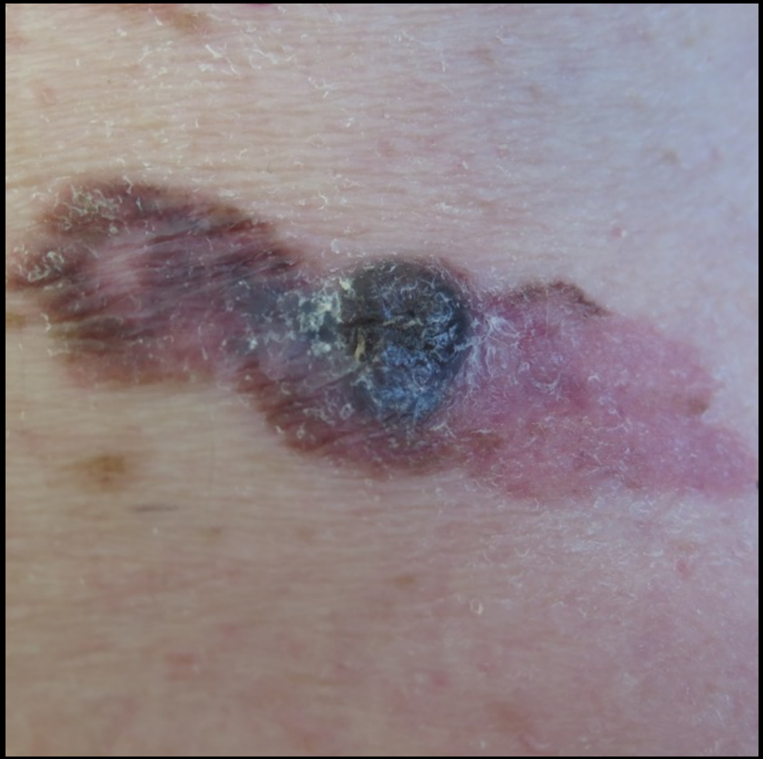

pT4b, 5.3 mm

Case number 235

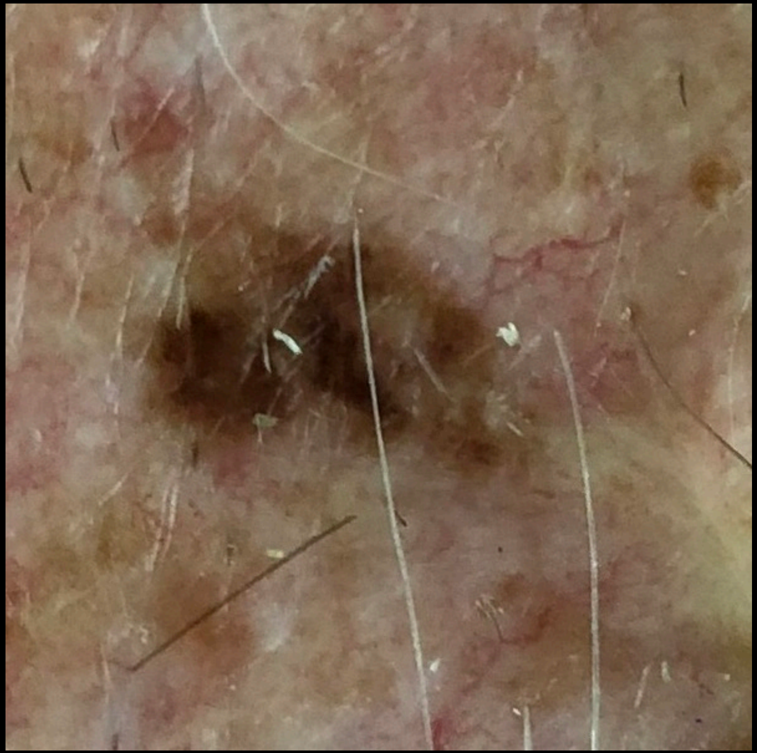

pTis

Case number 236

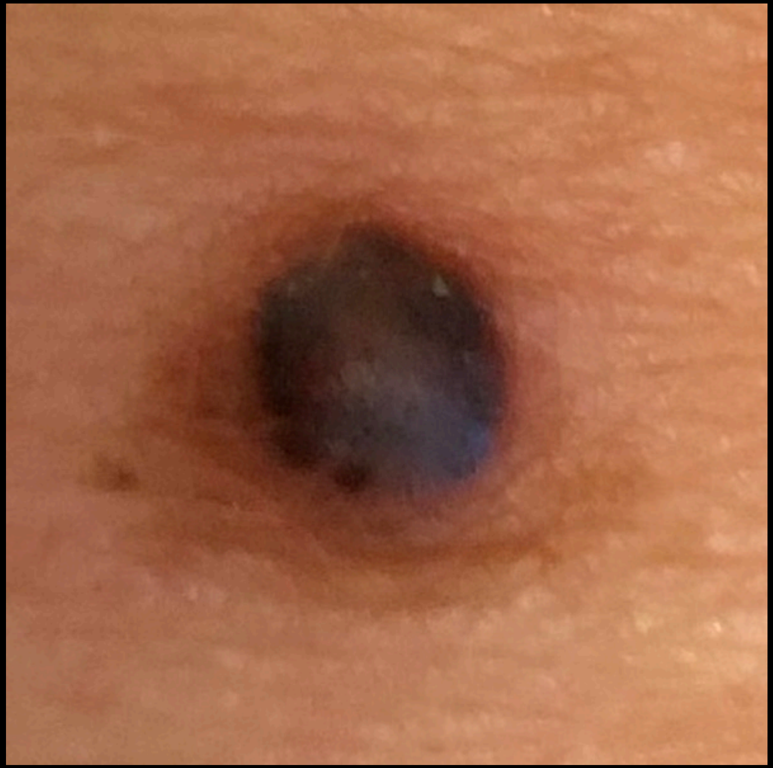

pT1a, 0.8 mm

Case number 237

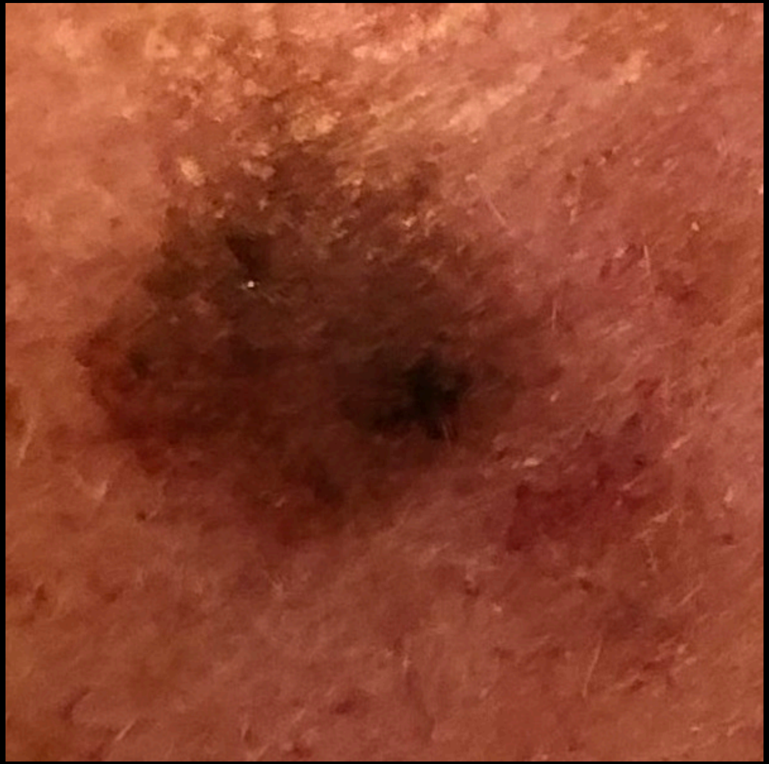

pT1a, 0.3 mm

Case number 238

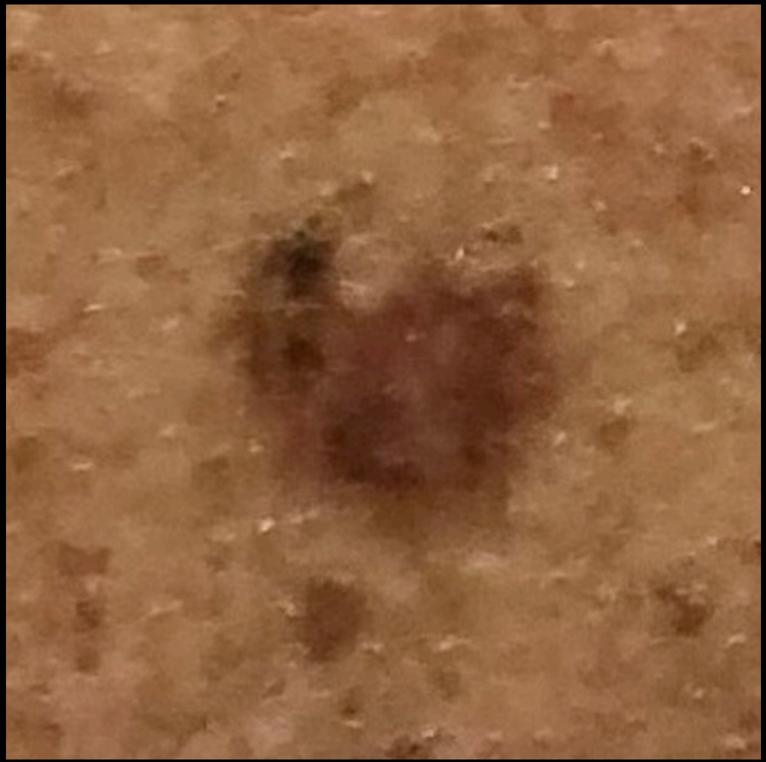

pTis

Case number 239

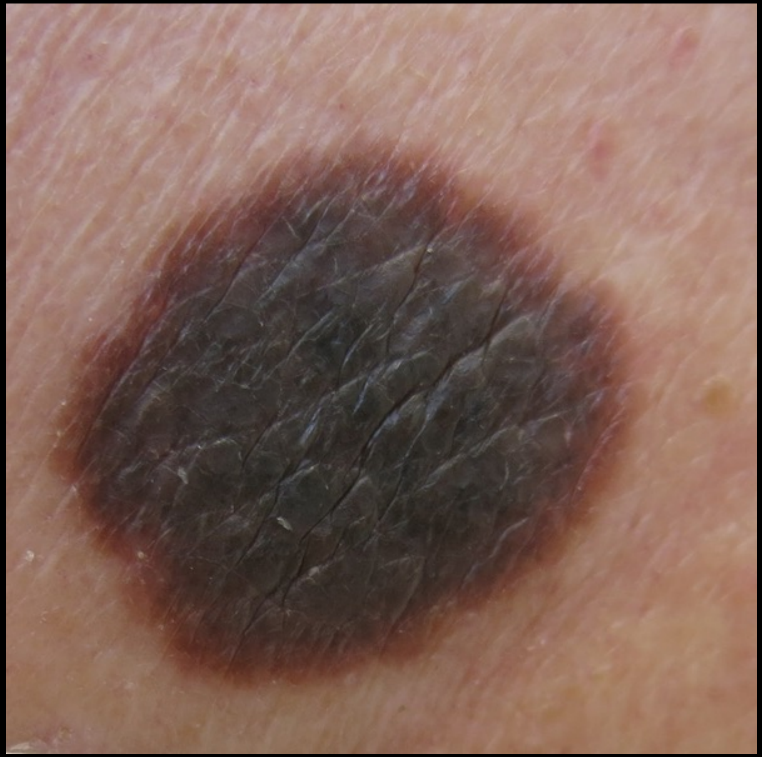

pT1b, 0.7 mm

Case number 240

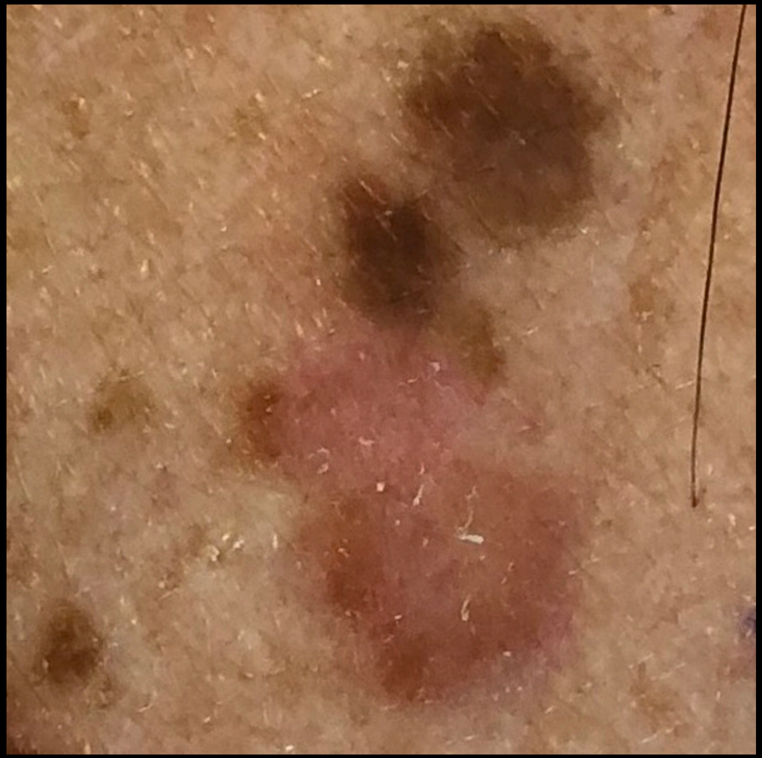

pTis

Case number 241

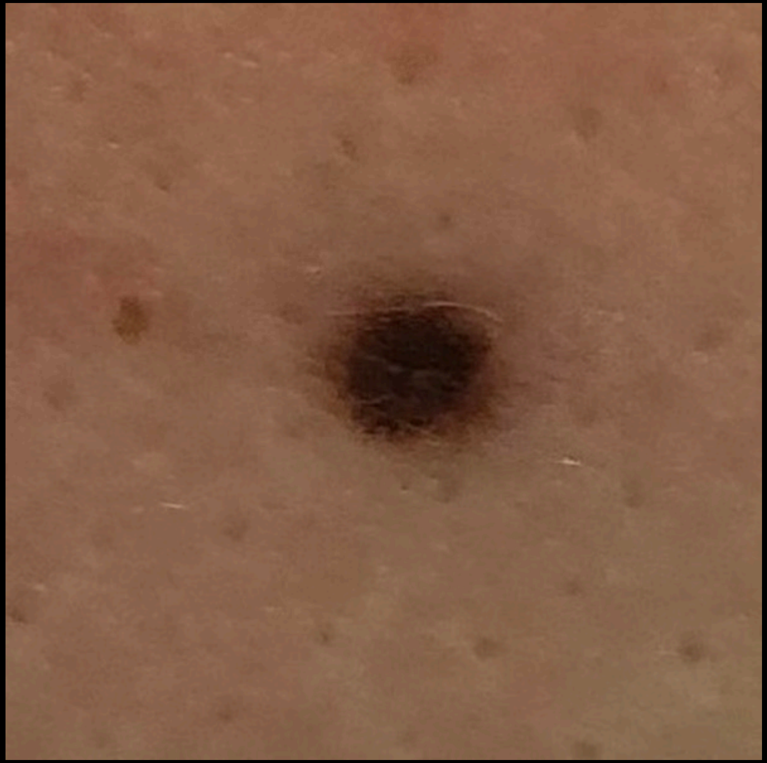

pTis

Case number 242

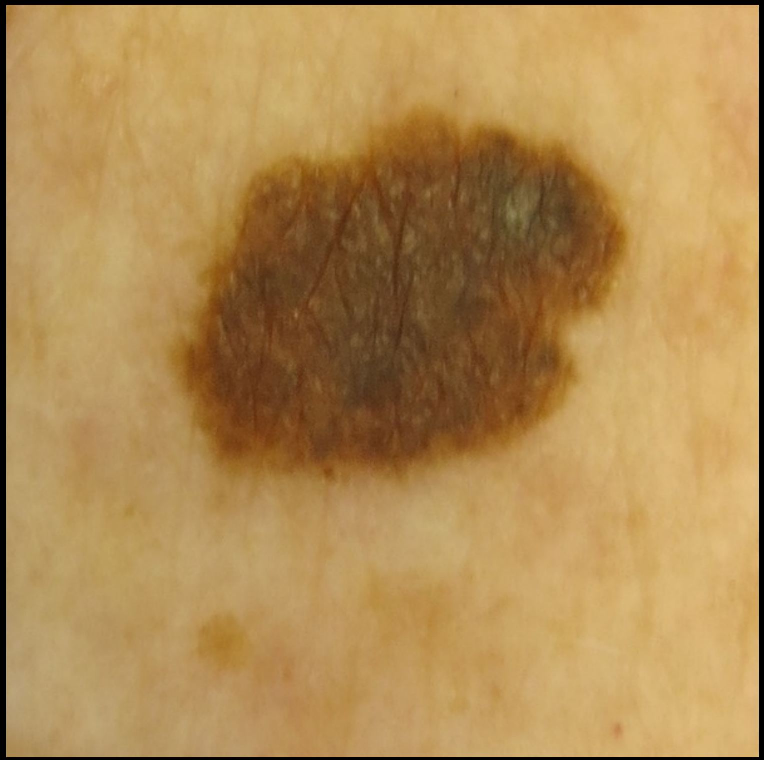

pTis

Case number 243

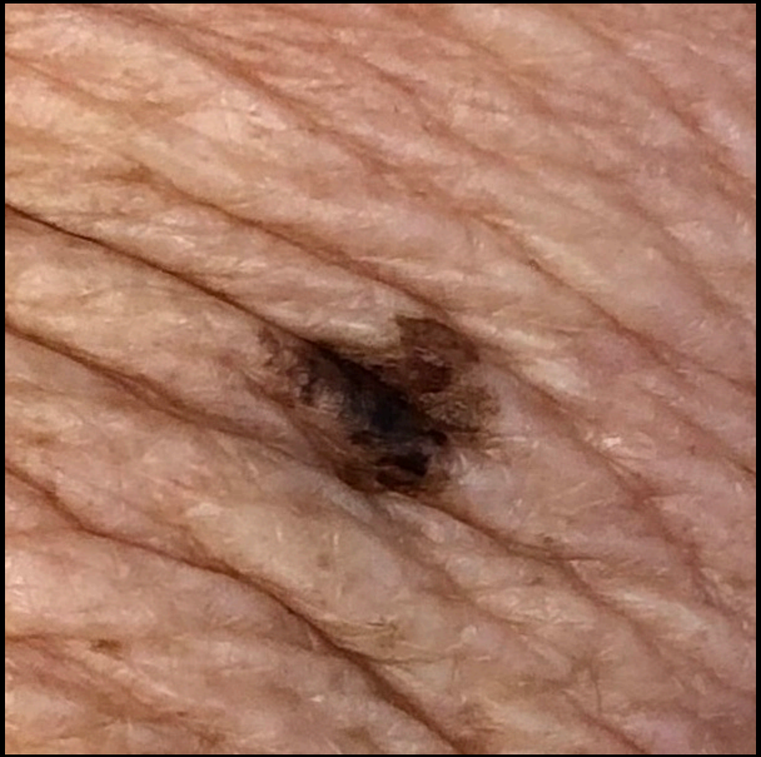

pT1a, 0.6 mm

Case number 244

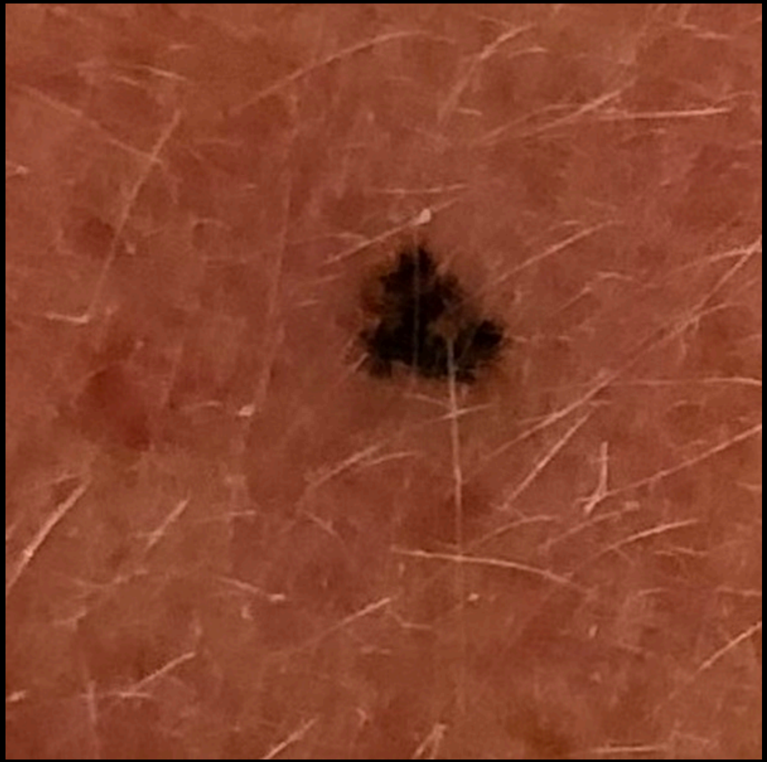

pT1a, 0.3 mm

Case number 245

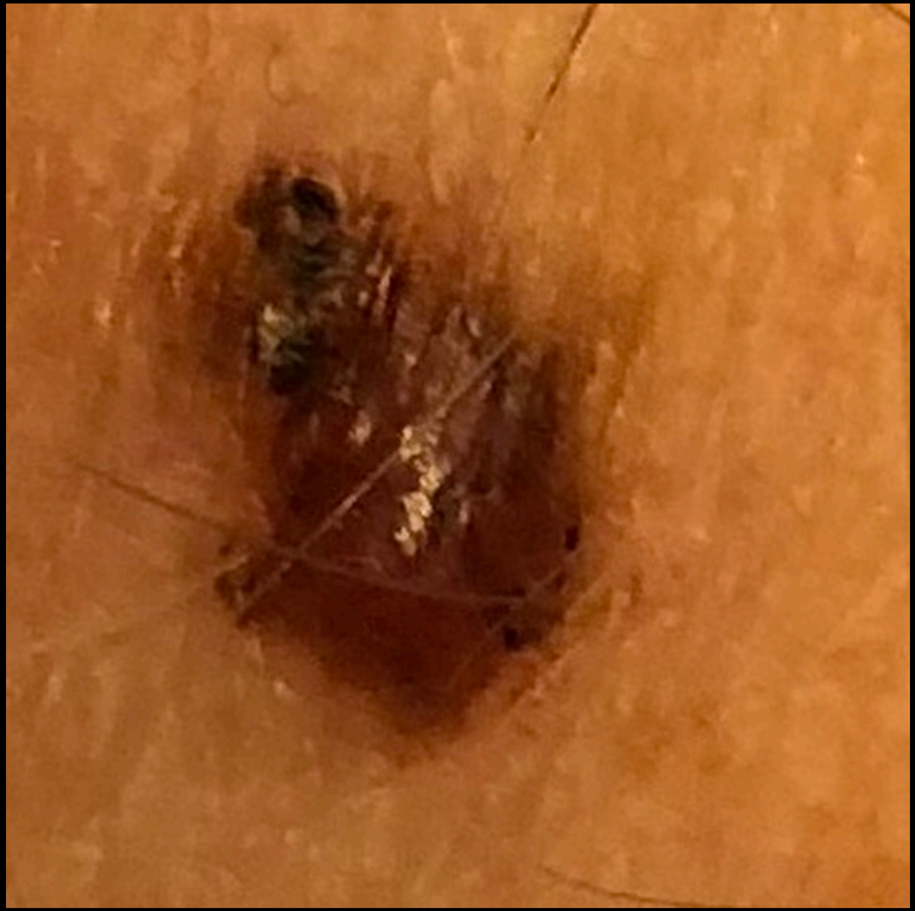

pT1a, 0.7 mm

Case number 246

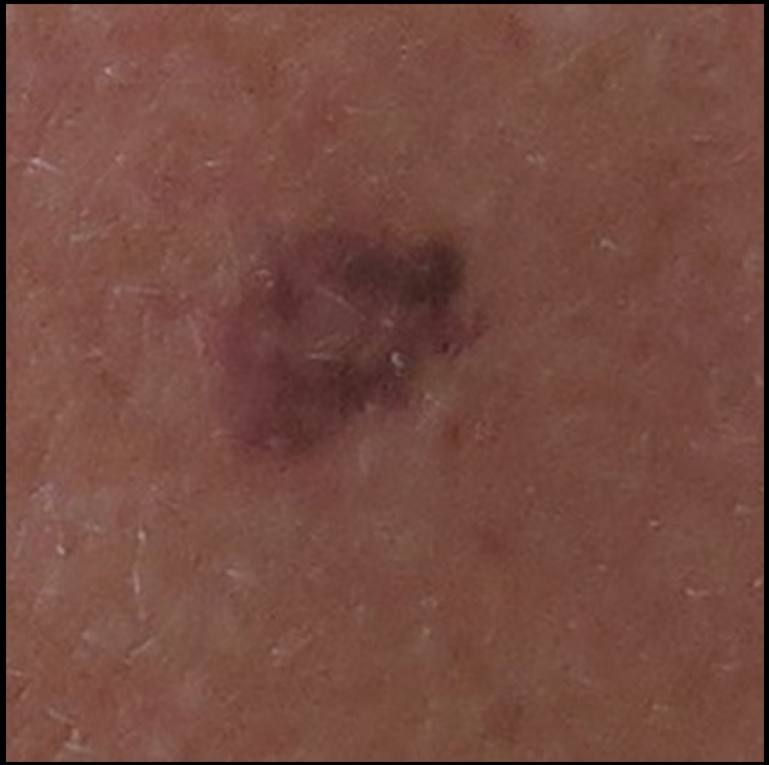

pTis

Case number 247

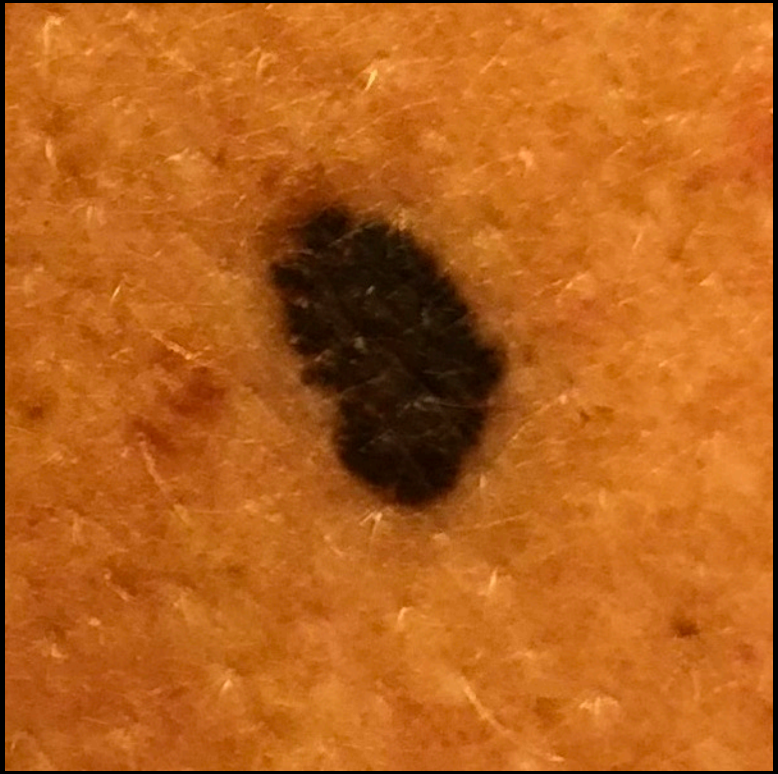

pTis

Case number 248

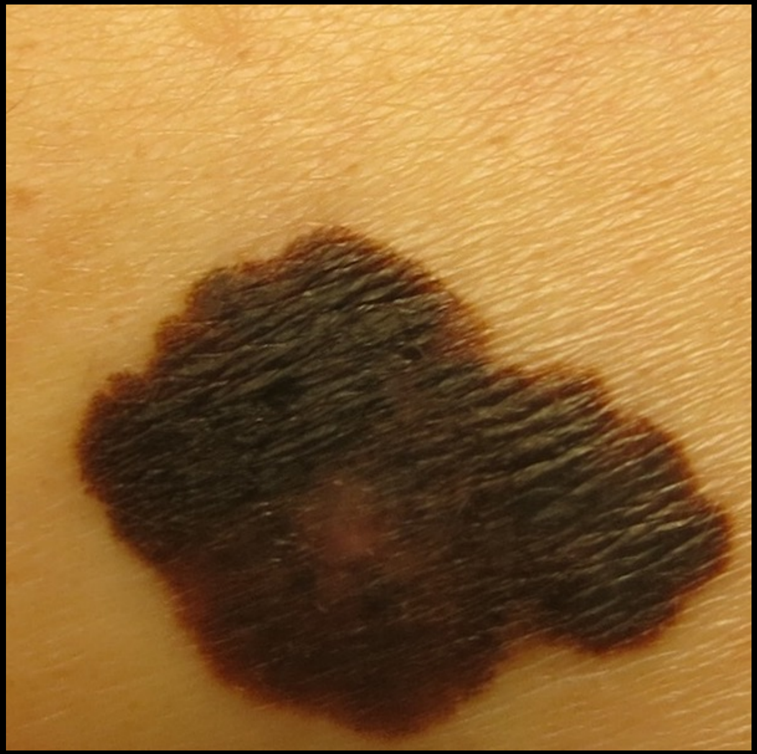

pT1a, 0.6 mm

Case number 249

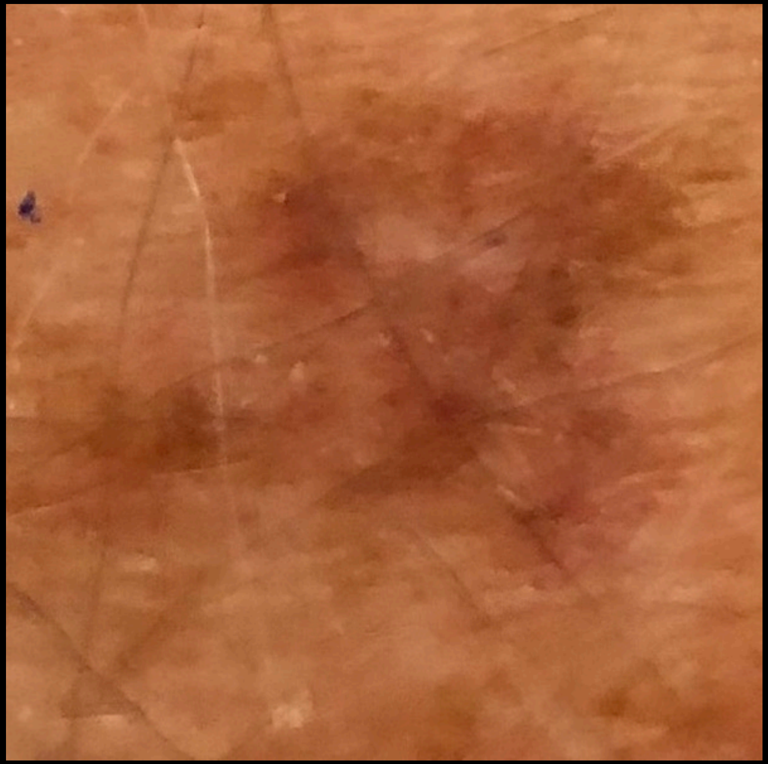

pT1a, 0.2 mm

Case number 250

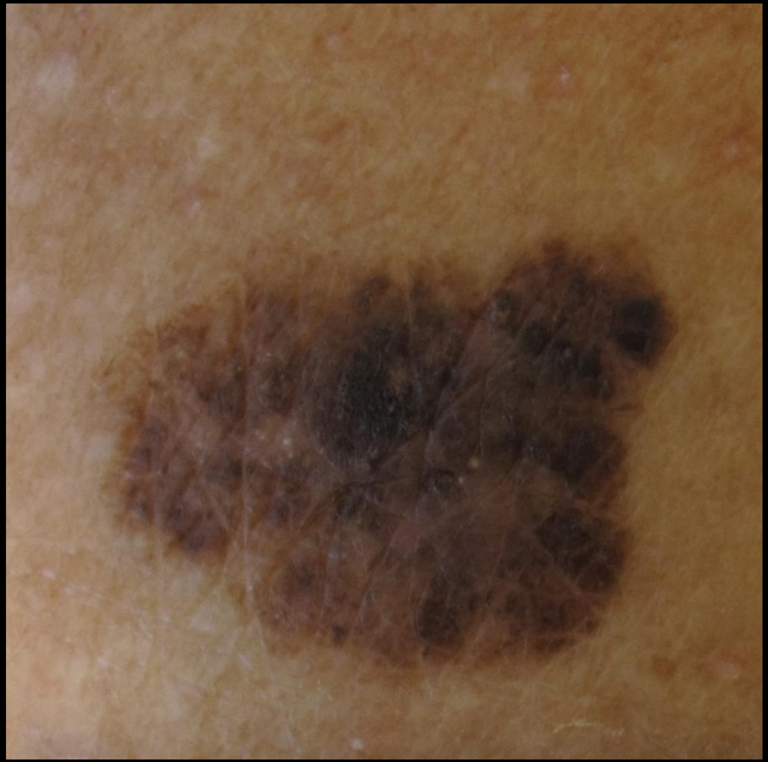

pT1a, 0.5 mm

Case number 251

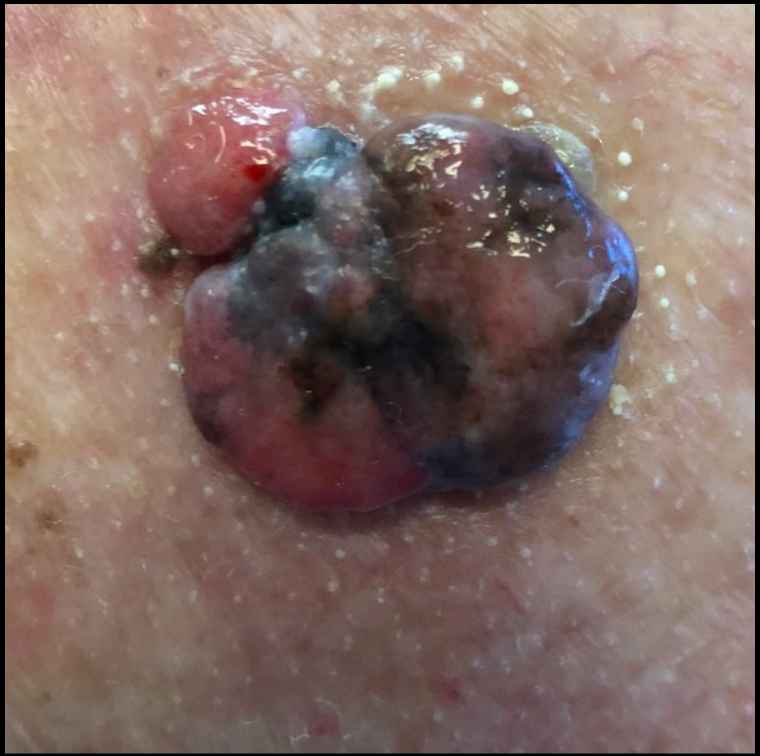

pT4b, 11 mm

Case number 252

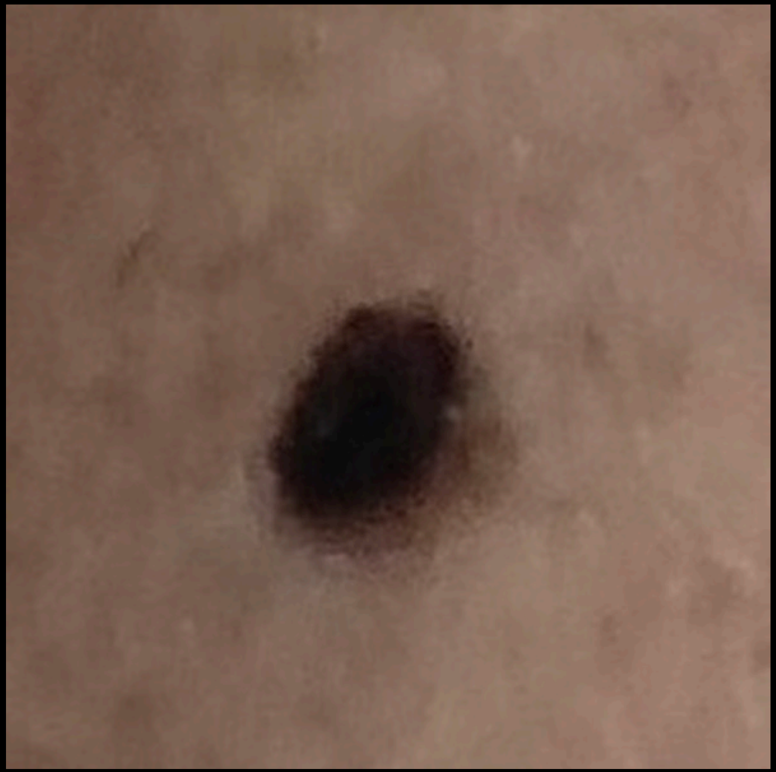

pT1a, 0.5 mm

Case number 253

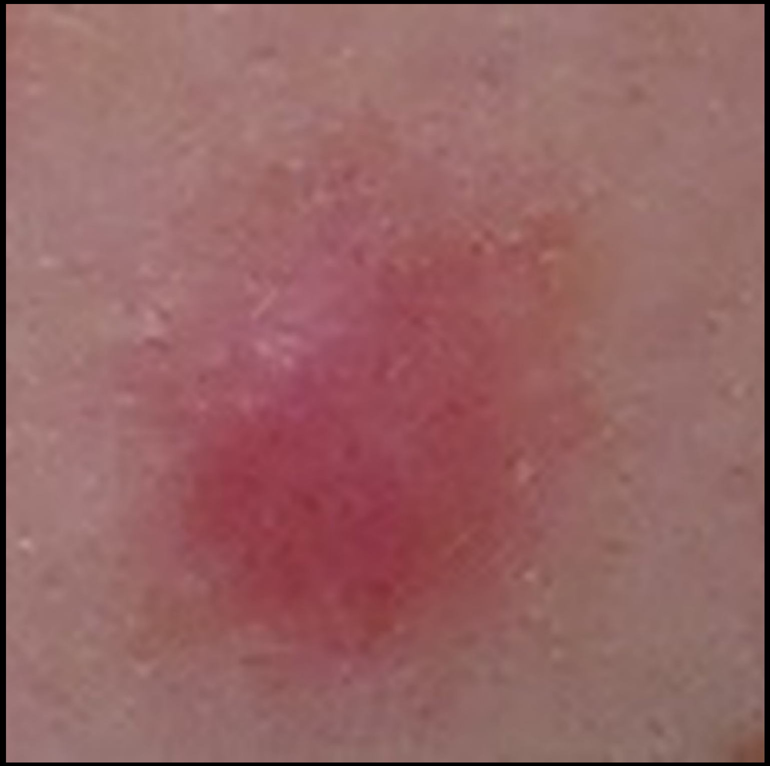

pT1b, 0.8 mm

Case number 254

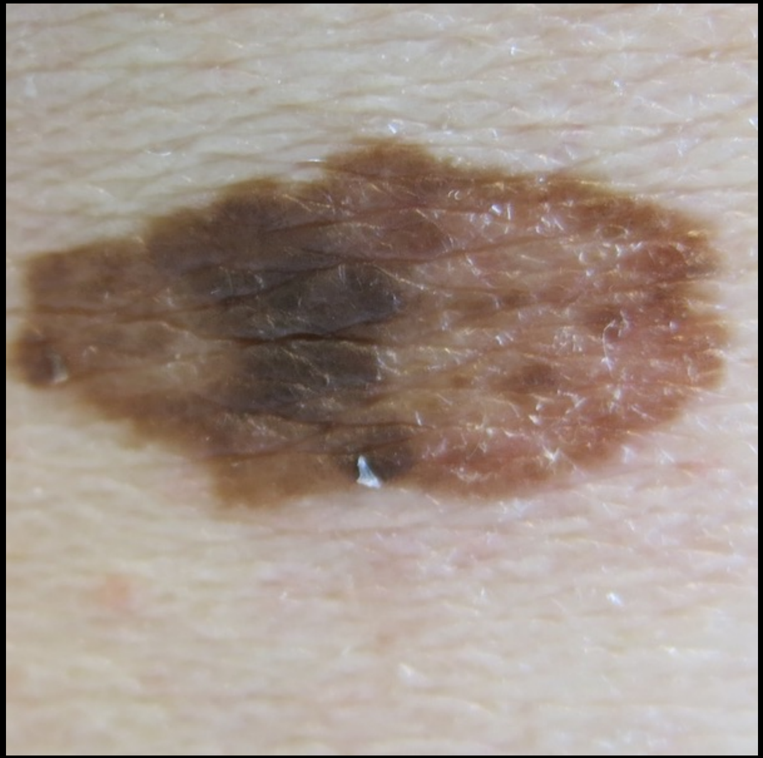

pTis

Case number 255

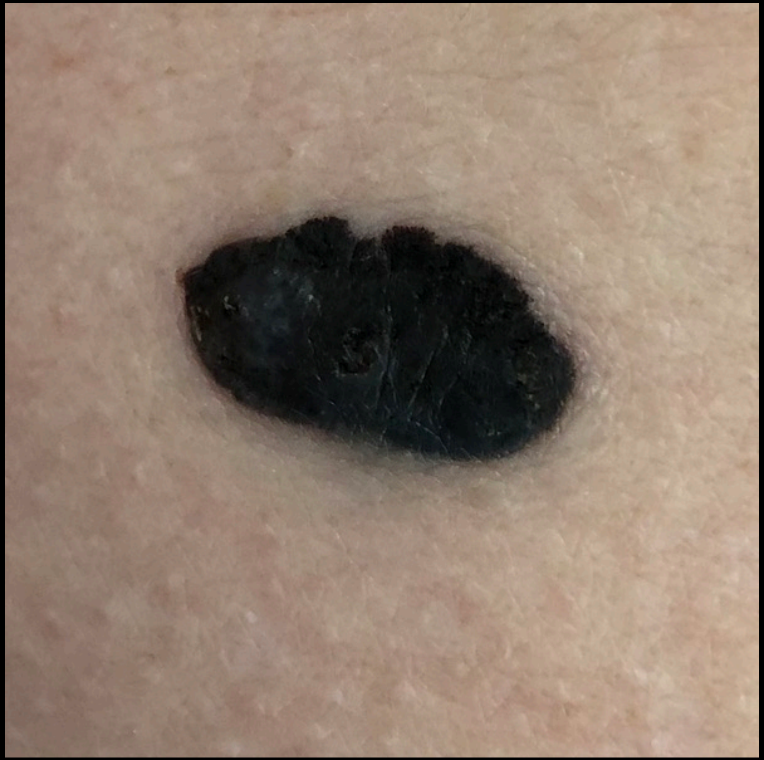

pT3b, 2.8 mm

Case number 256

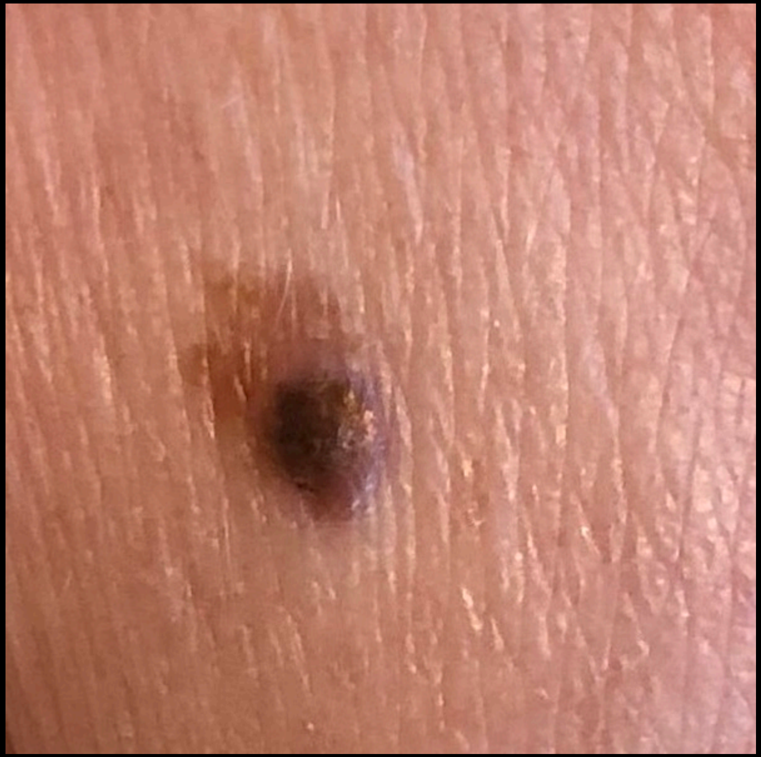

pT1b, 0.8 mm

Case number 257

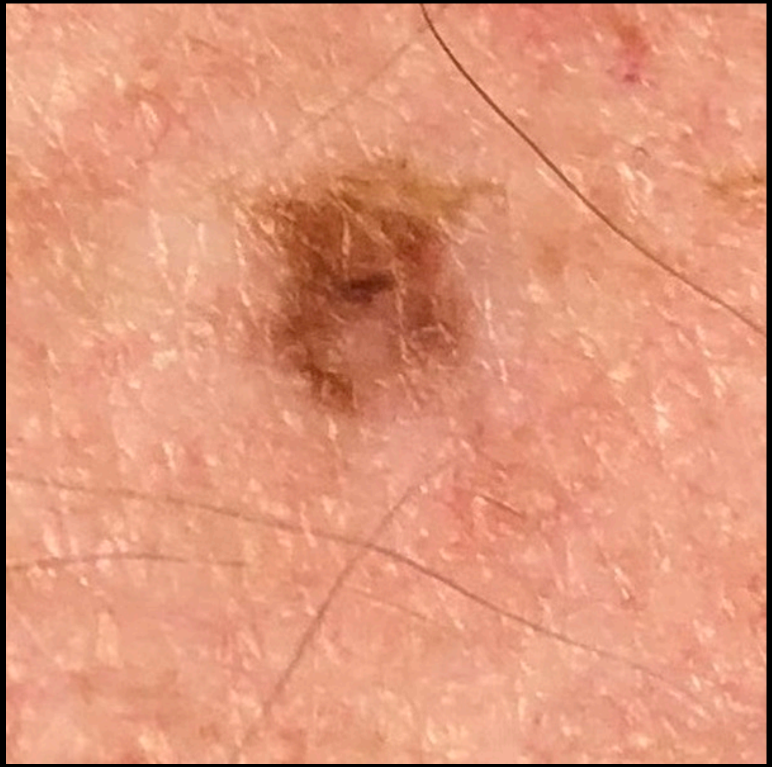

pTis

Case number 258

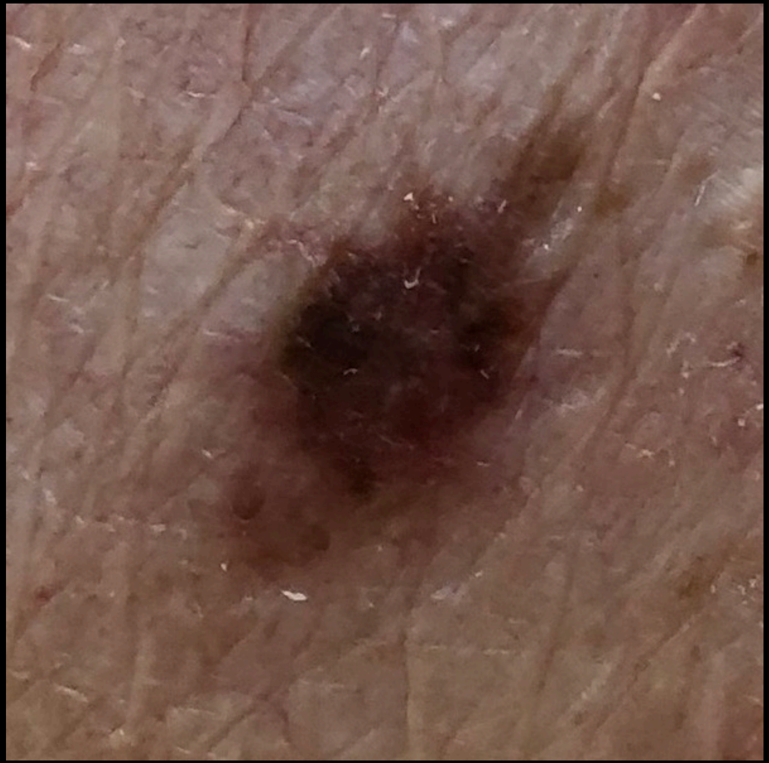

pT1a, 0.6 mm

Case number 259

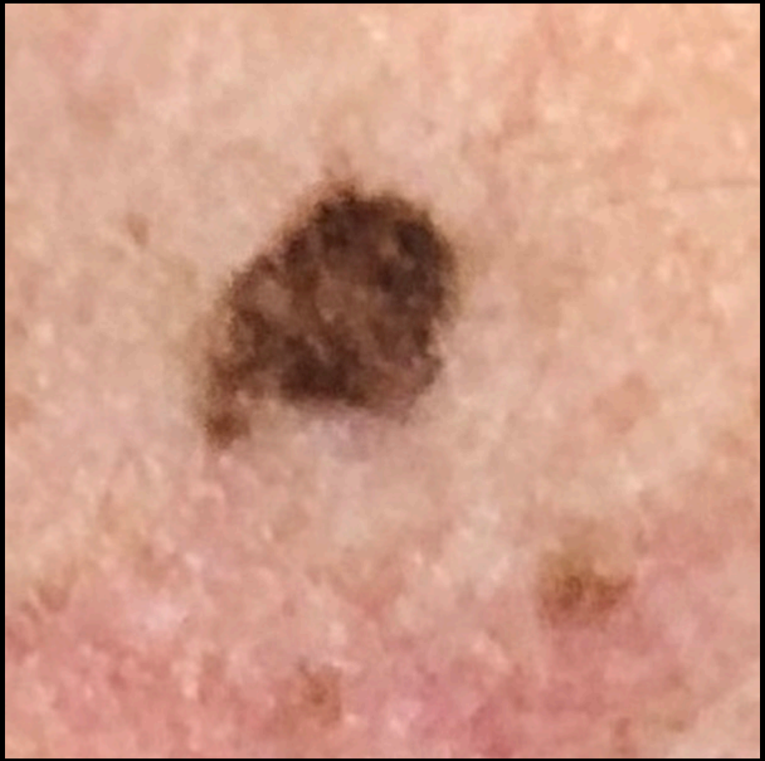

pTis

Case number 260

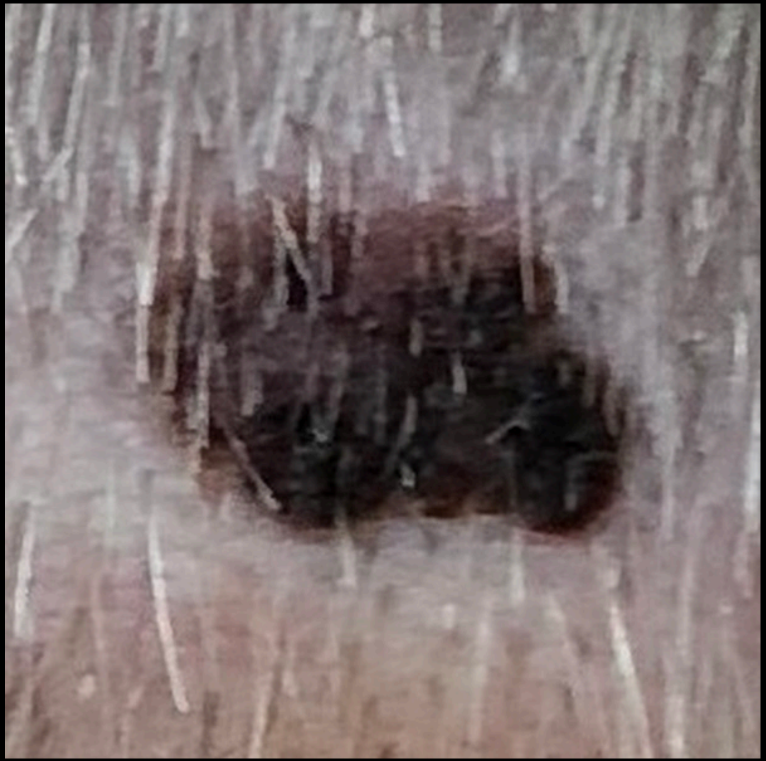

pTis

Case number 261

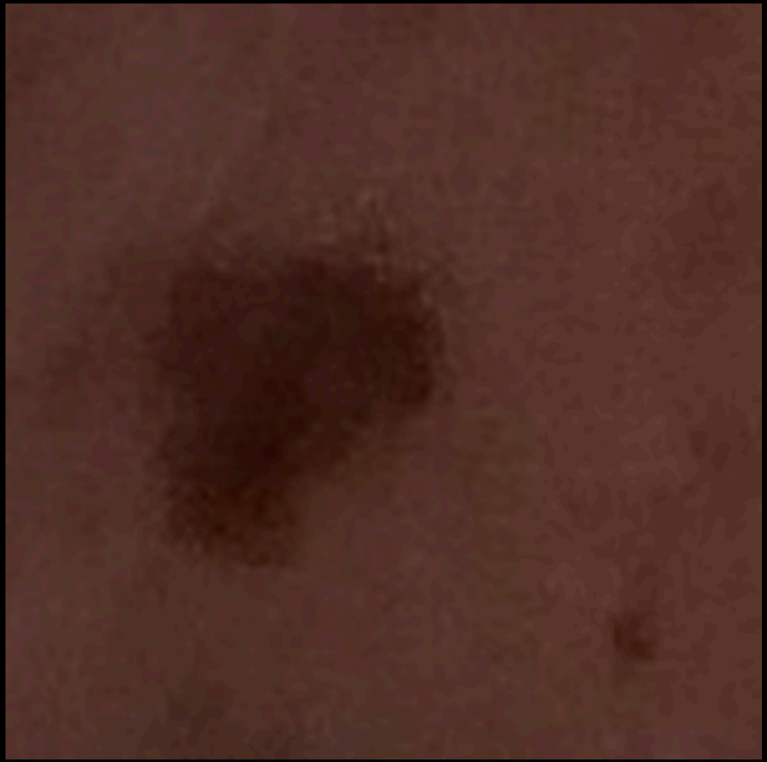

pTis

Case number 262

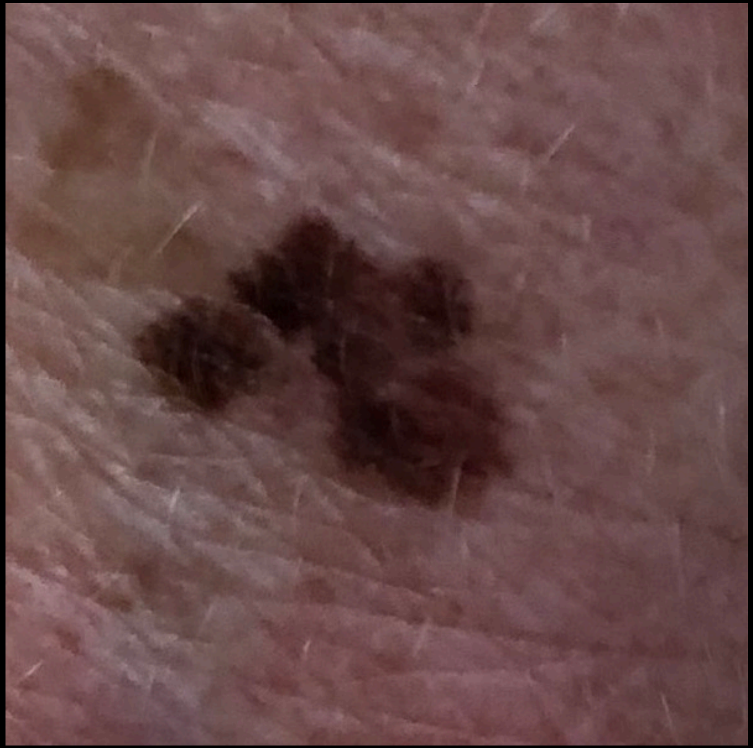

pT1a, 0.3 mm

Case number 263

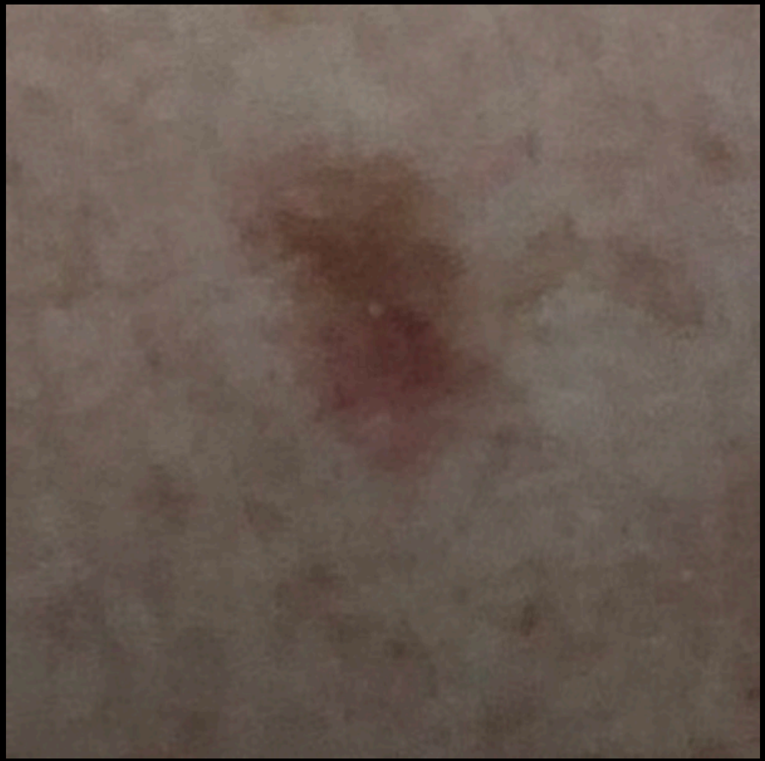

pTis

Case number 264

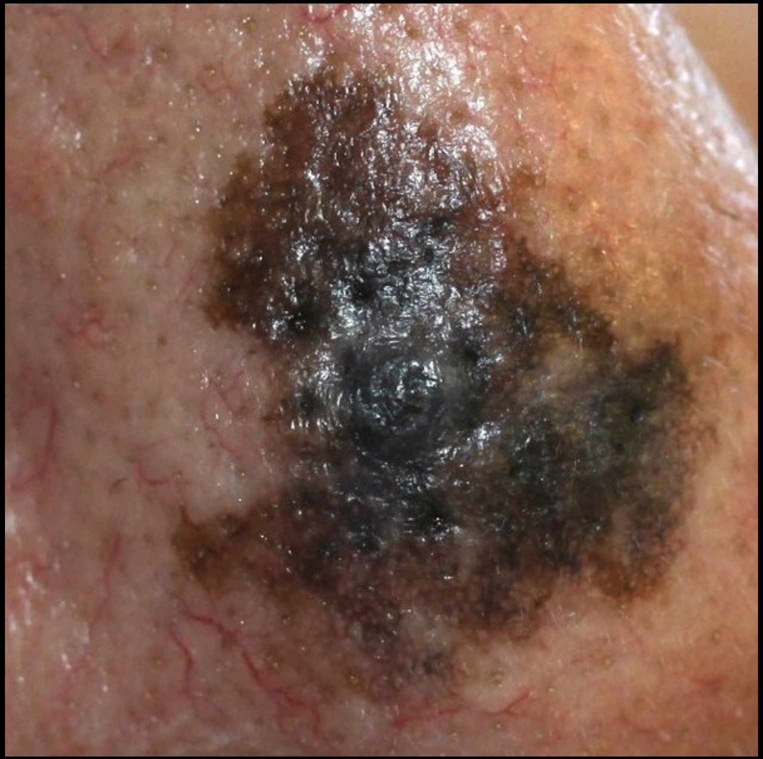

pT2a, 1.9 mm

Case number 265

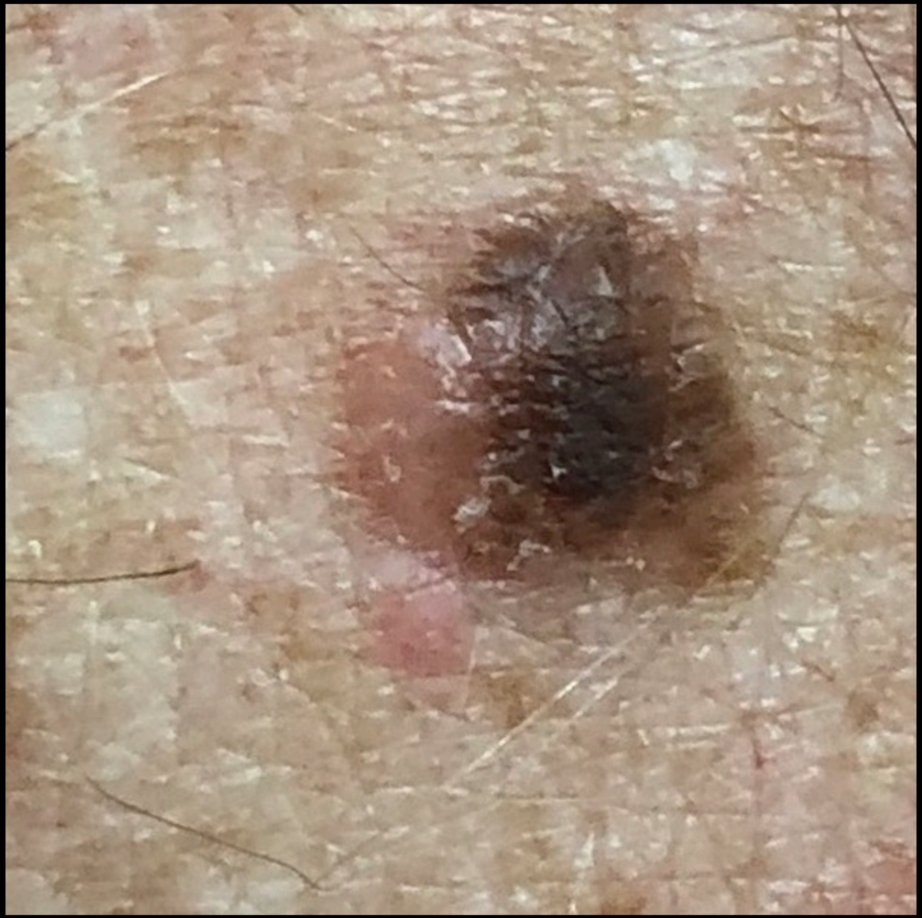

pTis

Case number 266

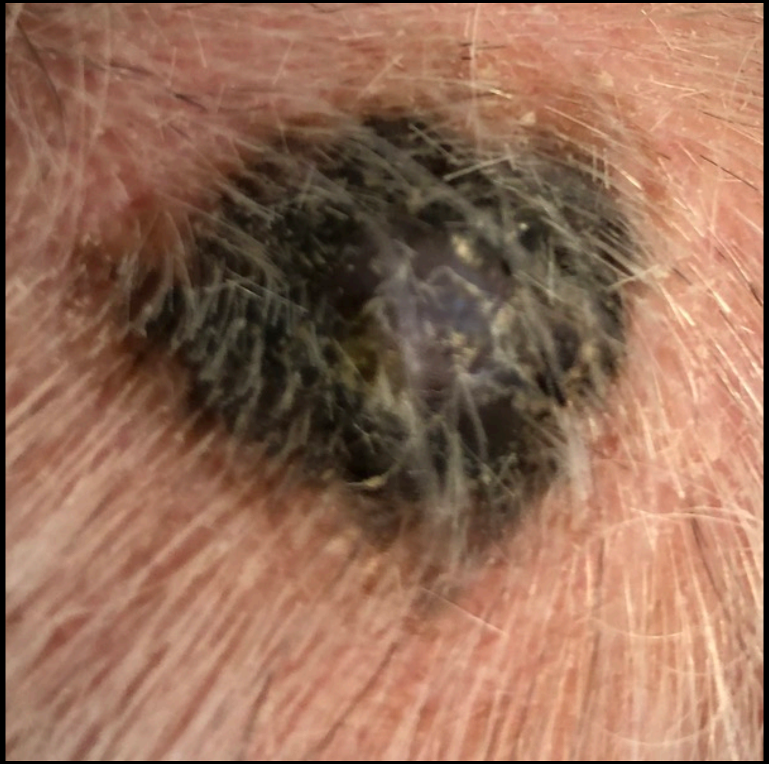

pT4a, 4.3 mm

Case number 267

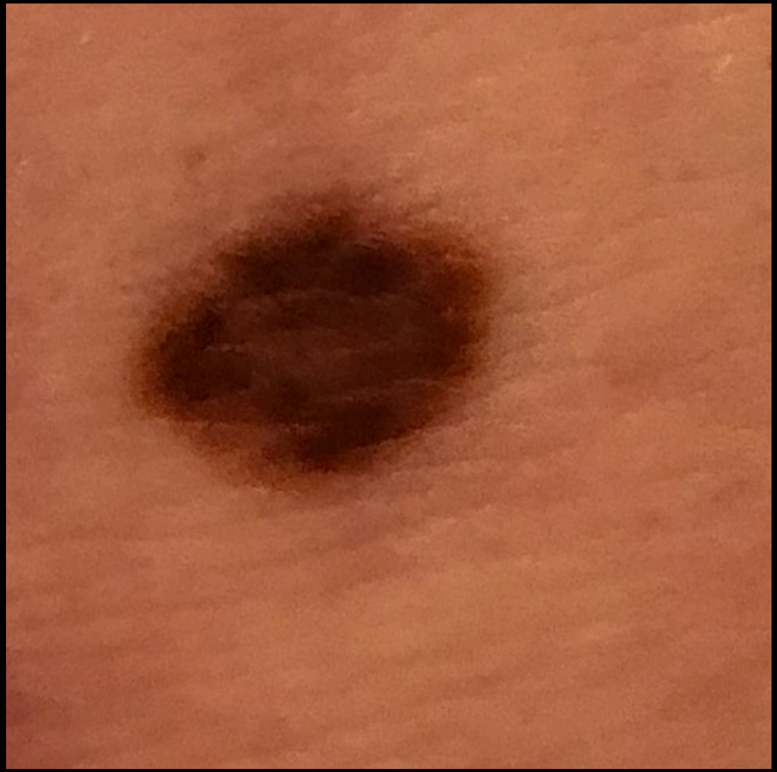

pTis

Case number 268

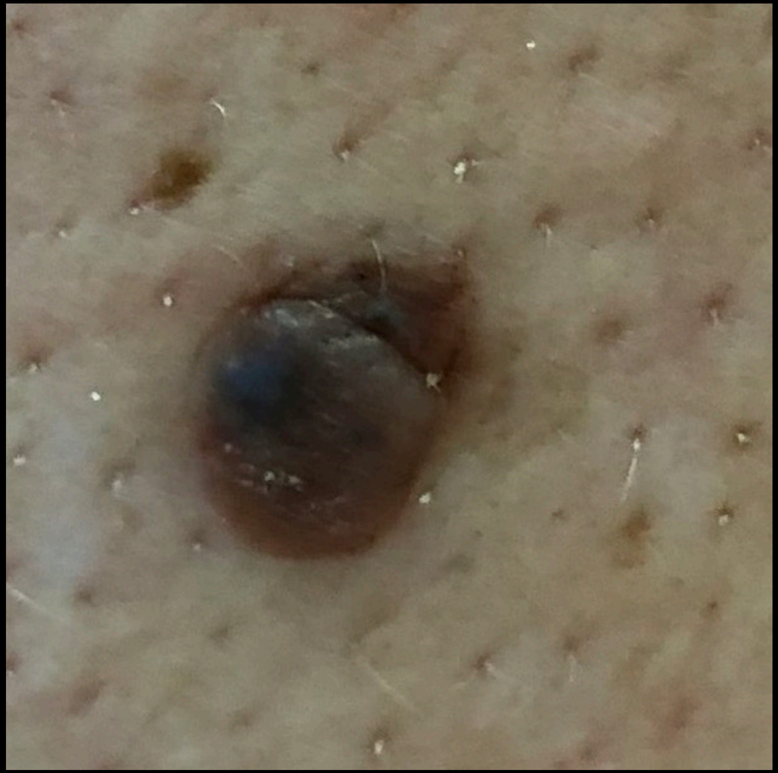

pT2a, 1.2 mm

Case number 269

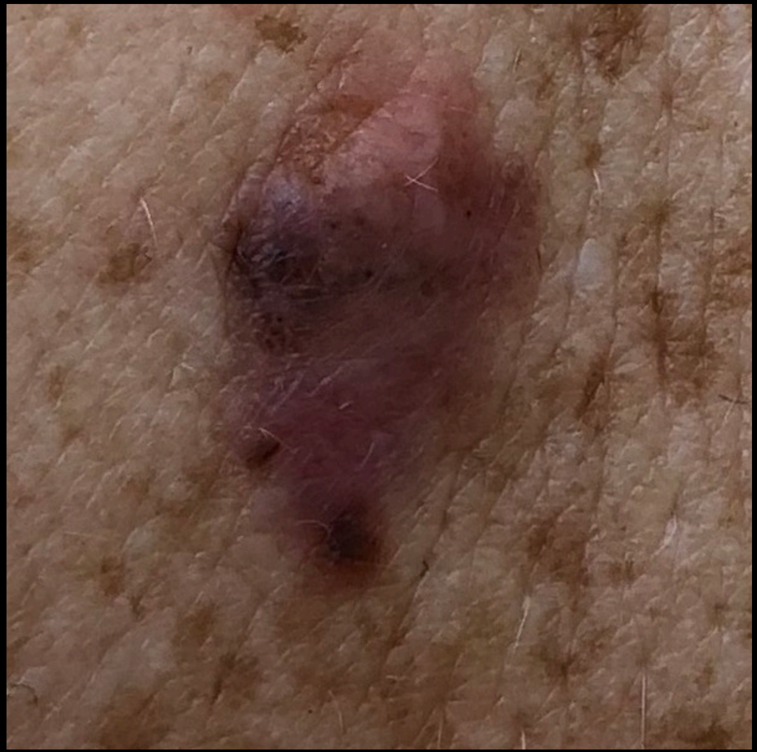

pT1b, 1.0 mm

Case number 270

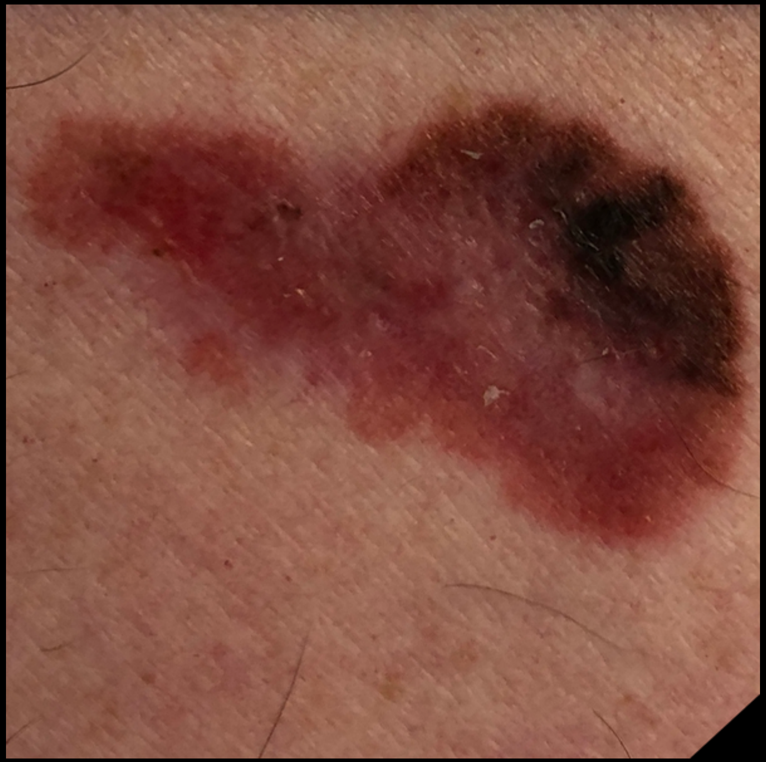

pT2a, 1.2 mm

Case number 271

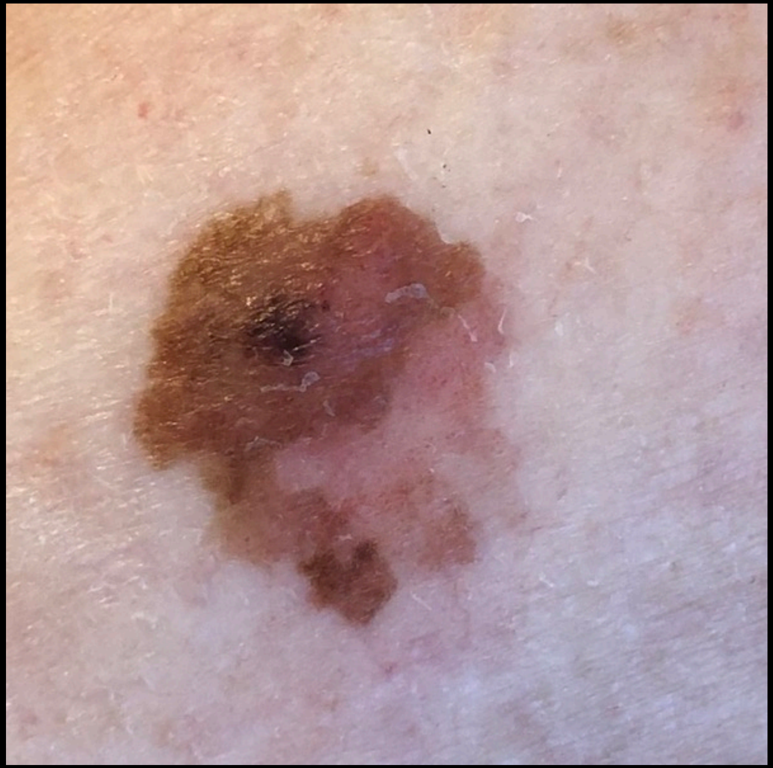

pT1a, 0.7 mm

Case number 272

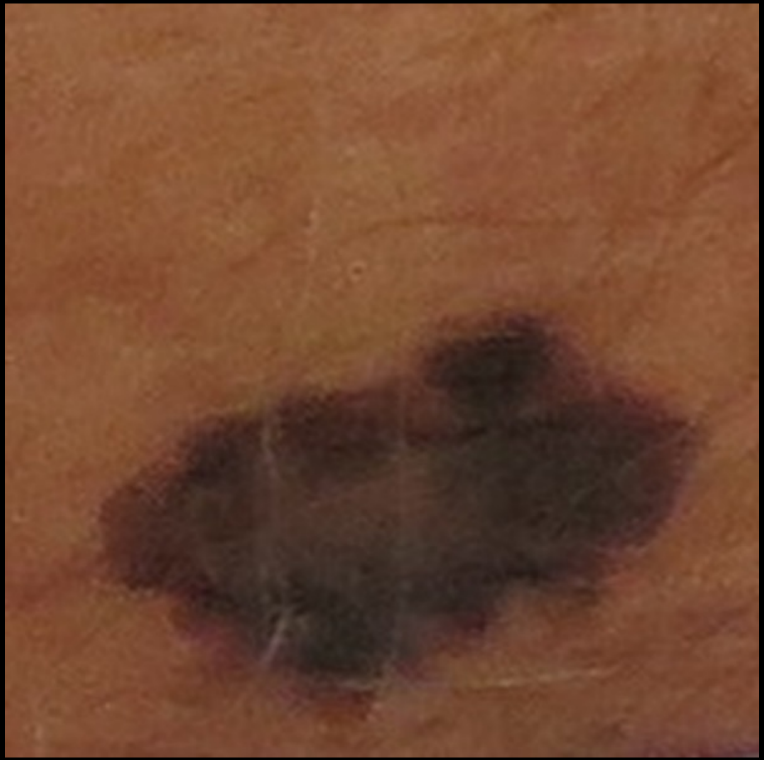

pT1b, 0.6 mm

Case number 273

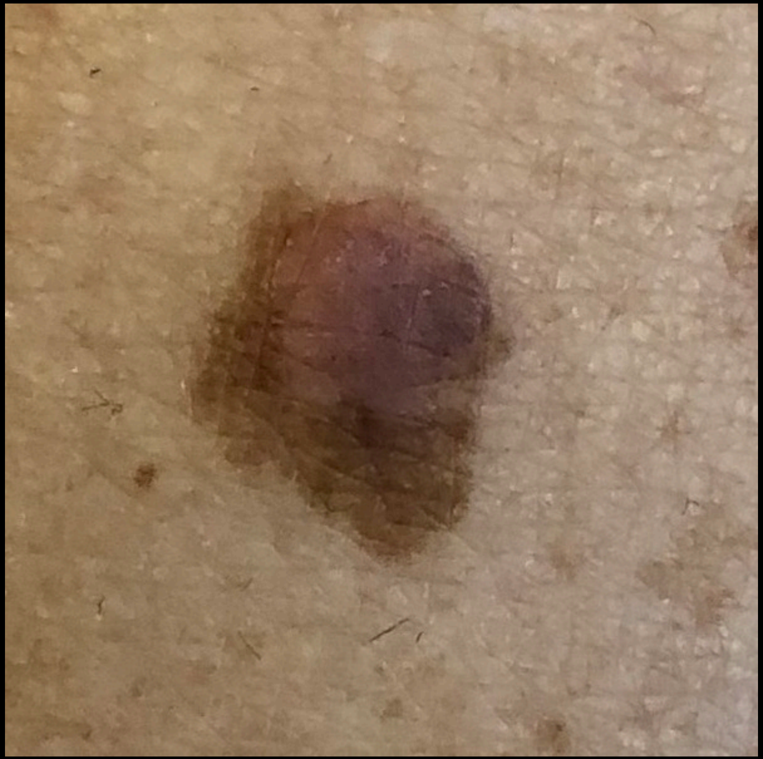

pT1a, 0.8 mm

Case number 274

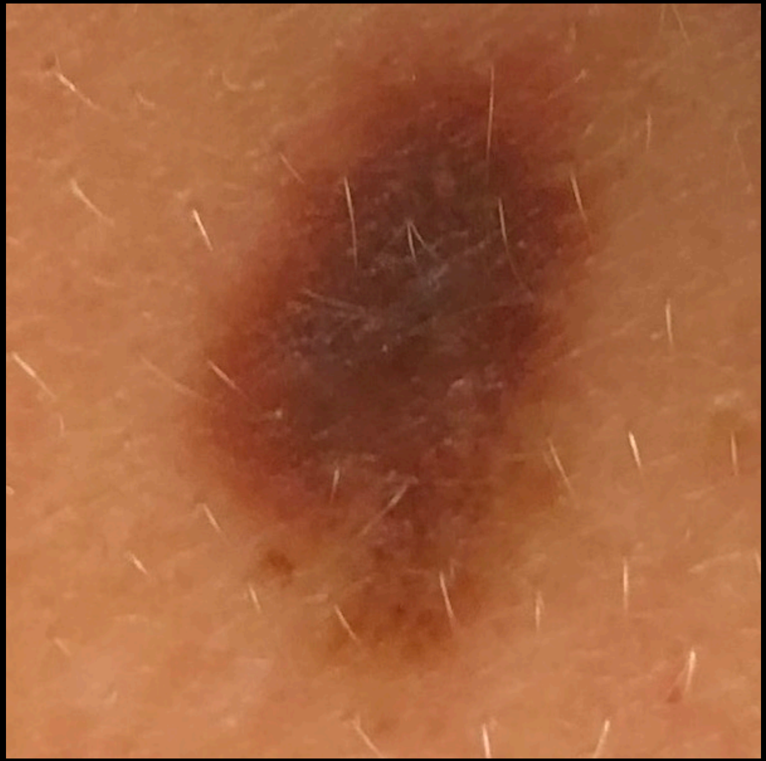

pTis

Case number 275

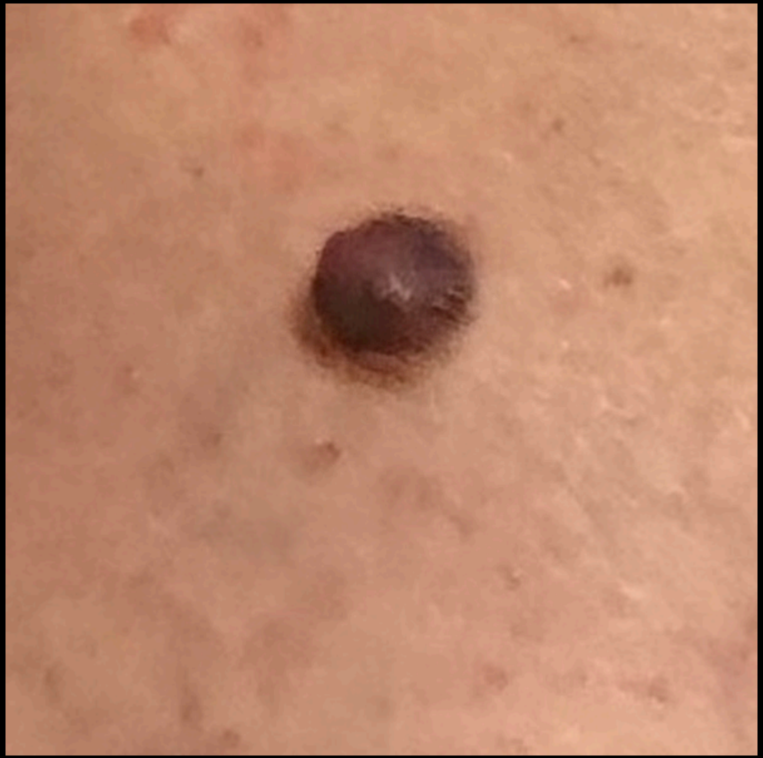

pT2a, 2.0 mm

Case number 276

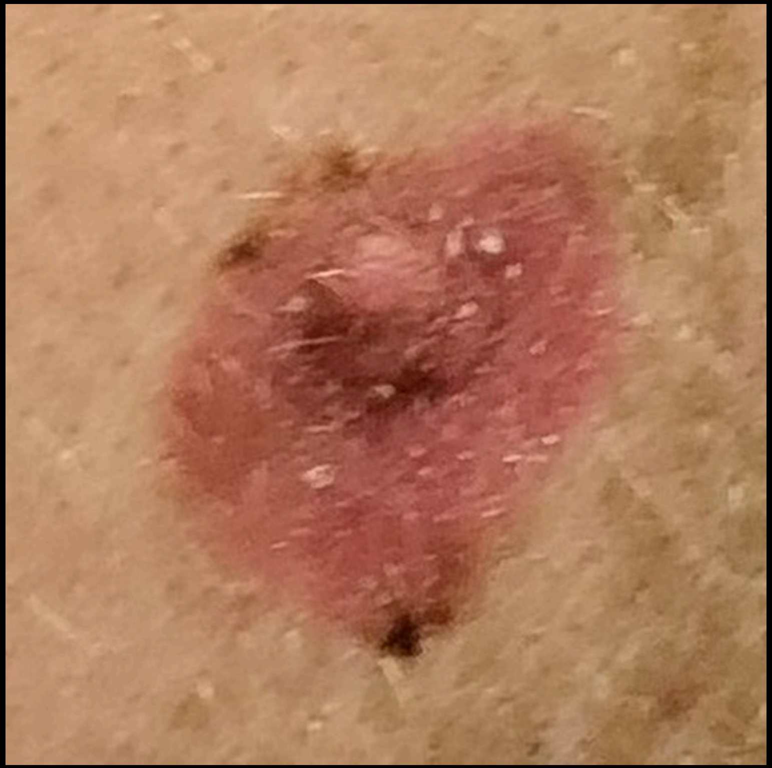

pT1a, 0.7 mm

Case number 277

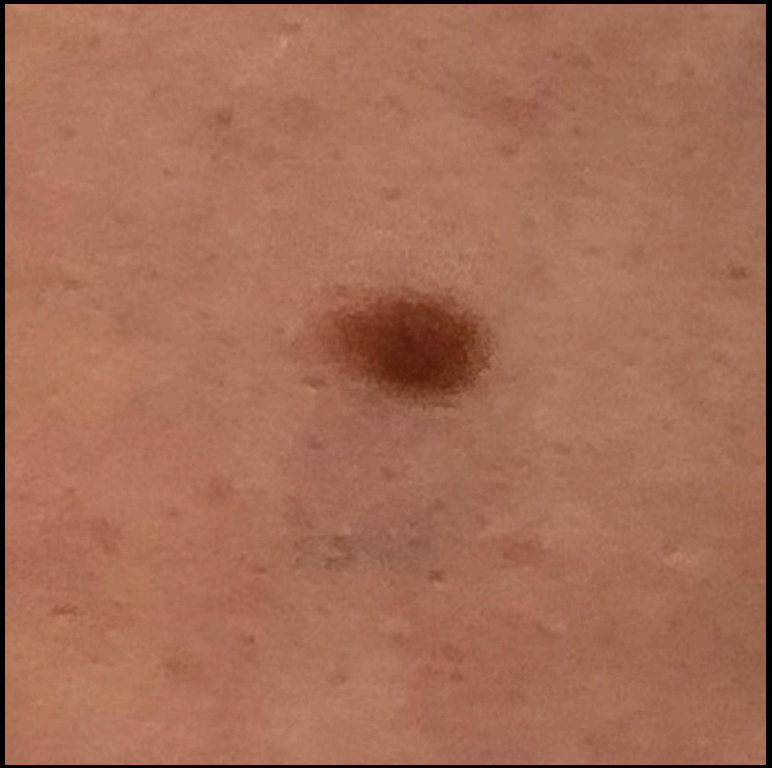

pTis

Case number 278

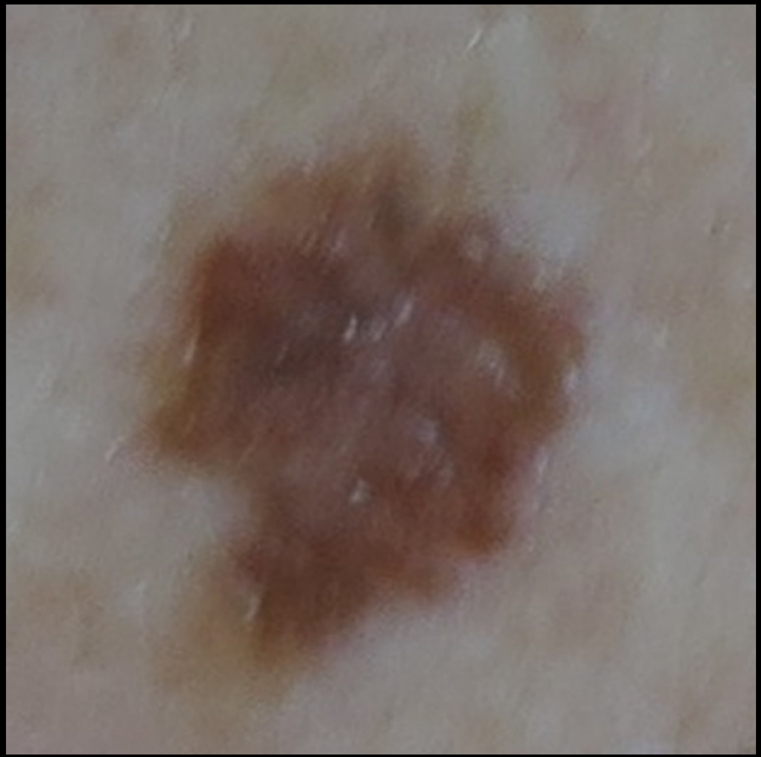

pTis

Case number 279

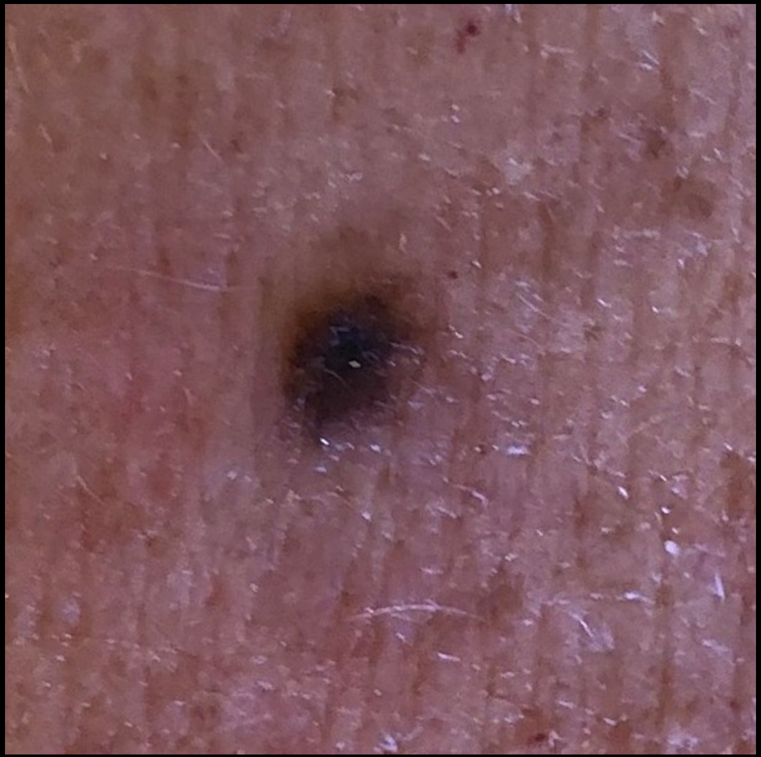

pTis

Case number 280

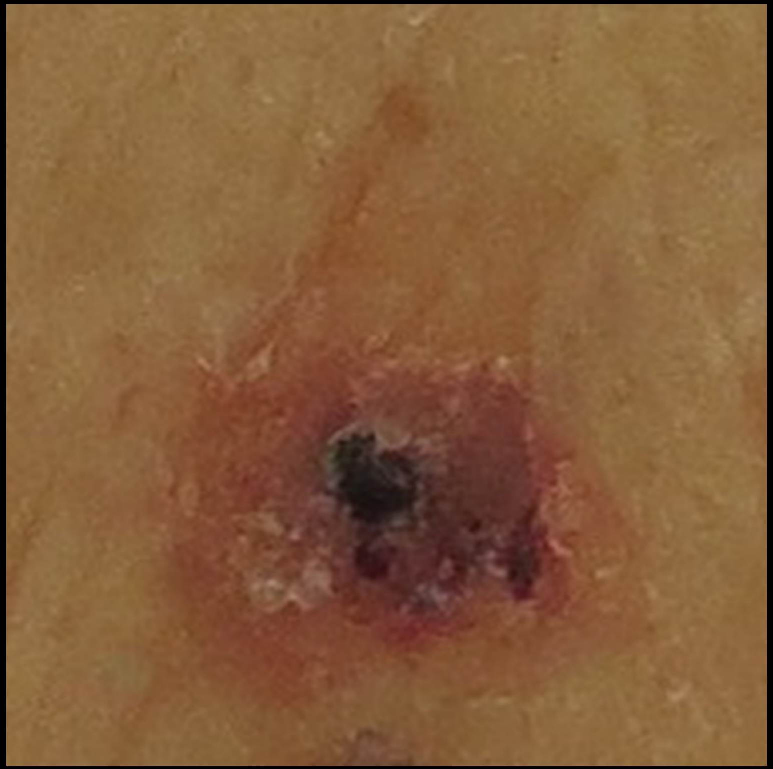

pT2a, 1.5 mm

Case number 281

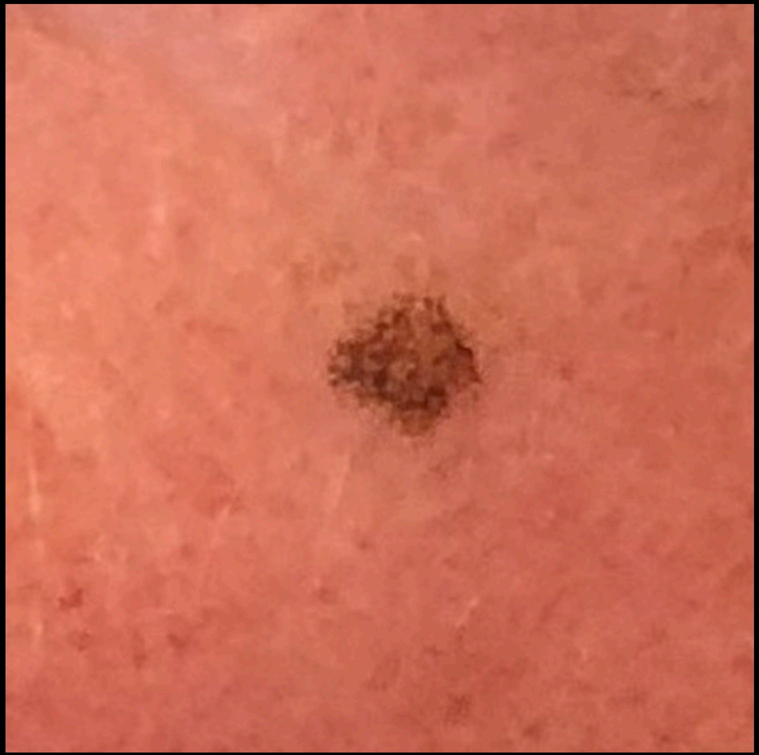

pTis

Case number 282

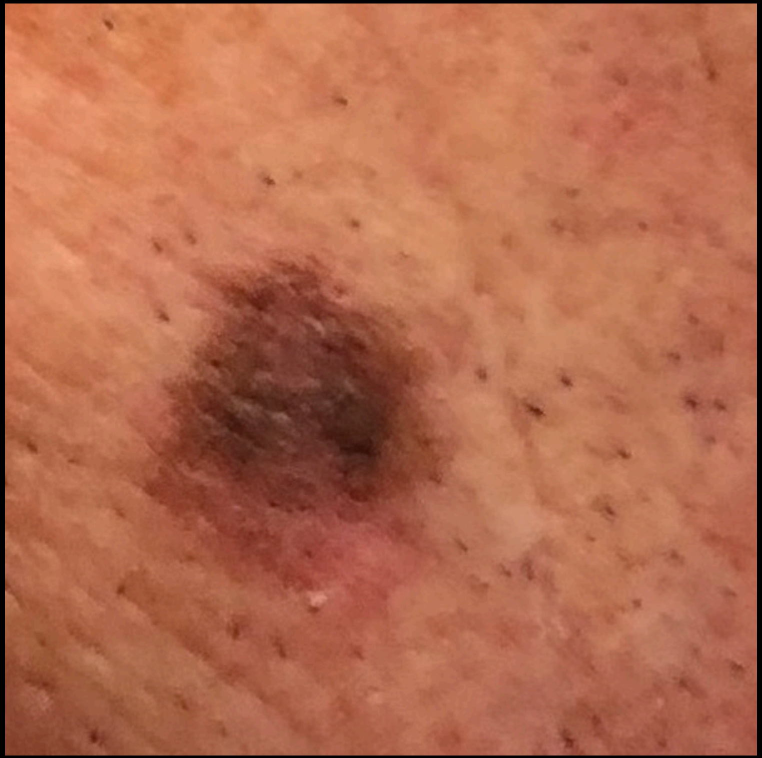

pT1a, 0.3 mm

Case number 283

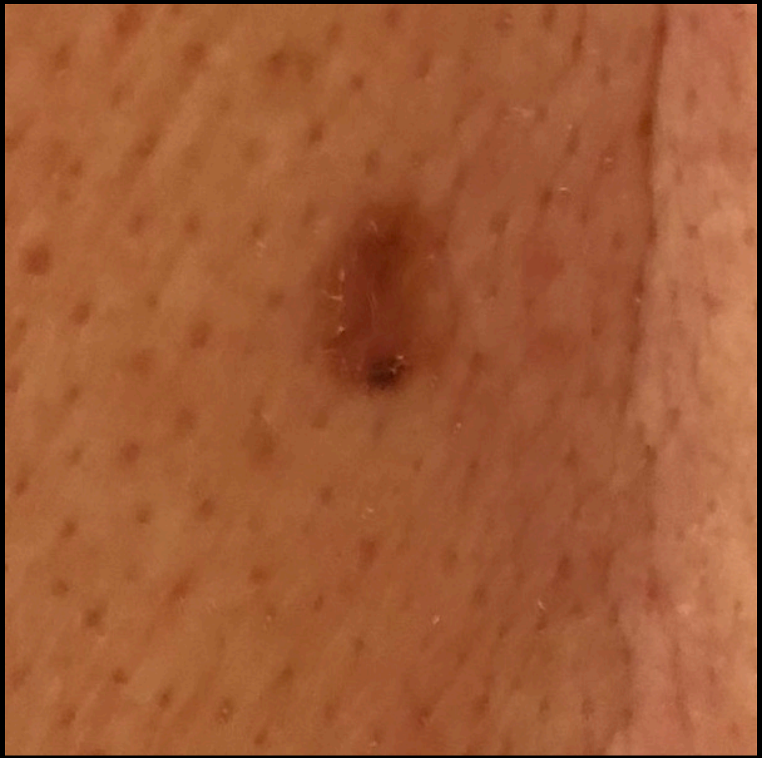

pTis

Case number 284

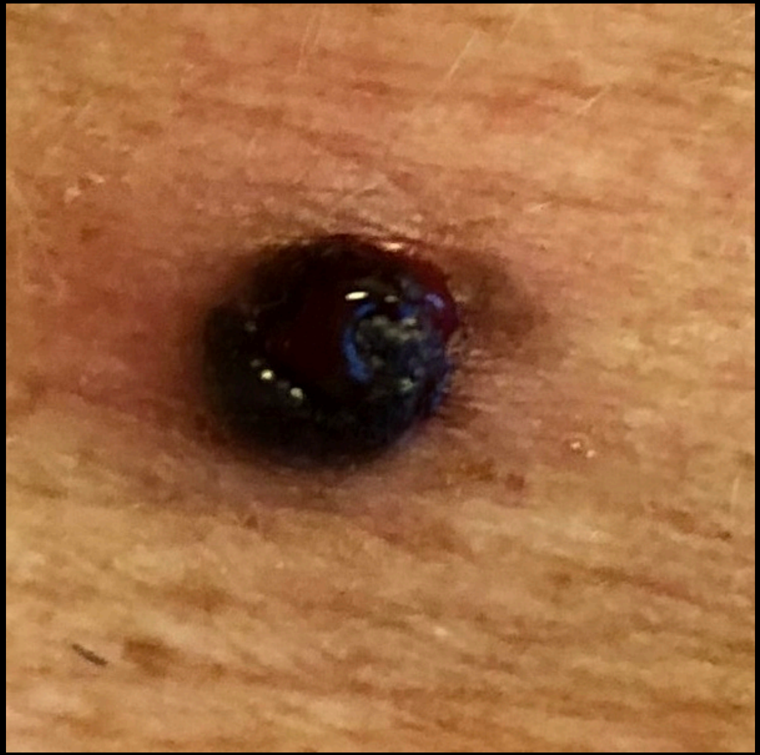

pT2a, 1.6 mm

Case number 285

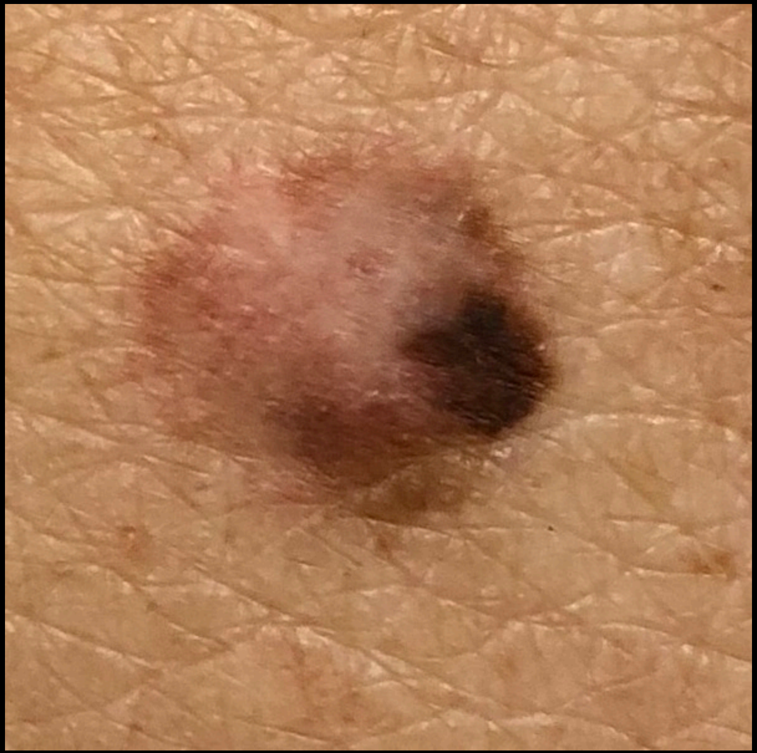

pTis

Case number 286

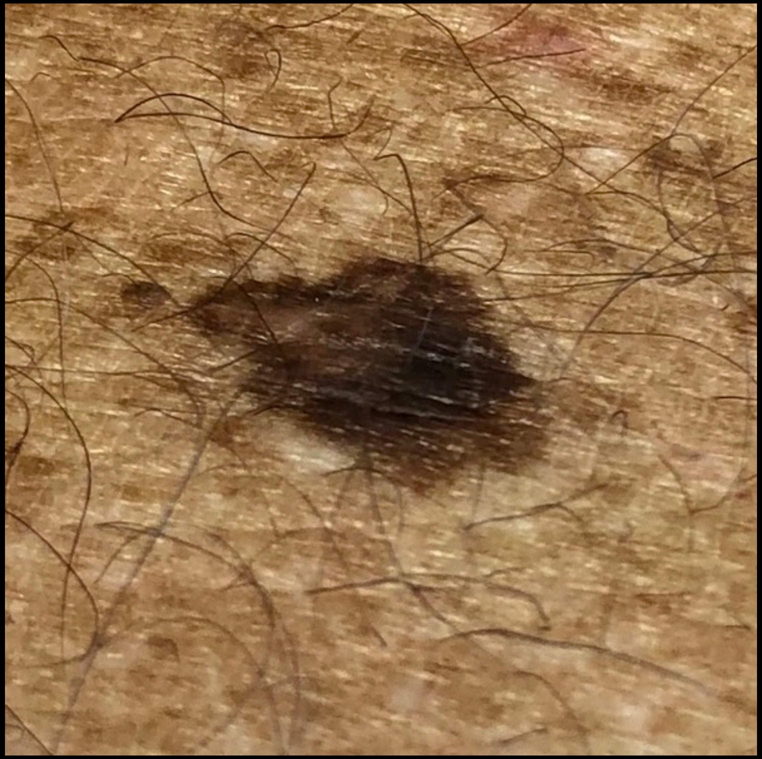

pTis

Case number 287

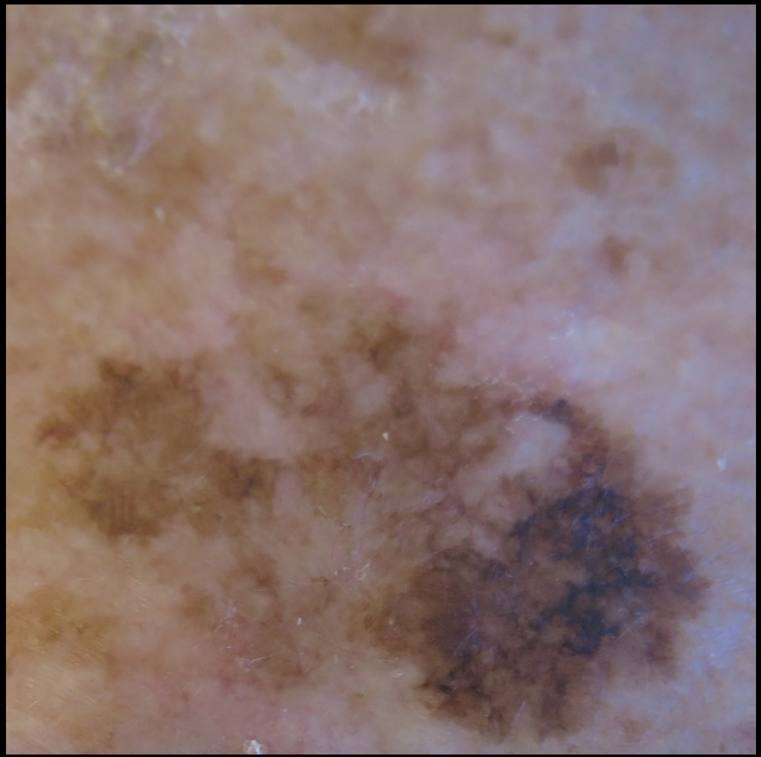

pTis

Case number 288

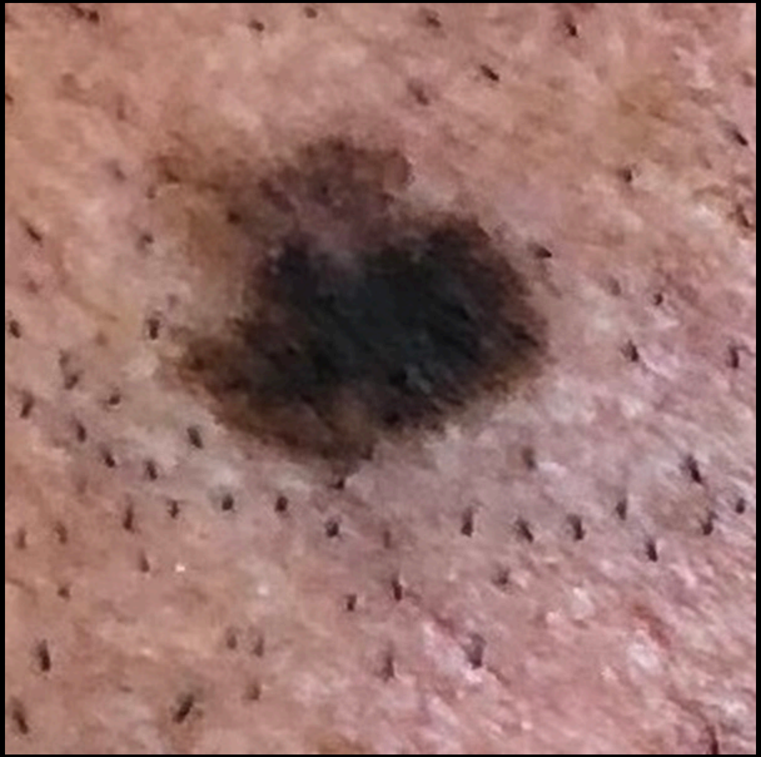

pT1a, 0.4 mm

Case number 289

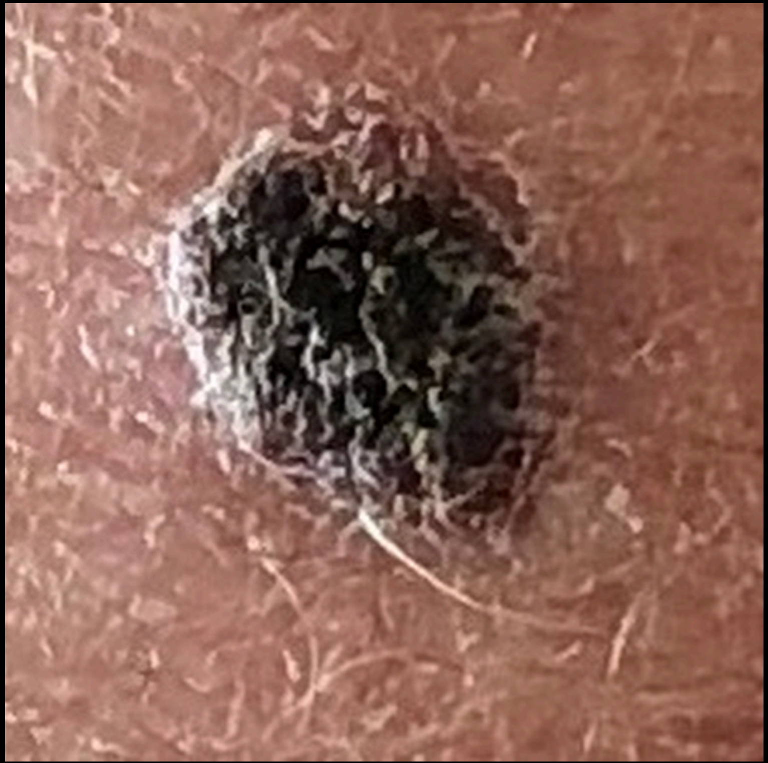

pT2b, 1.8 mm

Case number 290

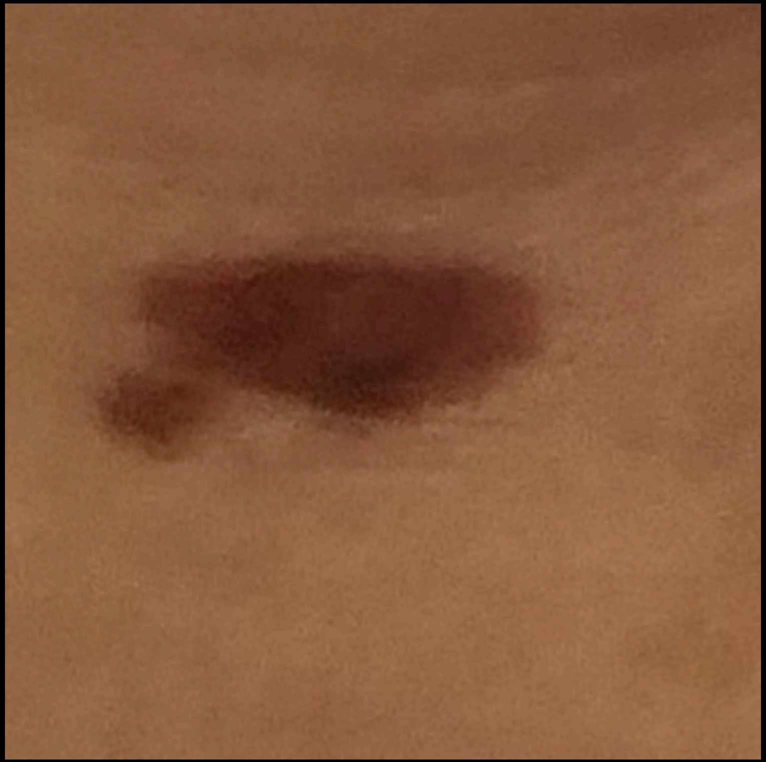

pTis

Case number 291

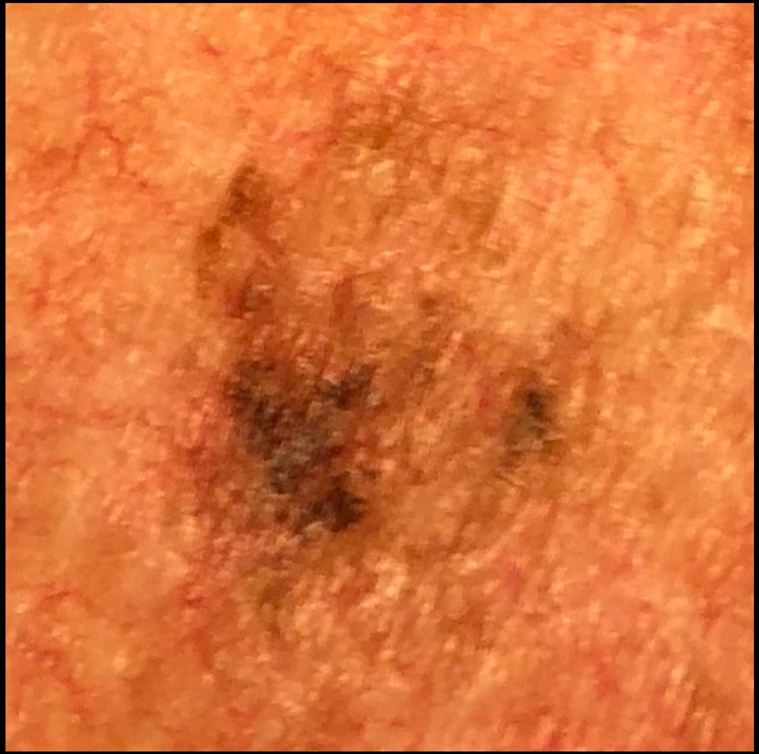

pTis

Case number 292

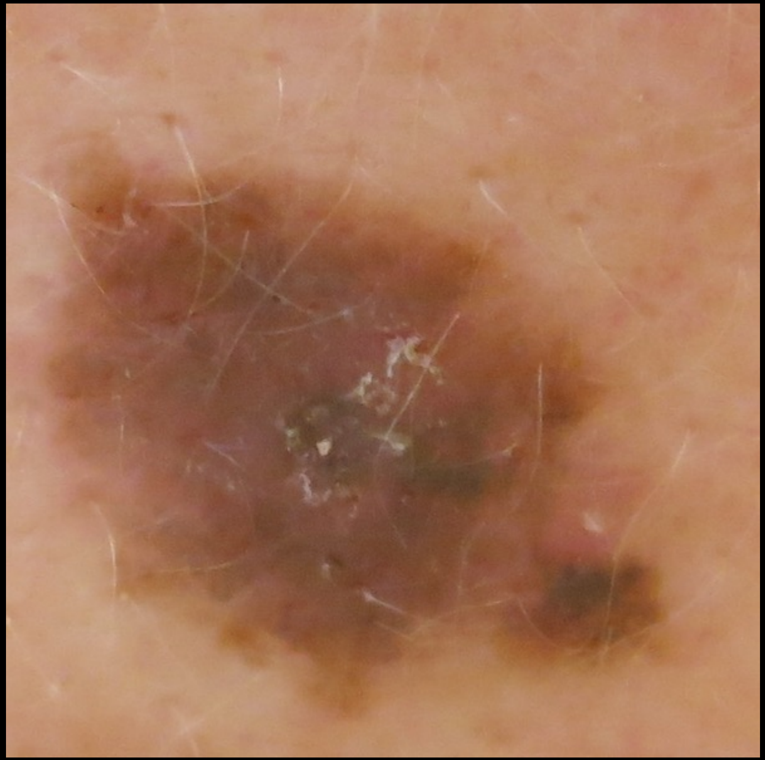

pT2b, 1.1 mm

Case number 293

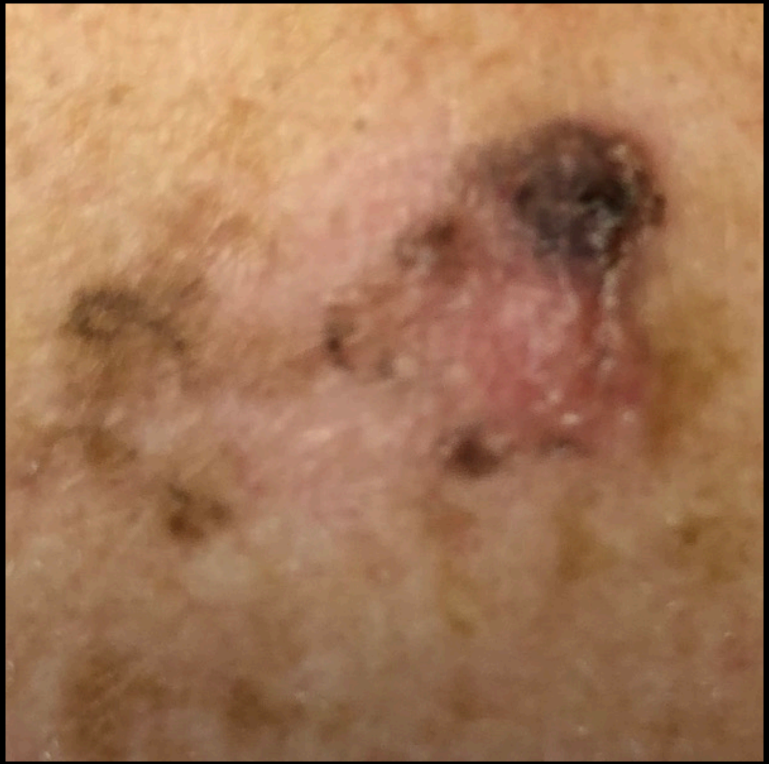

pT1b, 0.7 mm

Case number 294

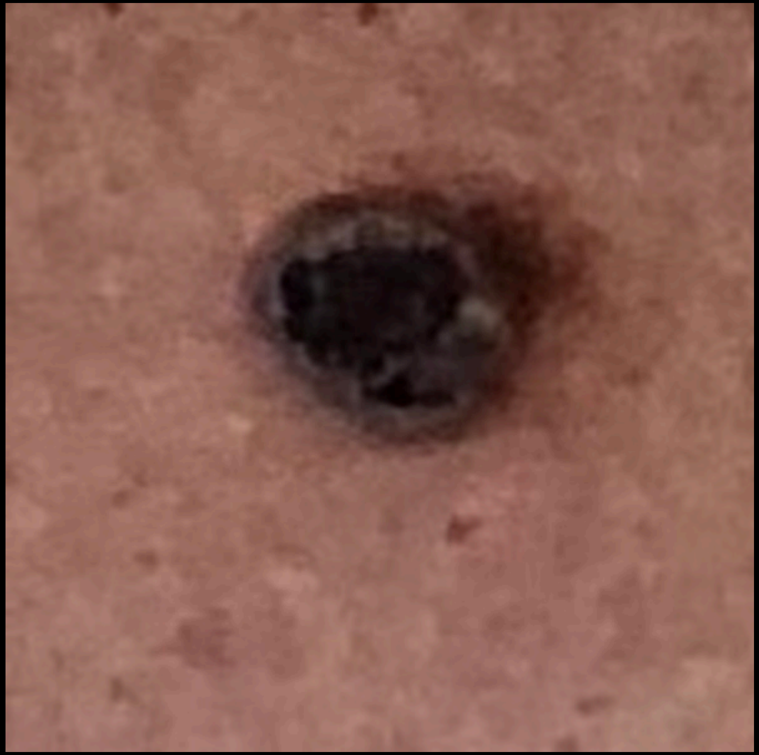

pT3b, 2.4 mm

Case number 295

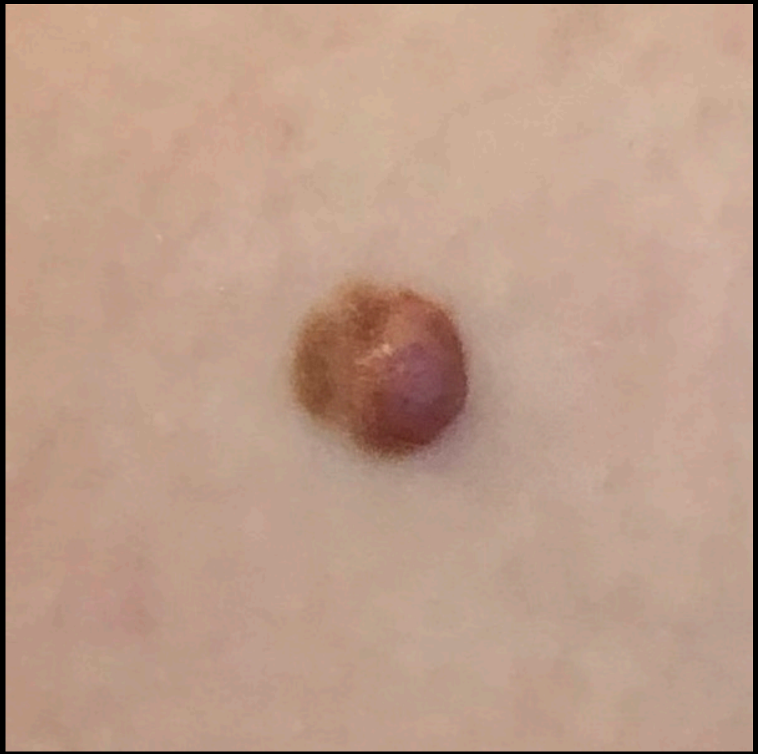

pT2a, 2.0 mm

Case number 296

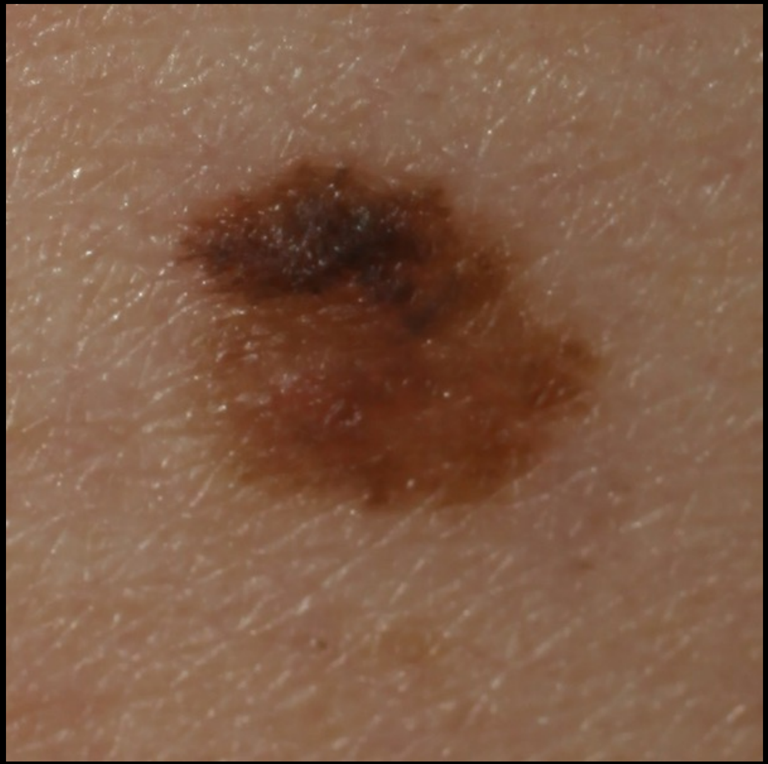

pT1a, 0.4 mm

Case number 297

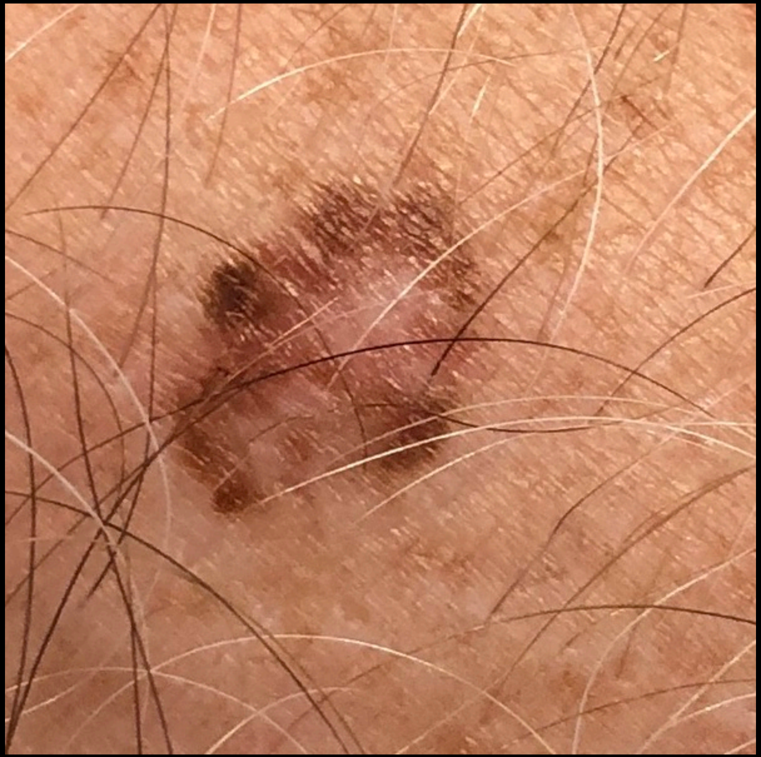

pTis

Case number 298

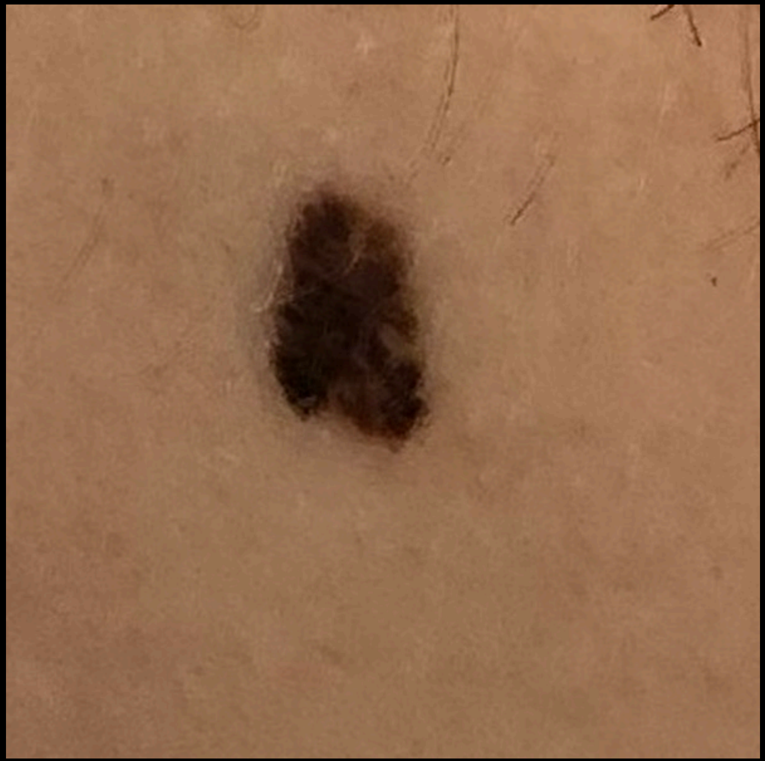

pTis

Case number 299

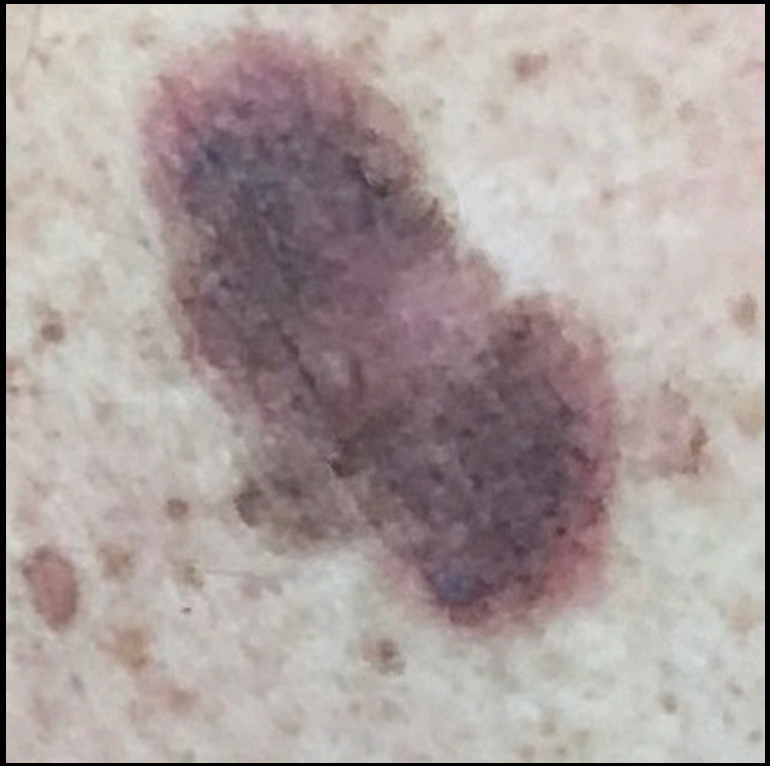

pT1a, 0.4 mm

Case number 300
